# Supplementary material for: DNA methylation and lncRNA control asynchronous DNA replication at specific imprinted gene domains
Source: Nat Commun. 2026 Jan 21;17:1844. doi: 10.1038/s41467-026-68558-2 (PMC12920997; doi:10.1038/s41467-026-68558-2)
Supplement: Supplementary file 1 — Supplementary Information [file 41467_2026_68558_MOESM1_ESM.pdf]

DNA methylation and lncRNA control asynchronous DNA replication at specific imprinted gene domains

Yui Imaizumi, François Charon, Caroline Surcis, Christel Picard, Pol Arnau-Romero, Jean-Christophe Andrau, Daan Noordermeer, Benoit Moindrot, Jean-Charles Cadoret and Robert Feil.

Supplementary Figures 1-8

Supplementary Tables 1-3

Supplementary References

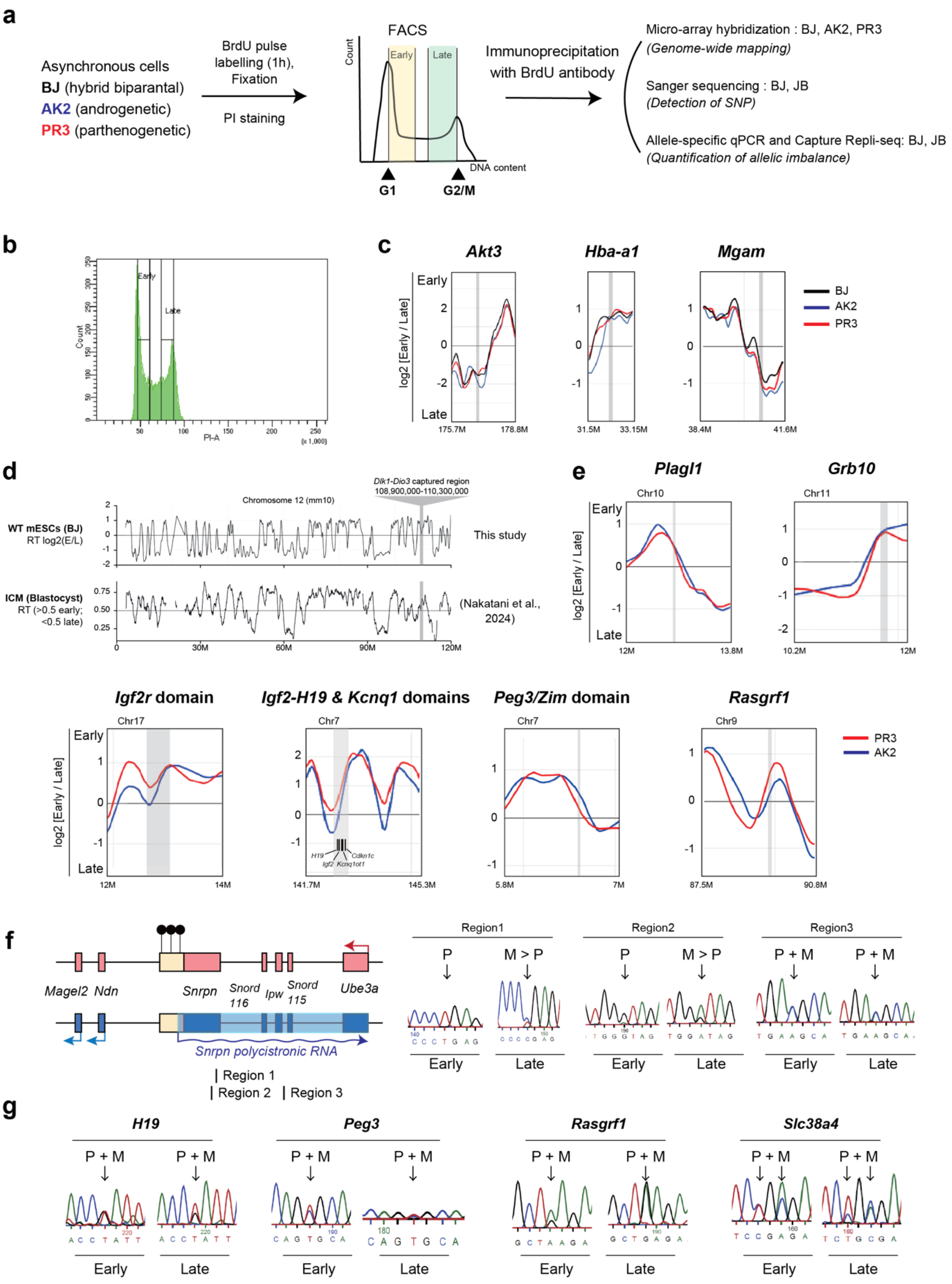
a
Asynchronous cells
BJ (hybrid biparantal)
AK2 (androgenetic)
PR3 (parthenogenetic)
BrdU pulse labelling (1h), Fixation
PI staining
FACS
Count
Early
Late
DNA content
G1
G2/M
Immunoprecipitation with BrdU antibody
Micro-array hybridization : BJ, AK2, PR3
(Genome-wide mapping)
Sanger sequencing : BJ, JB
(Detection of SNP)
Allele-specific qPCR and Capture Repli-seq: BJ, JB
(Quantification of allelic imbalance)
b
c
Akt3
Hba-a1
Mgam
BJ
AK2
PR3
log2 [Early / Late]
d
Chromosome 12 (mm10)
Dlk1-Dio3 captured region 108,900,000-110,300,000
WT mESCs (BJ)
RT log2(E/L)
This study
ICM (Blastocyst)
RT (>0.5 early; <0.5 late)
(Nakatani et al., 2024)
e
Plagl1
Grb10
Igf2r domain
Igf2-H19 & Kcnq1 domains
Peg3/Zim domain
Rasgrf1
f
Magel2
Ndn
Snrpn
Snord 116
Ipw
Snord 115
Ube3a
Snrpn polycistronic RNA
Region 1
Region 2
Region 3
Region1
Region2
Region3
P
M > P
P + M
Early
Late
g
H19
Peg3
Rasgrf1
Slc38a4

Supplementary Fig. 1. / Additional data related to genome-wide and locus-specific RT studies.

a, Schematic presentation of RT assays: Asynchronously growing cells were cultured for 1 hour in the presence of BrdU, ethanol-fixed, stained with propidium-iodide, and subjected to FACS to extract an early and a late cell cycle fraction. After DNA extraction and precipitation of BrdU-comprising DNA, samples were used for micro-array hybridization, allelic qPCR and Sanger sequencing, and for Capture Repli-seq. (see Methods). **b**, Representative FACS profile of fixed mESCs (*i.e.*, BJ cells) stained with propidium-iodide. **c,** Chromosomal RT profiles at representative active (*Hba-a1*) and repressed (*Akt3*, *Mgam*) genes obtained by array hybridisation in BJ, AK2 and PR3 mESCs. Vertical lines indicate positions of the genes indicated above the panels. **d,** RT profile of chromosome 12 in WT mESCs (BJ strain), aligned with the RT profile of chromosome 12 in the inner cell mass (ICM) of the mouse blastocyst (raw RT signal; data published in Nakatani *et al*, 2024 [1]). The grey highlight indicates the genomic window comprising the *Dlk1-Dio3* domain, explored by Capture Repli-seq and Hi-C in this study. **e,** RT profiles at selected imprinted gene loci in AK2 and PR3 mESCs. Vertical rectangles indicate the extent of the imprinted loci. **f,** Schematic presentation of the *Snrpn* domain, with the regions analysed by PCR-Sanger sequencing. Rectangles indicate genes on the maternal (red) and the paternal (blue) chromosome, with their expression status (arrows) in the embryo. The allelic gene expression is controlled by the locus' ICR (beige rectangle), which is methylated (black lollipops) on the maternal chromosome. To the right, Sanger sequencing profiles at *Snrpn* regions 1 and 2 indicate early replication on the paternal, and a trend towards late replication on the maternal chromosome. A third polymorphic region, at *Ipw* (region 3) does not show asynchronous replication. **g**, PCR- and Sanger sequencing-based assessment of RT in BJ mESCs at the imprinted *H19*, *Peg3*, *Rasgrf1* and *Slc38a4* genes. No overt allelic differences are detected at these loci in the early and/or late fractions.

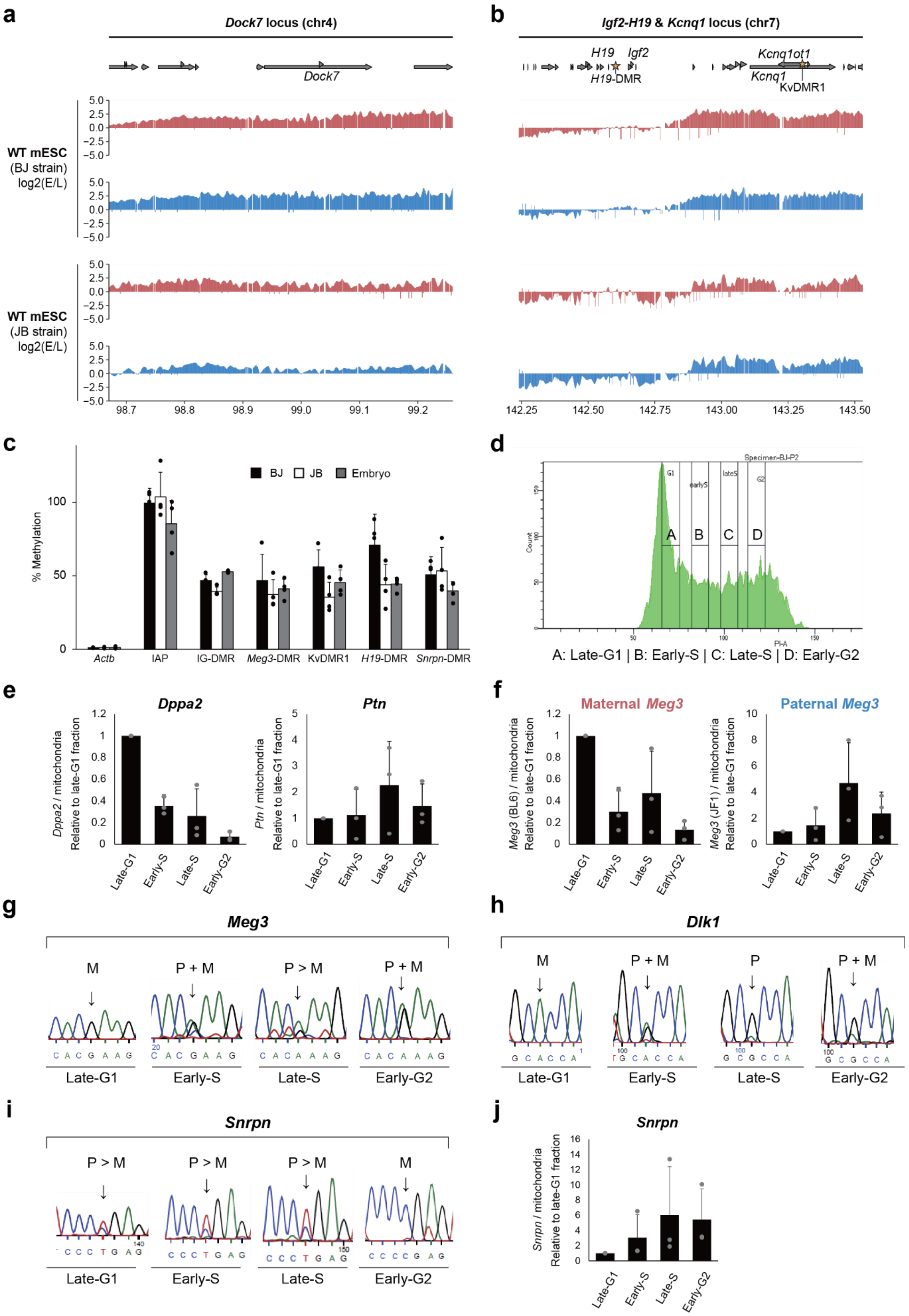
a
Dock7 locus (chr4)
Dock7
WT mESC (BJ strain) log2(E/L)
WT mESC (JB strain) log2(E/L)
5.0 2.5 0.0 −2.5 −5.0
98.7 98.8 98.9 99.0 99.1 99.2
b
Igf2-H19 & Kcnq1 locus (chr7)
H19 Igf2
H19-DMR
Kcnq1ot1
Kcnq1
KvDMR1
142.25 142.50 142.75 143.00 143.25 143.50
c
% Methylation
BJ JB Embryo
Actb IAP IG-DMR Meg3-DMR KvDMR1 H19-DMR Snrpn-DMR
d
Specimen-BJ-P2
G1 earlyS lateS G2
A B C D
Count
PI-A
A: Late-G1 | B: Early-S | C: Late-S | D: Early-G2
e
Dppa2
Dppa2 / mitochondria Relative to late-G1 fraction
Ptn
Ptn / mitochondria Relative to late-G1 fraction
Late-G1 Early-S Late-S Early-G2
f
Maternal Meg3
Meg3 (BL6) / mitochondria Relative to late-G1 fraction
Paternal Meg3
Meg3 (JF1) / mitochondria Relative to late-G1 fraction
g
Meg3
M P + M P > M P + M
Late-G1 Early-S Late-S Early-G2
h
Dlk1
M P + M P P + M
Late-G1 Early-S Late-S Early-G2
i
Snrpn
P > M P > M P > M M
Late-G1 Early-S Late-S Early-G2
j
Snrpn
Snrpn / mitochondria Relative to late-G1 fraction
Late-G1 Early-S Late-S Early-G2

Supplementary Fig. 2. / Further dissection of allelic RT at imprinted chromosomal domains.

a, Capture Repli-seq on a non-imprinted control region on chromosome 4, in WT (BJ and JB strains) mESCs. Maternal (in red) and paternal (in blue) chromosome enrichments are shown. Gene positions are aligned above. **b,** Capture Repli-seq on distal chromosome 7, across a region comprising the imprinted *Igf2-H19* and *Kcnq1* domains, in BJ and JB mESCs. Maternal (in red) and paternal (in blue) chromosome enrichments are shown. Gene positions are aligned above; asterisks indicate the *H19*-DMR and the KvDMR1 ICRs. **c,** Methylation-sensitive qPCR in BJ and JB mESCs and E13 embryo at the IG-DMR, *Meg3*-DMR, KvDMR1 (*Kcnq1* domain), *H19*-DMR (*Igf2-H19* domain), *Snrpn*-DMR (*Snrpn* domain), *actin-B* promoter (low-methylation control) and *IAP*s (high-methylation control). Bars represent means ± SD from 4 independent experiments. **d,** Representative FACS profile of mESCs (*i.e.*, BJ cells), with fractionation into four cell-cycle fractions: late-G1 (A), early-S (B), late-S (C), early-G2 (D). **e,** RT at *Dppa2* and *Ptn* determined by qPCR in the four fractions in BJ mESCs. Bars represent means ± SD from 3 independent experiments. **f,** RT at *Meg3* on the maternal (left) and the paternal chromosome (right) analysed by allele-specific qPCR in the four fractions in BJ mESCs. Bars represent means ± SD from 3 independent experiments. **g,h,i,** Sanger-sequencing-based assessment of allelic RT at *Meg3* (g), *Dlk1* (h) and *Snrpn* (i) in the four fractions in BJ mESCs. Arrows indicate SNPs used to distinguish the maternal (M) and paternal (P) chromosome. **j**, Overall quantification shows that *Snrpn* replicates mostly in late S and early-G2. Bars represent means ± SD from 3 independent experiments.

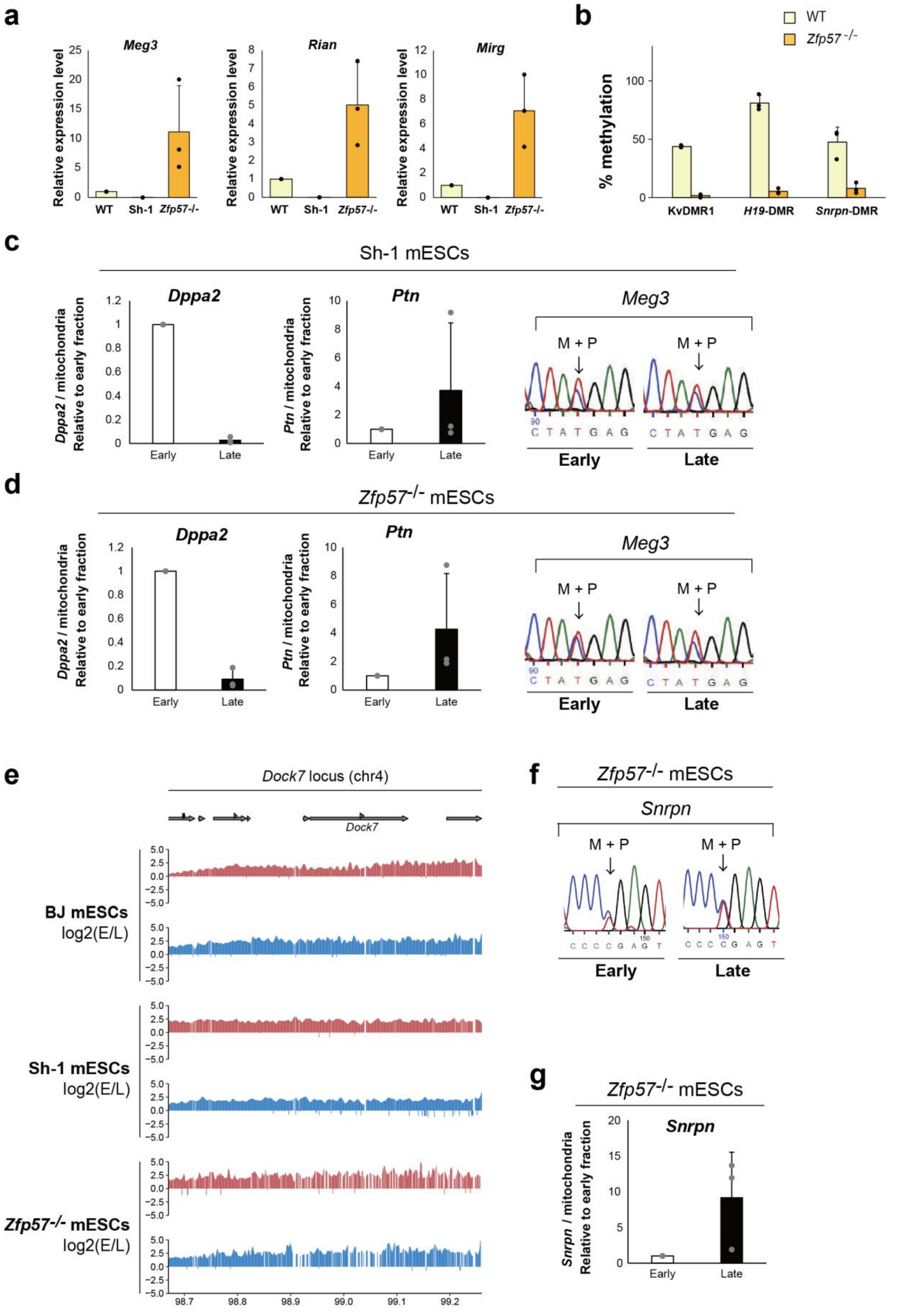
a
Meg3
Rian
Mirg
Relative expression level
WT
Sh-1
Zfp57-/-
b
WT
Zfp57 -/-
% methylation
KvDMR1
H19-DMR
Snrpn-DMR
c
Sh-1 mESCs
Dppa2
Ptn
Meg3
Dppa2 / mitochondria
Relative to early fraction
Ptn / mitochondria
Relative to early fraction
Early
Late
M + P
d
Zfp57-/- mESCs
Dppa2
Ptn
Meg3
M + P
Early
Late
e
Dock7 locus (chr4)
Dock7
BJ mESCs
log2(E/L)
Sh-1 mESCs
log2(E/L)
Zfp57-/- mESCs
log2(E/L)
f
Zfp57-/- mESCs
Snrpn
M + P
Early
Late
g
Zfp57-/- mESCs
Snrpn
Snrpn / mitochondria
Relative to early fraction
Early
Late

Supplementary Fig. 3. / Additional gene expression and RT data on Sh-1 and *Zfp57*$^{-/-}$ mESCs.

a, Expression levels of Meg3, Rian and Mirg RNA relative to Gapdh in WT (BJ), Sh-1 and *Zfp57*$^{-/-}$ mESCs, determined by RT-qPCR. Bars represent means ± SD from 3 independent experiments. **b,** Methylation qPCR analysis in *Zfp57*$^{-/-}$ and BJ mESCs, of the KvDMR1, *H19*-DMR and *Snrpn*-DMR. Bars represent means ± S.D. from 3 experiments. **c,** Left, qPCR assessment in early and late fractions of *Dppa2* (early replication) and *Ptn* (late replication) in Sh-1 mESCs. Bars represent means ± S.D. from 3 experiments. Right, Sanger-sequencing-based allelic assessment of RT at *Meg3* in the early and late fractions. Arrows indicate a SNP used for parental allele discrimination. **d,** Same as for c, but now in *Zfp57*$^{-/-}$ cells. **e,** Capture Repli-seq on the chromosome-4 control region in WT, Sh-1 and *Zfp57*$^{-/-}$ mESCs. Maternal (red) and paternal (blue) chromosome-specific enrichments are plotted. Gene positions are aligned above. **f,** Sanger-sequencing-based allelic assessment of RT at *Snrpn* region 1 (see Supplementary Fig. 1f) in *Zfp57*$^{-/-}$ mESCs. The parental alleles are equally present in the early and late fractions. **g**, Overall qPCR quantification shows late replication mostly in *Zfp57*$^{-/-}$ mESCs. Bars represent means ± S.D. from 3 experiments.

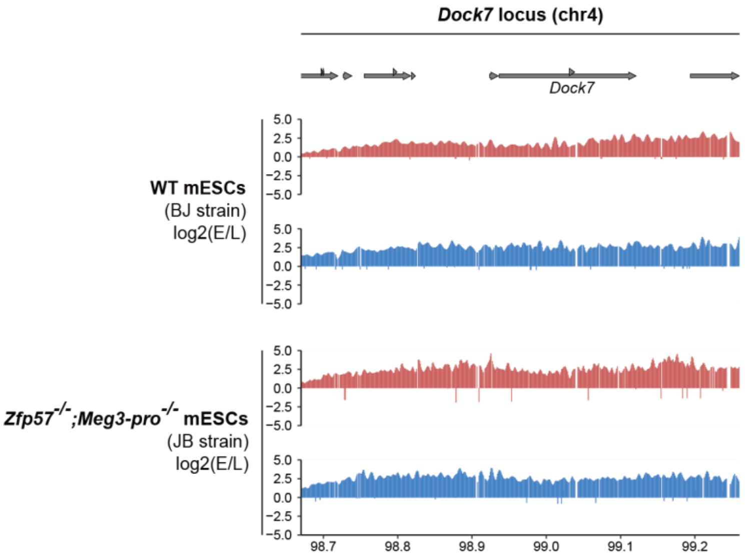

Supplementary Figure 4. / Additional RT data on *Zfp57*$^{-/-}$;*Meg3*-pro$^{-/-}$ mESCs.

Capture Repli-seq on the chromosome-4 *Dock7* control region in WT mESCs (top panel) and *Zfp57*$^{-/-}$;*Meg3*-pro$^{-/-}$ mESCs (lower panel). Maternal (red) and paternal (blue) chromosome-specific enrichments are plotted. Gene positions are aligned above.

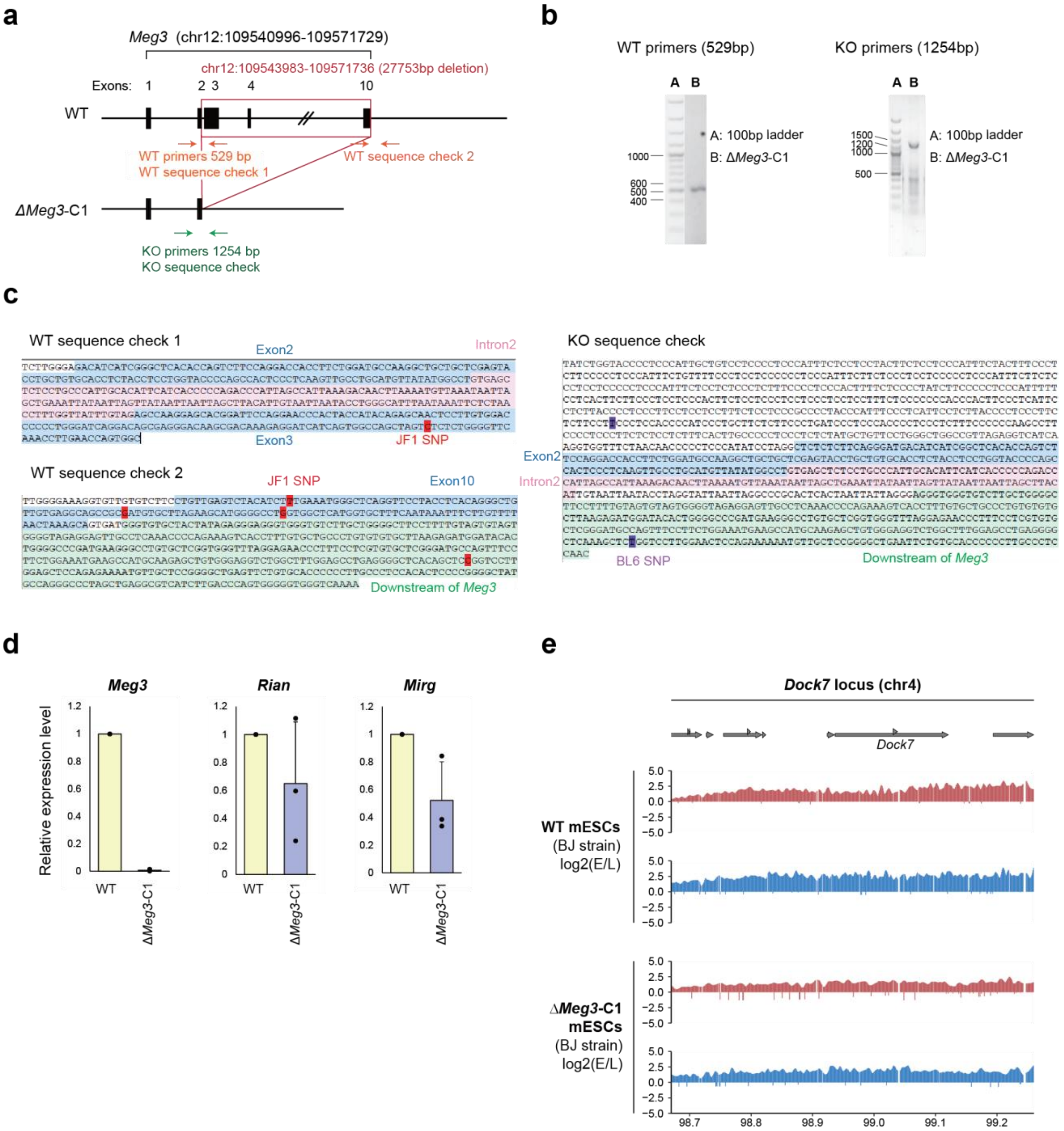
a
Meg3 (chr12:109540996-109571729)
chr12:109543983-109571736 (27753bp deletion)
Exons: 1 2 3 4 10
WT
WT primers 529 bp
WT sequence check 1
WT sequence check 2
ΔMeg3-C1
KO primers 1254 bp
KO sequence check
b
WT primers (529bp)
KO primers (1254bp)
A B
A: 100bp ladder
B: ΔMeg3-C1
1000
600
500
400
1500
1200
1000
500
c
WT sequence check 1
Exon2
Intron2
Exon3
JF1 SNP
WT sequence check 2
JF1 SNP
Exon10
Downstream of Meg3
KO sequence check
Exon2
Intron2
BL6 SNP
Downstream of Meg3
d
Meg3
Rian
Mirg
Relative expression level
1.2
1
0.8
0.6
0.4
0.2
0
WT
ΔMeg3-C1
e
Dock7 locus (chr4)
Dock7
WT mESCs
(BJ strain)
log2(E/L)
ΔMeg3-C1
mESCs
(BJ strain)
log2(E/L)
5.0
2.5
0.0
-2.5
-5.0
98.7
98.8
98.9
99.0
99.1
99.2

Supplementary Figure 5. / Genotyping and additional data on Δ*Meg3*-C1 mESCs.

a, Schematic presentation of the *Meg3* gene, which comprises 10 exons (black rectangles). The deleted region is indicated, as well as the primer pairs for the genotyping. **b**, PCR primers to detect WT Meg3 amplify a 529-bp fragment in Δ*Meg3*-C1 mESCs, indicating the presence of the WT gene. A PCR product of 1254-bp, obtained with primers to detect the deletion, is obtained in Δ*Meg3*-C1 mESCs as well. We conclude that the deletion in hemizygous. **c**, Sanger sequencing of PCR products indicates that the maternal *Meg3* allele (BL6) is deleted in Δ*Meg3*-C1 mESCs. Exonic sequences are in blue, intronic sequences in pink, and sequences downstream of *Meg3* in green. **d,** Expression levels of Meg3, Rian and Mirg RNA determined by RT-qPCR in WT (BJ) and Δ*Meg3-C1* mESCs, relative to U6, with WT values put at 1. Bars represent means ± SD from 3 independent experiments. See also Fig. 5d. **e**, Capture Repli-seq on the chromosome-4 *Dock7* control region in BJ (top panel) and Δ*Meg3*-C1 (lower panel). Maternal (red) and paternal (blue) chromosome-specific enrichments are plotted. Gene positions are aligned above.

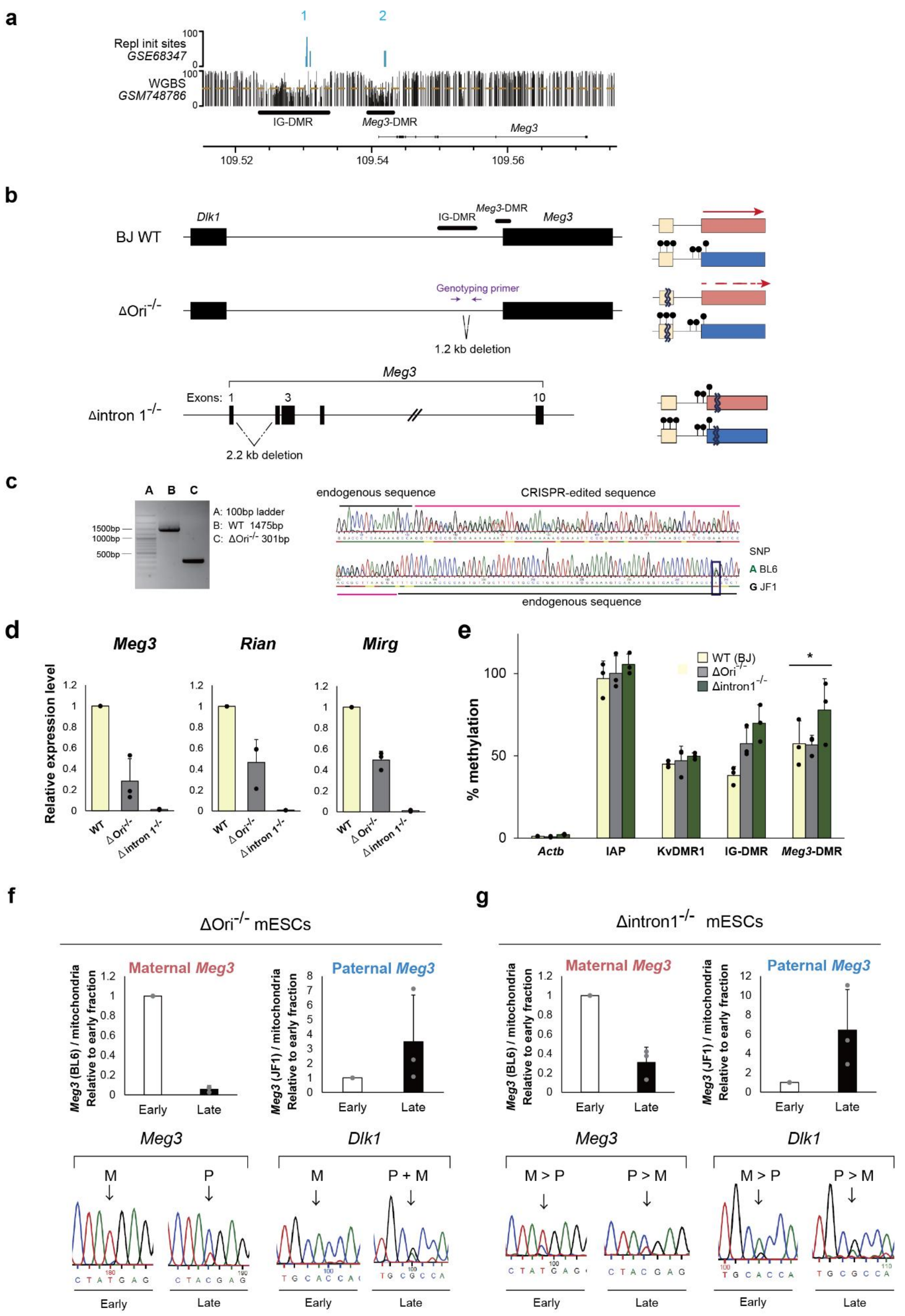
a
1
2
Repl init sites
GSE68347
WGBS
GSM748786
IG-DMR
Meg3-DMR
Meg3
109.52
109.54
109.56
b
BJ WT
Dlk1
IG-DMR
Meg3-DMR
Meg3
ΔOri-/-
Genotyping primer
1.2 kb deletion
Δintron 1-/-
Exons: 1
3
10
2.2 kb deletion
c
A B C
1500bp
1000bp
500bp
A: 100bp ladder
B: WT 1475bp
C: ΔOri-/- 301bp
endogenous sequence
CRISPR-edited sequence
SNP
A BL6
G JF1
endogenous sequence
d
Meg3
Rian
Mirg
Relative expression level
WT
ΔOri-/-
Δintron 1-/-
e
% methylation
WT (BJ)
ΔOri-/-
Δintron1-/-
Actb
IAP
KvDMR1
IG-DMR
Meg3-DMR
f
ΔOri-/- mESCs
Maternal Meg3
Paternal Meg3
Meg3 (BL6) / mitochondria Relative to early fraction
Meg3 (JF1) / mitochondria Relative to early fraction
Early
Late
Meg3
Dlk1
M
P
M
P + M
Early
Late
g
Δintron1-/- mESCs
Maternal Meg3
Paternal Meg3
Meg3
Dlk1
M > P
P > M
M > P
P > M
Early
Late

Supplementary Fig. 6. / RT analysis at *Dlk1-Dio3* in additional CRISPR-Cas9-generated mESC deletion lines.

a, The *Meg3* region, with replication initiation sites mapped within the IG-DMR and *Meg3*-DMR [data from (2)]. The indicated extent of the DMRs is based on mouse ESC WGBS data extracted from (51). **b,** Schematic presentation of the *Dlk1-Dio3* locus, in BJ, Δintron-1$^{-/-}$ and ΔOri$^{-/-}$ mESCs, with the position of their (biallelic) deletions indicated. To the right, summary of the lines' allelic expression and methylation statuses of the parental chromosomes. **c,** PCR-based genotyping of ΔOri$^{-/-}$ mESCs. Agarose gel: the ΔOri$^{-/-}$ PCR fragment in the right lane shows the (biallelic) deletion of the targeted 1.2-kb region comprising initiation site 2. To the right, Sanger sequencing of this 301-bp PCR fragment (from ΔOri$^{-/-}$ mESCs) confirms presence of both parental chromosomes, with the diagnostic SNP indicated with a box. **d,** Meg3, Rian and Mirg RNA levels assessed by RT-qPCR relative to Gapdh in BJ, Δintron-1$^{-/-}$ and ΔOri$^{-/-}$ mESCs. Bars represent means ± SD from 3 independent experiments. **e,** Methylation-sensitive qPCR analysis in Δintron 1$^{-/-}$, ΔOri$^{-/-}$ and BJ mESCs at the KvDMR1, IG-DMR, and *Meg3*-DMR. Bars represent means ± S.D. from 3 independent experiments. *, $p<0.05$. **f,g,** RT analysis at *Dlk1* and *Meg3* analysed by PCR-based approaches. Top panels, quantification of RT of maternal and paternal *Meg3* in Δintron-1$^{-/-}$ (f) and ΔOri$^{-/-}$ (g) mESCs. Bars represent means ± SD from 3 independent experiments. Bottom panels, Sanger sequencing-based assessment of *Meg3* and *Dlk1* allelism in early and late fractions in Δintron-1$^{-/-}$ and ΔOri$^{-/-}$ mESCs. Arrows indicate the SNPs used to distinguish the maternal (M) and paternal (P) alleles.

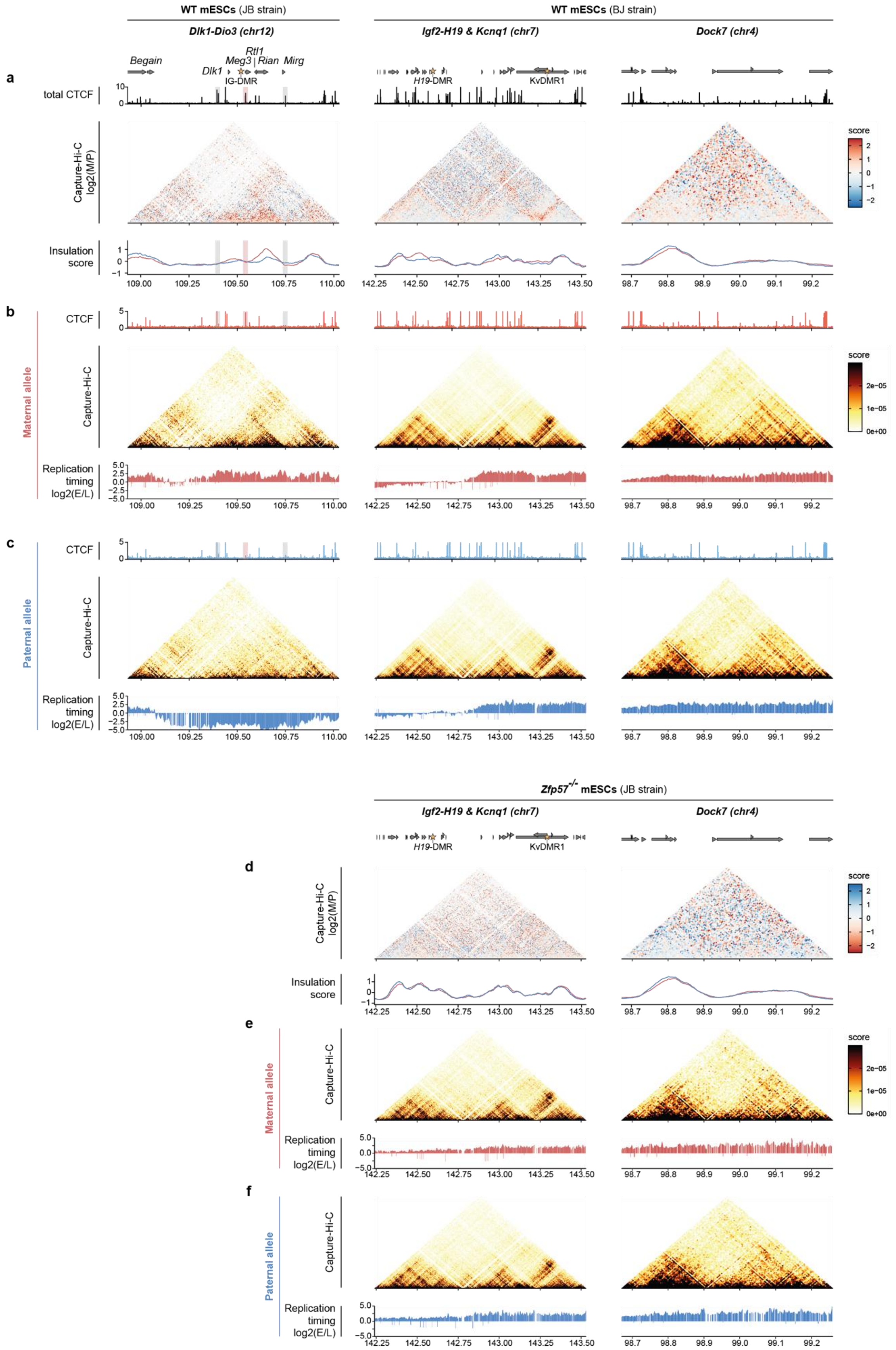

WT mESCs (JB strain)
WT mESCs (BJ strain)
Dlk1-Dio3 (chr12)
Igf2-H19 & Kcnq1 (chr7)
Dock7 (chr4)
a
b
c
total CTCF
Capture-Hi-C log2(M/P)
Insulation score
CTCF
Capture-Hi-C
Replication timing log2(E/L)
Maternal allele
Paternal allele
Begain
Rtl1
Meg3
Rian
Mirg
Dlk1
IG-DMR
H19-DMR
KvDMR1
score
Zfp57-/- mESCs (JB strain)
d
e
f

Supplementary Fig. 7. / Supplementary Capture Hi-C, Capture Repli-seq and CTCF ChIP data on the *Igf2-H19* and *Kcnq1* domains and on the chromosome 4 control locus in hybrid mESCs.

a, Comparison of the 3D-chromatin organization between the maternal and the paternal chromosomes using allele-specific Capture Hi-C in WT (left: JB; middle and right: BJ) mESCs at the *Dlk1-Dio3* locus (left), at the imprinted *Igf2-H19* & *Kcnq1* locus on chromosome 7 (middle) and the control non-imprinted locus *Dock7* on chromosome 4 (right). Bins are 5-kb. Stronger signal on the maternal allele (log2 ratio) is shown in red, while stronger signal on the paternal allele is shown in blue. Capture Hi-C is aligned with CTCF ChIP-seq profiles (on top) and the insulation scores across the maternal (red) and paternal chromosomes (blue). **b,** 3D chromatin architecture of the maternal chromosome determined by Capture Hi-C is aligned with maternal CTCF binding and RT in WT (left: JB; middle and right: BJ) mESCs at the *Dlk1-Dio3* locus (left), the imprinted *Igf2-H19* & *Kcnq1* locus on chromosome 7 (middle) and the control non-imprinted locus *Dock7* on chromosome 4 (right). **c,** 3D chromatin architecture of the paternal chromosome determined by Capture Hi-C is aligned with paternal CTCF binding and RT in WT (left: JB; middle and right: BJ) mESCs at the *Dlk1-Dio3* locus (left), the imprinted *Igf2-H19* & *Kcnq1* loci on chromosome 7 (middle) and the control non-imprinted locus *Dock7* on chromosome 4 (right). Gene positions are aligned above; asterisks indicate the IG-DMR, *H19*-DMR and KvDMR1 imprinting control regions. In (a), the red bars indicate the maternal-specific *Meg3*-DMR CTCF binding whereas grey highlights indicate CTCF peaks upstream *Dlk1* and downstream of *Mirg* that are important for the locus' architecture [56]. **d,e,f,** The same as in panels a, b and, c, but for *Zfp57*$^{-/-}$ mESCs, at the *Igf2-H19* & *Kcnq1* region on chromosome 7 (left) and the control non-imprinted locus *Dock7* on chromosome 4 (right).

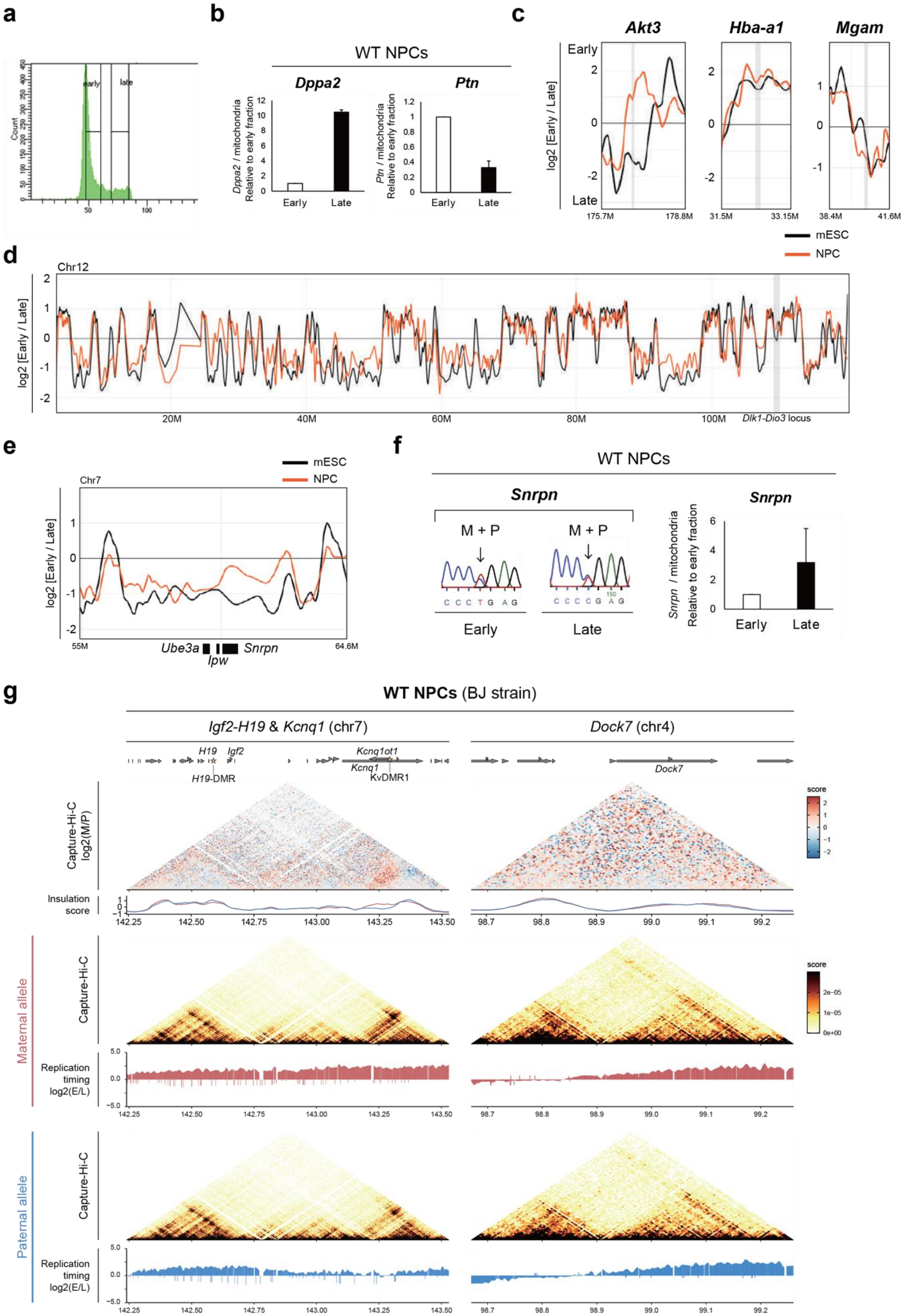

a
early
late
Count
b
WT NPCs
Dppa2
Ptn
Dppa2 / mitochondria Relative to early fraction
Ptn / mitochondria Relative to early fraction
Early
Late
c
Akt3
Hba-a1
Mgam
Early
Late
log2 [Early / Late]
175.7M
178.8M
31.5M
33.15M
38.4M
41.6M
mESC
NPC
d
Chr12
log2 [Early / Late]
20M
40M
60M
80M
100M
Dlk1-Dio3 locus
e
mESC
NPC
Chr7
log2 [Early / Late]
55M
64.6M
Ube3a
Ipw
Snrpn
f
WT NPCs
Snrpn
M + P
Early
Late
Snrpn / mitochondria Relative to early fraction
g
WT NPCs (BJ strain)
Igf2-H19 & Kcnq1 (chr7)
Dock7 (chr4)
H19
Igf2
H19-DMR
Kcnq1ot1
Kcnq1
KvDMR1
Dock7
Capture-Hi-C log2(M/P)
score
Insulation score
Maternal allele
Capture-Hi-C
Replication timing log2(E/L)
Paternal allele

Supplementary Fig. 8. / Supplementary RT and capture-HiC studies on NPCs.

a, Representative FACS profile of NPCs after 9 days of differentiation (of BJ mESCs), with the early and late fractions selected for RT studies. **b,** PCR-based assessment of RT at the *Dppa2* and *Ptn* loci in NPCs. Bars represent means ± S.D. from 3 experiments. **c**, Chromosomal RT profiles at the representative *Akt3* (late), *Hba-a1* (early) and *Mgam* (late) genes obtained by array hybridization in BJ mESCs (black lines) and BJ-derived NPCs (orange lines). Vertical lines indicate the gene positions. **d**, RT along chromosome 12 in NPCs (orange line) compared to mESCs (black line), assessed by array hybridisation. **e,** Comparison of RT profiles in mESCs versus NPCs around *Snrpn* on central chromosome 7. Upon neural differentiation, there is a shift towards earlier replication timing at this domain. **f,** Left, Sanger sequencing-based assessment of RT at *Snrpn* region 1 (see Supplementary Fig. 1f) in BJ-derived NPCs. The arrow indicate a SNP used to distinguish the maternal (M) and paternal (P) alleles. Right, PCR based assessment of RT at *Snrpn* in NPCs. Bars represent means ± S.D. from 3 experiments. **g**, Capture Hi-C in BJ-derived NPCs, aligned with allelic RT (Repli-seq) on the maternal (middle, red) and the paternal chromosome (bottom, blue) at the *Igf2-H19* and *Kcnq1* imprinted domains (left), and at the non-imprinted *Dock7* control locus on chromosome 4 (right). A comparison of the 3D-chromatin organization between the maternal and the paternal chromosome is shown on top, with the insulation score of each allele underneath the comparison heatmaps. Gene positions are aligned above; asterisks indicate the *H19*-DMR and the KvDMR1 imprinting control regions.

Supplementary Table 1—Genome-wide percentile differences in RT between parthenogenetic and androgenetic mESCs and biparental mESCs and NPCs.

	Parthenogenetic mESCs	Androgenetic mESCs	Biparental WT mESCs	Biparental WT NPCs
**Parthenogenetic mESCs**	0	10.3	8.9	-
**Androgenetic mESCs**	10.3	0	10.5	-
**Biparental WT mESCs**	8.9	10.5	0	37
**Biparental WT NPCs**	-	-		0

Supplementary Table 2—Heatmap of RT at imprinted domains in biparental mESCs and NPCs.

Imprinted gene domain	Germline DMR	Methylated allele (M / P)	Centre of the germline DMR (mm10)	Biparental WT mESCs (RT)	Biparental WT NPCs (RT)
Gpr1/Zdbf2	*Liz* promoter DMR	M	chr1: 63,232,617	0.96	1.19
Mcts2/H13	*Mcts2* DMR	M	chr2: 152,687,015	0.85	0.83
Nnat/Blcap	*Nnat* DMR	M	chr2: 157,560 856	0.88	1.15
Gnas	*Nespas-Gnasxl* DMR	M	chr2: 174,298,344	0.67	0.33
Gnas	Exon 1A DMR	M	chr2: 174,327,969	0.67	0.33
Peg10/Sgce	*Peg10* DMR	M	chr6: 4,747,754	0.58	0.53
Klf14/Mest	*Peg1* DMR	M	chr6: 30,737,911	0.47	1.12
Herc3/Nap1L5	Intron 22 *Herc3* DMR	M	chr6: 58,906,879	-0.22	-1.02
Peg3/Zim	*Peg3* DMR	M	chr7: 6,729,971	0.15	0.13
Snrpn	*Snrpn* DMR	M	chr7: 60,005,204	-0.99	-0.58
Inpp5f	*Inpp5f*-V2 DMR	M	chr7: 128,688,276	0.84	0.67
H19-Igf2	*H19* DMR	P	chr7: 142,581,479	0.05	1.04
Kcnq1	KvDMR1	M	chr7: 143,378,397	0.99	0.76
Cdh15	*Cdh15* DMR	M	chr8 : 122,865,203	1.06	0.78
Rasgrf1	*Rasgrf1* DMR	M	chr9: 89,881,159	0.24	-0.27
Plagl1 (Zac1)	*Plagl1 (Zac1)* DMR	M	chr10: 13,090,882	0.66	0.33
Grb10	*Grb10* DMR	M	chr11: 12,025,933	0.78	0.55
U2af1-rs1-Murr1	*U2af1-rs1* DMR	M	chr11: 22,972,516	0.83	0.5
Dlk1-Dio3	IG-DMR	P	chr12: 109,534,965	0.08	0.42
Peg13/KcnK9	*Peg13* DMR	M	chr15: 72,809,763	0.45	0.1
Slc38a4	*Scl38a4* DMR	M	chr15: 97,054,755	0.62	-0.12
Igf2r	Airn DMR	M	chr17: 12,755,662	0.65	0.85
Impact	*Impact* DMR	M	chr18: 12,973,037	0.77	-0.015

The colour range, from green (early replication) to red (late replication), indicates RT and was determined by the earliest and latest values obtained from the smooth curves generated by START-R (3). 0 corresponds to the middle of the S phase. Each value comes from the smooth curves generated by START-R with the loess method (Fits a polynomial surface determined by one or more numerical predictors, using local fitting) and the span parameter = 800 000, which controls the degree of smoothing.

Supplementary Table 3—Guide RNAs and PCR oligonucleotides used in this study.

Guide RNAs

Targeted region	sgRNA1	sgRNA2	Reference
IG-DMR Rep origin	ATATCTCTCACCTGACTAA	TCGTATTGAGACCACAACC	This study.
Meg3 promoter	GGGTGTTGGTCATGGCGGCC	TCGTCTTCTGTGCTAGGGGC	(4)
Meg3 exon 2-10	TGTAATTAATACCTATACCT	TGGGTGTGCTACTAATACGA	This study.

Genotyping primers

IG-DMR Ori-deletion<br>(ΔOri$^{-/-}$ mESCs)	Fw<br>Rv-WT<br>Rv-KO	AAACAATTCCCACTTGATTTACGG<br>ACTCATGGTCCTGAGCGTTG<br>GCTGGCCATATGGTGGATCA	1475 bp (WT) This study.<br>302 bp (KO)
Meg3 promoter deletion (*Zfp57*$^{-/-}$;*Meg3*-pro$^{-/-}$ mESCs)	Fw<br>Rv	CCGGTACCCCACCTTTATCC<br>AGCCGAAGCCAGATAGTGATG	529 bp (WT) This study.<br>270 bp (KO)
Meg3 exon 2-10<br>(ΔMeg3-C1 mESCs)	Fw-WT<br>Rv-WT<br>Fw-KO<br>Rv-KO	ACAACCCCCTCCCATATCCT<br>ACTAGGGCACTGGTTCAAGG<br>CATGCGCACACAGCTAACAC<br>GACCACCCATCTGGGTCAAG	503 bp (WT) This study.<br>1254 bp (KO)

PCR primers for genomic DNA replication studies

Gene name	Primers		Size / Reference
Mitochondrial gene	Fw<br>Rv	GACATCTGGTTCTTACTTCA<br>GTTTTTGGGGTTTGGCATTA	345 bp. (5)
Dppa2	Fw<br>Rv	CCACAGGAAGACAGGAAGCAGT<br>AGCCAGACAGGAGCCCTAGAGT	199 bp. (6)
Ptn	Fw<br>Rv	CTGGAATGAGTTACTGACGGGG<br>CTGGCCCCACTGTGTAATAAGC	230 bp. (7)
Meg3 allele-specific	Fw (BL6)<br>Fw (JF1)	TTGCTTGGGTATAATGGGGAATAC<br>TTGCTTGGGTATAATGGGGAATTT	188 bp. This study.<br>SNP: A (B6), T (JF1)

qPCR	Rv-common	AGGCTTGGTCCTCAGTCAGA	
Meg3<br>Sanger sequen.	Fw<br>Rv	TTGCCAGCTTGATTGCTCTC<br>CGAGCATCCATCTCCTGCAA	287 bp. This study.<br>SNP: T (B6), C (JF1).
Dlk1	Fw<br>Rv	CTCTGGGGAAGCCTGCGATG<br>CCCCTCTTGAACGCCACTTC	232 bp. This study.<br>SNP: A (B6), T (JF1).
Snrpn,<br>Region 1	Fw<br>Rv	GGACGCGCAATCAGTCTCTA<br>AGCTATCCAACCCTTCGCTT	272bp. This study.<br>SNP: C (B6), T (JF1).
Snrpn,<br>Region 2	Fw<br>Rv	AGGTTGTGACTGGGATCCTG<br>GCGGCAACAGAACTTCT	229 bp. (8)<br>SNP: A (B6), G (JF1).
Ipw<br>Region 3	Fw<br>Rv	TCCCTTAGTGAGAAACCAAAGG<br>ATGGGATTTAGGGTTTTCTGGCA	298 bp. This study.<br>SNP: A (B6), G (JF1)
H19	Fw<br>Rv	CACGCTATACAACCCCACCA<br>TGCACTTACGGAATGGTCCC	327 bp. This study.<br>SNP: A (B6), T (JF1)
Peg3	Fw<br>Rv	TGGTGCAGACATTGAAGACC<br>GTACTTGTCGTCTGCCTGCT	244 bp. This study.<br>SNP: T (B6), C (JF1)
Rasgrf1	Fw<br>Rv	TCTCTCTGGGGGCTTTGCTA<br>CTGACCTTTGCCACTGGGAT	371 bp. This study.<br>SNP: A (B6), G (JF1)
Slc38a4	Fw<br>Rv	ATGCCCCAAATGTTCCGACT<br>TGCCGTTGAGGTACCATTCC	273 bp. This study.<br>SNP: T, C (B6); C, A (JF1

Primers for PCR-based DNA methylation studies

Gene/locus	DNA methylation primers	Size / Reference
IG-DMR	Fw GCCTGCTGCTCATTGTTAGC<br>Rv TTCATTCATGGGGCCAGGTC	210 bp. This study.
Meg3 promoter	Fw GGGTAGGCAGAGCAGCCGGA<br>Rv AGGGGTACCCAGCAACCCGG	278 bp. (9)
Meg3 promoter, for Sanger sequencing	Fw CTCTTCCAGCTTCATGTCCTCC<br>Rv AGGGGTACCCAGCAACCCGG	488 bp. This study.<br>SNP: G (B6) A (JF1)
KvDMR1	Fw CTCAGTTCCACGATACCCTTCC<br>Rv CTTACAGAAGCAGGGGTGGTCT	300 bp. (5)
IAP	Fw CAAATTAAAGAGCTTGCCGAGT	136 bp. (7)

	Rv	TAGGGAGAGCGGCTTTTACA	
Col1a2	Fw	AAAGAGAAGGATTGGTCAGAGCAGT	60 bp. (5)
	Rv	GCCAAGGGAGGAGACTTAGTTG	
Snrpn-DMR	Fw	TGACCTTCCTCGCTCCATTG	255 bp. This study.
	Rv	TCCGCAGTAGGAATGCTC	
H19-DMR	Fw	CCAGCAGCTCCCCTTTATC	178 bp. (9)
	Rv	GTTGAAGGACTGAGGGGCTA	

PCR primers for RNA expression studies

**Gene**	**RNA expression primers**		**Size / Reference**
Meg3	Fw	CGAGGACTTCACGCACAACAC	72 bp. (10)
	Rv	CCACGGAGGATTCCAGATGATG	
Rian	Fw	CAATGGGTGGATCGTACCTC	190 bp. (9)
	Rv	GTGCTGCCTCAGTCTTTGTG	
Mirg	Fw	TCGGCAGTACATACCAGGTG	196 bp. (9)
	Rv	ACTGATGGCTTCAGGTCAGG	
Gapdh	Fw	GGAGCGAGACCCCACTAACA	51 bp. (10)
	Rv	ACATACTCAGCACCGGCCTC	

Supplementary References

1. Nakatani, T. *et al.* Emergence of replication timing during early mammalian development. *Nature* **625,** 401-409 (2024).

2. Cayrou, C. *et al.* The chromatin environment shapes DNA replication origin organization and defines origin classes. *Genome Res* **25**, 1873-85 (2015).

3. Hiratani, I. *et al.* Genome-wide dynamics of replication timing revealed by in vitro models of mouse embryogenesis. *Genome Res* **20**, 155-69 (2010).

4. Sanli, I. *et al.* Meg3 Non-coding RNA Expression Controls Imprinting by Preventing Transcriptional Upregulation in cis. *Cell Rep* **23**, 337-348 (2018).

5. Varrault, A. *et al.* Mouse parthenogenetic embryonic stem cells with biparental-like expression of imprinted genes generate cortical-like neurons that integrate into the injured adult cerebral cortex. *Stem Cells* **36**, 192-205 (2018).

6. Sima, J. *et al.* Identifying cis elements for spatiotemporal control of mammalian DNA replication. *Cell* **176**, 816-830 e18 (2019).

7. Therizols, P. *et al.* Chromatin decondensation is sufficient to alter nuclear organization in embryonic stem cells. *Science* **346**, 1238-42 (2014).

8. Gregory, R.I. *et al.* DNA methylation is linked to deacetylation of histone H3, but not H4, on the imprinted genes Snrpn and U2af1-rs1. *Mol Cell Biol* **21**, 5426-36 (2001).

9. Farhadova, S. *et al.* The long non-coding RNA Meg3 mediates imprinted gene expression during stem cell differentiation. *Nucleic Acids Res* **52**, 6183-6200 (2024).

10. Kota, S.K. *et al.* ICR Noncoding RNA Expression Controls Imprinting and DNA Replication at the *Dlk1-Dio3* Domain. *Dev. Cell* **31**, 19-33 (2014).
