# Supplementary material for: DNA methylation and lncRNA control asynchronous DNA replication at specific imprinted gene domains
Source: Nat Commun. 2026 Jan 21;17:1844. doi: 10.1038/s41467-026-68558-2 (PMC12920997; doi:10.1038/s41467-026-68558-2)
Supplement: Supplementary file 2 — Reporting Summary [file 41467_2026_68558_MOESM2_ESM.pdf]

nature portfolio

Corresponding author(s): Robert Feil, Jean-Charles Cadoret and Benoit Moindrot

Last updated by author(s): Dec 12, 2025

Reporting Summary

Nature Portfolio wishes to improve the reproducibility of the work that we publish. This form provides structure for consistency and transparency in reporting. For further information on Nature Portfolio policies, see our Editorial Policies and the Editorial Policy Checklist.

Statistics

For all statistical analyses, confirm that the following items are present in the figure legend, table legend, main text, or Methods section.

n/a	Confirmed	
☐	☒	The exact sample size (n) for each experimental group/condition, given as a discrete number and unit of measurement
☐	☒	A statement on whether measurements were taken from distinct samples or whether the same sample was measured repeatedly
☐	☒	The statistical test(s) used AND whether they are one- or two-sided<br>*Only common tests should be described solely by name; describe more complex techniques in the Methods section.*
☒	☐	A description of all covariates tested
☒	☐	A description of any assumptions or corrections, such as tests of normality and adjustment for multiple comparisons
☐	☒	A full description of the statistical parameters including central tendency (e.g. means) or other basic estimates (e.g. regression coefficient) AND variation (e.g. standard deviation) or associated estimates of uncertainty (e.g. confidence intervals)
☐	☒	For null hypothesis testing, the test statistic (e.g. F, t, r) with confidence intervals, effect sizes, degrees of freedom and P value noted<br>*Give P values as exact values whenever suitable.*
☒	☐	For Bayesian analysis, information on the choice of priors and Markov chain Monte Carlo settings
☒	☐	For hierarchical and complex designs, identification of the appropriate level for tests and full reporting of outcomes
☒	☐	Estimates of effect sizes (e.g. Cohen's d, Pearson's r), indicating how they were calculated

Our web collection on statistics for biologists contains articles on many of the points above.

Software and code

Policy information about availability of computer code

Data collection	Softwares used and softwares versions are described in the methods section of the manuscript
Data analysis	RT-qPCR and Repli-qPCR data were analysed with Excel<br>Methylation analysis data were analysed with Excel<br>Sanger Sequencing data were plotted by the Sanger sequencing company (pdf)<br>Repli-Array, Repli-seq, RNA-seq, ChIP-seq and Hi-C data were analysed and displayed as described in the method section (including software version numbers)

For manuscripts utilizing custom algorithms or software that are central to the research but not yet described in published literature, software must be made available to editors and reviewers. We strongly encourage code deposition in a community repository (e.g. GitHub). See the Nature Portfolio guidelines for submitting code & software for further information.

Data

Policy information about availability of data

All manuscripts must include a data availability statement. This statement should provide the following information, where applicable:

- Accession codes, unique identifiers, or web links for publicly available datasets
- A description of any restrictions on data availability
- For clinical datasets or third party data, please ensure that the statement adheres to our policy

All raw and processed data asscociated with this manuscript are available on GEO under the accession numbers GSE287936, GSE289027, GSE306486, GSE289022, GSE207166

Research involving human participants, their data, or biological material

Policy information about studies with human participants or human data. See also policy information about sex, gender (identity/presentation), and sexual orientation and race, ethnicity and racism.

Reporting on sex and gender	There were no human participants in this study
Reporting on race, ethnicity, or other socially relevant groupings	There were no human participants in this study
Population characteristics	There were no human participants in this study
Recruitment	There were no human participants in this study
Ethics oversight	There were no human participants in this study

Note that full information on the approval of the study protocol must also be provided in the manuscript.

Field-specific reporting

Please select the one below that is the best fit for your research. If you are not sure, read the appropriate sections before making your selection.

☒ Life sciences ☐ Behavioural & social sciences ☐ Ecological, evolutionary & environmental sciences

For a reference copy of the document with all sections, see nature.com/documents/nr-reporting-summary-flat.pdf

Life sciences study design

All studies must disclose on these points even when the disclosure is negative.

Sample size	Genomics experiments were performed in one/two biological replicates depending on the experiments (see Methods section for details).<br>Repli-qPCR experiments were performed in 3-4 biological replicates<br>Methylation analyses were performed in 4-6 biological replicates<br>RT-qPCR expression analyses were performed in 3 biological replicates
Data exclusions	No samples were excluded
Replication	Genomics experiments were performed in one/two biological replicates depending on the experiments (see Methods section for details).<br>Repli-qPCR experiments were performed in 3-4 biological replicates<br>Methylation analyses were performed in 3-6 biological replicates<br>RT-qPCR expression analyses were performed in 3 biological replicates
Randomization	Not applicable
Blinding	Not applicable

Reporting for specific materials, systems and methods

We require information from authors about some types of materials, experimental systems and methods used in many studies. Here, indicate whether each material, system or method listed is relevant to your study. If you are not sure if a list item applies to your research, read the appropriate section before selecting a response.

Materials & experimental systems

n/a	Involved in the study	
☐	☒	Antibodies
☐	☒	Eukaryotic cell lines
☒	☐	Palaeontology and archaeology
☒	☐	Animals and other organisms
☒	☐	Clinical data
☒	☐	Dual use research of concern
☒	☐	Plants

Methods

n/a	Involved in the study	
☒	☐	ChIP-seq
☐	☒	Flow cytometry
☒	☐	MRI-based neuroimaging

Antibodies

Antibodies used

BrdU (BD Biosciences, #347580, lot 3016583)
Nestin (Abcam, #ab81755, 1:500, lot GR154015-3)
Tubulin-B3 (Biolegend, #801201, 1:500, lot B353040)

For Repli-array, Repli-qPCR and Capture Repli-seq, 10 µg of BrdU antibody was used.
For immunostaining, 1: 500 dilution of Nestin and Tubulin-B3 antibody was used.

Validation

BrdU antibody was validated using Immunofluorescence and FACS by BD Biosciences company.
The Nestin antibody (Abcam, #ab81755) was validated for Western Blotting experiment by the supplier.
The Tubulin-B3 (Biolegend, #801201) was validated by Western blotting by the supplier.
No further validations were performed.

Eukaryotic cell lines

Policy information about cell lines and Sex and Gender in Research

Cell line source(s)

All cell lines are mouse embryonic stem cells:
Androgenetic mESCs are described in Khosla et al., 2001
Parthenogenetic mESCs are described in Allen et al.,1994
BJ and JB cells are described in Koide et al., 1998
Sh-1 mESCs are described in Kota et al., 2014
Δintron-1-/- mESCs were generated in Sanli et al., 2018
Zfp57 -/- mESCs were generated in Riso et al., 2016

Authentication

Cell lines were not authenticated, beyond their capacity for proliferation and morphology in the dedicated embryonic stem cell medium.

Mycoplasma contamination

All cell lines were regularly validated to be negative for mycoplasma contamination.

Commonly misidentified lines
(See ICLAC register)

No commonly misidentified cell lines were used in the study.

Plants

Seed stocks

-

Novel plant genotypes

-

Authentication

-

Flow Cytometry

Plots

Confirm that:

☒ The axis labels state the marker and fluorochrome used (e.g. CD4-FITC).

☒ The axis scales are clearly visible. Include numbers along axes only for bottom left plot of group (a 'group' is an analysis of identical markers).

☒ All plots are contour plots with outliers or pseudocolor plots.

☒ A numerical value for number of cells or percentage (with statistics) is provided.

Methodology

Sample preparation	Cells were stained with 80 µg/mL propidium iodide (Invitrogen, P3566) at room temperature in the presence of 0.4 mg/mL RNaseA (Roche, #10109169001) for 1h
Instrument	AriaII FACS machine (BD Biosciences)
Software	-
Cell population abundance	The cells were sorted into an early and late replicating fractions based on propidium iodide staining.
Gating strategy	An ‘early fraction’ comprised of late-G1 and the first ~40% of S-phase, was compared with a ‘late fraction’ covering the last ~40% of S-phase and early G2

☒ Tick this box to confirm that a figure exemplifying the gating strategy is provided in the Supplementary Information.
