# Supplementary material for: DNA methylation and lncRNA control asynchronous DNA replication at specific imprinted gene domains
Source: Nat Commun. 2026 Jan 21;17:1844. doi: 10.1038/s41467-026-68558-2 (PMC12920997; doi:10.1038/s41467-026-68558-2)
Supplement: Supplementary file 4 — Source data [file 41467_2026_68558_MOESM4_ESM.zip › Source data/Sanger-sequencing data/Fig2c-d/BJ early-Dlk1.pdf]

Sequence: EF70929976

Samples: 16227
Bases: 633
Average spacing: 26.0
Average quality >= 10: 275, 20: 90, 30: 170

Quality:
0 - 9
10 - 19
20 - 29
>= 30

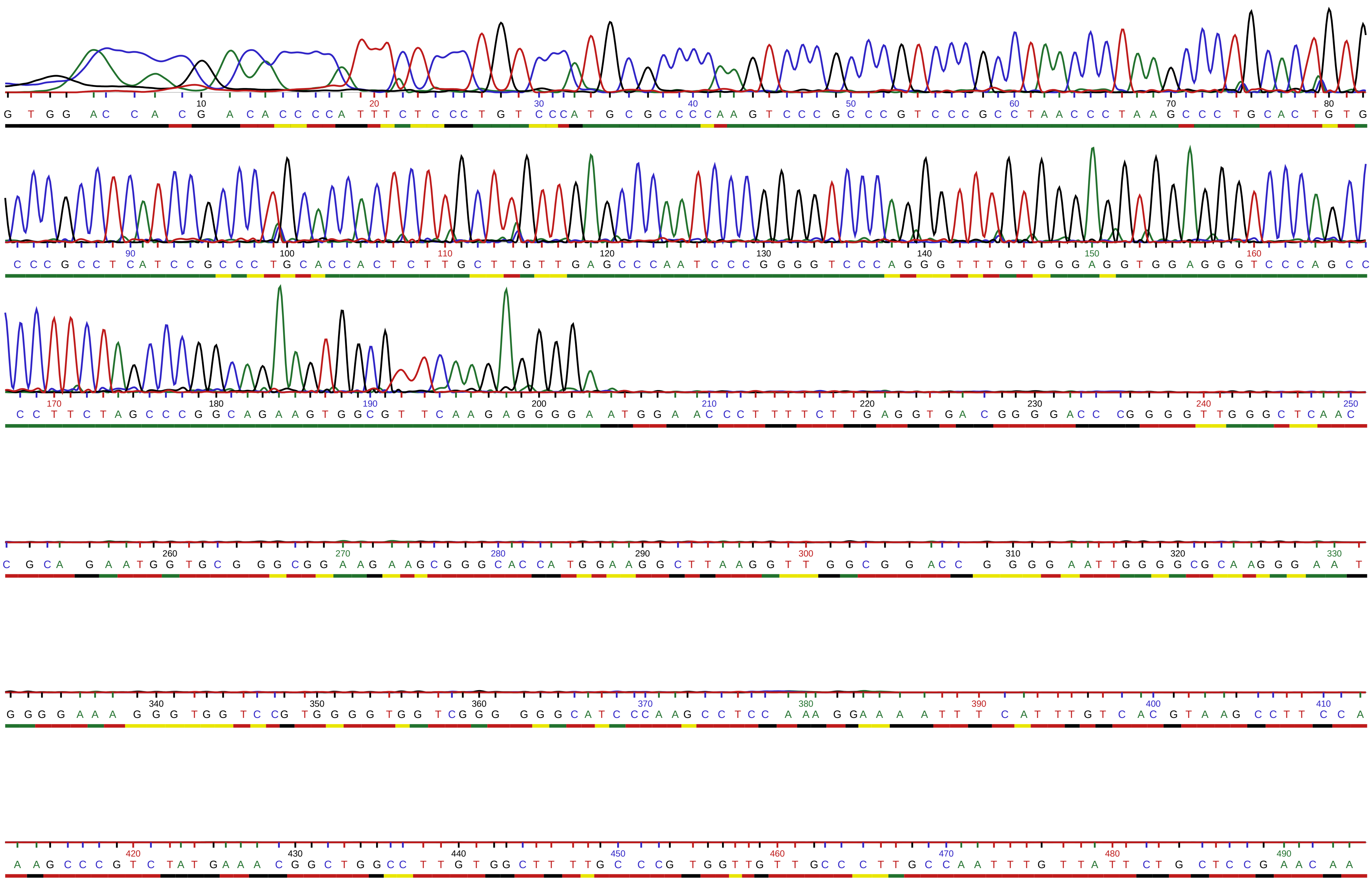

Sequence: EF70929976

Samples:	16227
Bases:	633
Average spacing:	26.0
Average quality >=	10: 275, 20: 90, 30: 170

Quality:

- 0 - 9
- 10 - 19
- 20 - 29
- >= 30

500 510 520 530 540 550 560 570

T G C A T T A T T C T T A C C A G T G C A A G G C G A G C A C A A G G A T C T C C T T C A T T C T G G C T T C T T C C C C T C T T C C A C C C C A C T T T T G C

580 590 600 610 620 630

T C A A T T T A G T T T T T A A T T A T T T T T C G C T A C T A T T G T G C T C T A A T G C T C C A G C C T C T
