# Supplementary material for: DNA methylation and lncRNA control asynchronous DNA replication at specific imprinted gene domains
Source: Nat Commun. 2026 Jan 21;17:1844. doi: 10.1038/s41467-026-68558-2 (PMC12920997; doi:10.1038/s41467-026-68558-2)
Supplement: Supplementary file 4 — Source data [file 41467_2026_68558_MOESM4_ESM.zip › Source data/Sanger-sequencing data/Fig2c-d/JB late-Dlk1.pdf]

Sequence: EF70929846

Samples: 20441
Bases: 1119
Average spacing: 19.0
Average quality >= 10: 363, 20: 326, 30: 236

Quality: 0 - 9, 10 - 19, 20 - 29, >= 30

13.12.2023

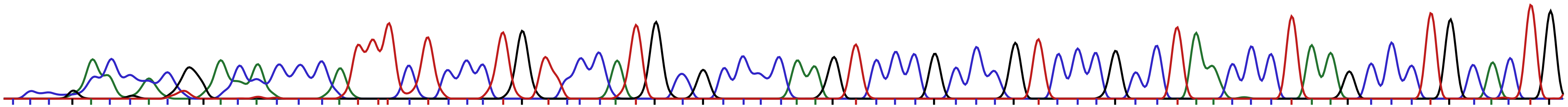

C C C G A C C A C GG A C A C C C A T TT C T C C C T G T CC C A T G C G C C C C A A G T C C C G C C C G T C C C G C C T A A C C C T A A G C C C T G C A C T G

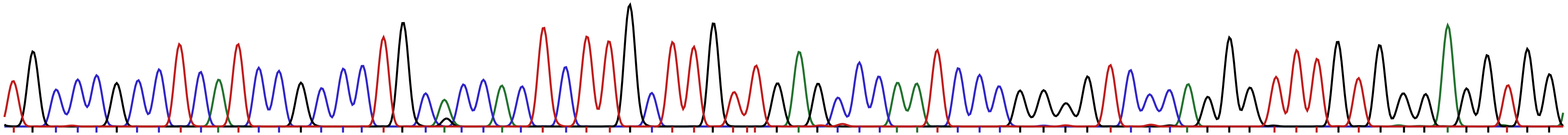

T G C C C G C C T C A T C C G C C C T G C A C C A C T C T T G C T T G T TT G A G C C C A A T C C C G G G G T C C C A G G G T T T G T G G G A G G T G G

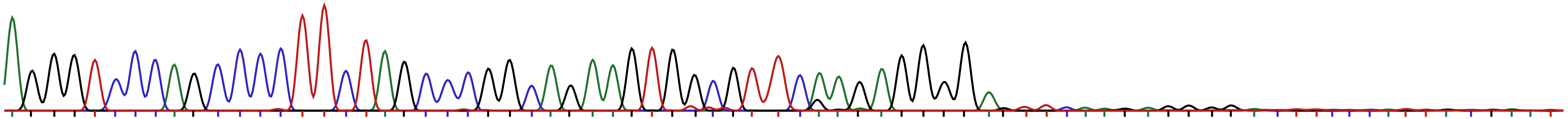

A G G G T C C C A G C C C C T T C T A G C C C G G C A G A A G T G G C G T T C A A G A G G G G A G T T C A A G A G G G G A C T T C C C A T T A C G A A T

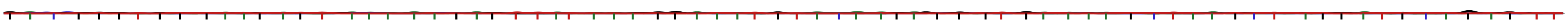

G A C G G G T G G G A A G A G T A A A A A G A G T T A T A A A G G A A A T G T A C A A G A G G G T G A A A A G C T A A G C T A G G A T G C A G A G T T

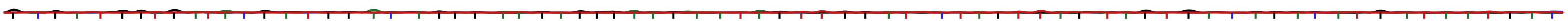

G C G A T G G T G A T A C G A T G G A C A G G G A A G A G G G A A G A A T A G T T G G A T C T A G T T A G T A G T G A C A G A C A T G A T A A T G A C

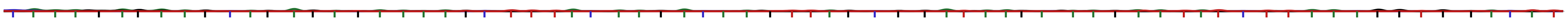

C A A A G A G A A G C A G A G A G A A A A G C T T T A C A A G A C A A G T T A G C G G A T A A G A A A G A A T A T C T T A A A G G T G G A C A T

Sequence: EF70929846

Samples:	20441	Quality: 0 - 9	
Bases:	1119	10 - 19	
Average spacing:	19.0	20 - 29	
Average quality >=	10: 363, 20: 326, 30: 236	>= 30	

460 470 480 490 500 510 520

C G C G A C G T A A A A G A A A A A C A A G C A G A A C G A A G A G A A A A G A A GT A C A G C A G A T A T A A A C T T A A G G AG A G A A A A

530 540 550 560 570 580 590

A T A A A T C T C A G A C A A A A G C C G C C A A G G A C T T A G A T C G T G A C T T G G A C G C A T A A A G T G A A G G T A A A A A A T A G

600 610 620 630 640 650 660 670

G T T G A A A A A G A T T G A T C A A A C T T G A T T G C G T G A A A C T T G A T T A A G G A T T A G AA G A A G AA G A A A C A A A G T C GT C A G G A

680 690 700 710 720 730 740 750

C GT C A A CG C C G T A C A A G G T A C TA T C A A C G G T G A C A A A G G A A C G G A T T C A CT G T G A G A G G AT A A T G A A A T T G G G T T GG A G A A A

760 770 780 790 800 810 820 830 840

A G A A T T G A A G CT A A C A C T T T G A C C T GG A C T G A A G T A T G C A T A A C T G T T A G A A G A A A C A A G A A A A C C AG A G G C T T A A T A A C T G C

850 860 870 880 890 900 910 920

AA A G C T G G A ATC A A G G G A A C T T C C G T GC G A GG A G C T T G G G A A C G G C A T T A A A C A G A G G A A A G G C T A A A G T A A A T C A A C G T T T G A A A

Sequence: EF70929846

Samples:	20441	Quality: 0 - 9	
Bases:	1119	10 - 19	
Average spacing:	19.0	20 - 29	
Average quality >=	10: 363, 20: 326, 30: 236	>= 30	

930 940 950 960 970 980 990 1000

A C A A G G C T T T T A A C G A A G A A A G G C A G A A A A G G C A A A A A T A T T A G T C T G T C T A C T T T G A A A A A A A C T T T A G C C A A A A A A A T C

1010 1020 1030 1040 1050 1060 1070 1080

T T T G C A A C A A A G G T C A A A A A A A A A T T A A C C G G G A A A T A T A G A A A G A A A T T T T G G A A A T T T A G G A A G G T G A A A A A T T T

1090 1100 1110

T G G A A A G C A A A A A A G C A A A A A G G C C C T T T C A A A

Sequence: EF70929846

Samples:	20441	Quality:	0 - 9	
Bases:	1119		10 - 19	
Average spacing:	19.0		20 - 29	
Average quality >=	10: 363, 20: 326, 30: 236		>= 30	

13.12.2023
