# Supplementary material for: DNA methylation and lncRNA control asynchronous DNA replication at specific imprinted gene domains
Source: Nat Commun. 2026 Jan 21;17:1844. doi: 10.1038/s41467-026-68558-2 (PMC12920997; doi:10.1038/s41467-026-68558-2)

Sequence: EF71390844

Samples:	19660	Quality:	0 - 9	
Bases:	465		10 - 19	
Average spacing:	43.0		20 - 29	
Average quality >=	10: 8, 20: 13, 30: 438		>= 30	

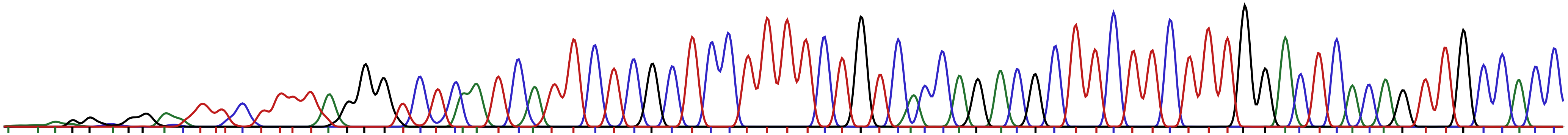

A A A G G A G G A C T T T C T T T T A G G G T C T C A A T C A T T C T C G C T C C T T T T C T G T C A C C A G A C G C T T C T T C T T T G G A C T C A C A G T T G C C A C C

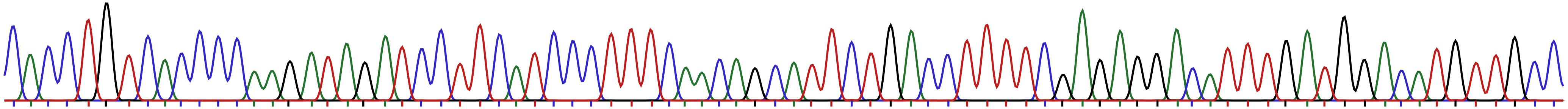

C A C C T G T C A C C C C A A G A T A G A T C C T T C A T C C C T T T C A A C A G C A T T C T G A C C T T T T C G A G A G G A C A T T T G A T G G A C A T G T T G C C

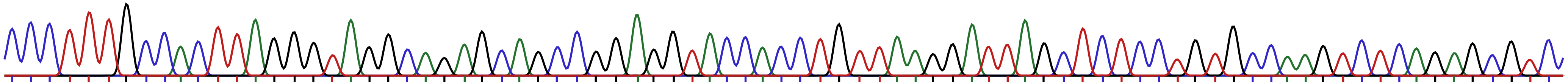

C C C T T T G C C A C T T A G G G T A G G C A G A G C A G C C G G A G G T A C C A C C T G T T A A G G A T T A G C T C T C C T G T G C C A A G T C T C A G A G C G T C

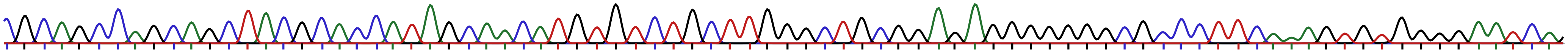

C G C A G C C A G C A G C T A C G C A C C A T A G C A A C A T G T G T C T G C T T G G G C T G C G G A G A G G G G G G G C G C C C T T C A A A G T G T G G G G A A T C A

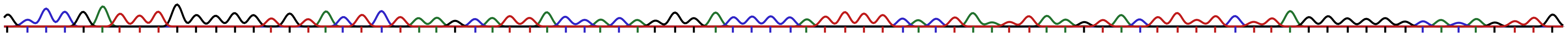

G C C C G A T T T G G G G G T G T A C T C T A A G C A T T A C C A C A G G G A C C C C A T T T T C A C T A A T T A A G T A C T T T T C T T A G G G G G G C A C A G T T G

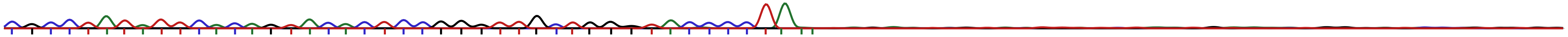

C G C C T A T A T T C A C A G T A C A C T C C G G G T T G C T G G G T A C C C C T A A A

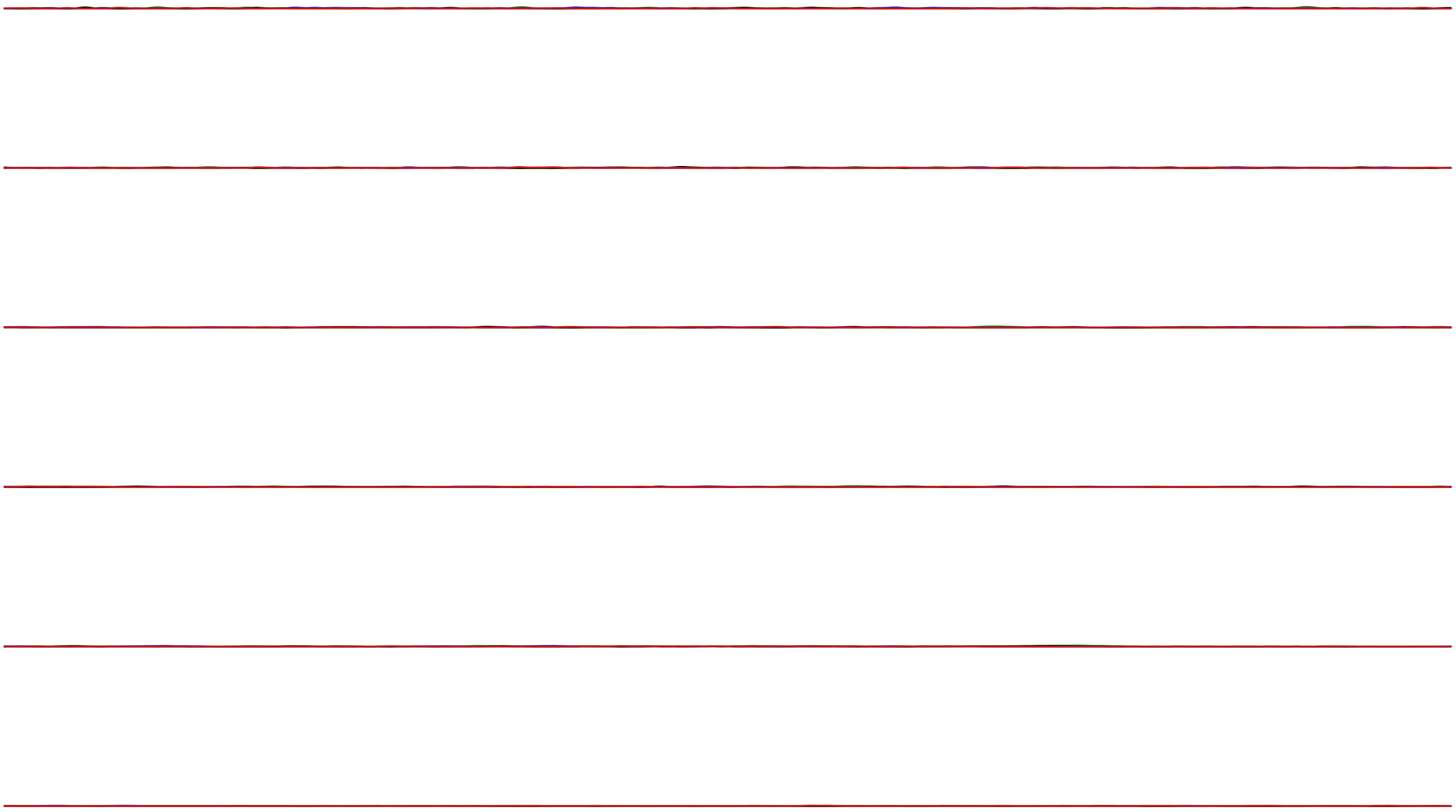

Sequence: EF71390844

Samples:	19660	Quality:	0 - 9	
Bases:	465		10 - 19	
Average spacing:	43.0		20 - 29	
Average quality >=	10: 8, 20: 13, 30: 438		>= 30	

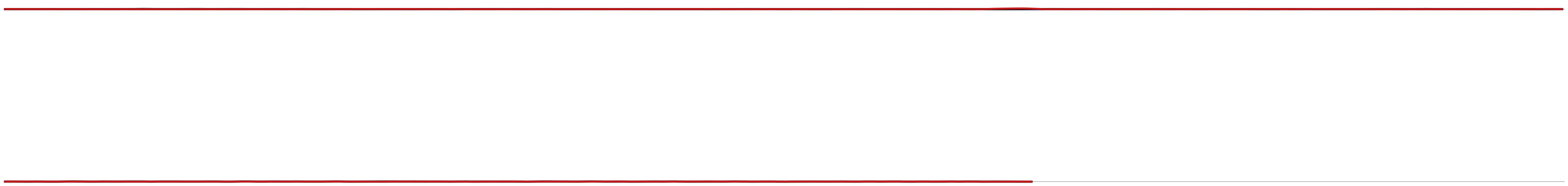

Supplement: Supplementary file 4 — Source data [file 41467_2026_68558_MOESM4_ESM.zip › Source data/Sanger-sequencing data/Fig4d/Meg3DMR-WT-Hpa2+.pdf]
