# Supplementary material for: DNA methylation and lncRNA control asynchronous DNA replication at specific imprinted gene domains
Source: Nat Commun. 2026 Jan 21;17:1844. doi: 10.1038/s41467-026-68558-2 (PMC12920997; doi:10.1038/s41467-026-68558-2)
Supplement: Supplementary file 4 — Source data [file 41467_2026_68558_MOESM4_ESM.zip › Source data/Sanger-sequencing data/Suppl.Fig1f/BJ late SnrpnR2.pdf]

Sequence: EF72763617

Samples: 18333
Bases: 1165
Average spacing: 16.0
Average quality >= 10: 445, 20: 217, 30: 170

Quality: 0 - 9
10 - 19
20 - 29
>= 30

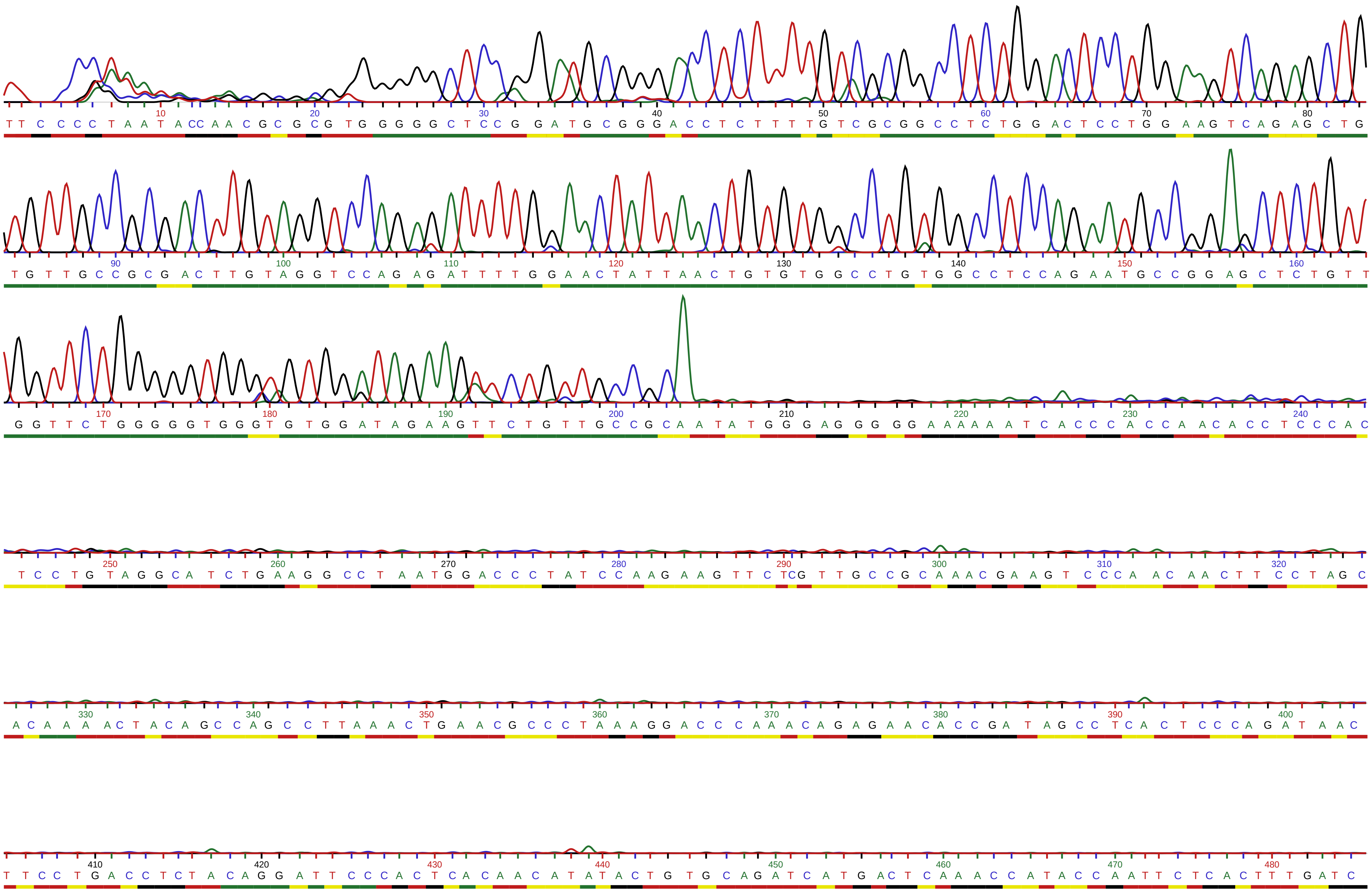

Sequence: EF72763617

Samples: 18333
Bases: 1165
Average spacing: 16.0
Average quality >= 10: 445, 20: 217, 30: 170

Quality: 0 - 9 / 10 - 19 / 20 - 29 / >= 30

G A T G A G T C C A T C C C C A G C C A C T A C A C T A T C G T T G A C T T C G C T T G A A T A A C T C C A G C C C A T G A C T G A A T G A T C T A G C T A T A C C G T C

T G T C C G G C C G A G C C C T A A A C C T C T T A T G G C G C T G C C T G T G C G G T A C C G C A T C G A C A C G G C G A T T C T G A T T C C G A T T G A C C A T G A A G T G A C A G

T C C C A C G A C C T G T T A G G G T C A T G A C A C G T G T T G A C C C A G A C C C T A C T C T A A C C G G T C T C G G C A G A G T G C A G C C G C G T C T G C G C T T C C T C G G T A G

C T T T C A C T G C T T G A A C C T C G G T C G T A C G C T G G C T A T G T G C G A C T A C T C A G C A A C A G T C G C T A C T C G C C C T A T A G C A C G T G T C C T T G C T A T C A T A C T A G C G A G A G C A T G

T C G A A A C T A T A C C A G C A T G C A T C G A T A C A C G G A T A G A A T T G T A T G C T T A C T C C A A T G G C A T A G T A C G G T T A C C A T A G G T A A C A C G A G A G G A A T G C A T C G C A A T C A G T A T G C T A

T A C A T T G C C T A G G G G T A A A C A G C A T C G A A T C T C C G G G C A C T G G A A C A G A T C C T T C A C C A T C A C T T G G G G C A C C C T T C C T T C T T C T G C G C T A G G A C C C T T C T G T T G

Sequence: EF72763617

Samples: 18333
Bases: 1165
Average spacing: 16.0
Average quality >= 10: 445, 20: 217, 30: 170

Quality: 0 - 9 / 10 - 19 / 20 - 29 / >= 30

1090 1100 1110 1120 1130 1140 1150 1160

G C A C C C T C C T A T C C T C A C G G G A A T G C A T A C C T T C G C G C C T T A G A G C C C C C T G T C C A G G A T T G C G C T C T G T G C A T T G G G C T T G C
