# Supplementary material for: DNA methylation and lncRNA control asynchronous DNA replication at specific imprinted gene domains
Source: Nat Commun. 2026 Jan 21;17:1844. doi: 10.1038/s41467-026-68558-2 (PMC12920997; doi:10.1038/s41467-026-68558-2)
Supplement: Supplementary file 4 — Source data [file 41467_2026_68558_MOESM4_ESM.zip › Source data/Sanger-sequencing data/Suppl.Fig2g/Early-G2-Meg3.pdf]

Sequence: EF71385741

Samples:	18260	Quality:	0 - 9		
Bases:	279		10 - 19		12.01.2024
Average spacing:	66.0		20 - 29		
Average quality >=	10: 11, 20: 27, 30: 234		>= 30		

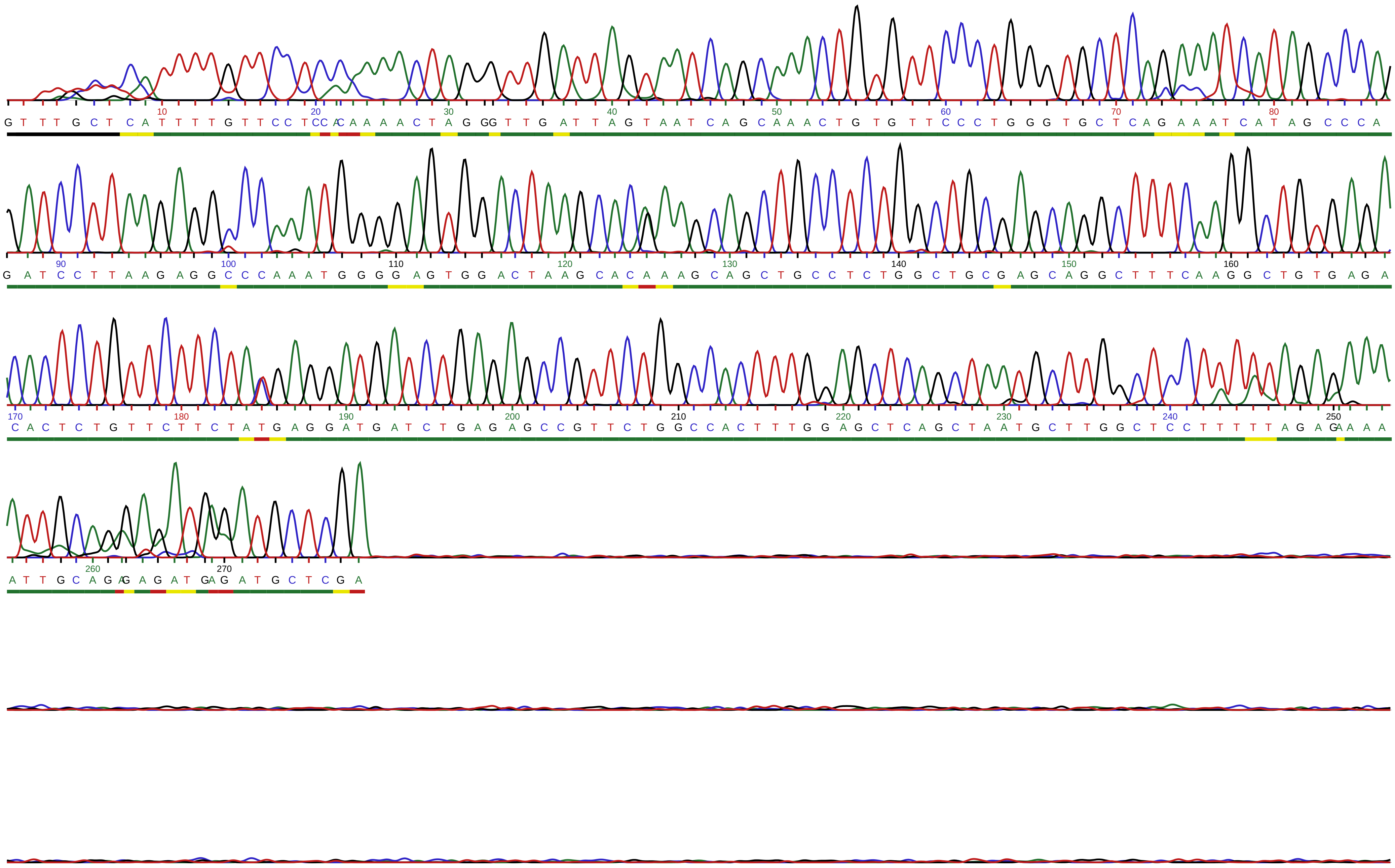

Sequence: EF71385741

Samples:	18260	Quality:	0 - 9	
Bases:	279		10 - 19	
Average spacing:	66.0		20 - 29	
Average quality >=	10: 11, 20: 27, 30: 234		>= 30	

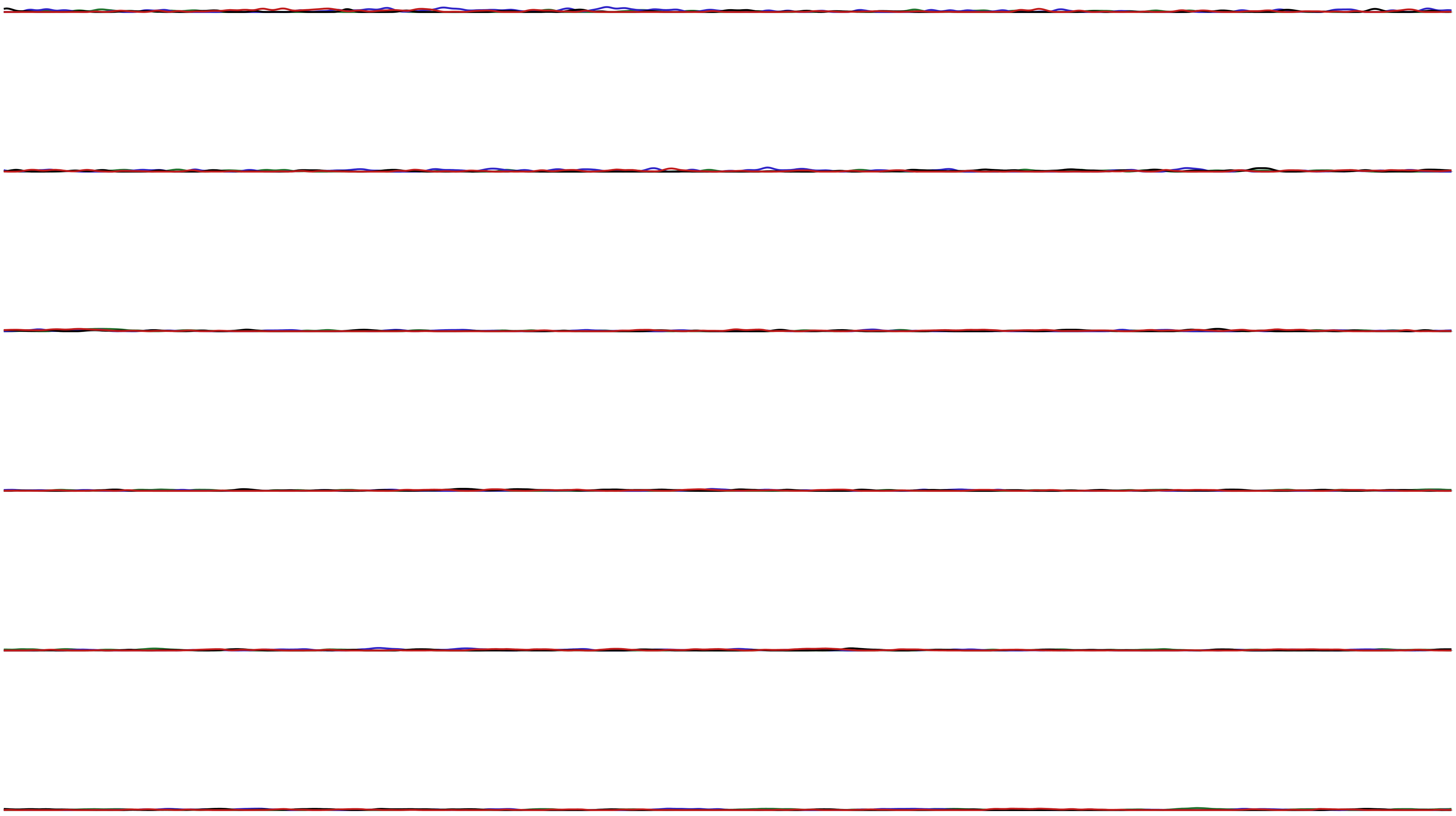

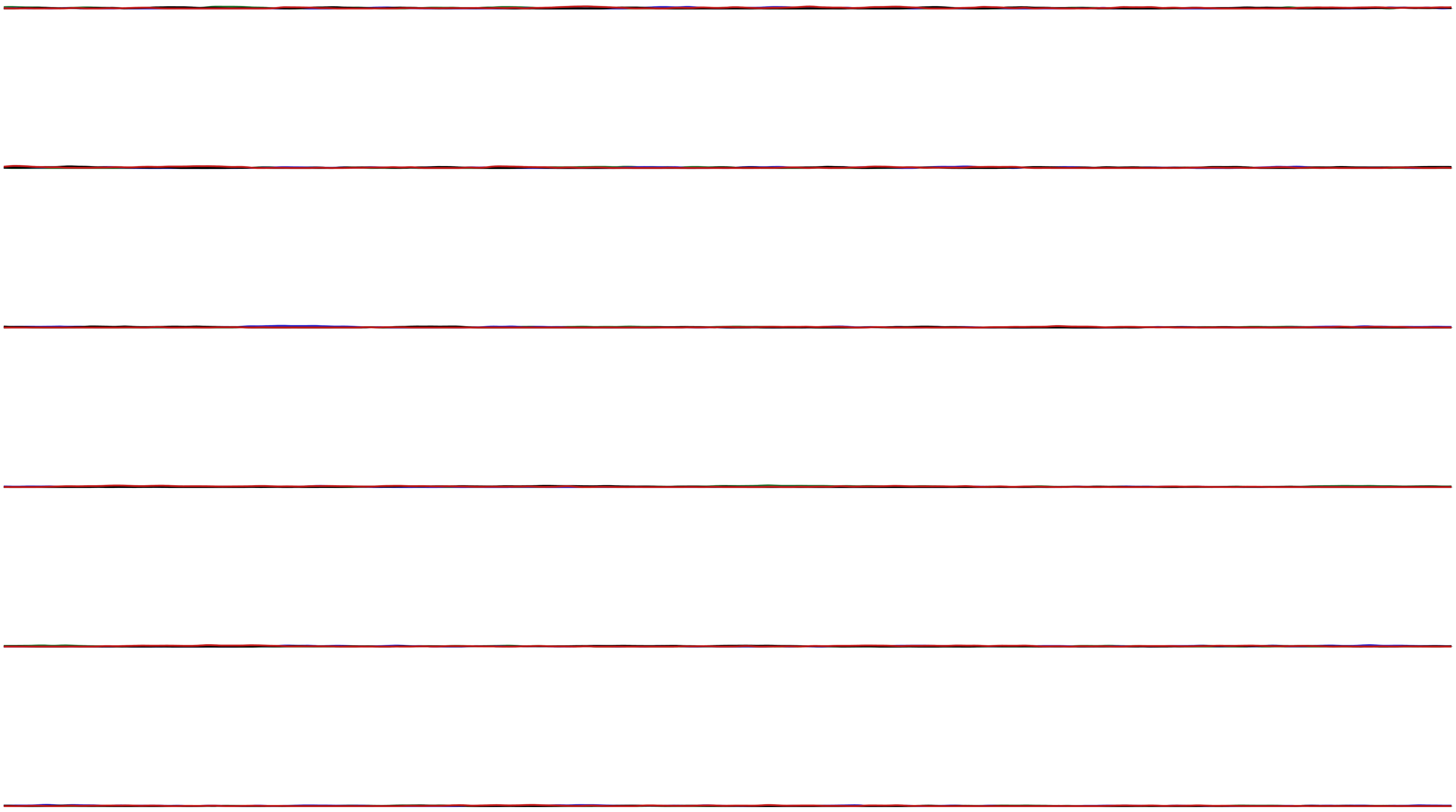

Sequence: EF71385741

Samples:	18260	Quality:	0 - 9	
Bases:	279		10 - 19	
Average spacing:	66.0		20 - 29	
Average quality >=	10: 11, 20: 27, 30: 234		>= 30	
