# Supplementary material for: DNA methylation and lncRNA control asynchronous DNA replication at specific imprinted gene domains
Source: Nat Commun. 2026 Jan 21;17:1844. doi: 10.1038/s41467-026-68558-2 (PMC12920997; doi:10.1038/s41467-026-68558-2)
Supplement: Supplementary file 4 — Source data [file 41467_2026_68558_MOESM4_ESM.zip › Source data/Sanger-sequencing data/Suppl.Fig6c/Ori-sequence.pdf]

Sequence: EF70467675

Samples: 16303
Bases: 623
Average spacing: 27.0
Average quality >= 10: 315, 20: 46, 30: 157

Quality: 0 - 9
10 - 19
20 - 29
>= 30

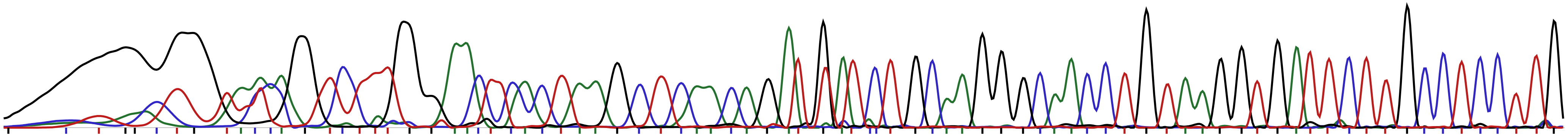

G C T GG C T G T A C C A G T C T T T G G A A C T C A C T A A G C T C A A C A G A T G A T CC T G C A A G G G C A A C C T G T A A G G T G A T T C T T G C C T C C T T G

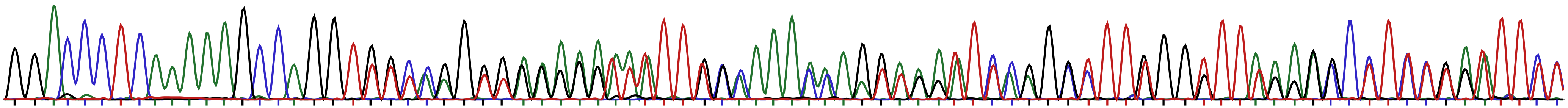

G G A C C C T C A A A A A G C C A G G T G G C C G G G G A A A A A A T T T G C A A A A A A A G G A A A T T C C G G G T T T G G G T T T A A A G C C T T C C G A A T T C C

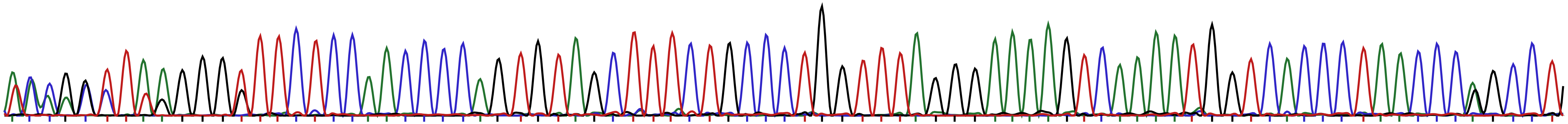

A C C G C T T A A G G G T T T C T C C A A C C C C A G T G T A G C T T T C T G C C C T G G T T T A G G G A A A A G T C A A A A T G G T C A C C C T A A C C C A G C C T

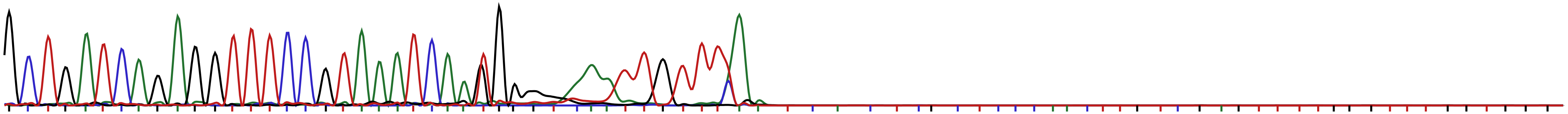

G C T G A T C A G A G G T T T C C G T A A A T C A A T G G G T C A A T T G T T T A A T C A C T C G C T C C C A A C T T G T C G A G T T T T G G G T T T G G T G G G

C A T C C G C T A A C T A T T G A T C T G C T T A A T C G G T T G A C C A C A C C C T G C A G A T A C A C C A T T G G C T G T C A T G G C T C T A A A A T C A C A

A C A A G G C C C G G C C T G G A A G A G T C G A T C G C C G C T A A A A A G A T C A G T A A T A A A G A A A T G G A G A A A G G G C G G T C T T G C A A C A

Sequence: EF70467675

Samples:	16303	Quality: 0 - 9	black
Bases:	623	10 - 19	red
Average spacing:	27.0	20 - 29	yellow
Average quality >=	10: 315, 20: 46, 30: 157	>= 30	green

12.07.2022

500 510 520 530 540 550 560 570

G C T T T T A A T G C T T T G G T A T A A C C G C T A G A C C T G T C C C A G A G G A G C G T T A G T G C A T G A C G T C A G A G T A A G A G A T G C C T C T C

580 590 600 610 620

T A T G G A G A A A T C G T T G T A T A A A C G A C A T A A A T T T T C T T A A T T T T G A C T A
