# Supplementary material for: DNA methylation and lncRNA control asynchronous DNA replication at specific imprinted gene domains
Source: Nat Commun. 2026 Jan 21;17:1844. doi: 10.1038/s41467-026-68558-2 (PMC12920997; doi:10.1038/s41467-026-68558-2)

Sequence: EF72763645

Samples: 32767
Bases: 228
Average spacing: 144.0
Average quality >= 10: 13, 20: 17, 30: 189

Quality: 0 - 9; 10 - 19; 20 - 29; >= 30

07.06.2024

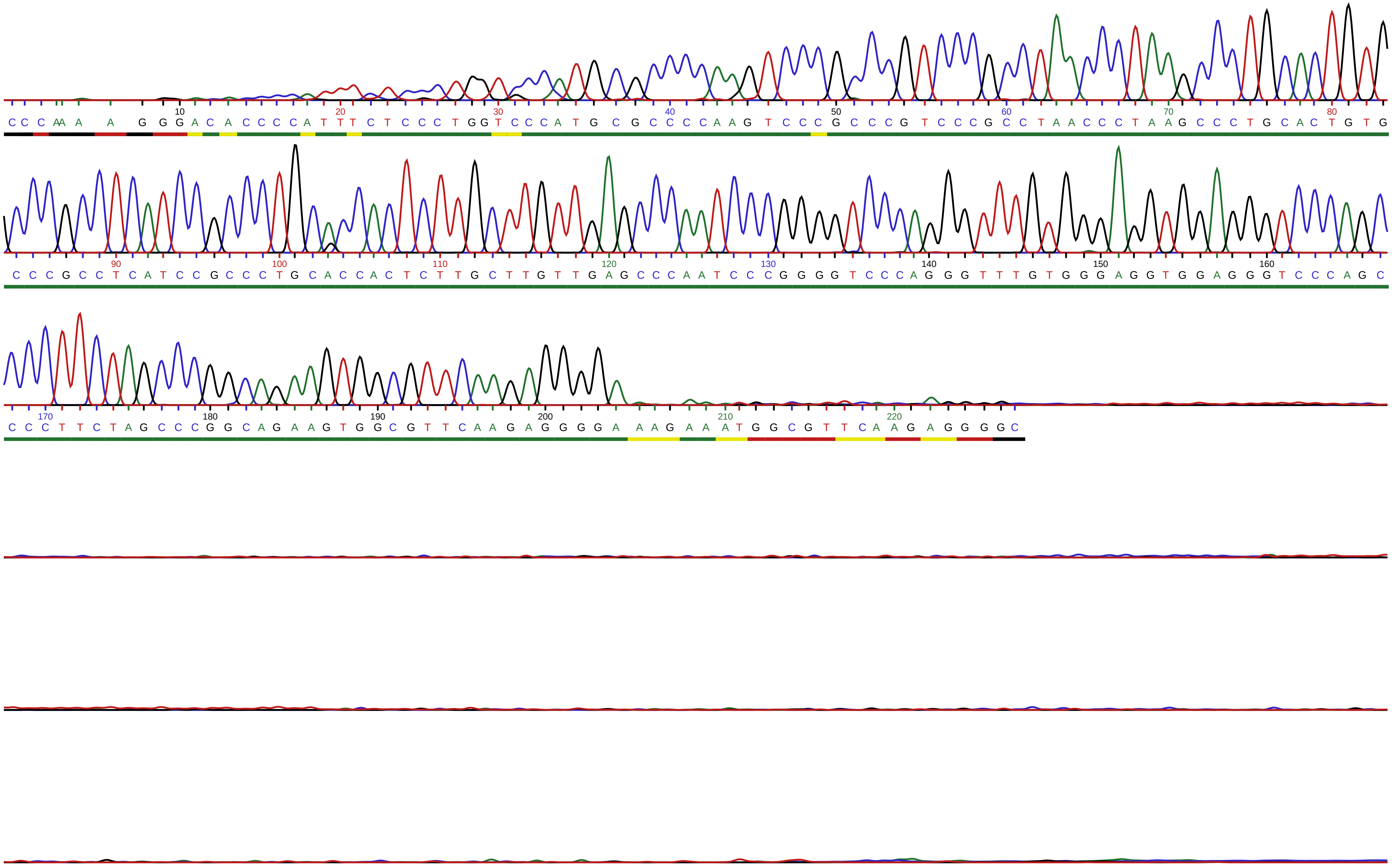

Sequence: EF72763645

Samples:	32767	Quality: 0 - 9	black
Bases:	228	10 - 19	red
Average spacing:	144.0	20 - 29	yellow
Average quality >=	10: 13, 20: 17, 30: 189	>= 30	green

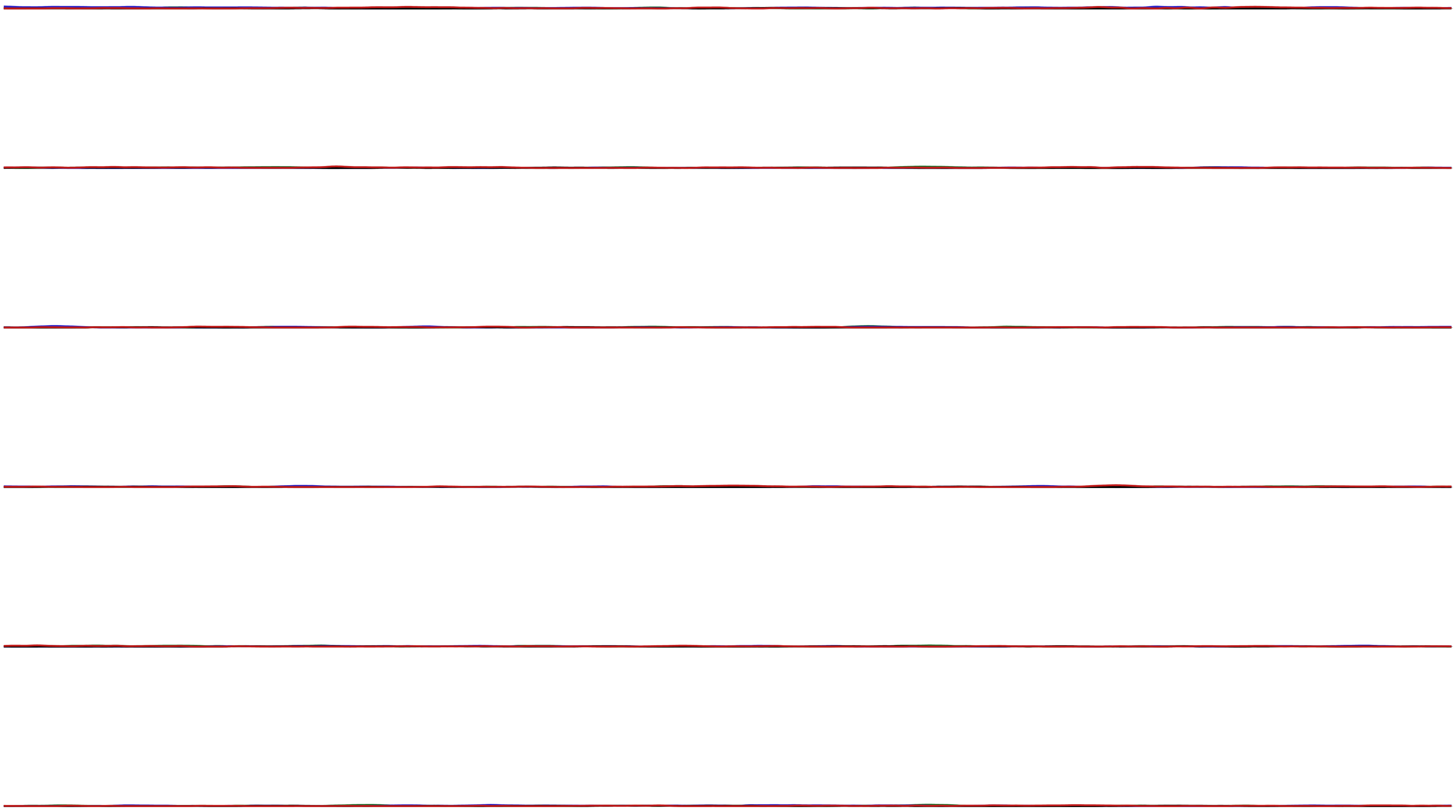

Sequence: EF72763645

Samples:	32767	Quality:	0 - 9		Page: 3 / 6
Bases:	228		10 - 19		07.06.2024
Average spacing:	144.0		20 - 29		
Average quality >=	10: 13, 20: 17, 30: 189		>= 30		

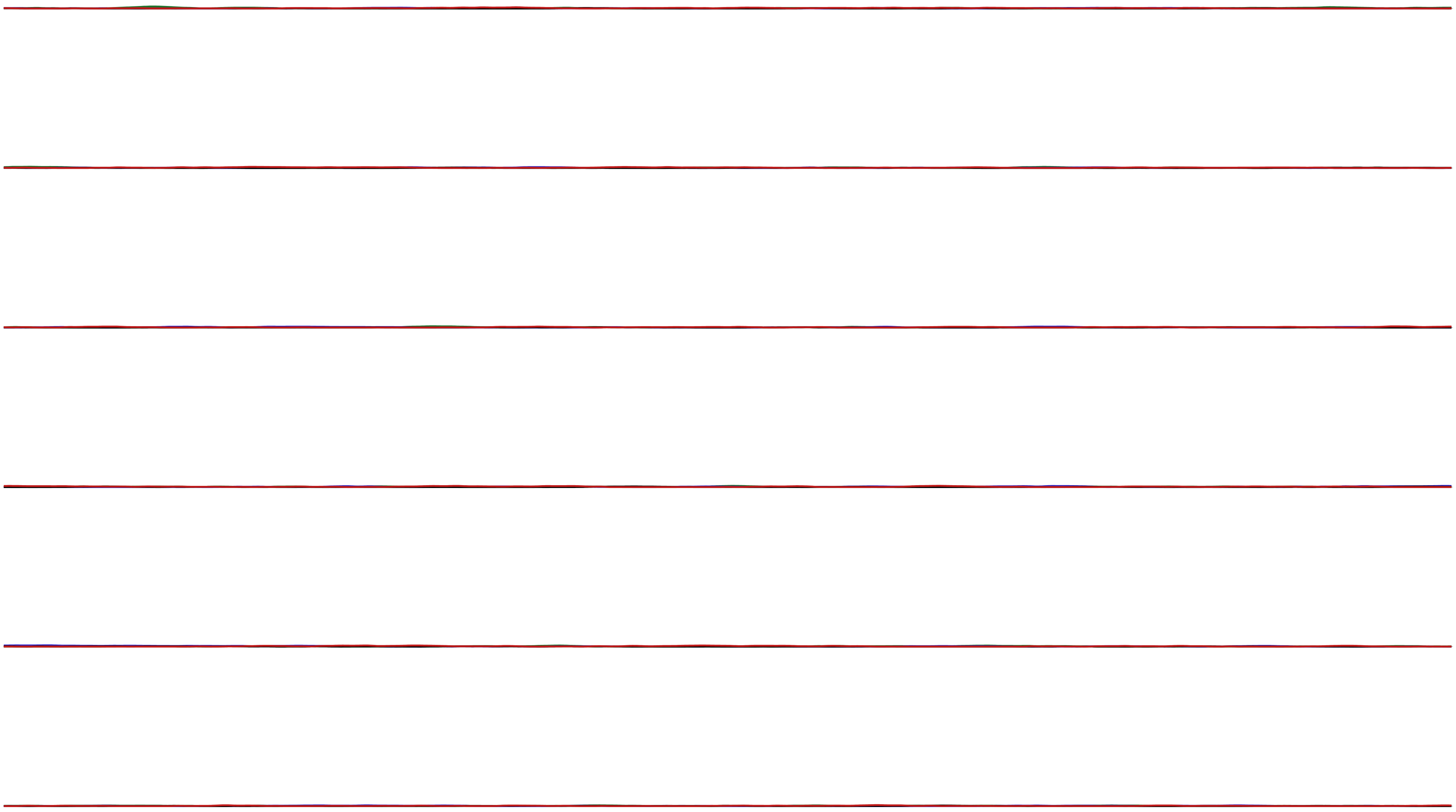

Sequence: EF72763645

Samples:	32767	Quality:	0 - 9	
Bases:	228		10 - 19	
Average spacing:	144.0		20 - 29	
Average quality >=	10: 13, 20: 17, 30: 189		>= 30	

07.06.2024

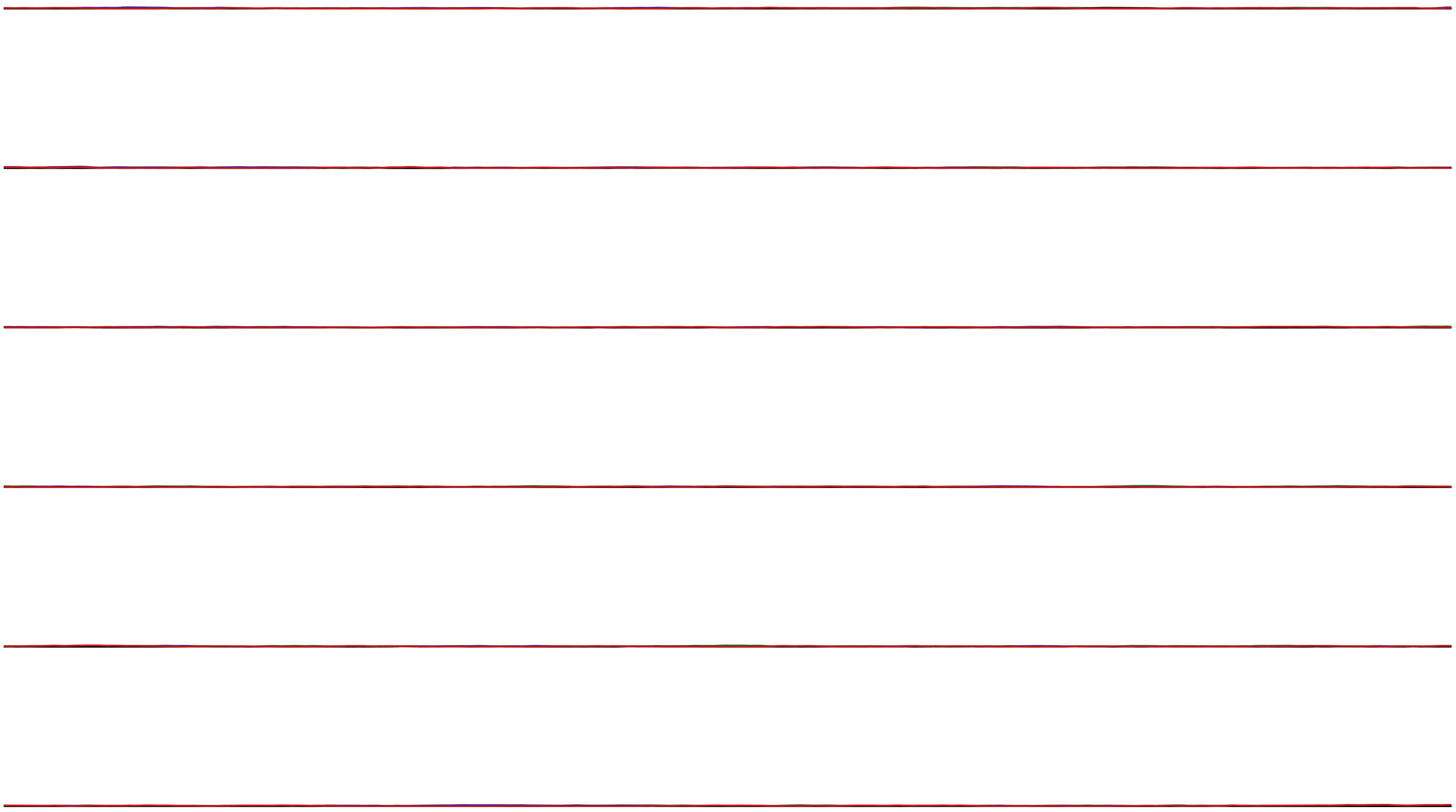

Sequence: EF72763645

Samples:	32767	Quality:	0 - 9	black
Bases:	228		10 - 19	red
Average spacing:	144.0		20 - 29	yellow
Average quality >=	10: 13, 20: 17, 30: 189		>= 30	green

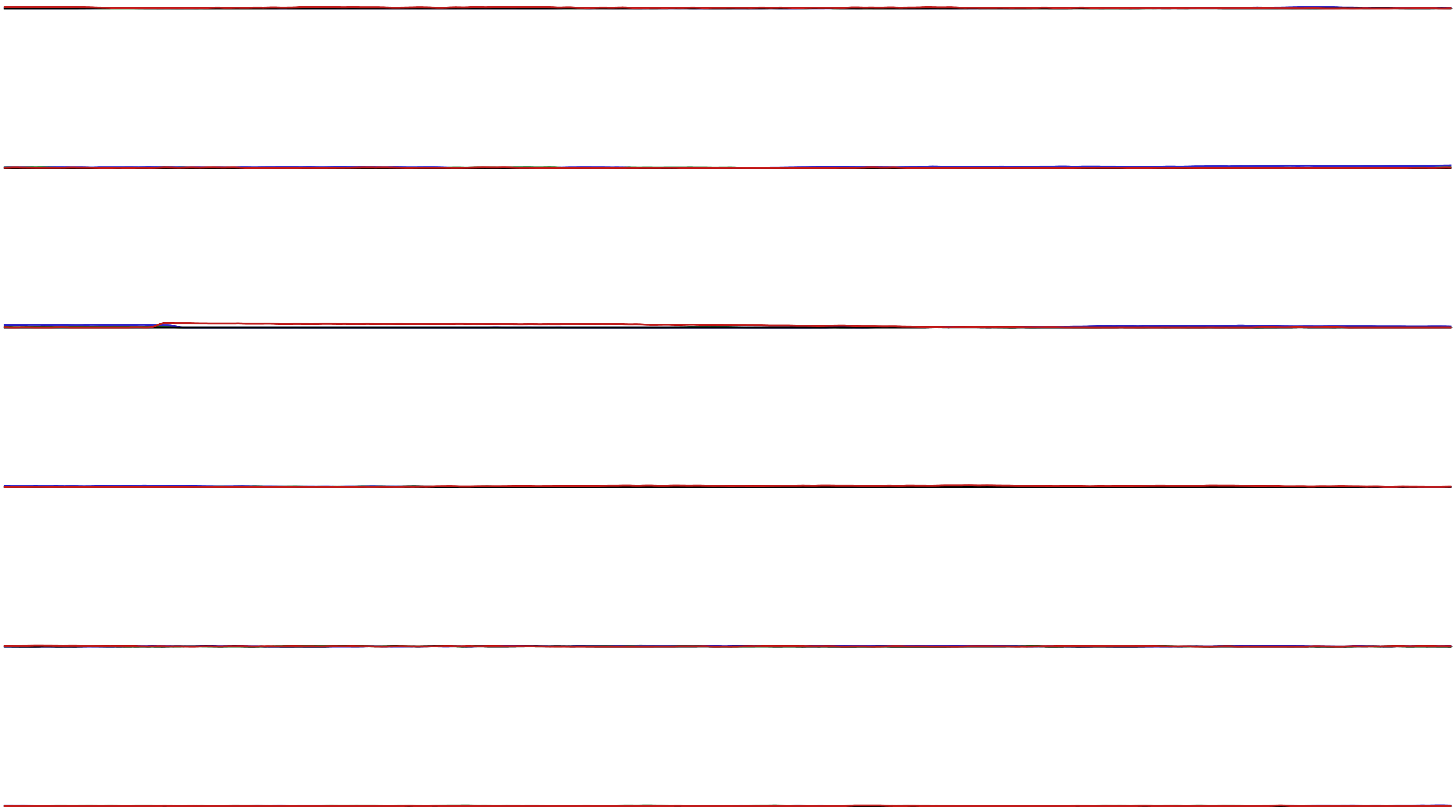

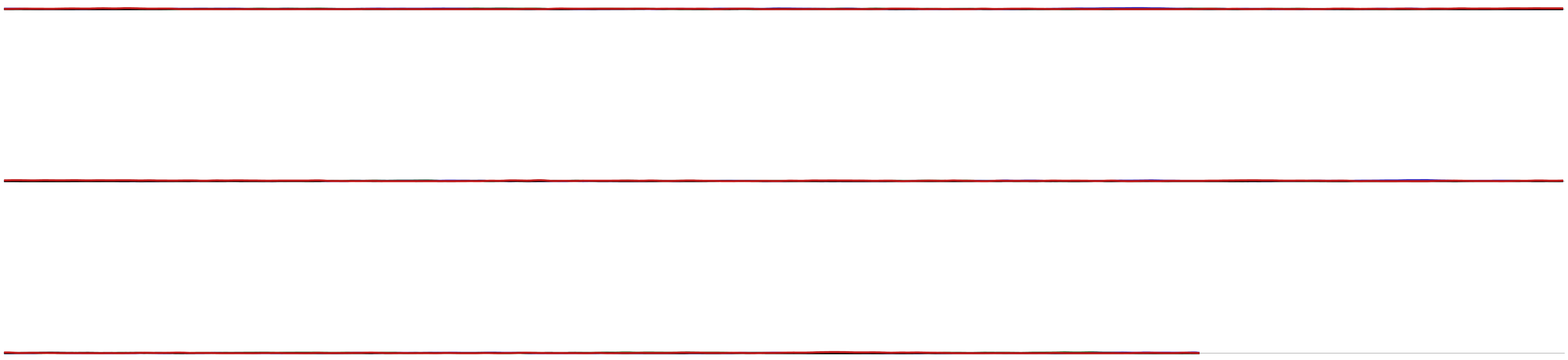

Supplement: Supplementary file 4 — Source data [file 41467_2026_68558_MOESM4_ESM.zip › Source data/Sanger-sequencing data/Suppl.Fig6g/in1-early-Dlk1.pdf]
