# Supplementary figures and images for: DNA methylation and lncRNA control asynchronous DNA replication at specific imprinted gene domains
Source: Nat Commun. 2026 Jan 21;17:1844. doi: 10.1038/s41467-026-68558-2 (PMC12920997; doi:10.1038/s41467-026-68558-2)

# BD FACSDiva 9.0.1

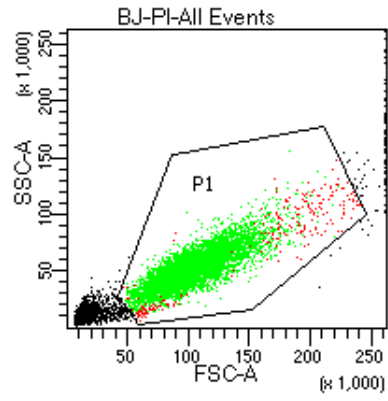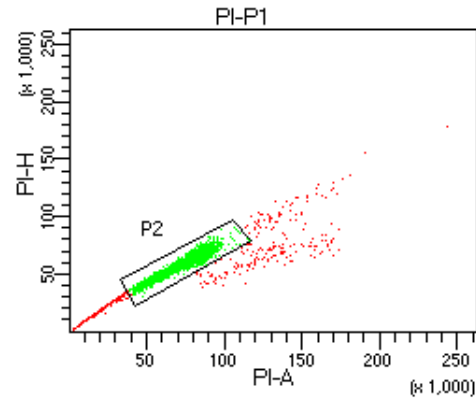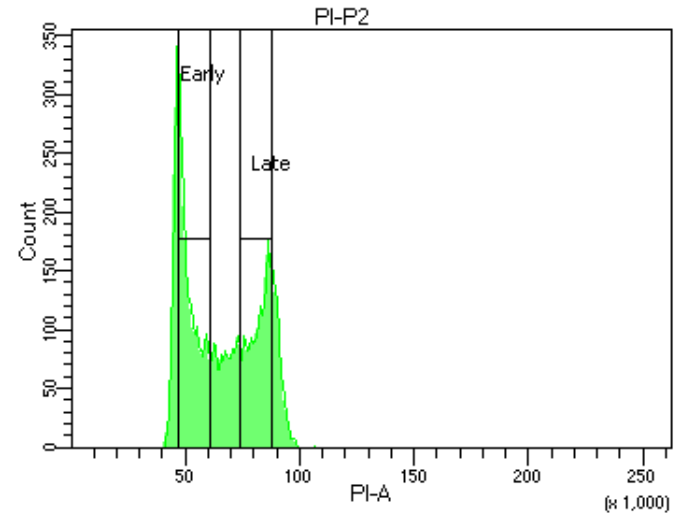

Tube: PI

| Population | #Events | %Parent | %Total |
|------------|---------|---------|--------|
| All Events | 10,000  | ####    | 100.0  |
| P1         | 6,308   | 63.1    | 63.1   |
| P2         | 5,934   | 94.1    | 59.3   |
| Early      | 1,541   | 26.0    | 15.4   |
| Late       | 1,566   | 26.4    | 15.7   |

Supplement: Supplementary file 4 — Source data [file 41467_2026_68558_MOESM4_ESM.zip › Source data/FACS data/Suppl.Fig1b/FACS profile -BJ.pdf]

# BD FACSDiva 9.0.1

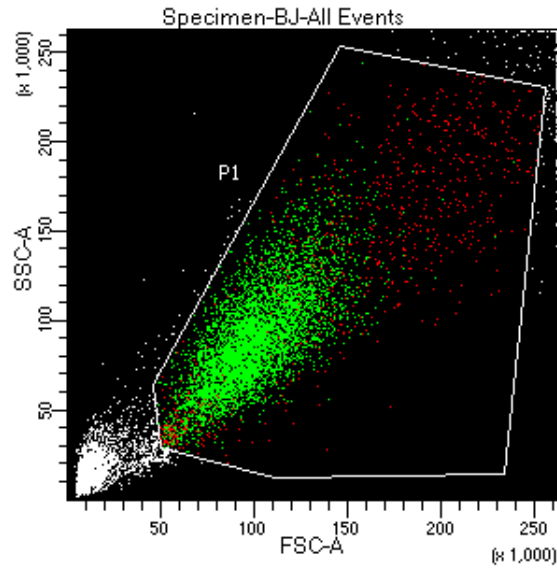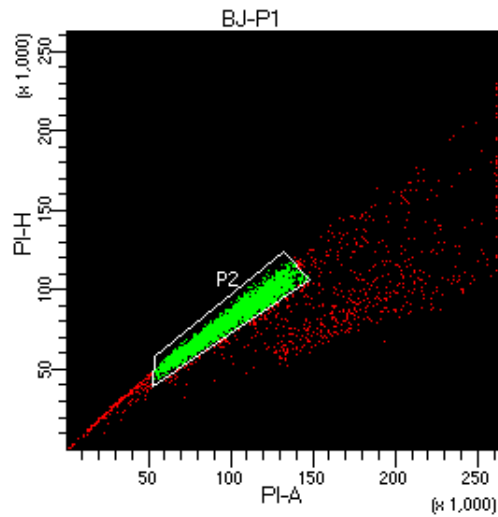

Tube: BJ

| Population | #Events | %Parent | %Total |
|------------|---------|---------|--------|
| All Events | 10,000  | ####    | 100.0  |
| P1         | 5,838   | 58.4    | 58.4   |
| P2         | 4,977   | 85.3    | 49.8   |
| G1         | 1,035   | 20.8    | 10.4   |
| earlyS     | 472     | 9.5     | 4.7    |
| lateS      | 435     | 8.7     | 4.4    |
| G2         | 525     | 10.5    | 5.2    |

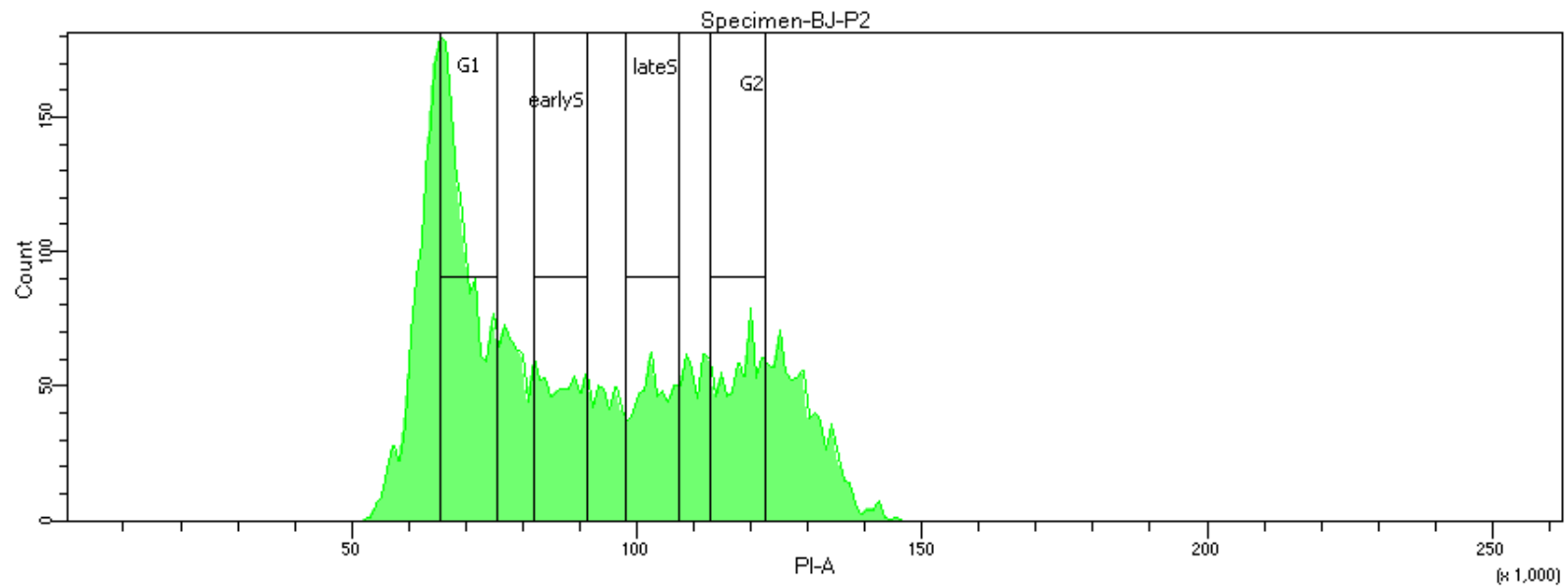

Supplement: Supplementary file 4 — Source data [file 41467_2026_68558_MOESM4_ESM.zip › Source data/FACS data/Suppl.Fig2d/BJ-4fractions.pdf]

# BD FACSDiva 9.0.1

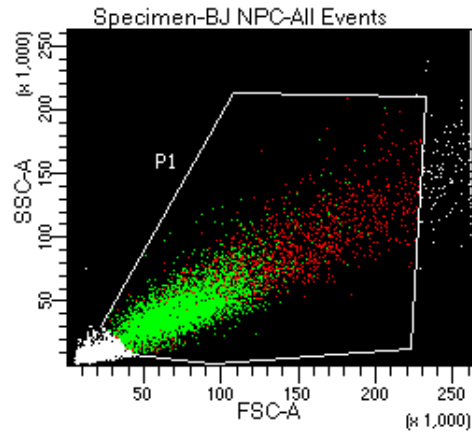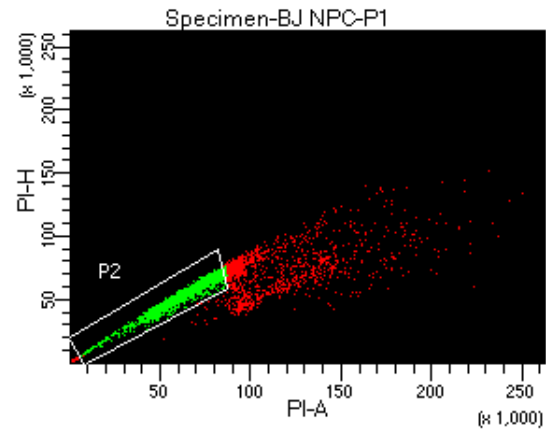

Tube: BJ NPC

| Population | #Events | %Parent | %Total |
|------------|---------|---------|--------|
| All Events | 10,000  | ####    | 100.0  |
| P1         | 5,415   | 54.2    | 54.2   |
| P2         | 4,036   | 74.5    | 40.4   |
| early      | 1,544   | 38.3    | 15.4   |
| late       | 511     | 12.7    | 5.1    |

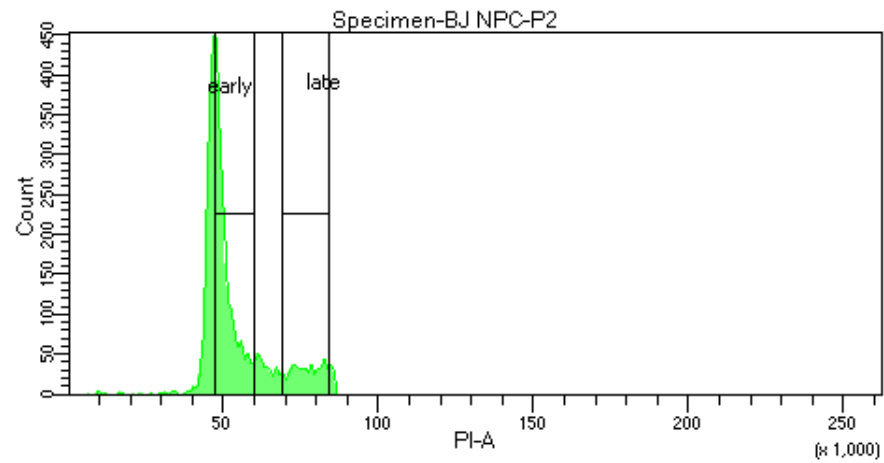

Supplement: Supplementary file 4 — Source data [file 41467_2026_68558_MOESM4_ESM.zip › Source data/FACS data/Suppl.Fig8a/BJ NPC.pdf]

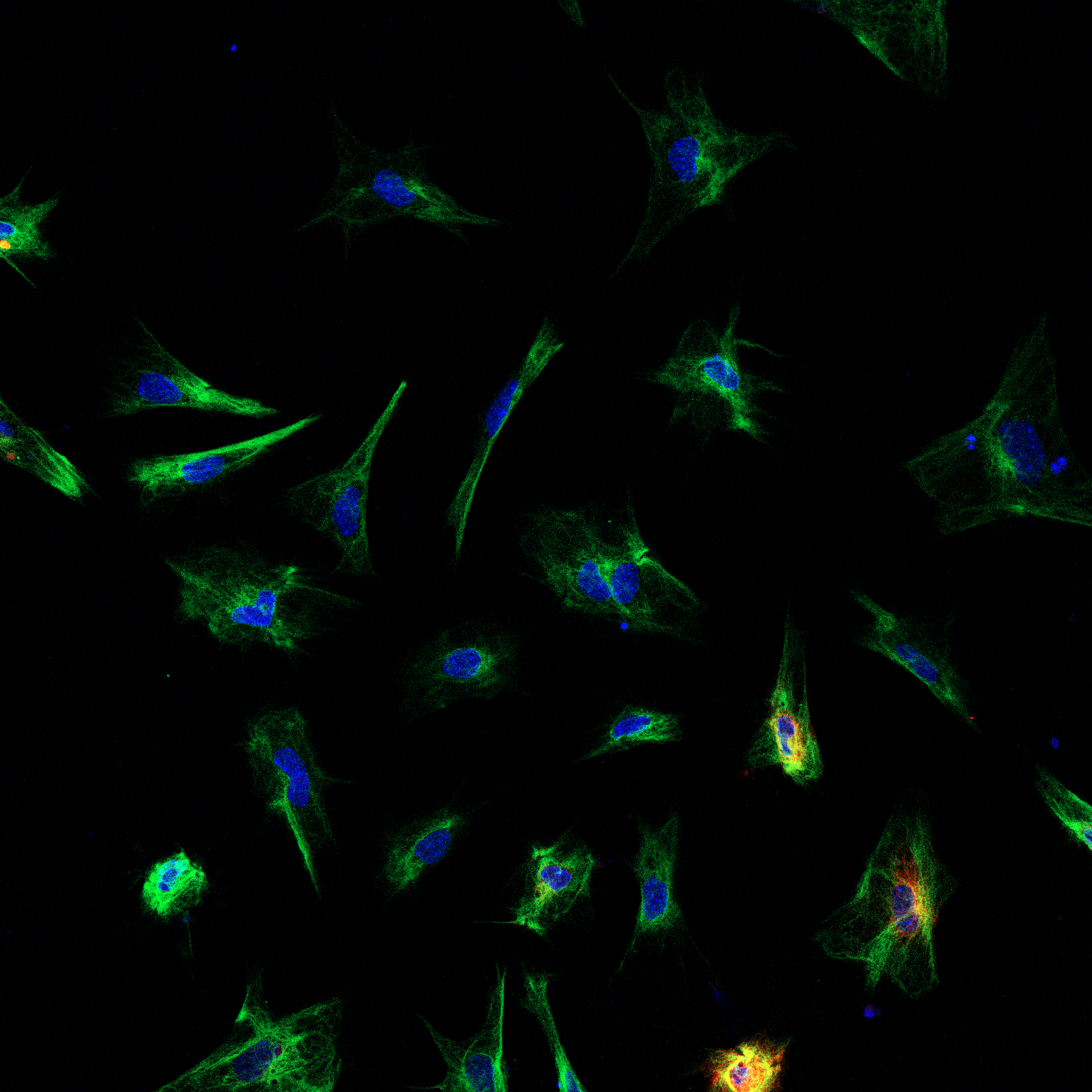

Supplement: Supplementary file 4 — Source data [file 41467_2026_68558_MOESM4_ESM.zip › Source data/immunofluorescence data/Nestin-Tuj1-merge (Fig. 7b)..tif]

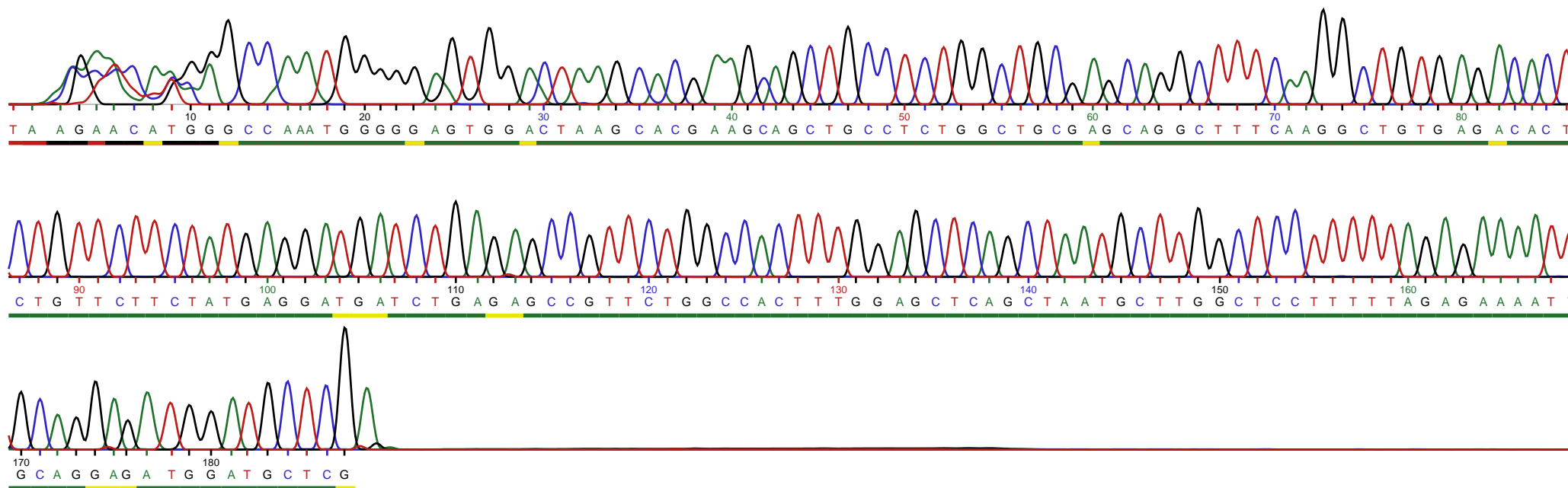

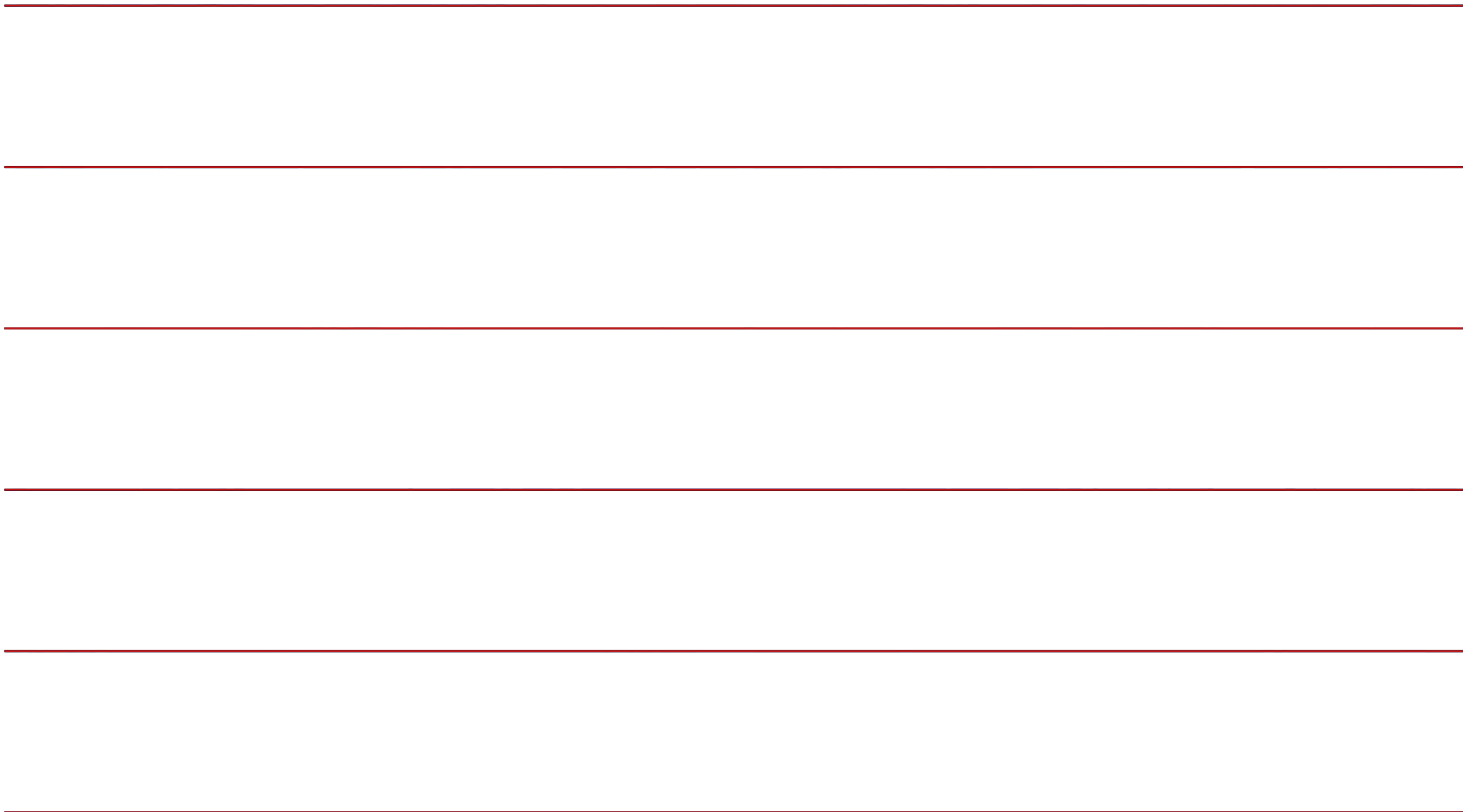

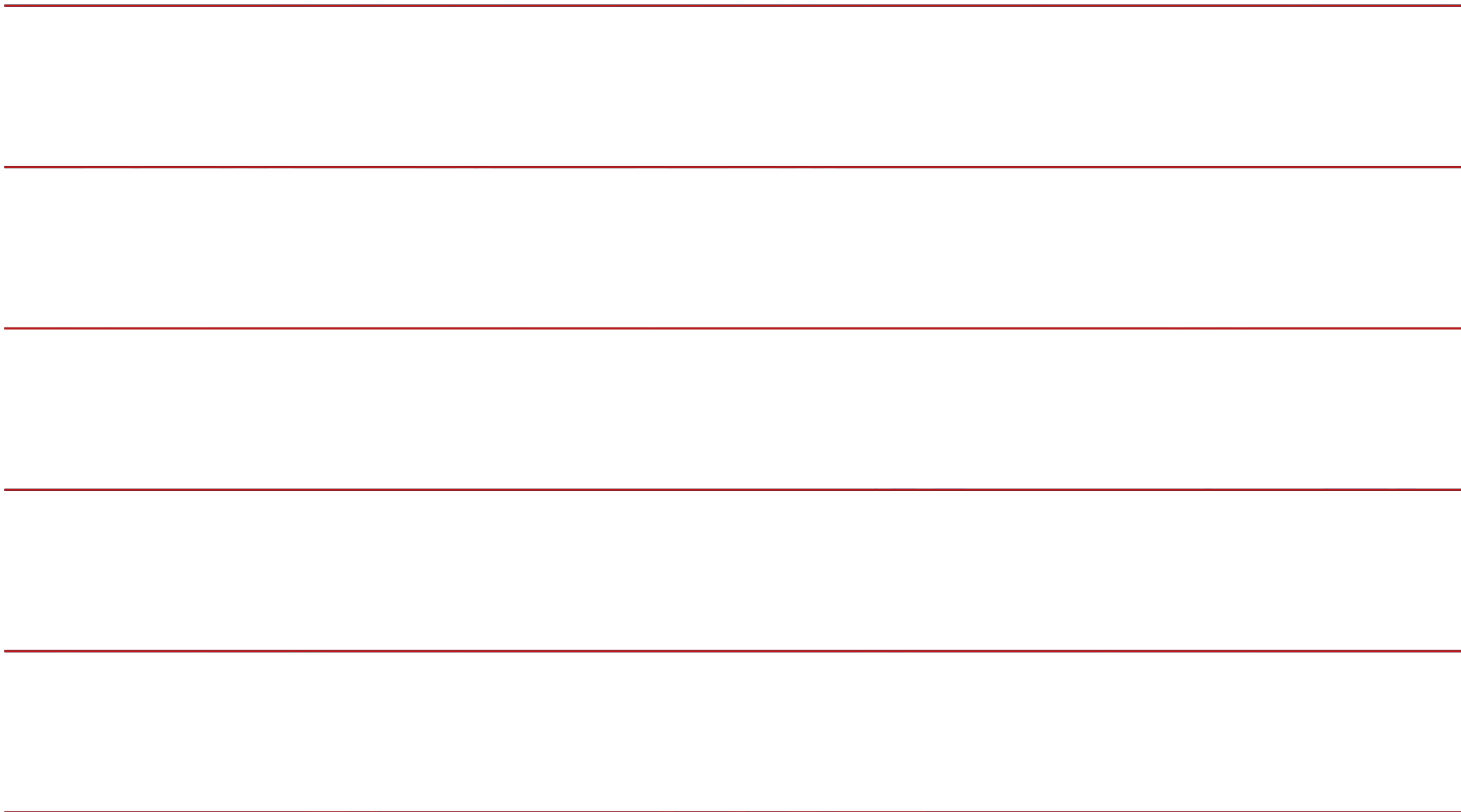

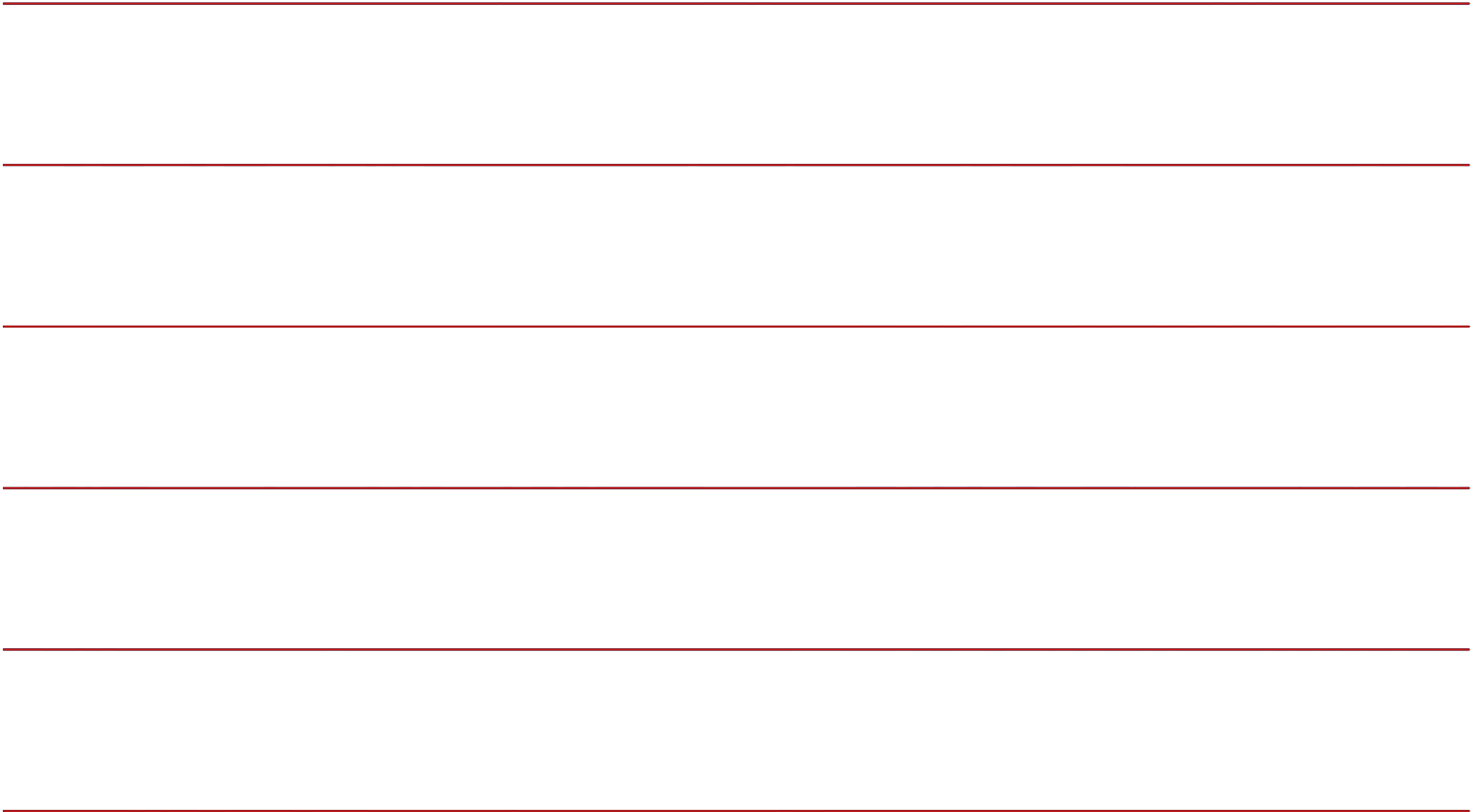

Sequence: EF70467478

Samples: 24236  
Bases: 188  
Average spacing: 129.0  
Average quality >= 10: 4, 20: 15, 30: 162

Quality: 0 - 9 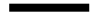  
10 - 19 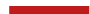  
20 - 29 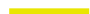  
>= 30 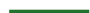

Page: 5 / 5  
06.10.2022

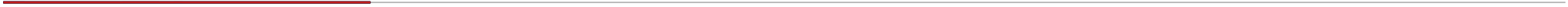

Supplement: Supplementary file 4 — Source data [file 41467_2026_68558_MOESM4_ESM.zip › Source data/Sanger-sequencing data/Fig2c-d/BJ early-Meg3.pdf]

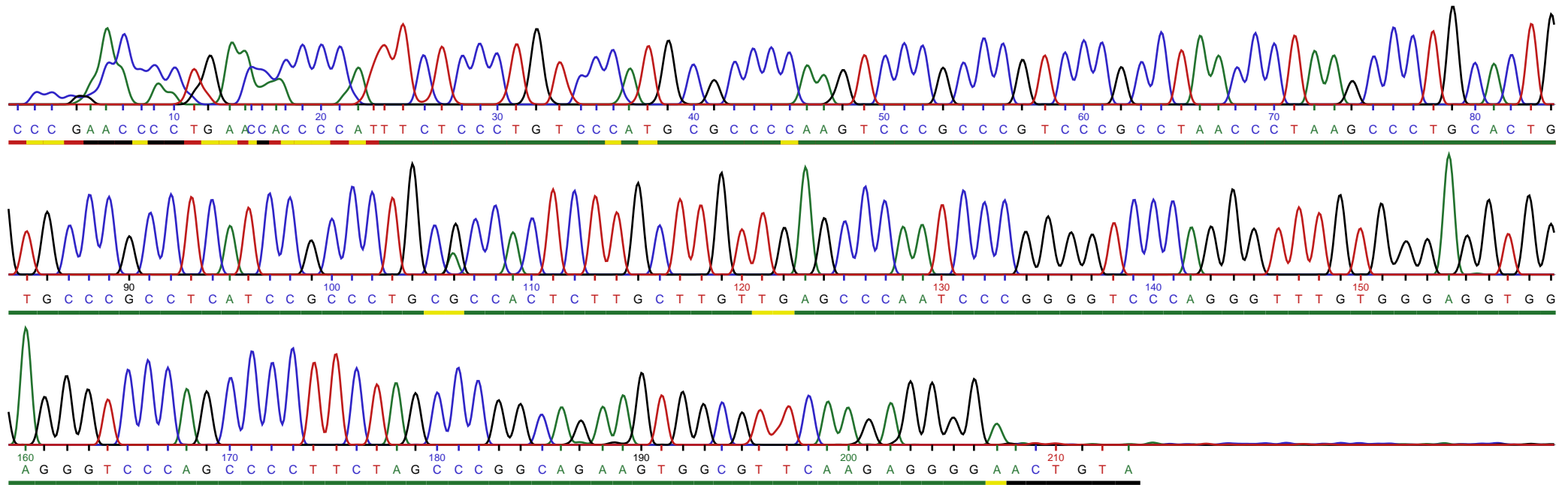

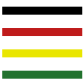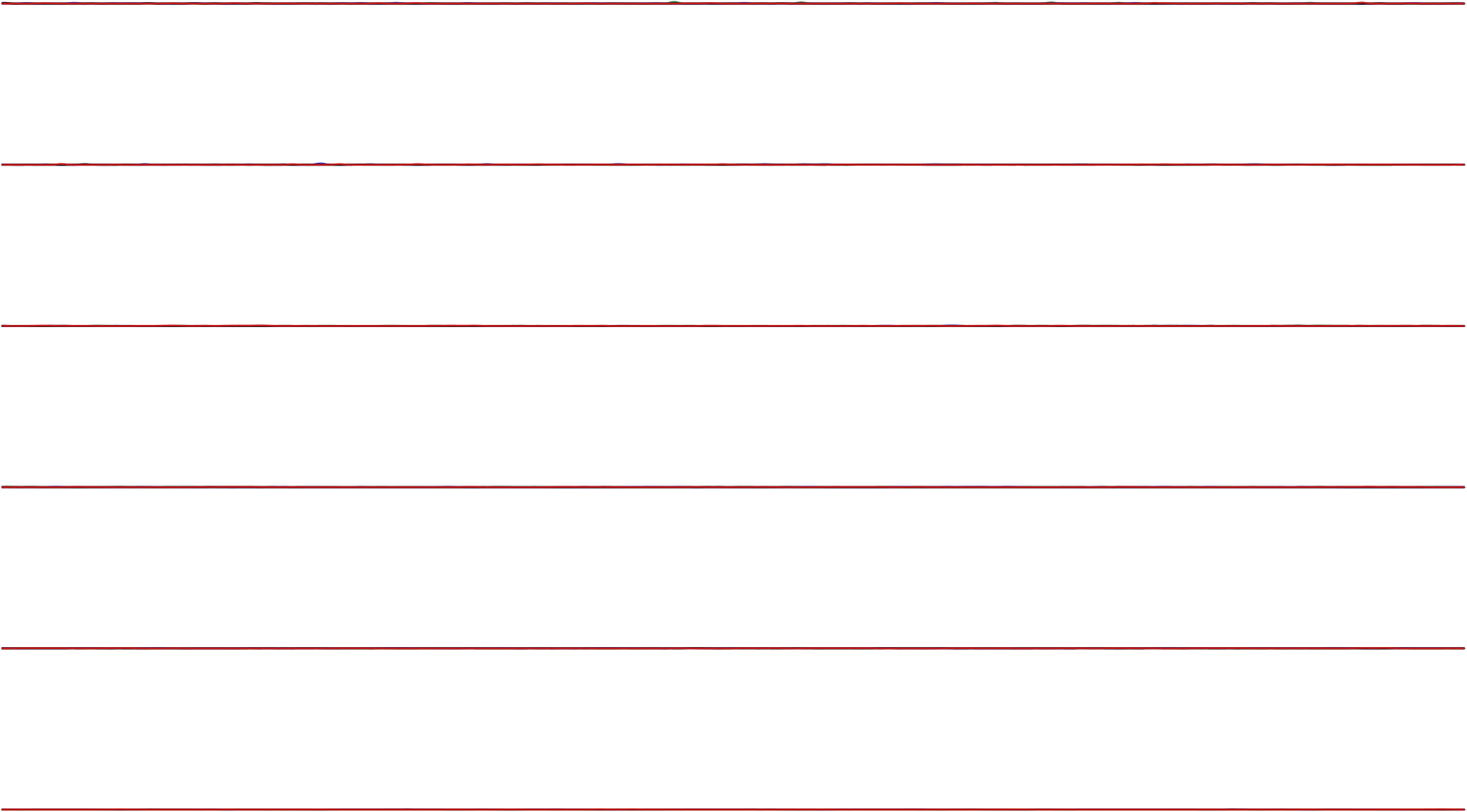

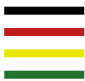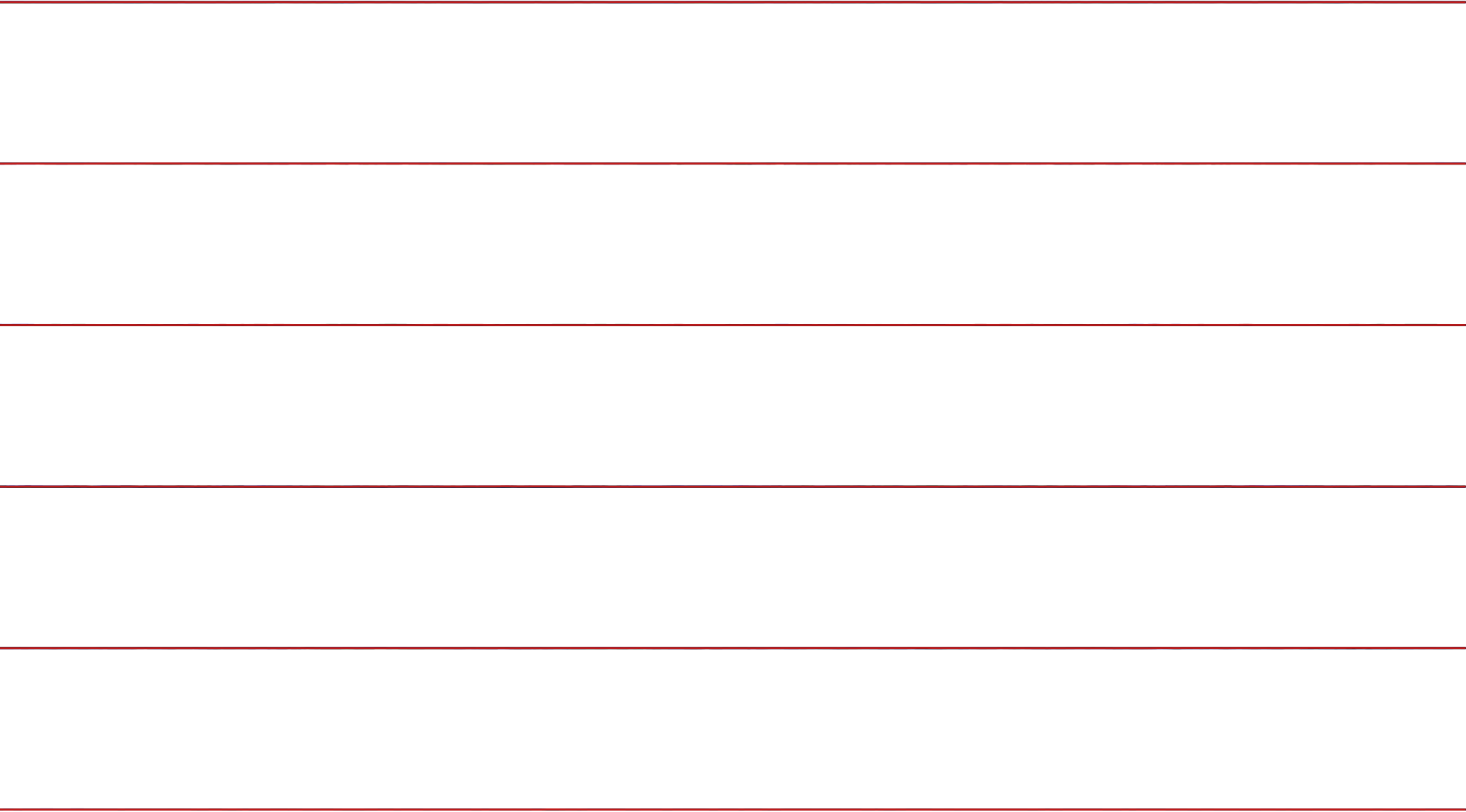

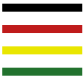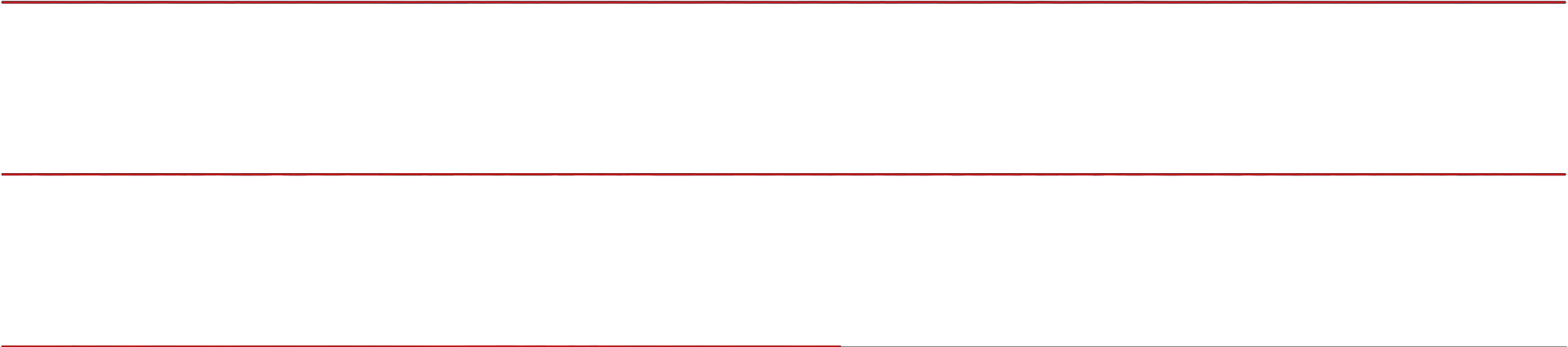

Supplement: Supplementary file 4 — Source data [file 41467_2026_68558_MOESM4_ESM.zip › Source data/Sanger-sequencing data/Fig2c-d/BJ late-Dlk1.pdf]

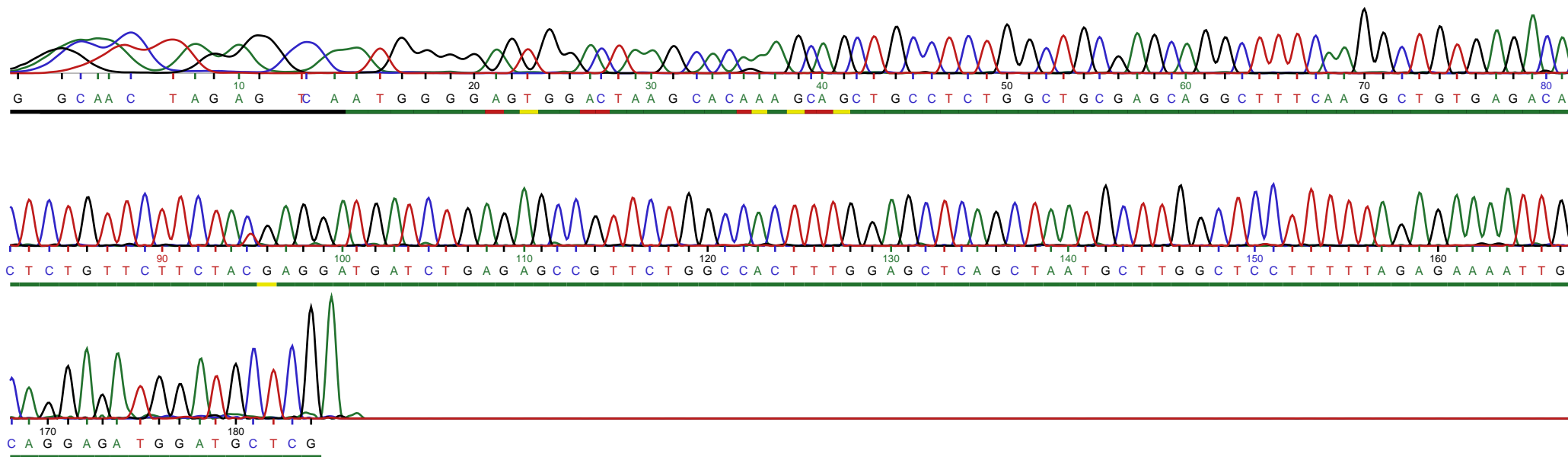

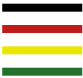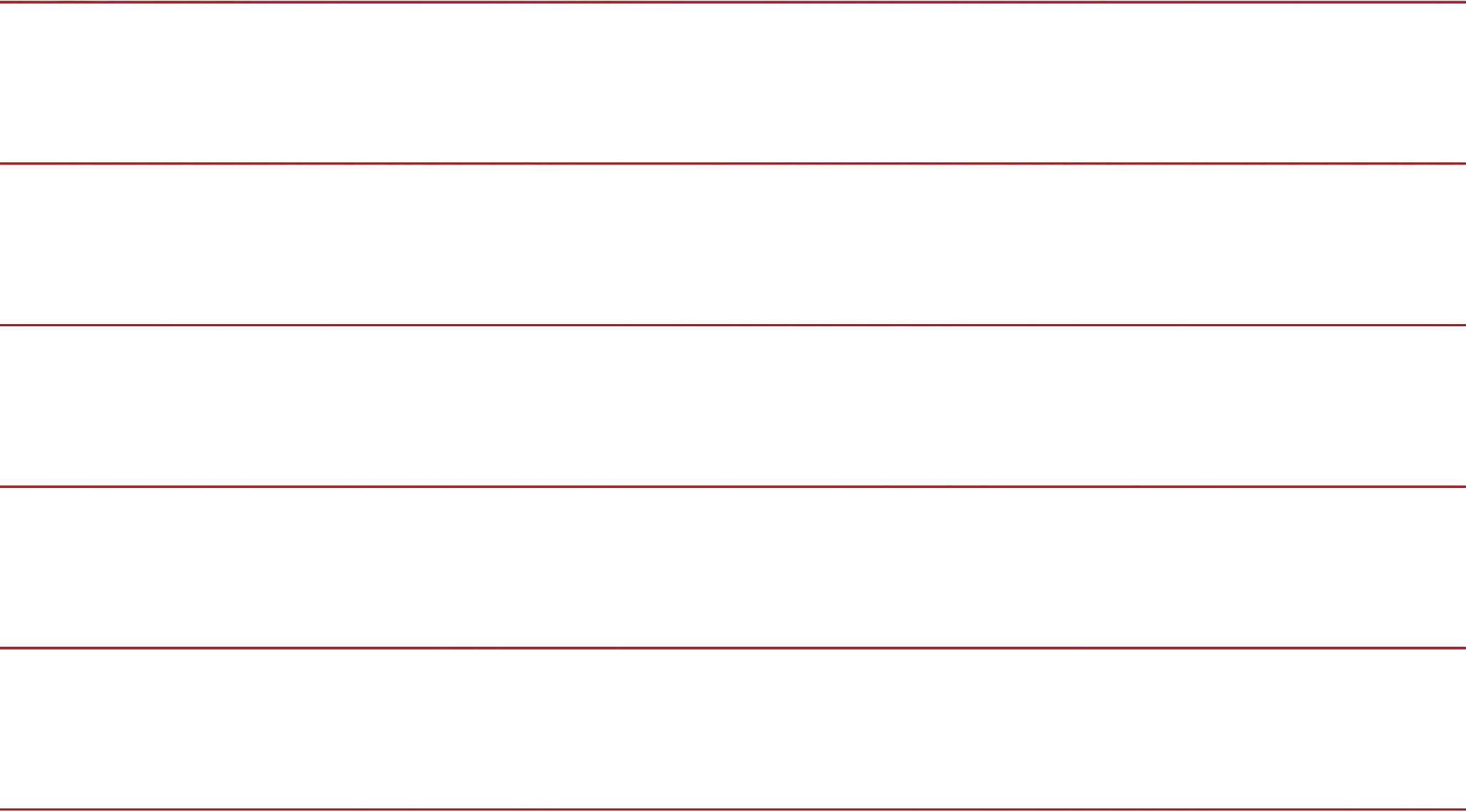

---

---

---

Supplement: Supplementary file 4 — Source data [file 41467_2026_68558_MOESM4_ESM.zip › Source data/Sanger-sequencing data/Fig2c-d/BJ late-Meg3.pdf]

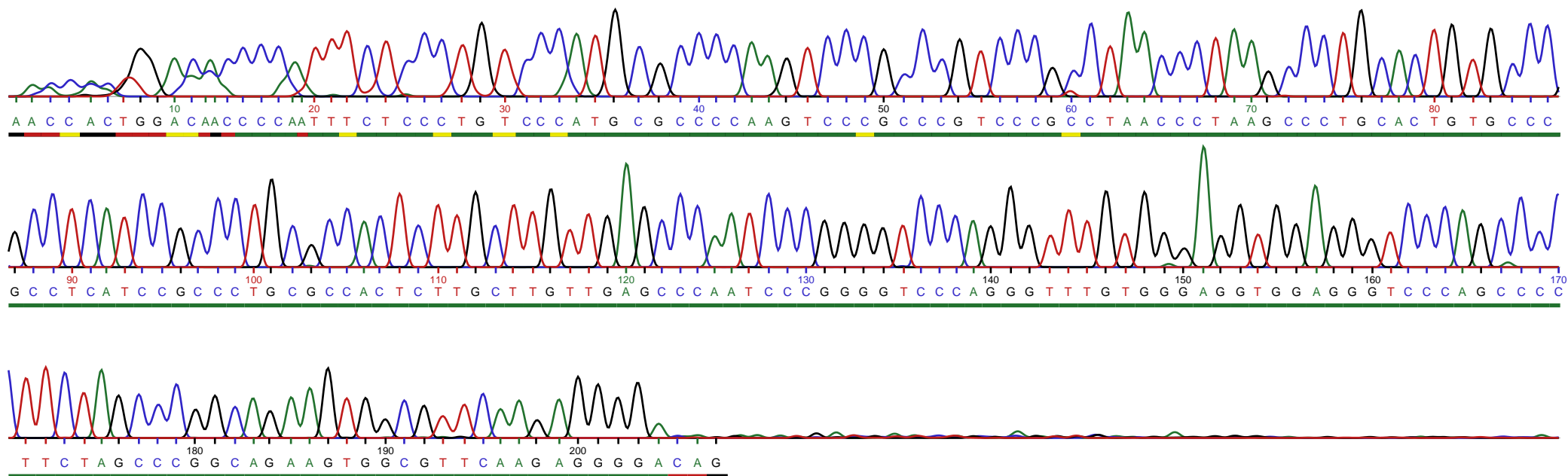

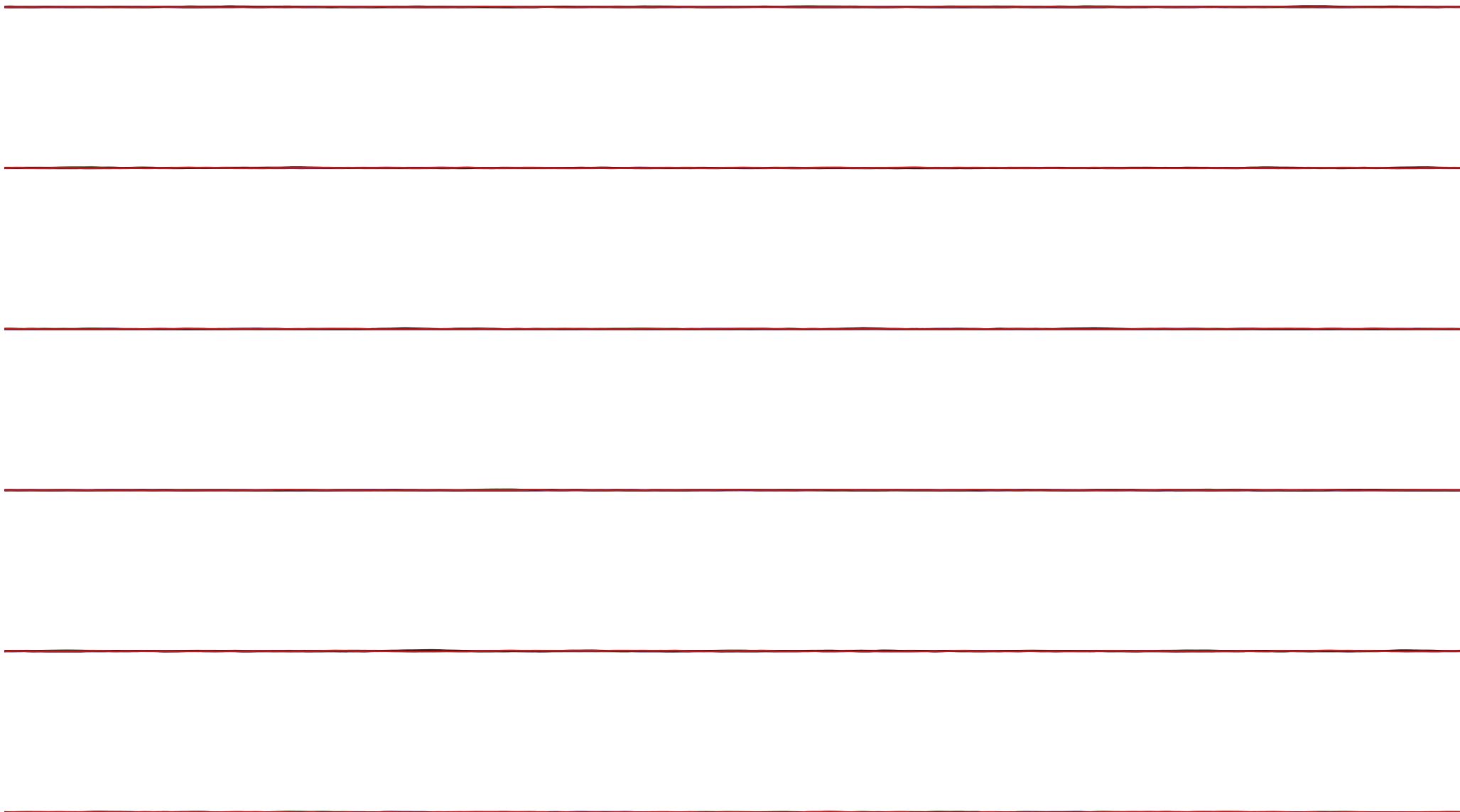

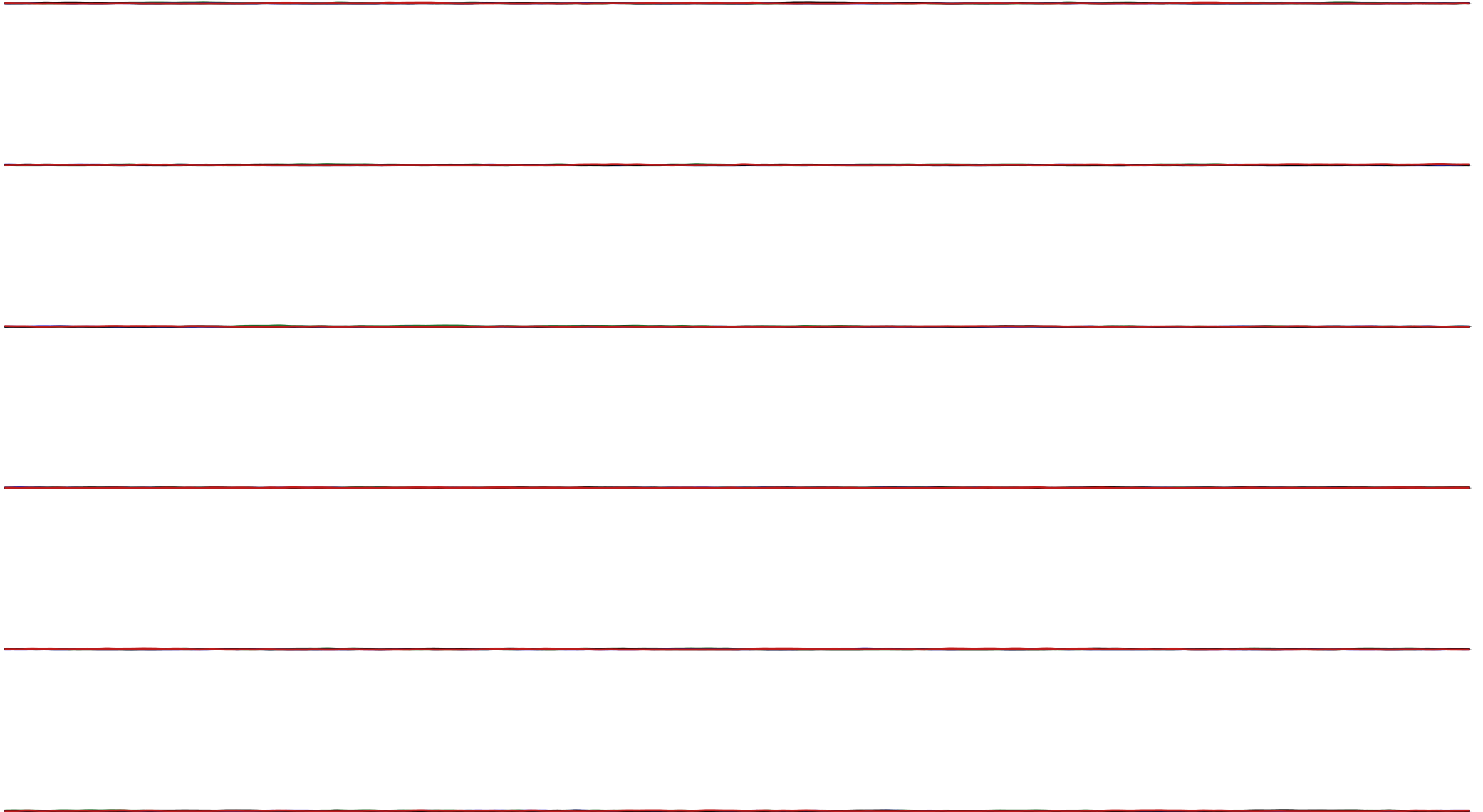

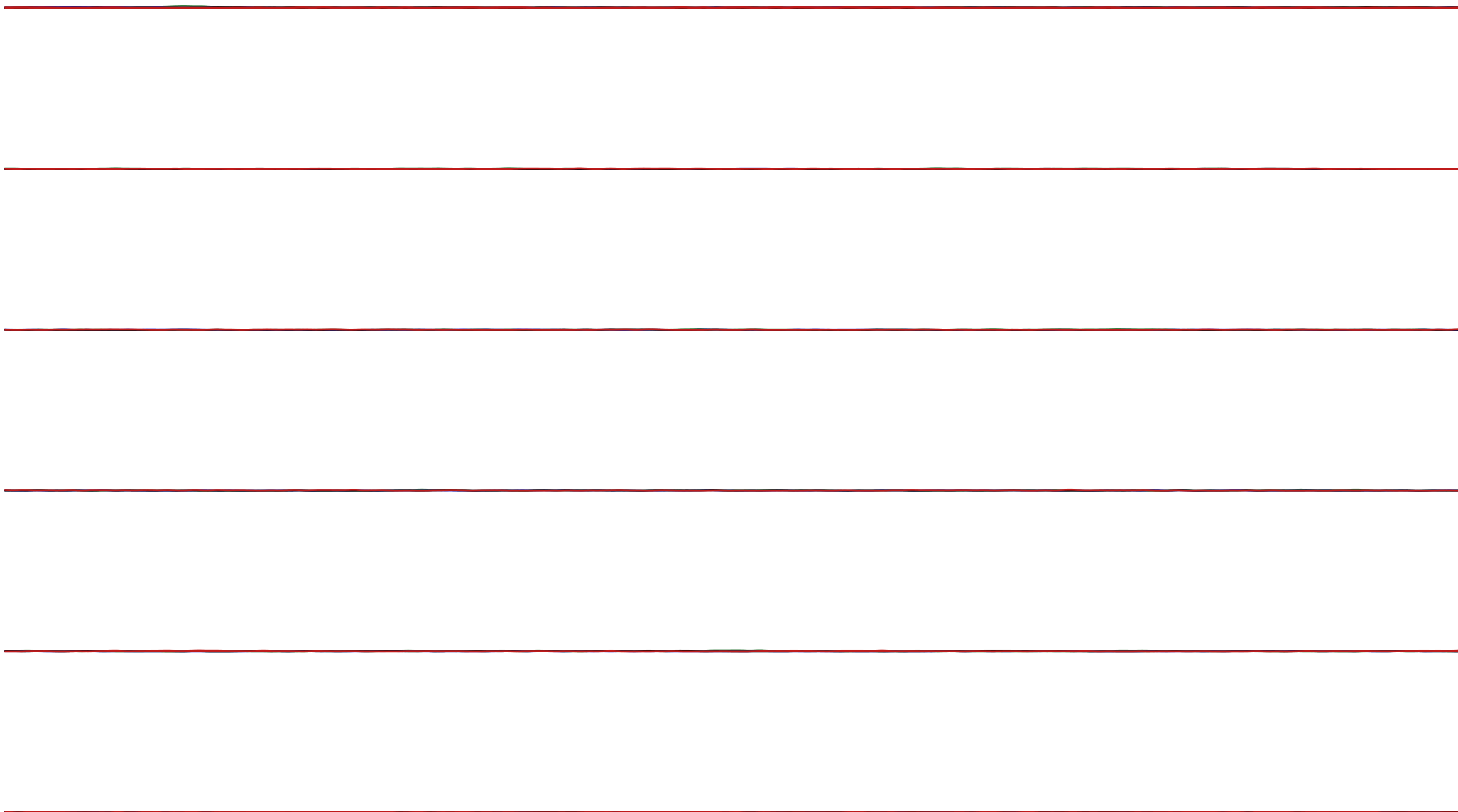

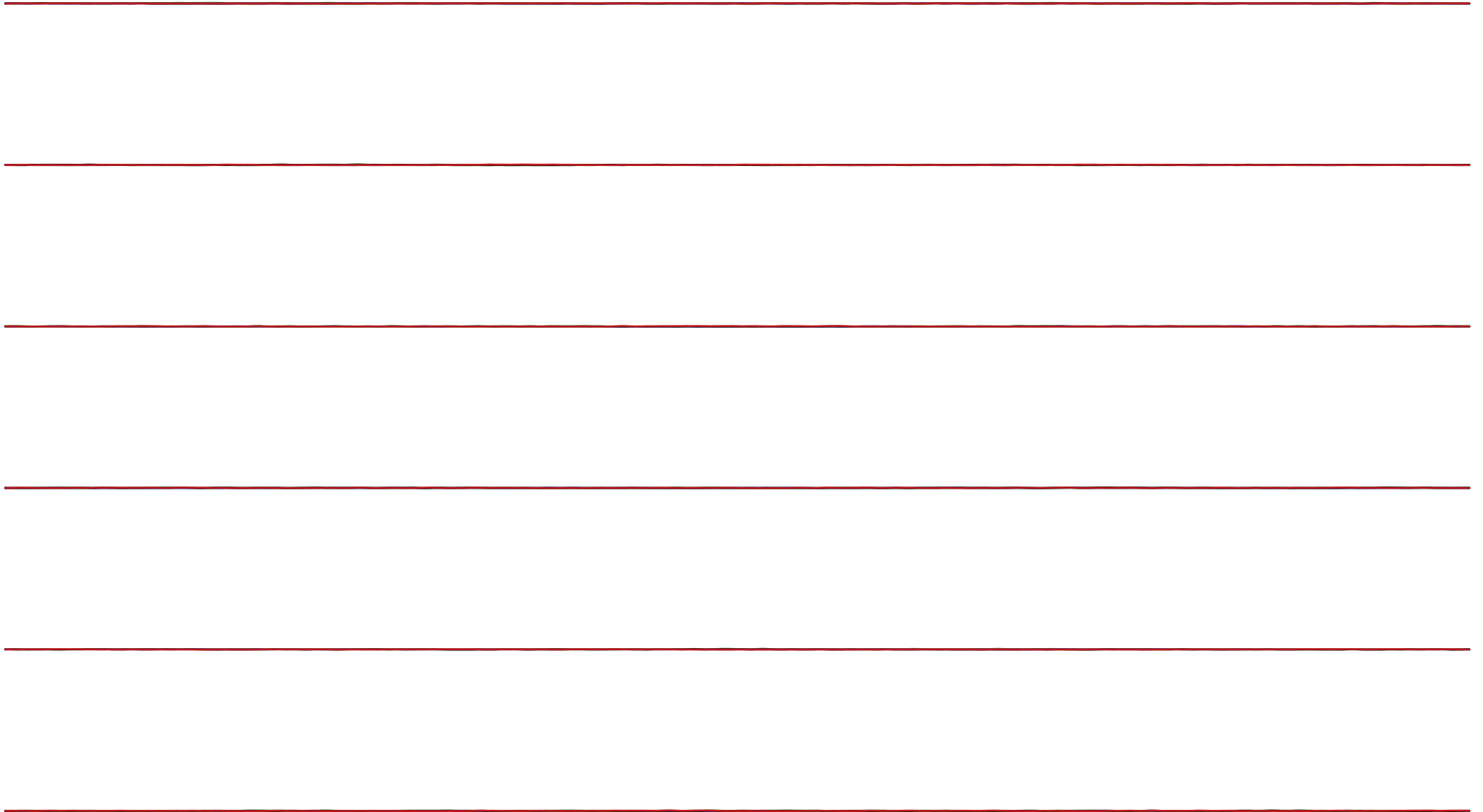

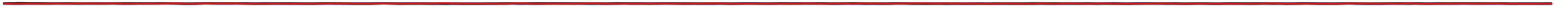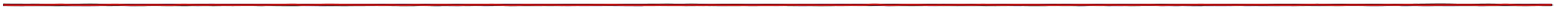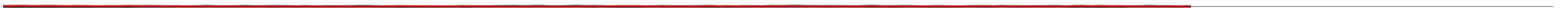

Supplement: Supplementary file 4 — Source data [file 41467_2026_68558_MOESM4_ESM.zip › Source data/Sanger-sequencing data/Fig2c-d/JB early-Dlk1.pdf]

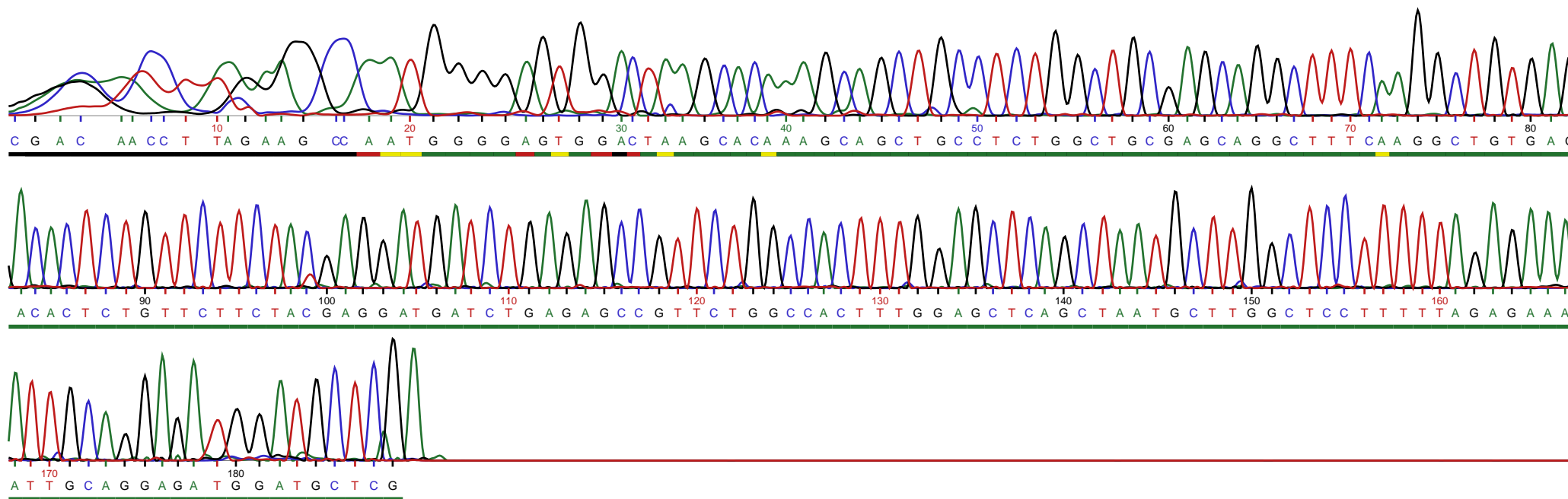

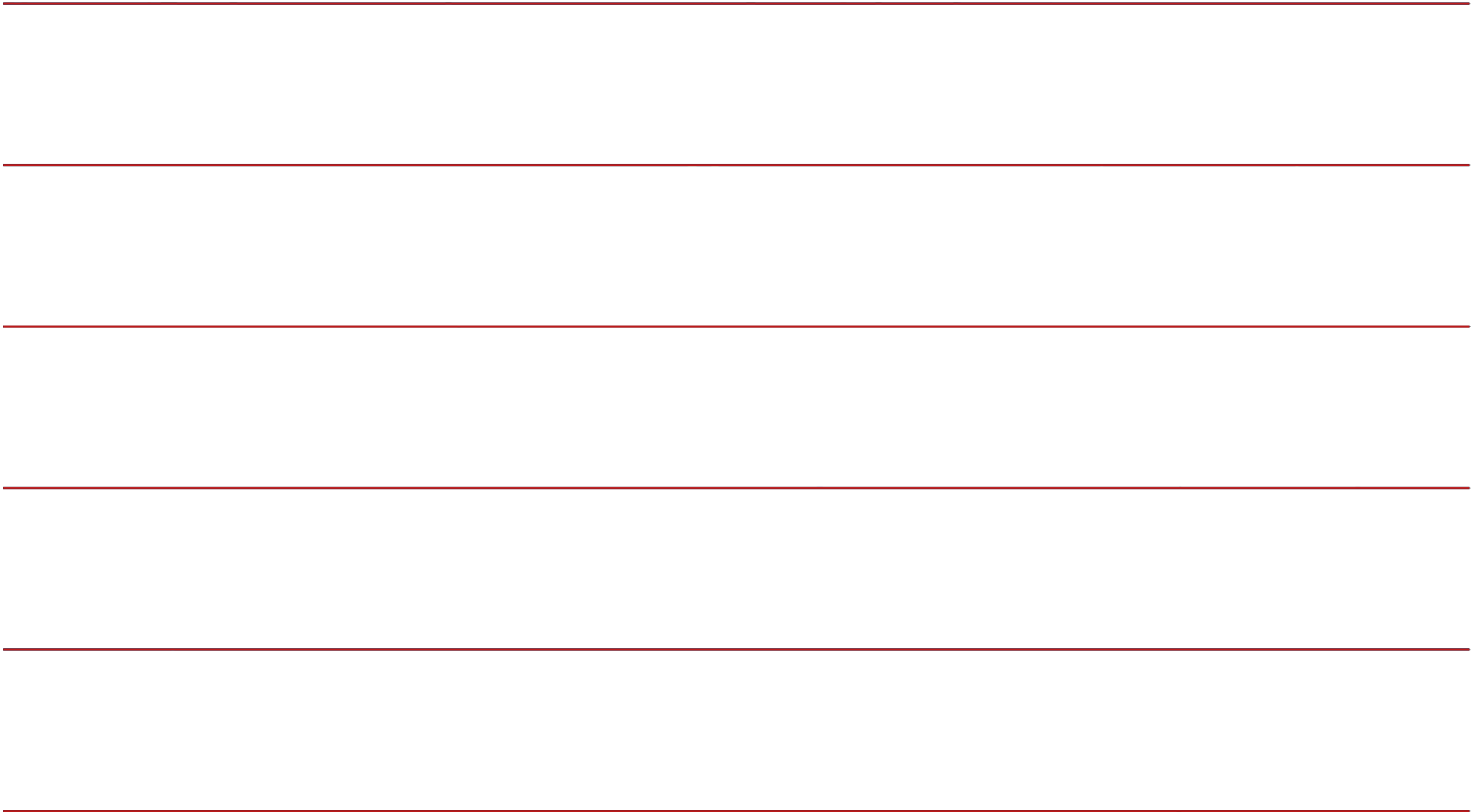

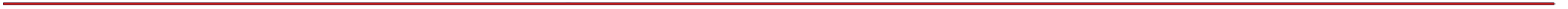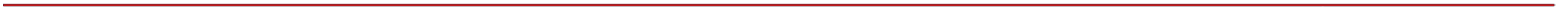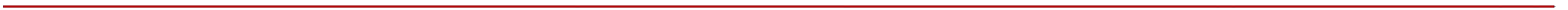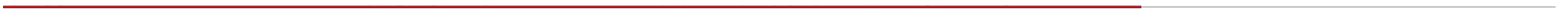

Supplement: Supplementary file 4 — Source data [file 41467_2026_68558_MOESM4_ESM.zip › Source data/Sanger-sequencing data/Fig2c-d/JB early-Meg3.pdf]

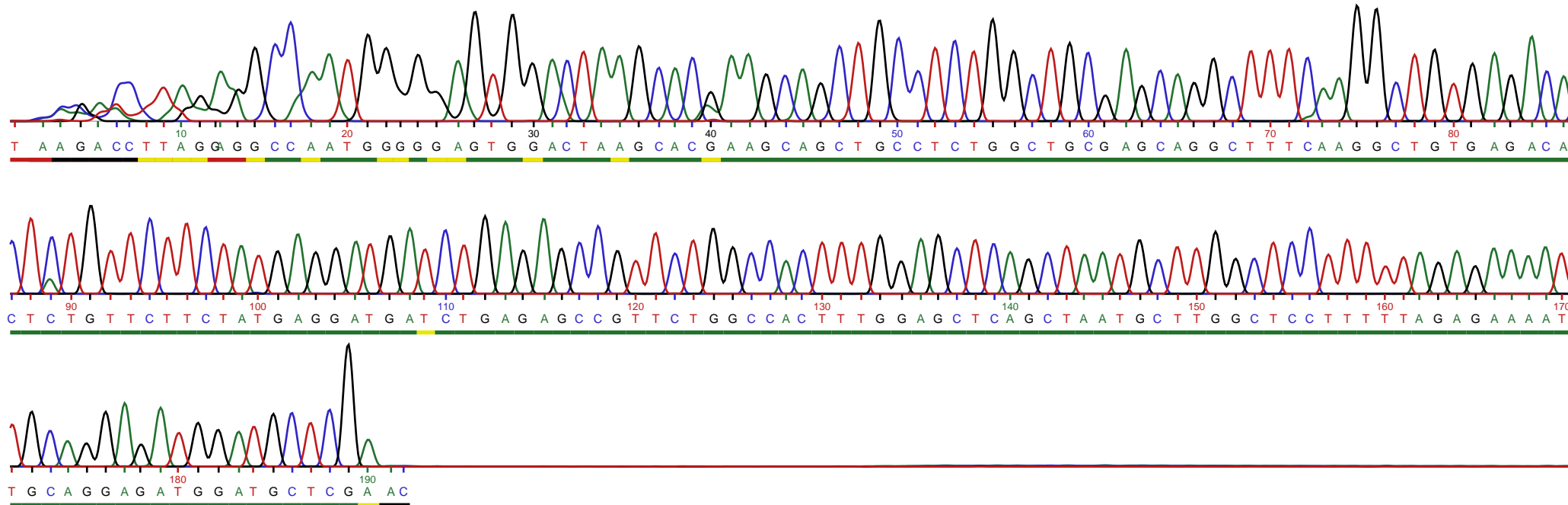

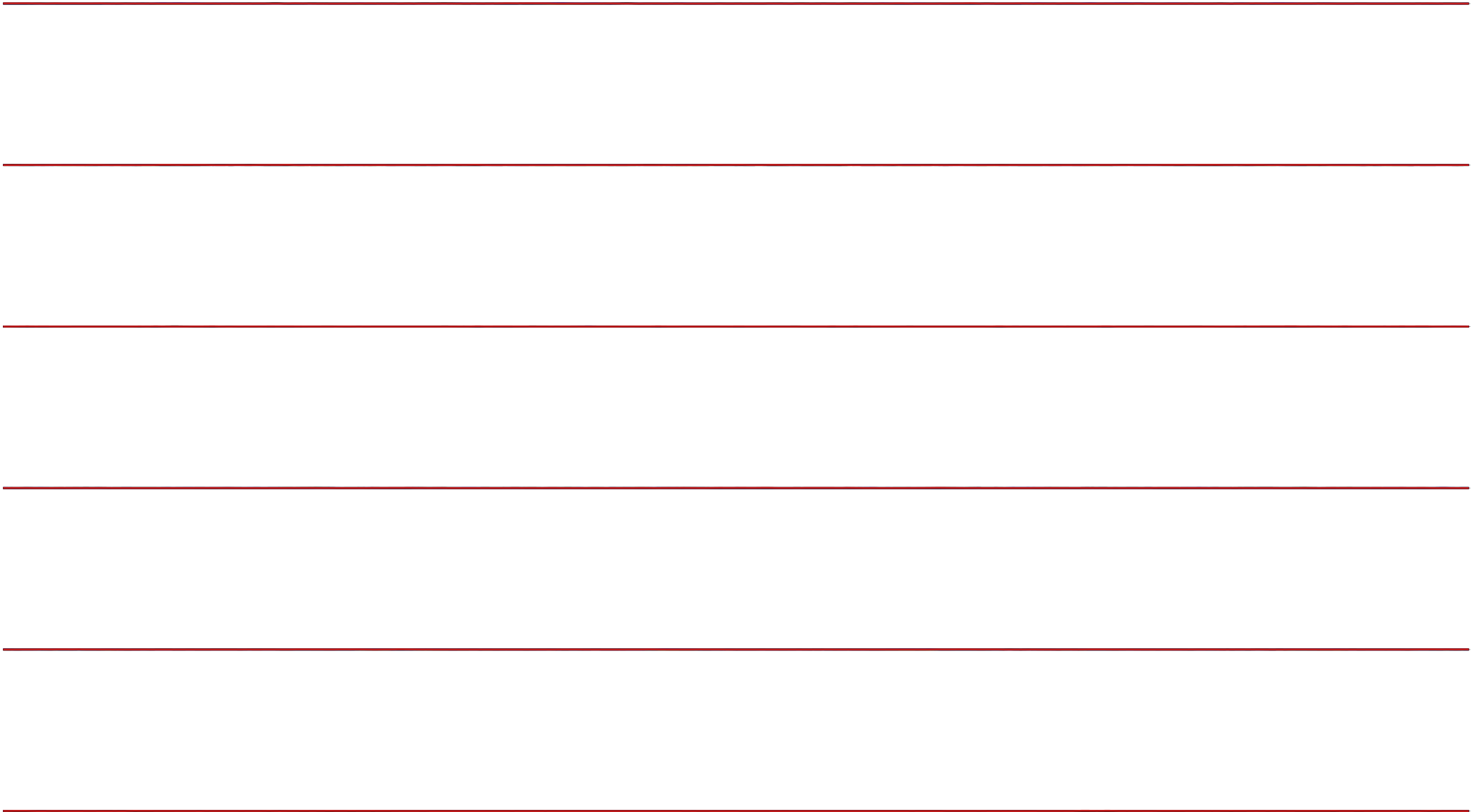

---

---

---

Supplement: Supplementary file 4 — Source data [file 41467_2026_68558_MOESM4_ESM.zip › Source data/Sanger-sequencing data/Fig2c-d/JB late-Meg3.pdf]

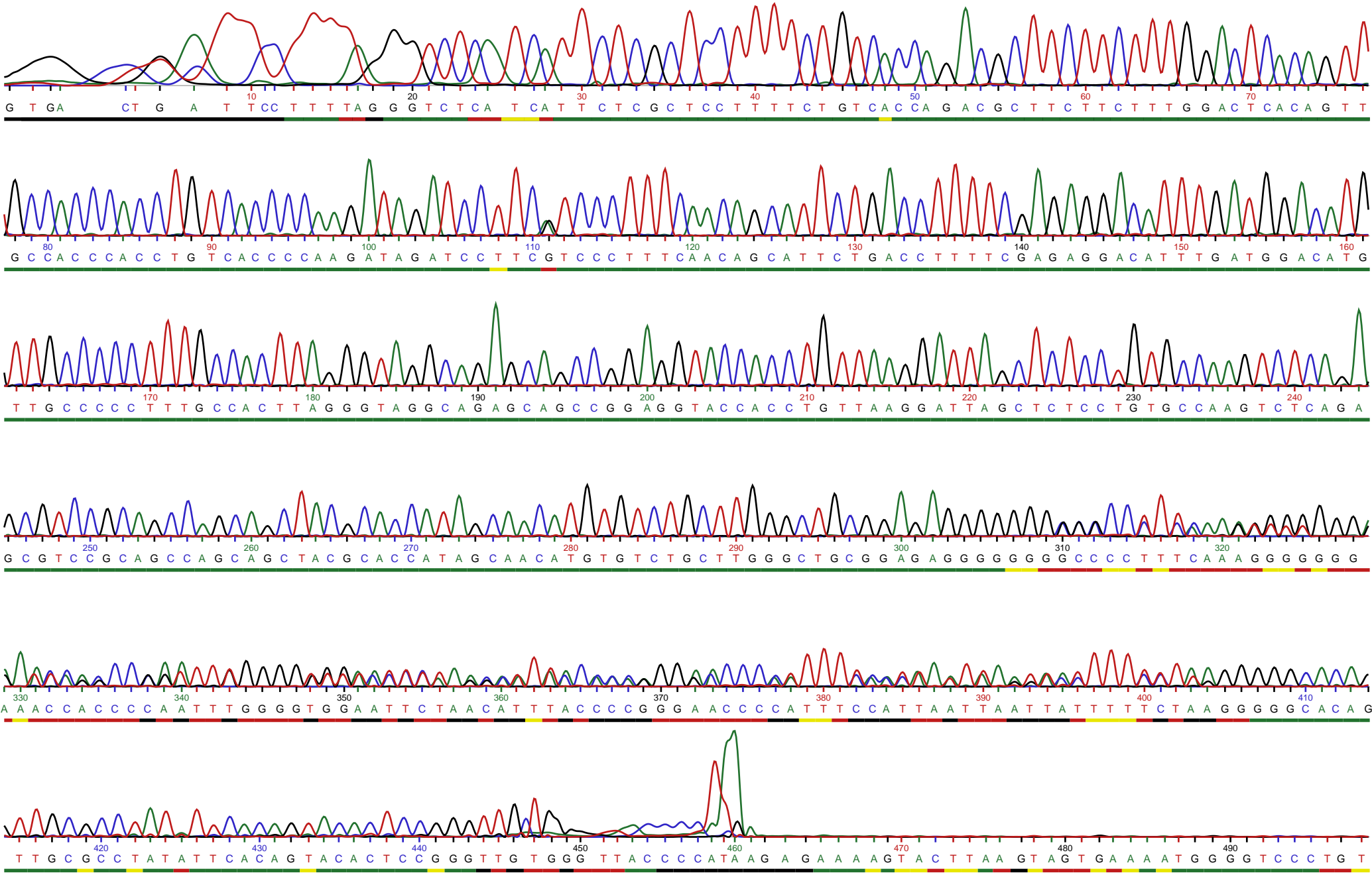

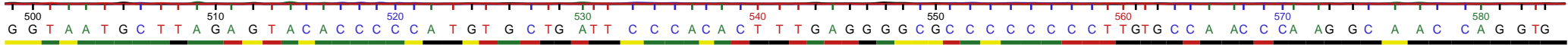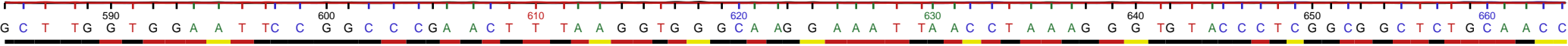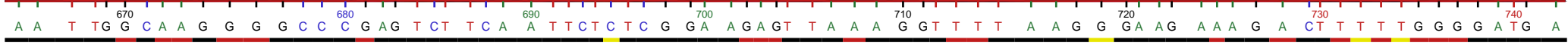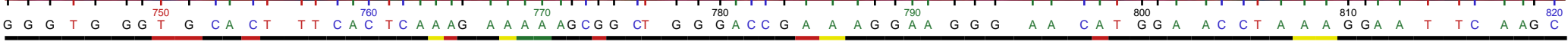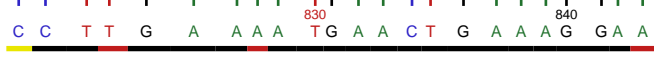

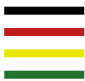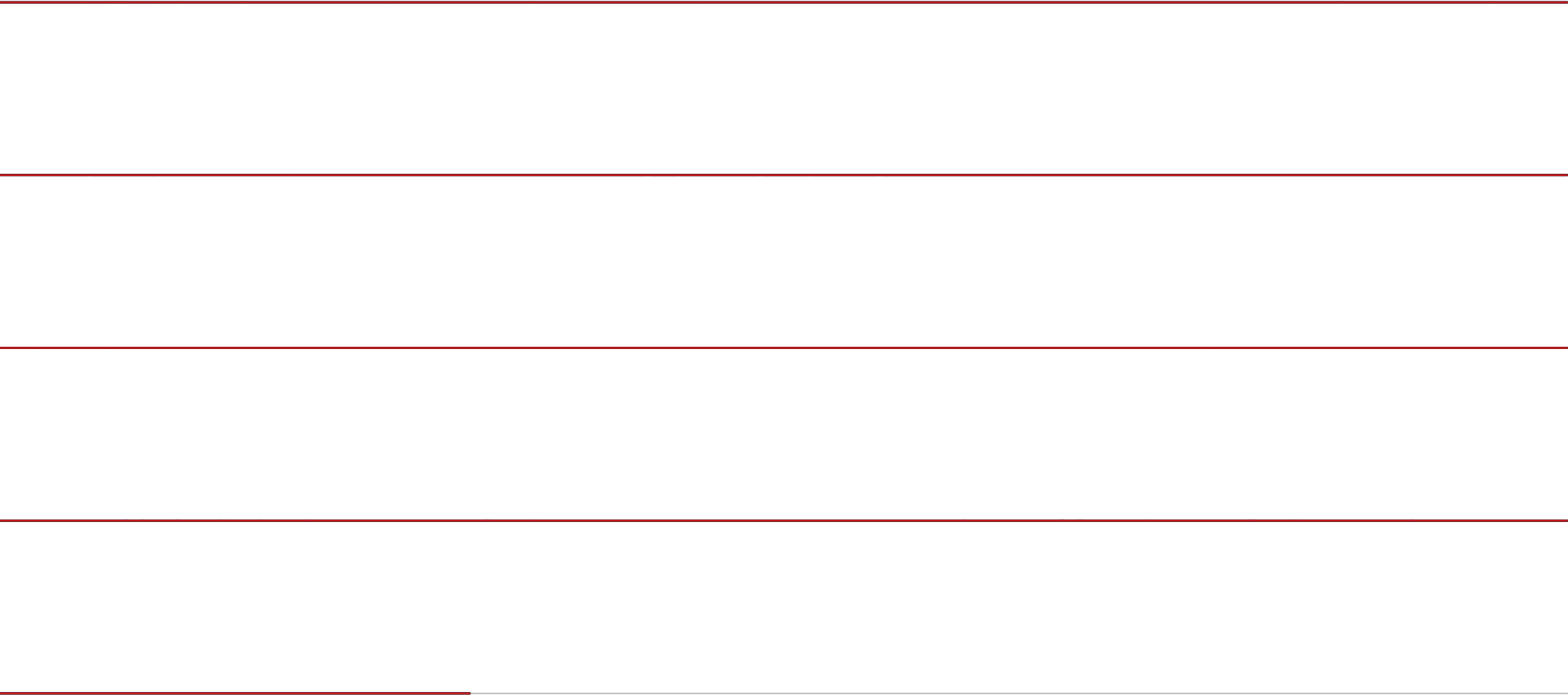

Supplement: Supplementary file 4 — Source data [file 41467_2026_68558_MOESM4_ESM.zip › Source data/Sanger-sequencing data/Fig4d/Meg3DMR -WT-Hpa2-.pdf]

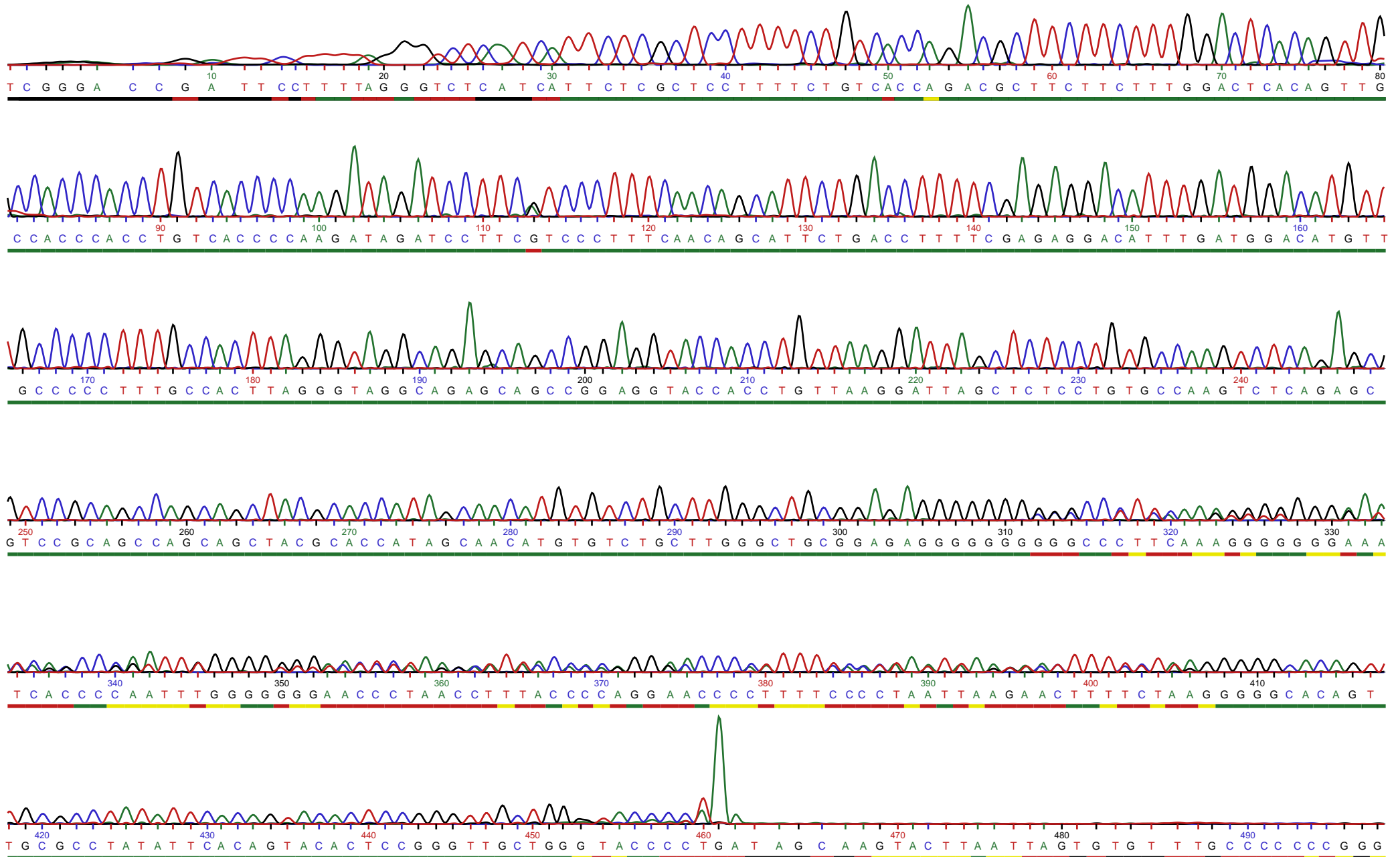

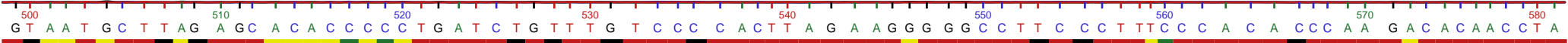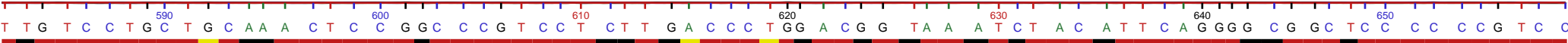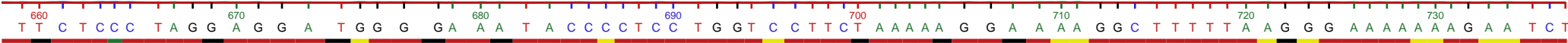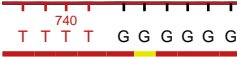

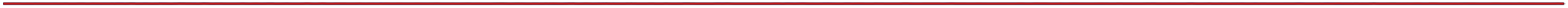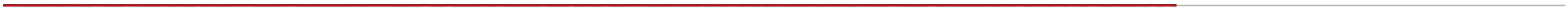

Supplement: Supplementary file 4 — Source data [file 41467_2026_68558_MOESM4_ESM.zip › Source data/Sanger-sequencing data/Fig4d/Meg3DMR-Zfp57KO-Meg3pro-Hpa2+.pdf]

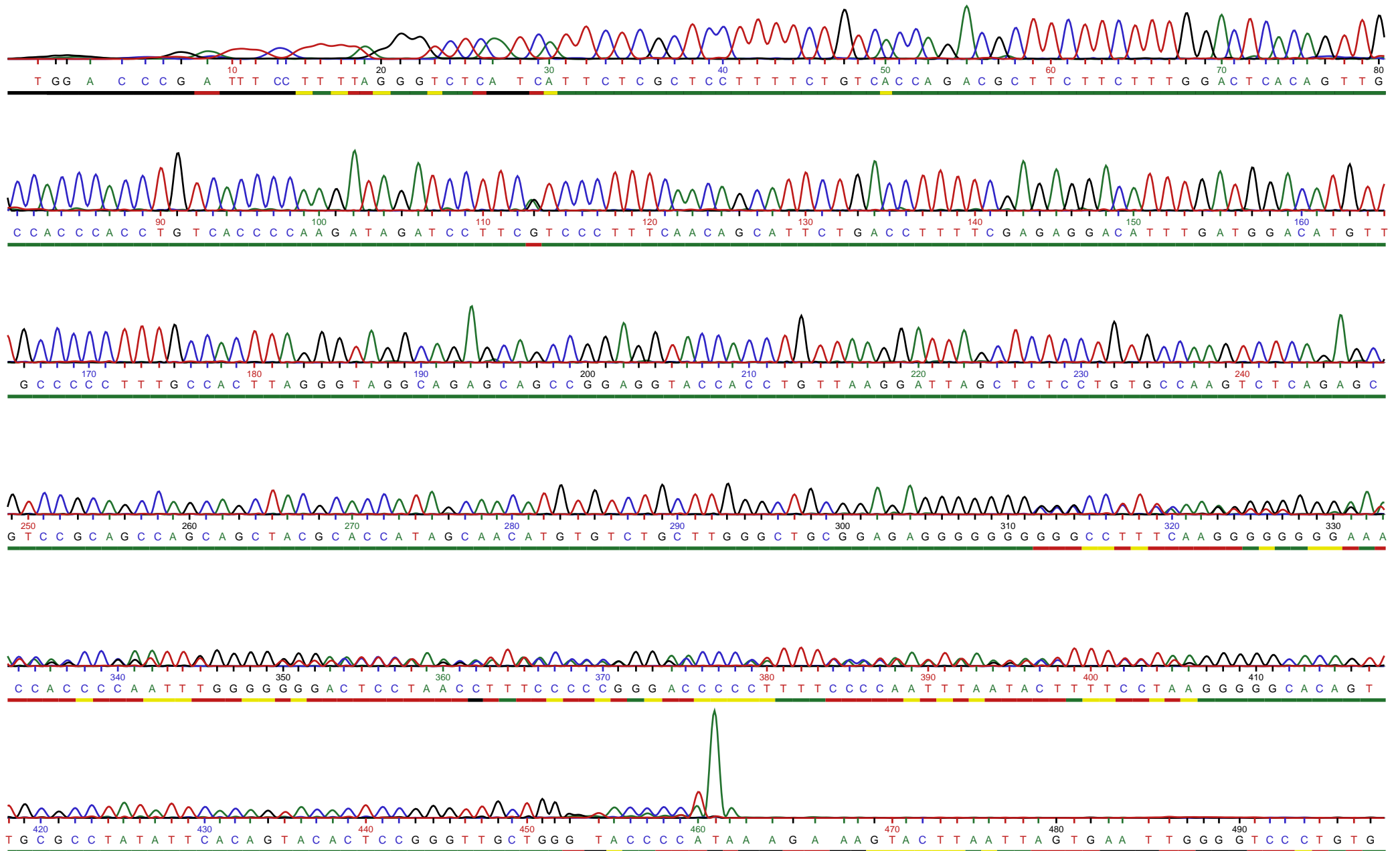

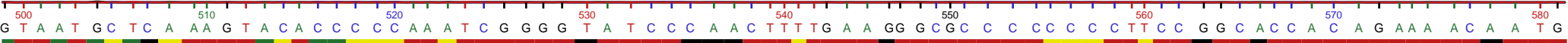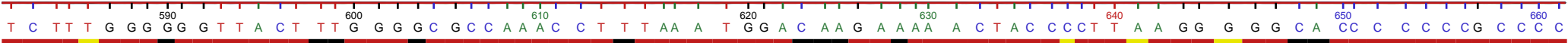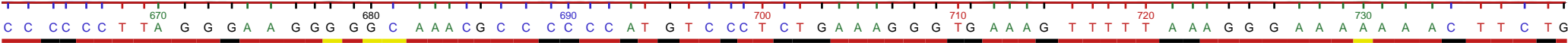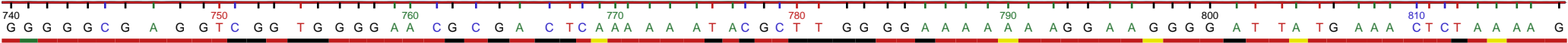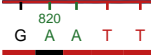

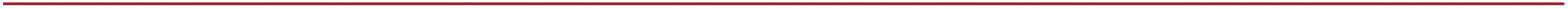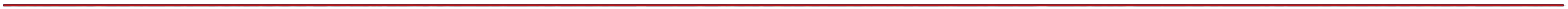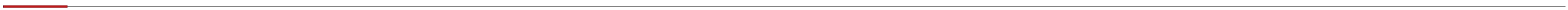

Supplement: Supplementary file 4 — Source data [file 41467_2026_68558_MOESM4_ESM.zip › Source data/Sanger-sequencing data/Fig7d/NPC-Meg3DMR-Hpa2-.pdf]

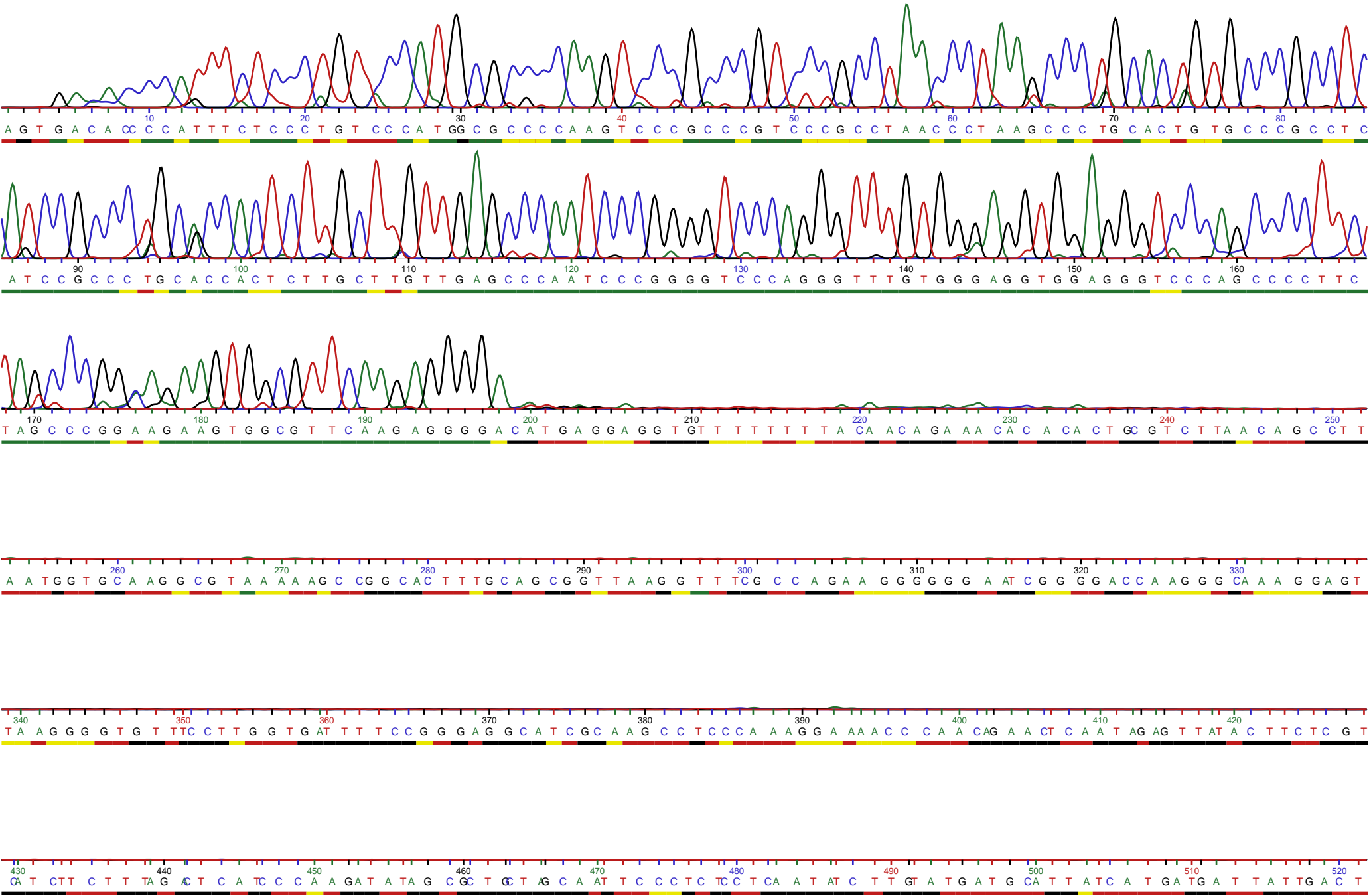

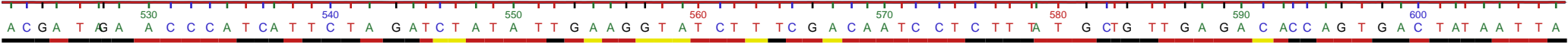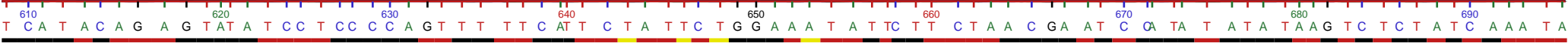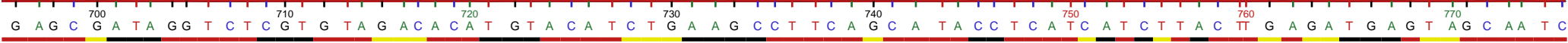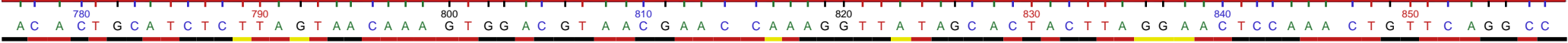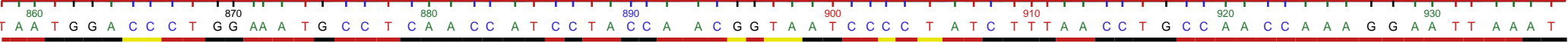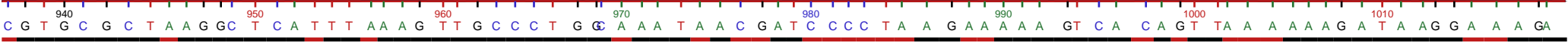

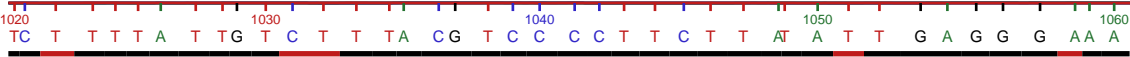

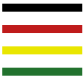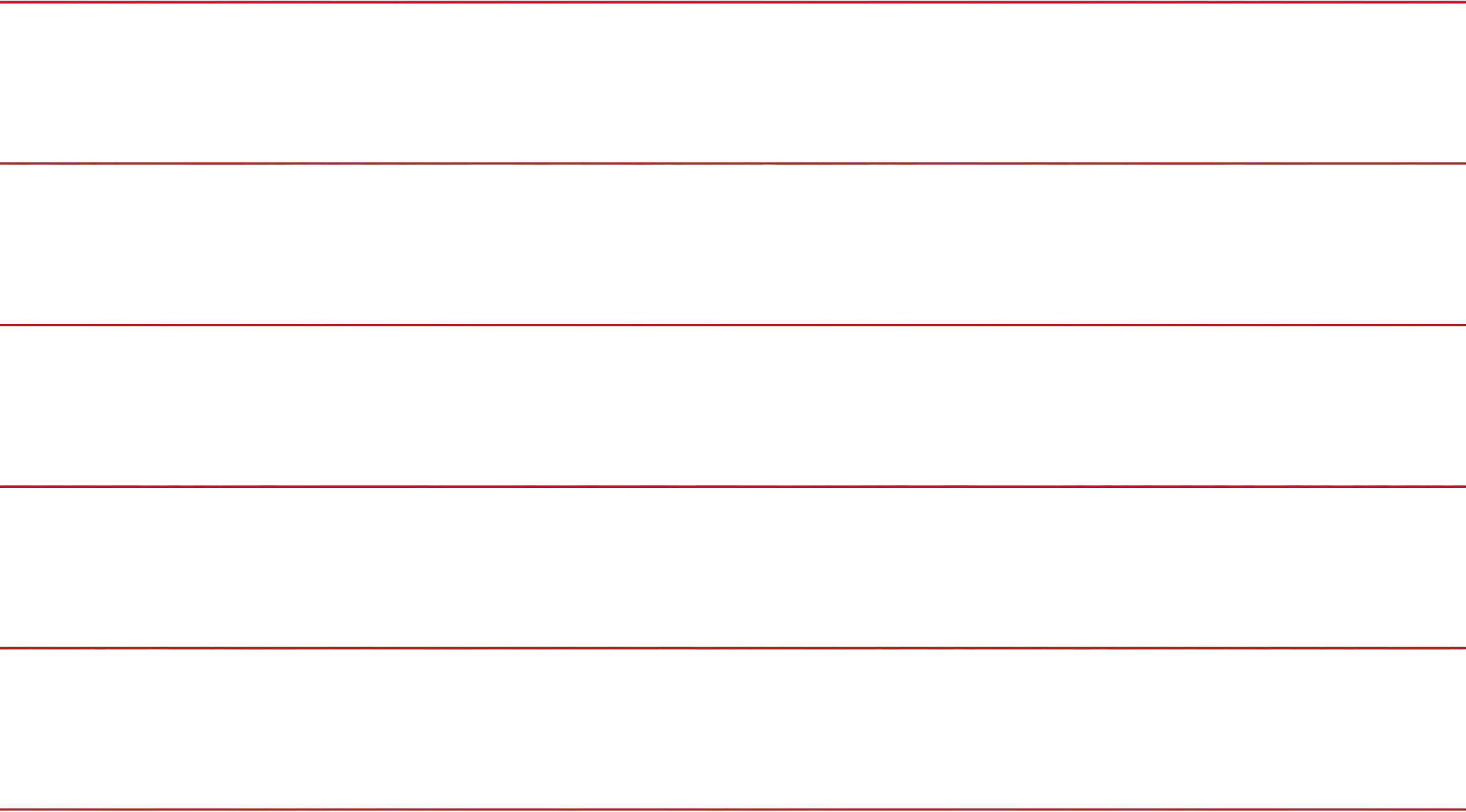

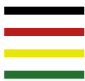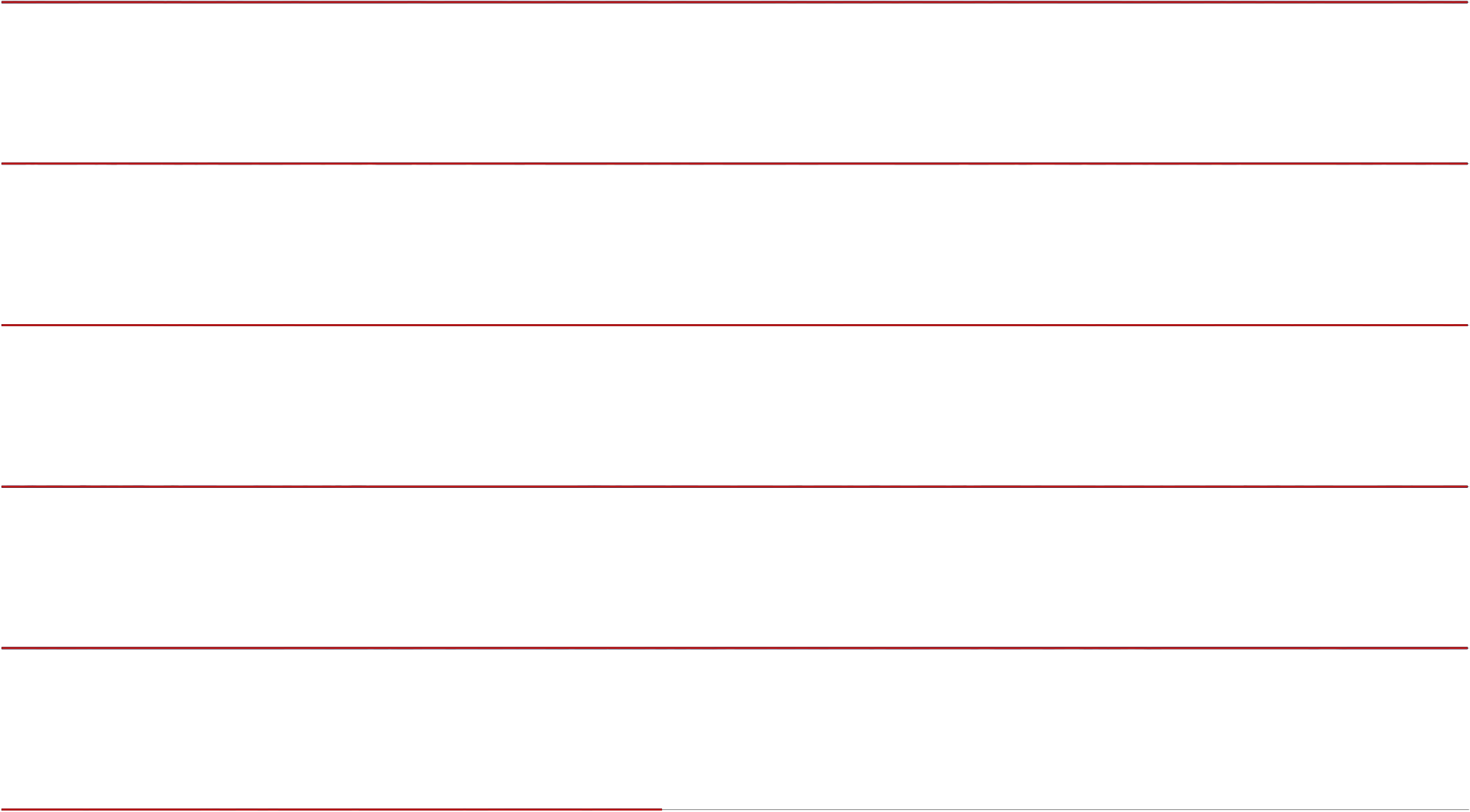

Supplement: Supplementary file 4 — Source data [file 41467_2026_68558_MOESM4_ESM.zip › Source data/Sanger-sequencing data/Fig7h/NPC-early-Dlk1.pdf]

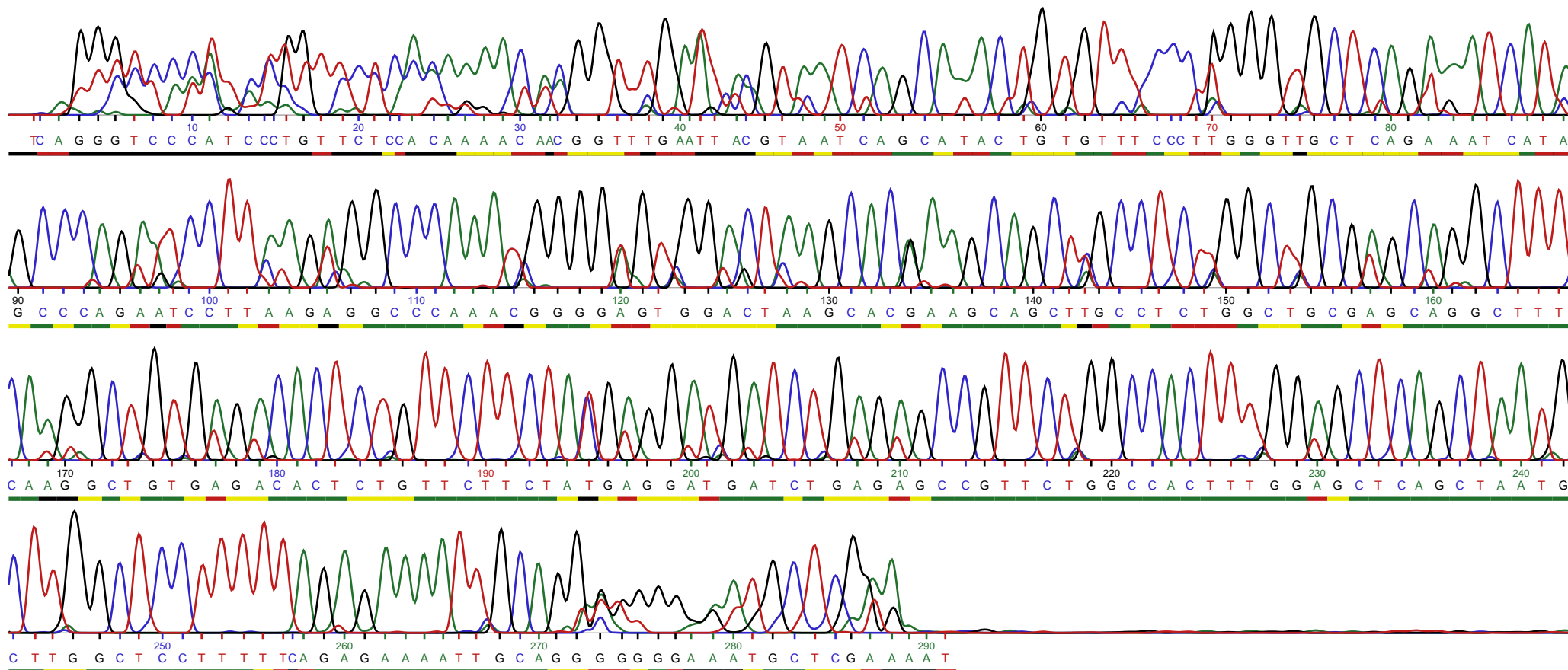

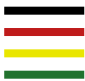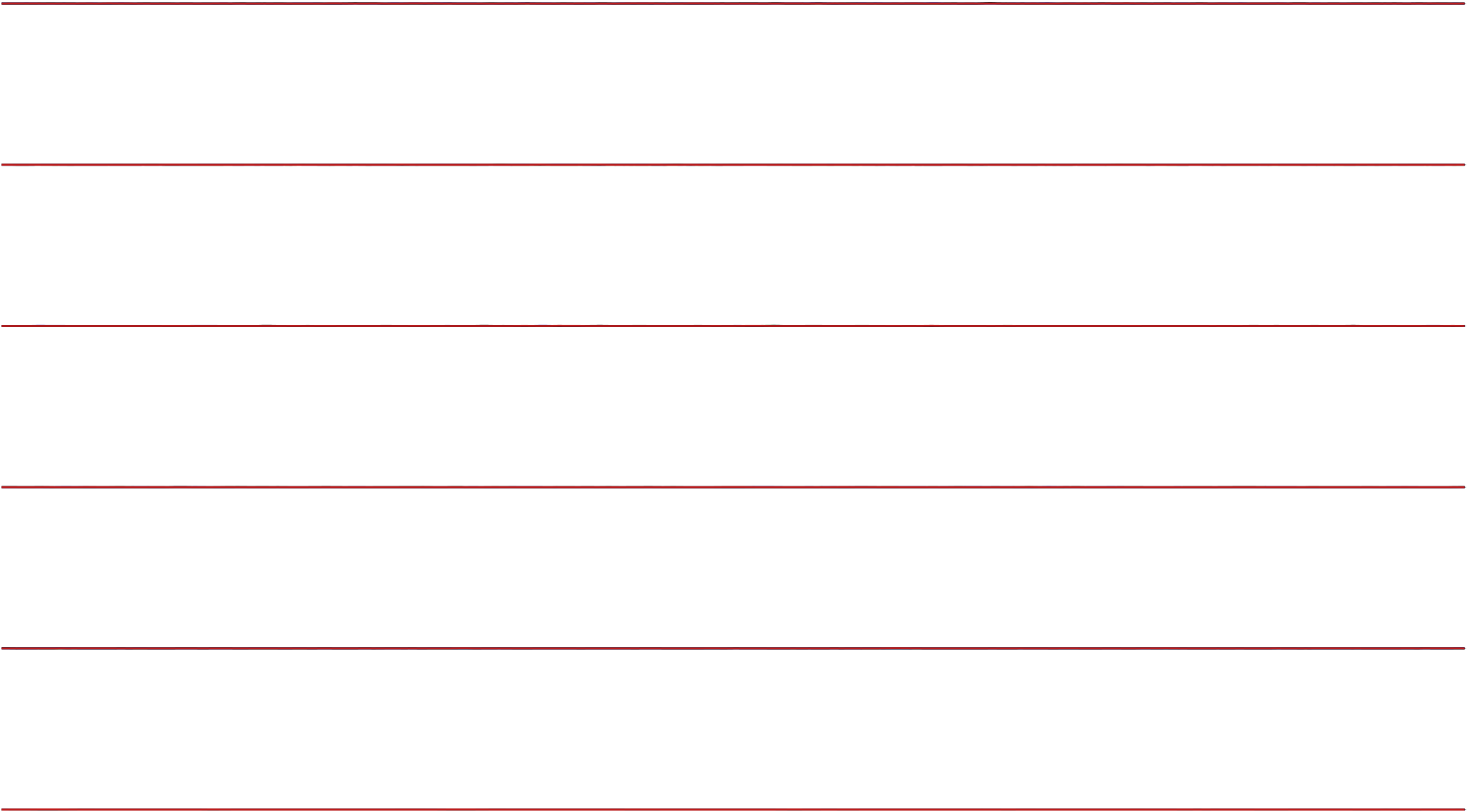

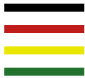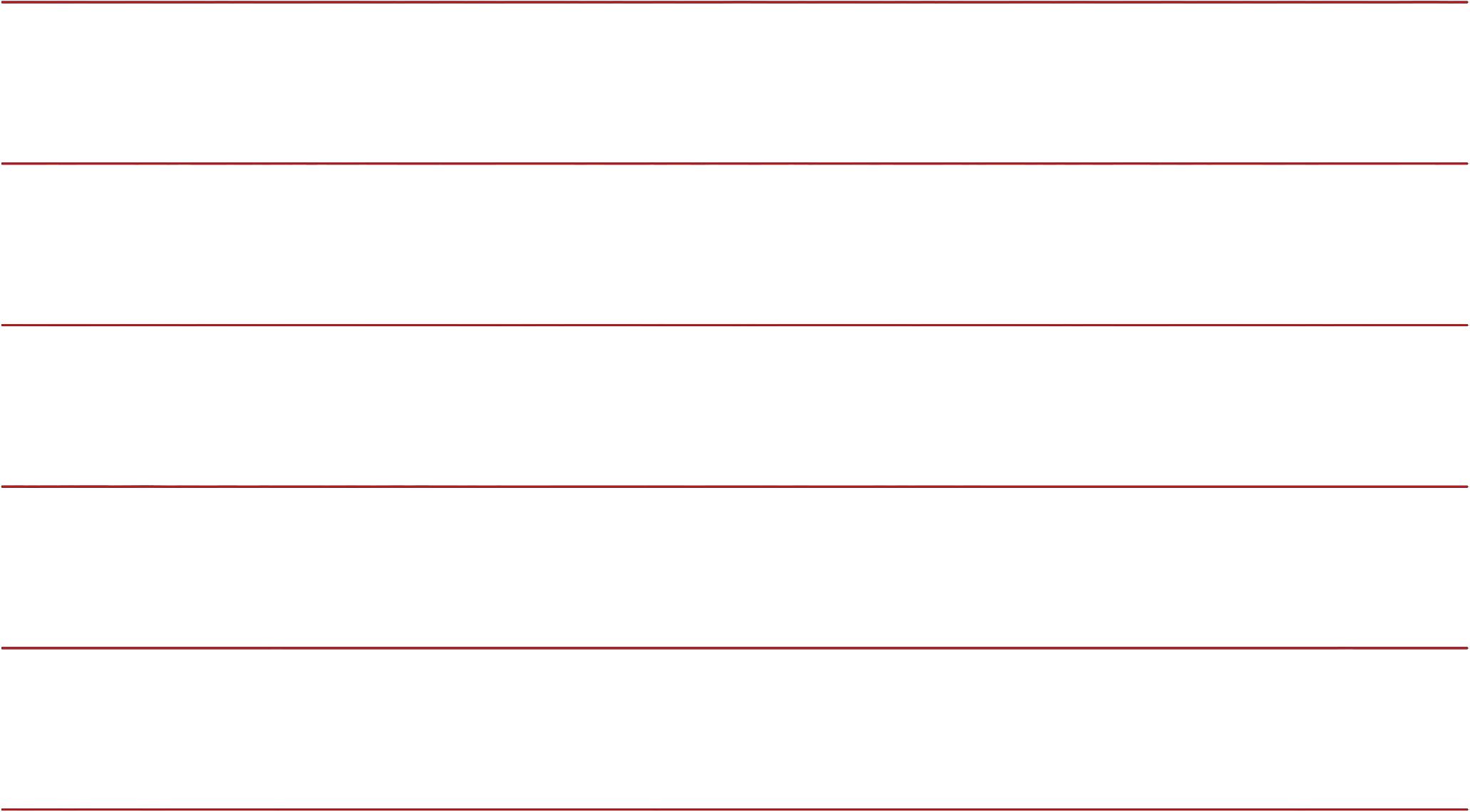

---

---

---

Supplement: Supplementary file 4 — Source data [file 41467_2026_68558_MOESM4_ESM.zip › Source data/Sanger-sequencing data/Fig7h/NPC-early-Meg3.pdf]

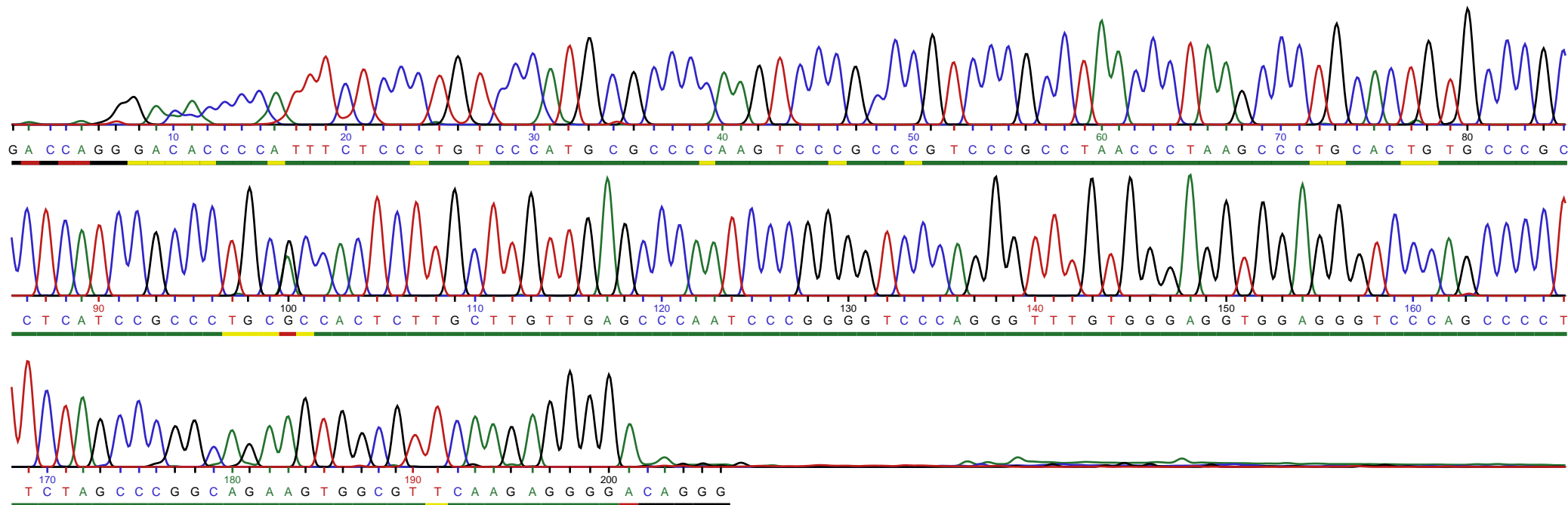

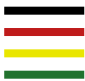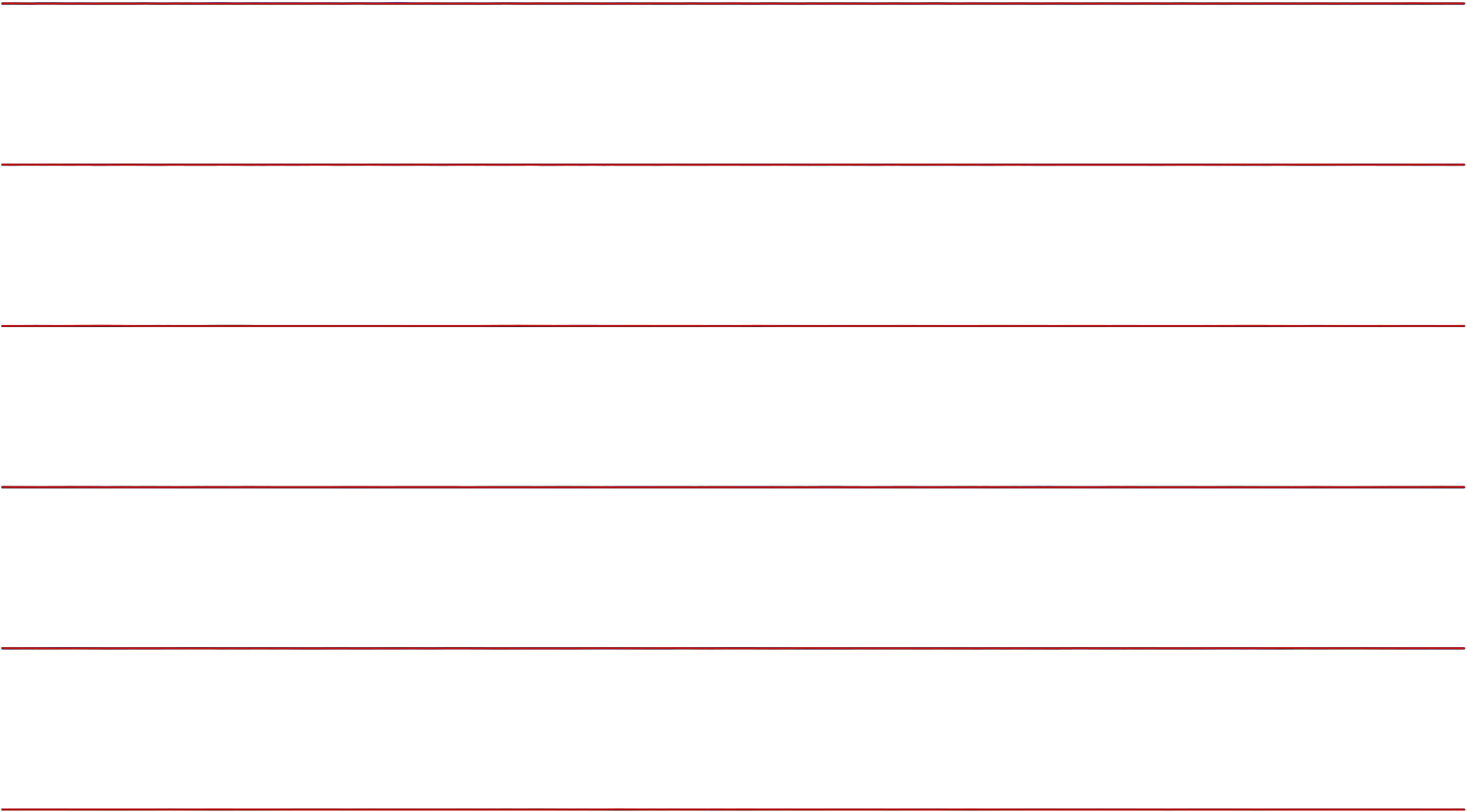

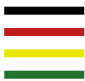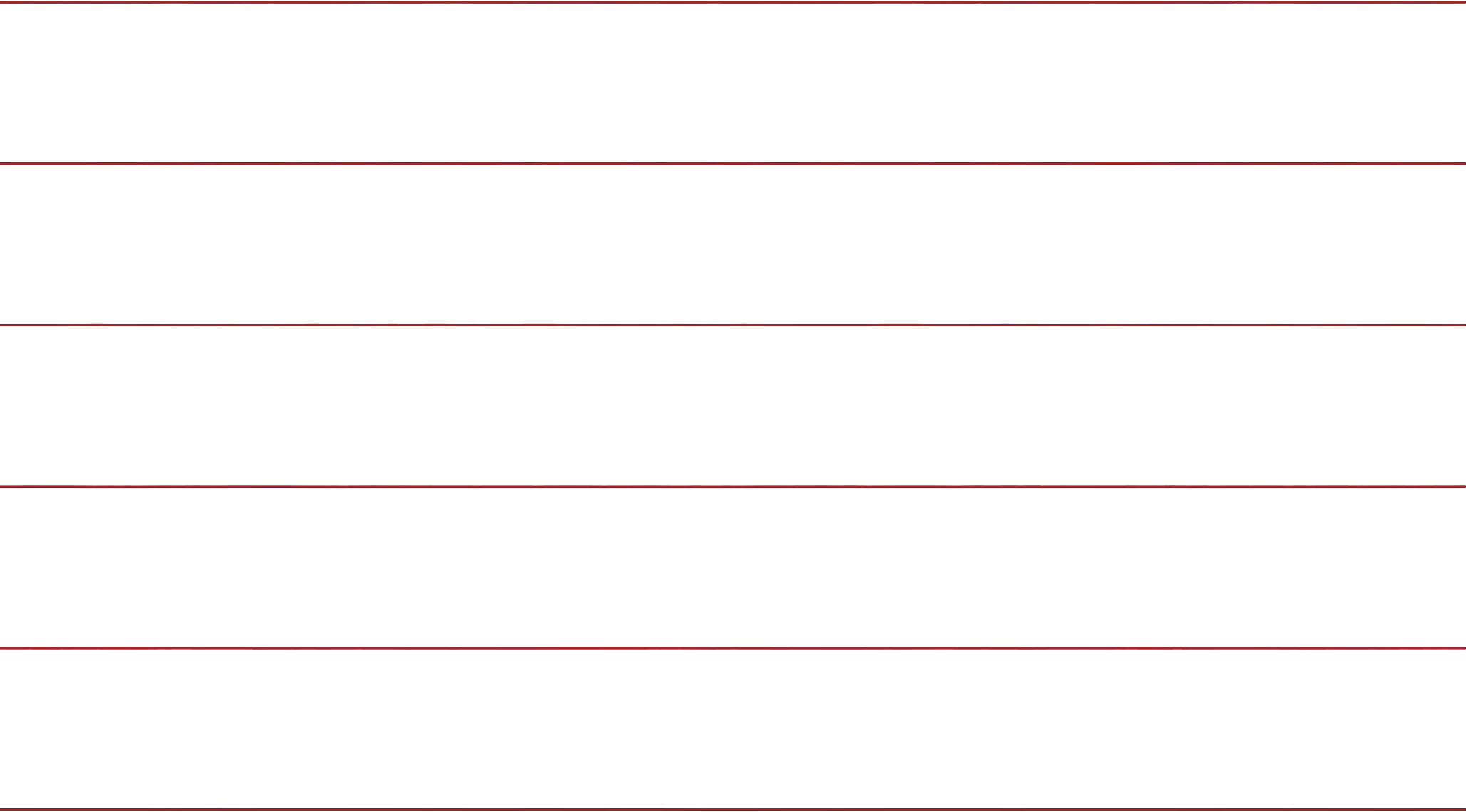

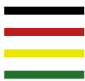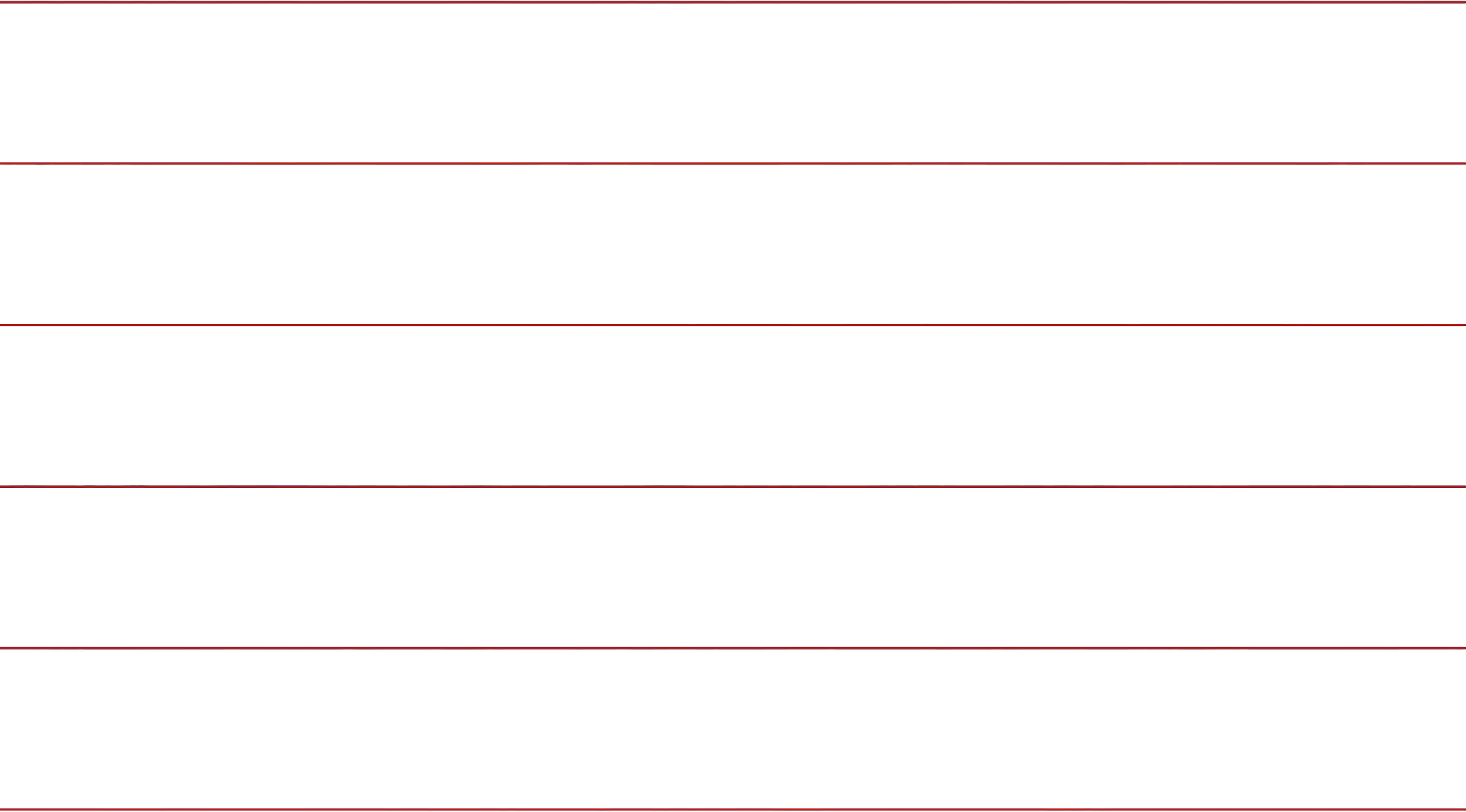

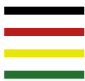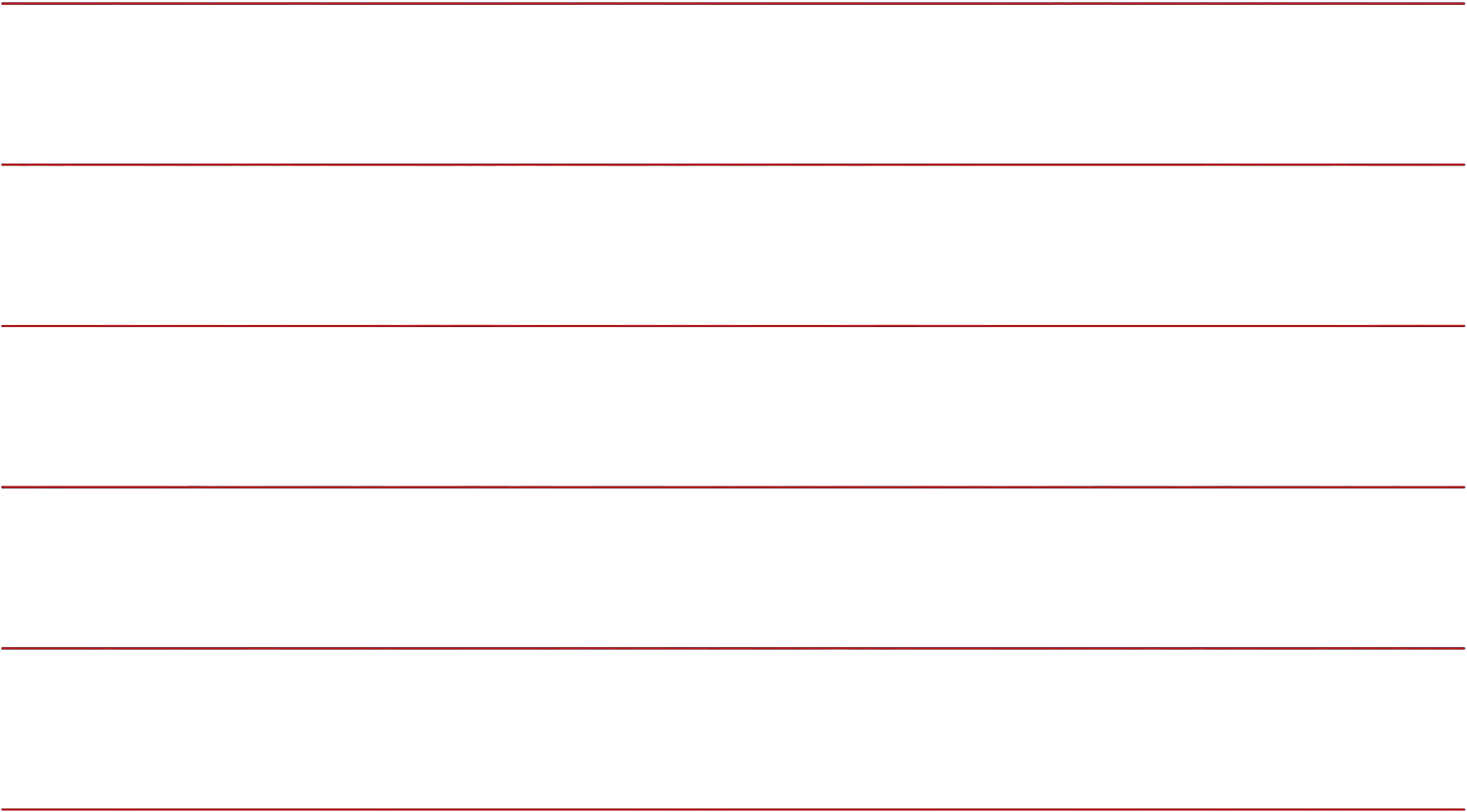

---

---

---

Supplement: Supplementary file 4 — Source data [file 41467_2026_68558_MOESM4_ESM.zip › Source data/Sanger-sequencing data/Fig7h/NPC-late-Dlk1.pdf]

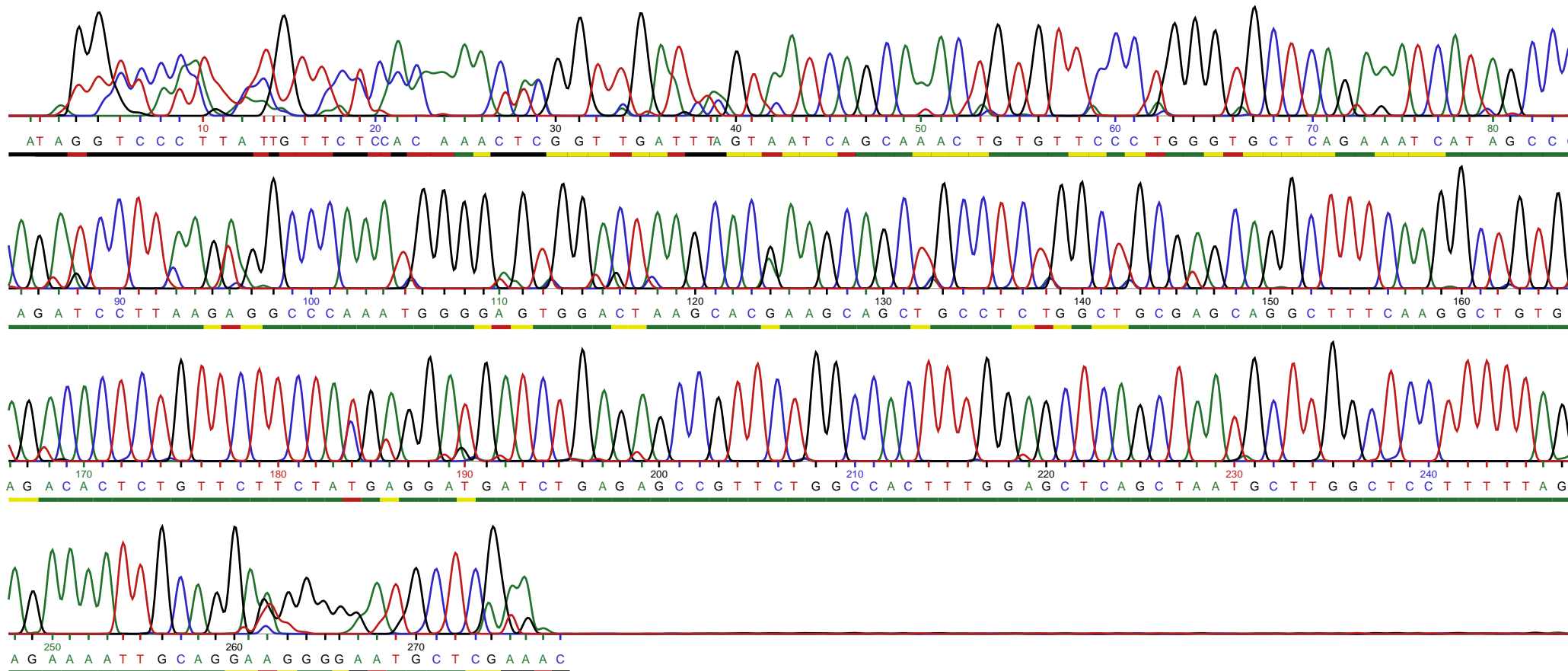

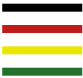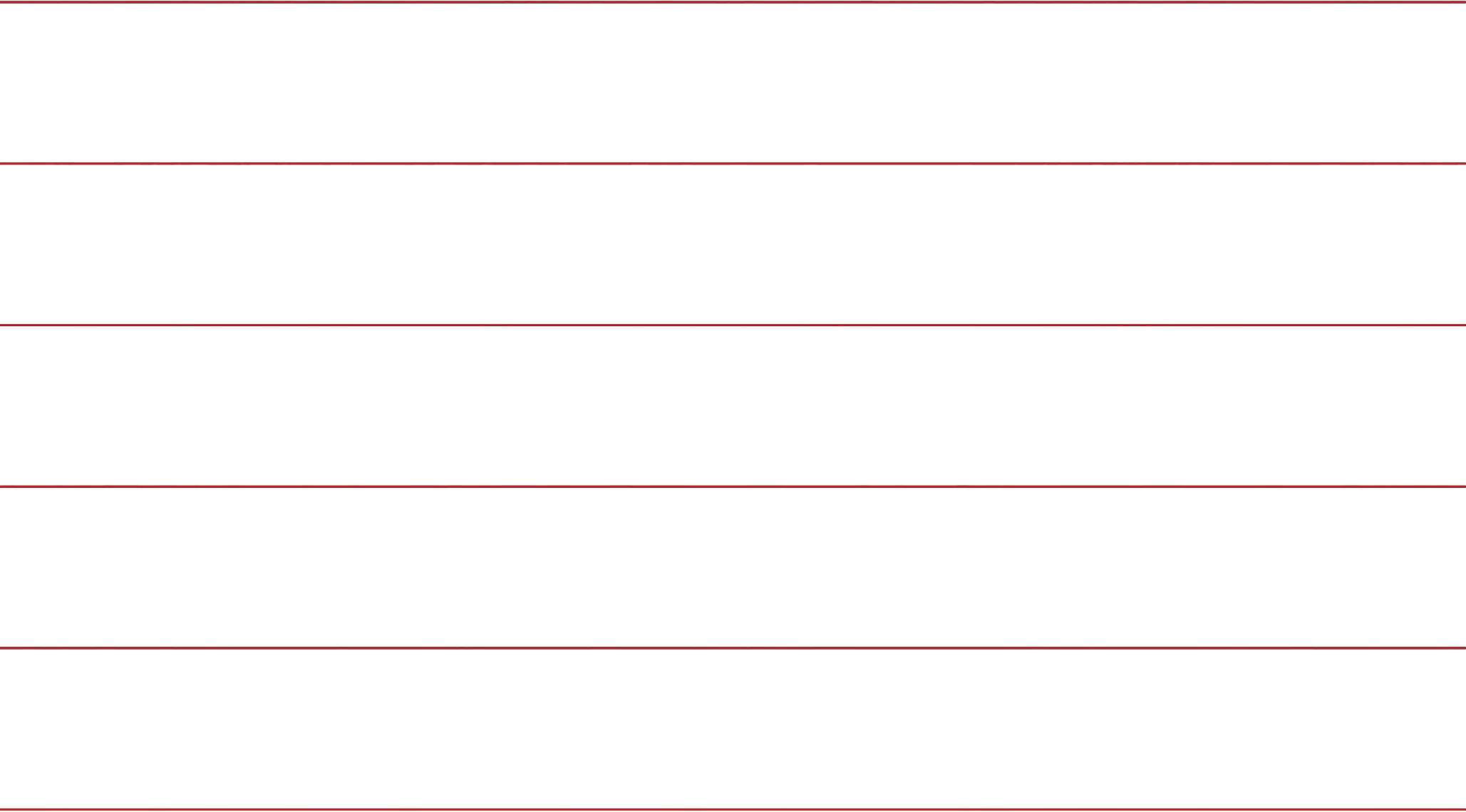

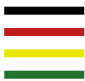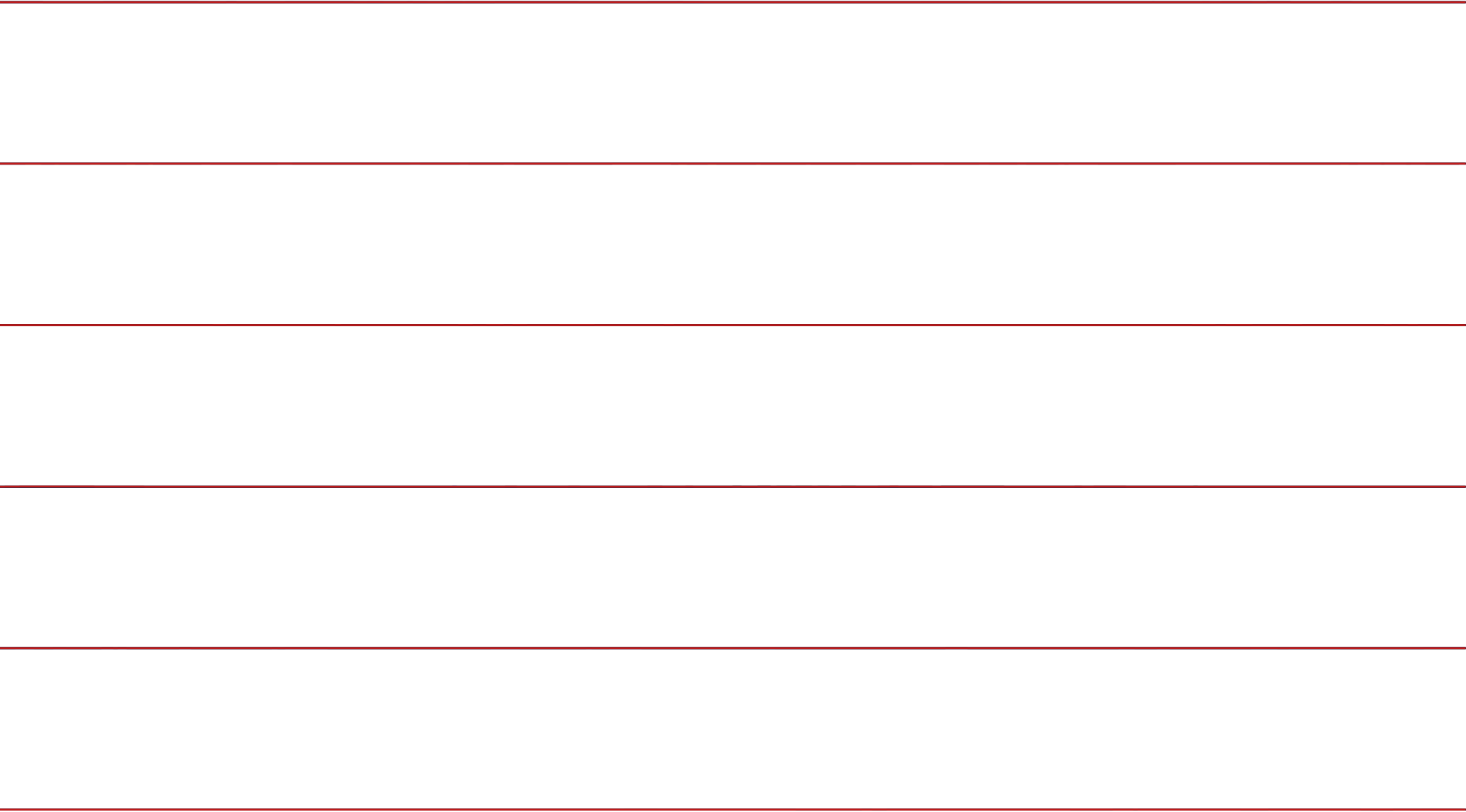

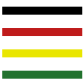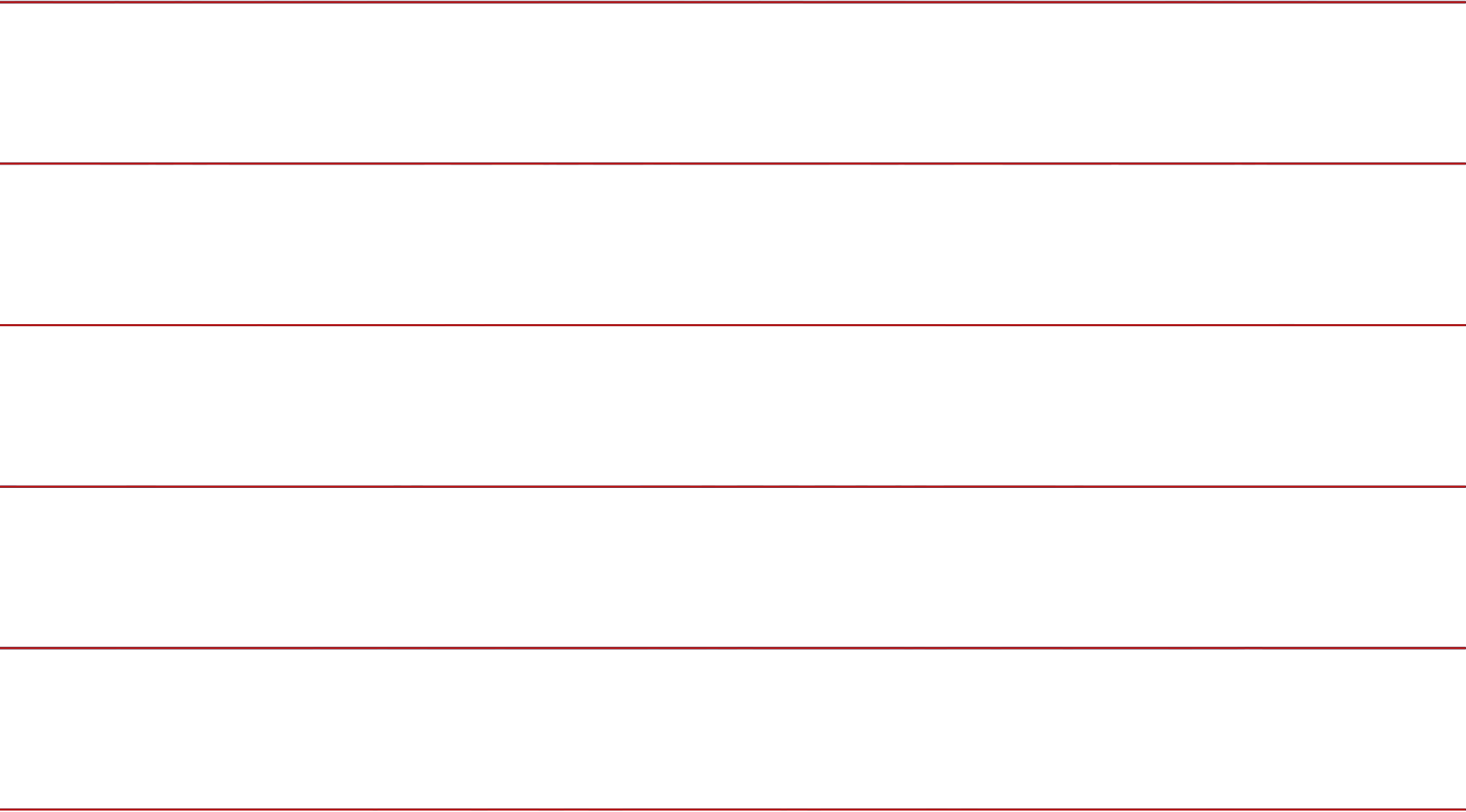

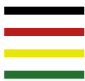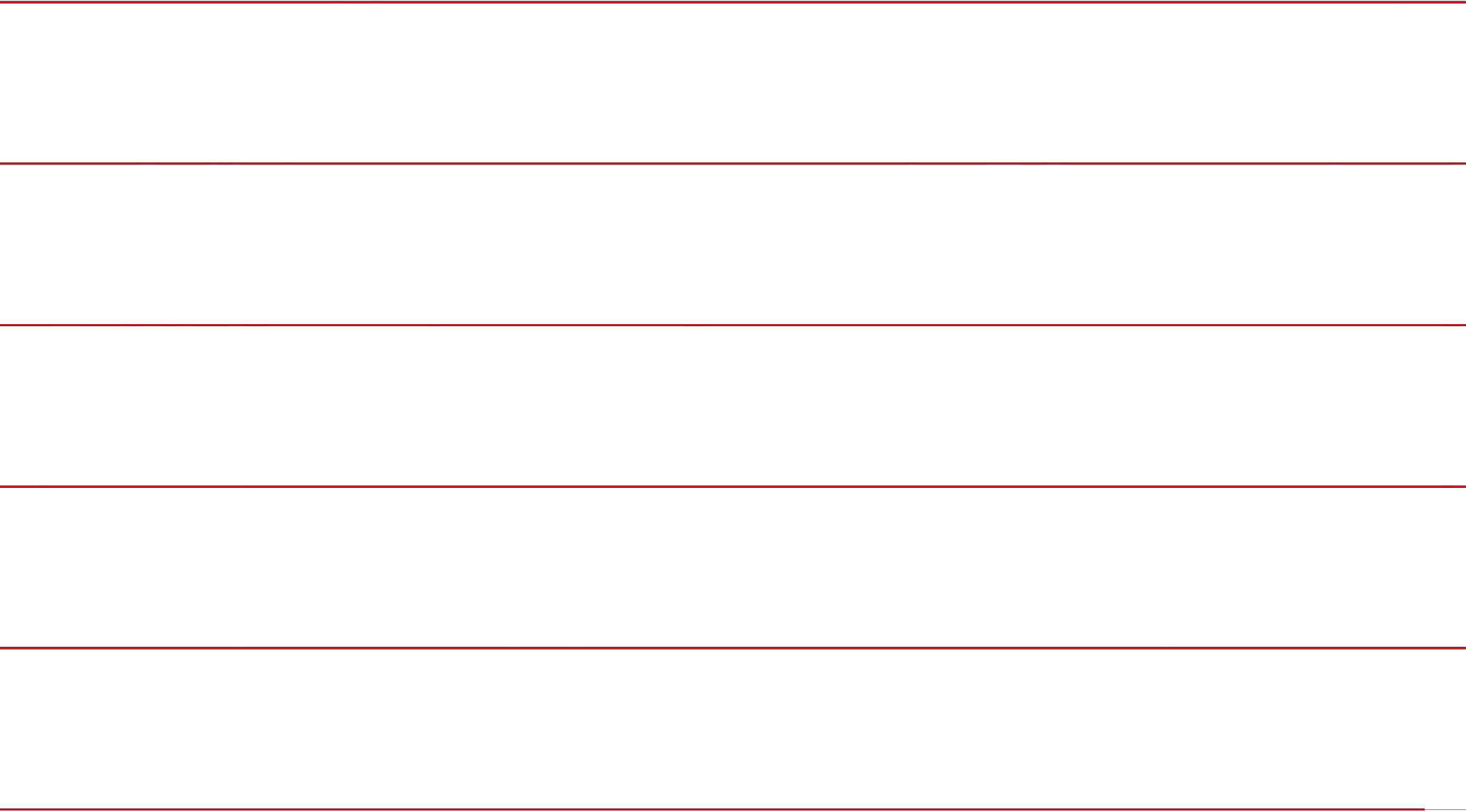

Supplement: Supplementary file 4 — Source data [file 41467_2026_68558_MOESM4_ESM.zip › Source data/Sanger-sequencing data/Fig7h/NPC-late-Meg3.pdf]

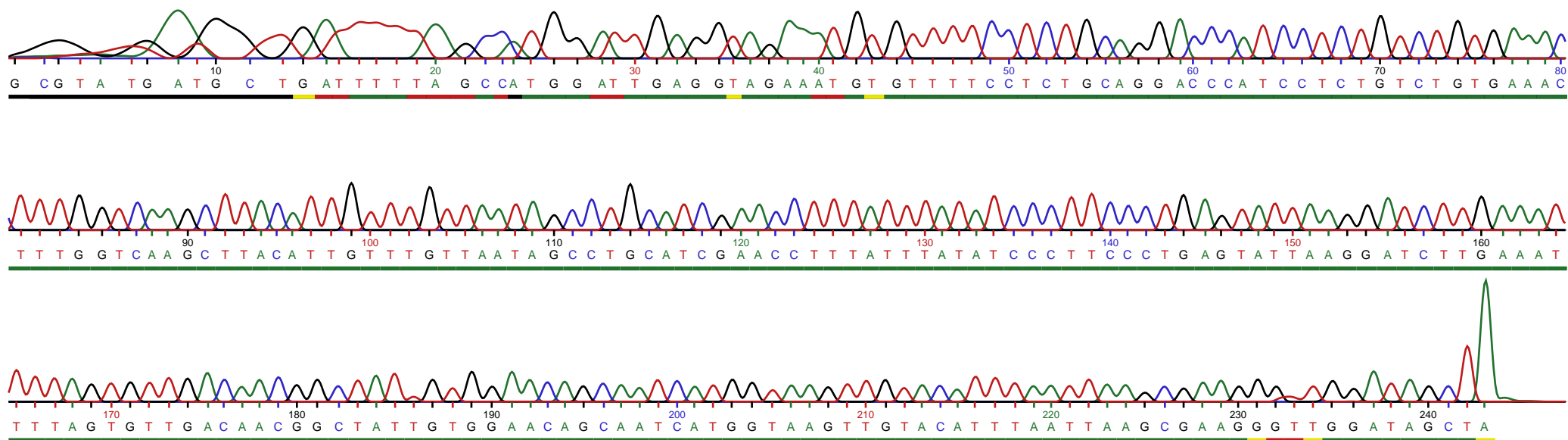

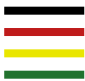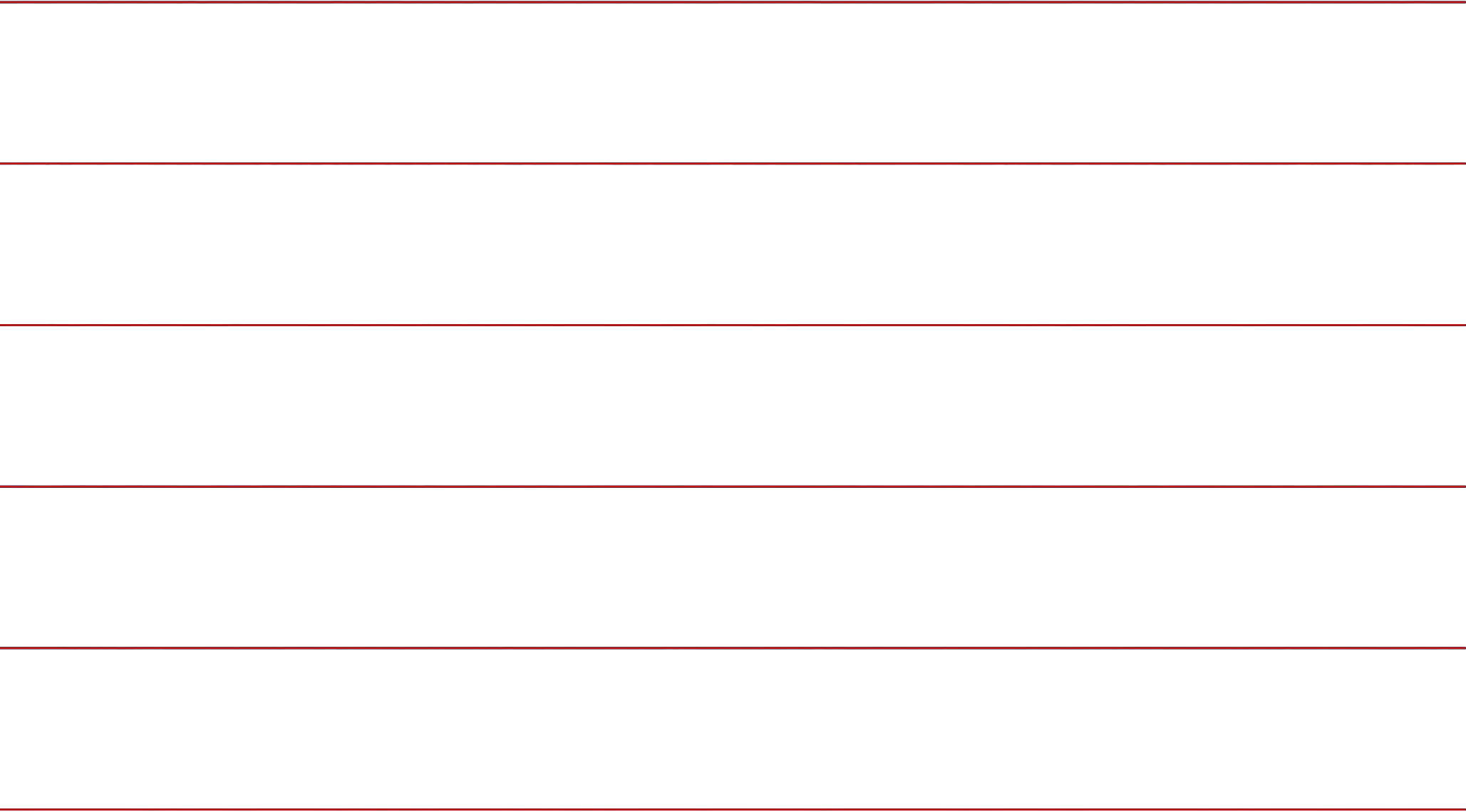

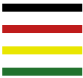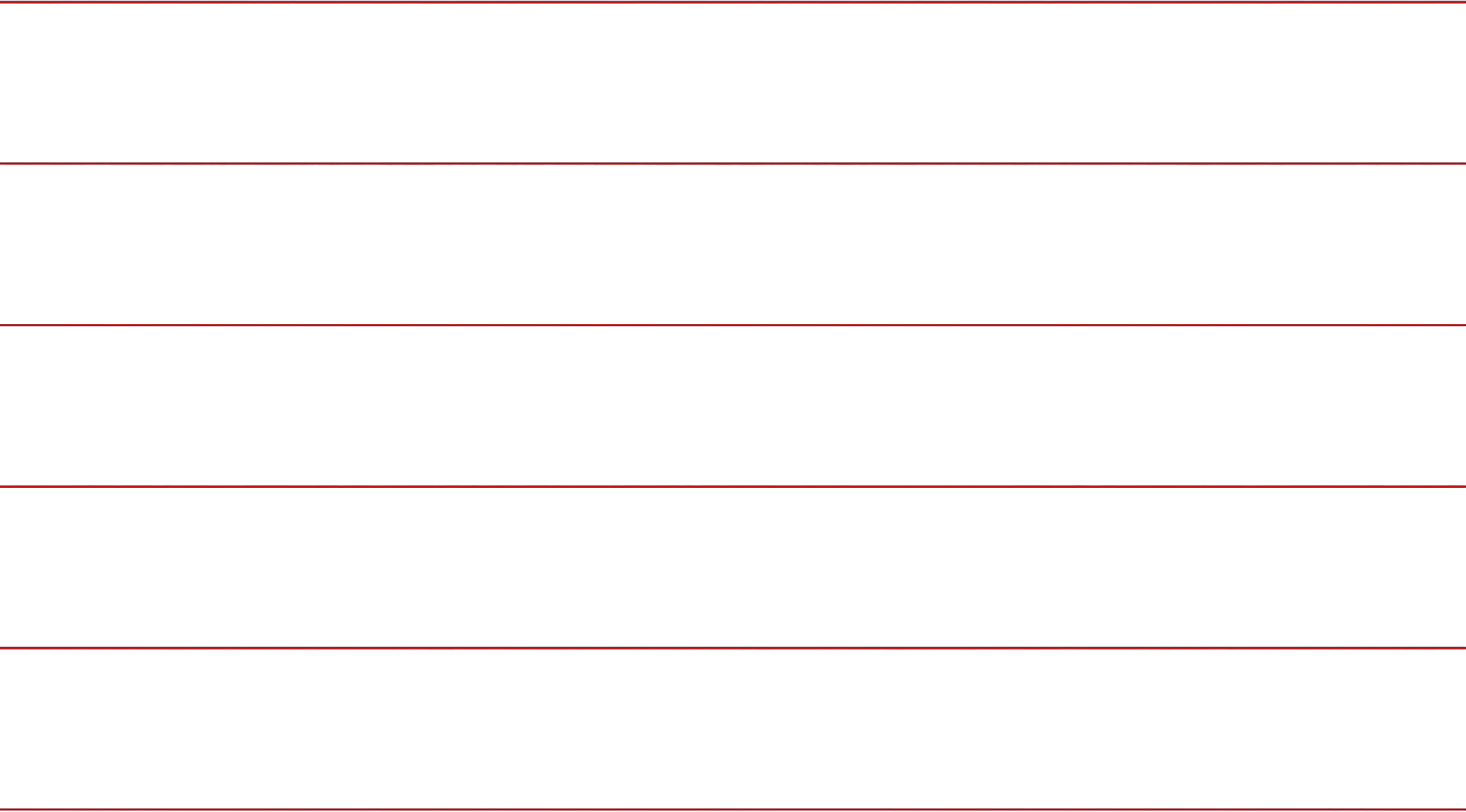

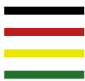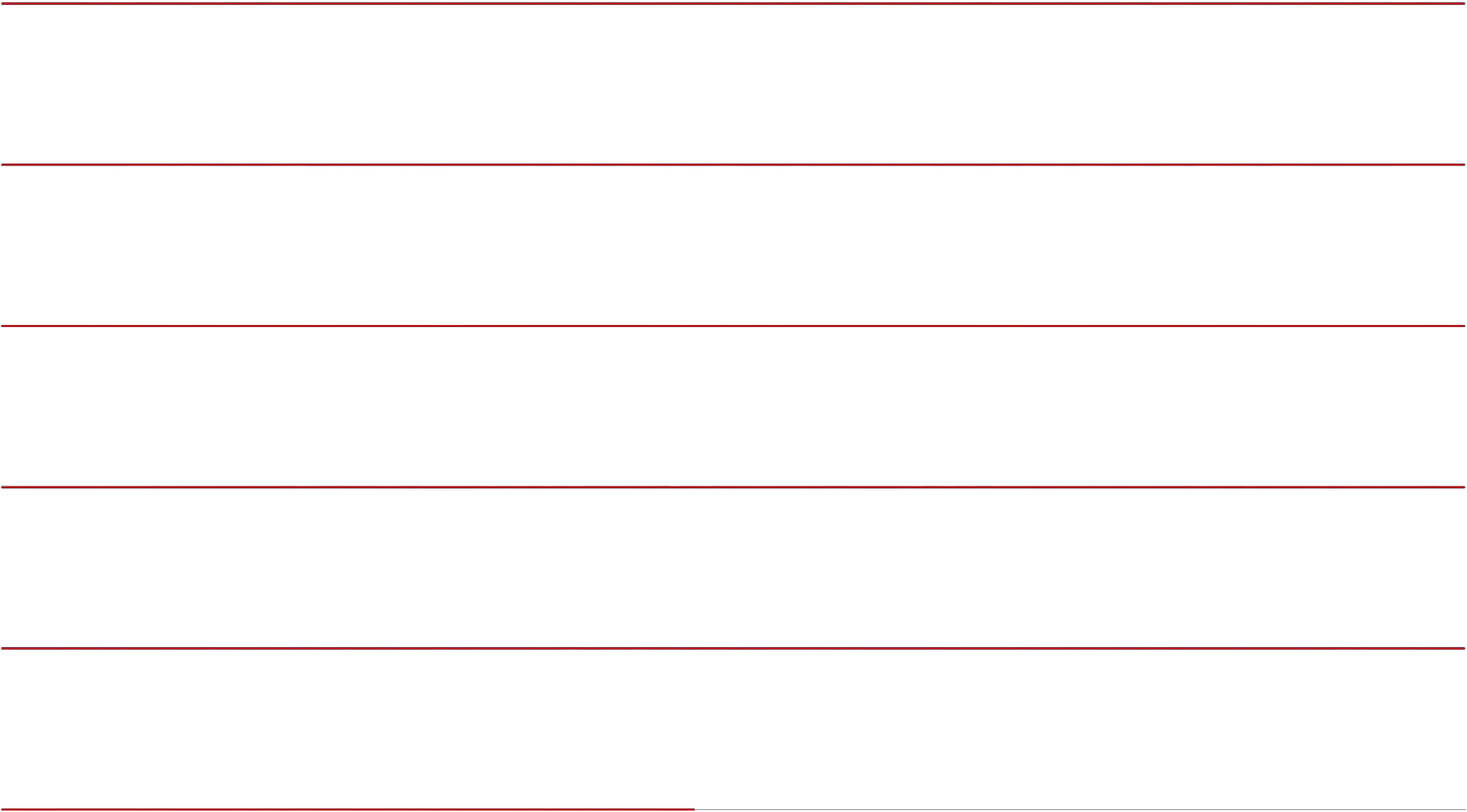

Supplement: Supplementary file 4 — Source data [file 41467_2026_68558_MOESM4_ESM.zip › Source data/Sanger-sequencing data/Suppl.Fig1f/BJ early SnrpnR1.pdf]

Samples: 21108  
Bases: 1371  
Average spacing: 16.0  
Average quality >= 10: 575, 20: 296, 30: 151

Quality: 0 - 9  
10 - 19  
20 - 29  
>= 30

Page: 1 / 4  
27.05.2024

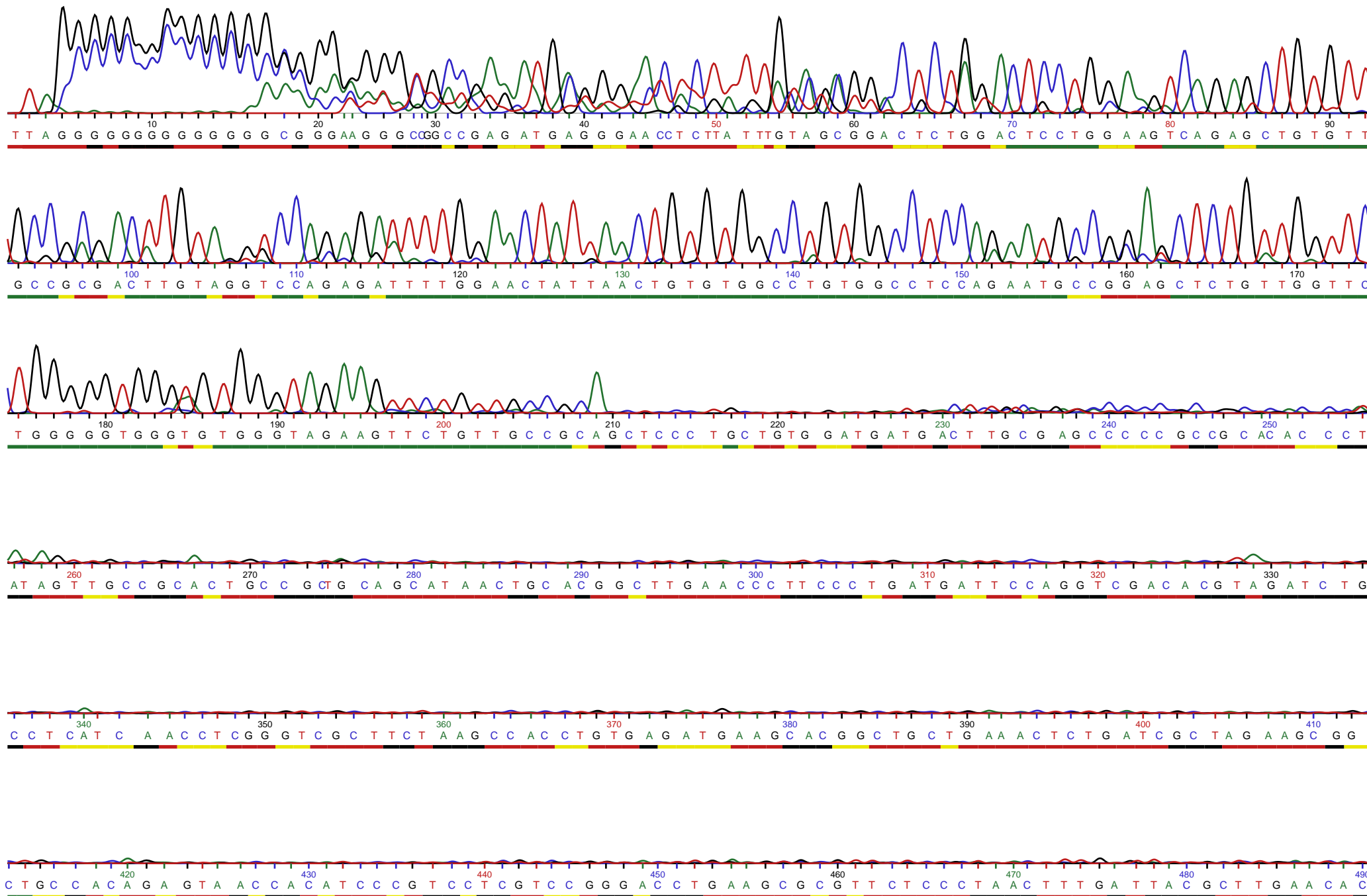

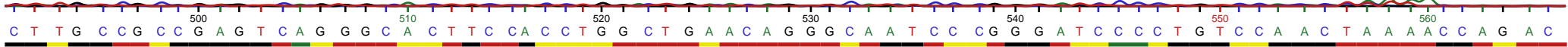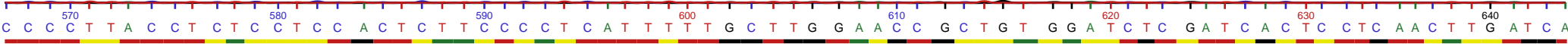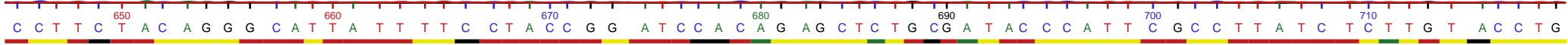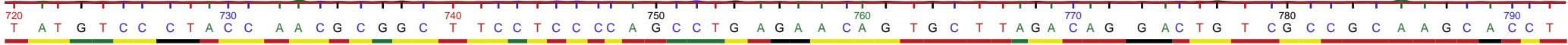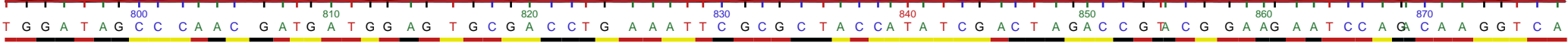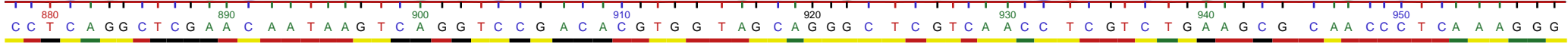

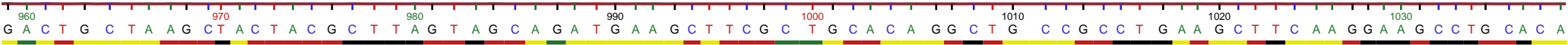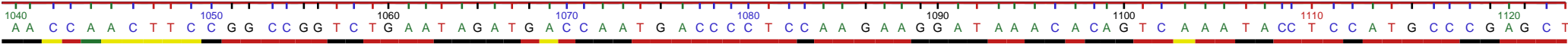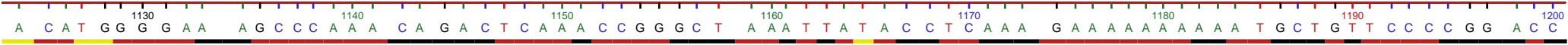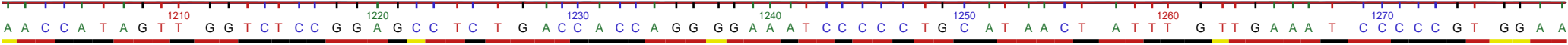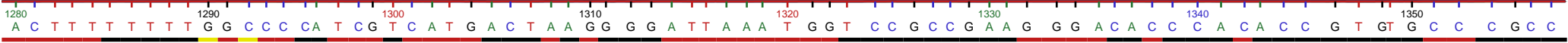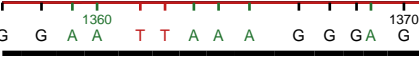

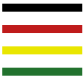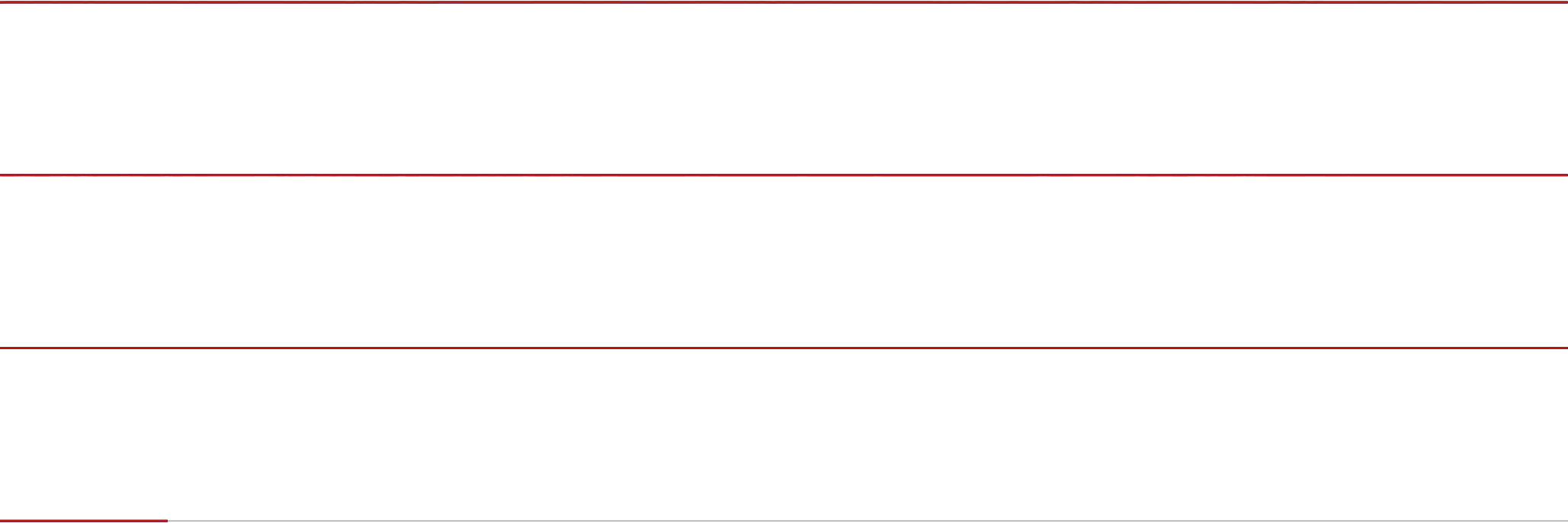

Supplement: Supplementary file 4 — Source data [file 41467_2026_68558_MOESM4_ESM.zip › Source data/Sanger-sequencing data/Suppl.Fig1f/BJ early SnrpnR2.pdf]

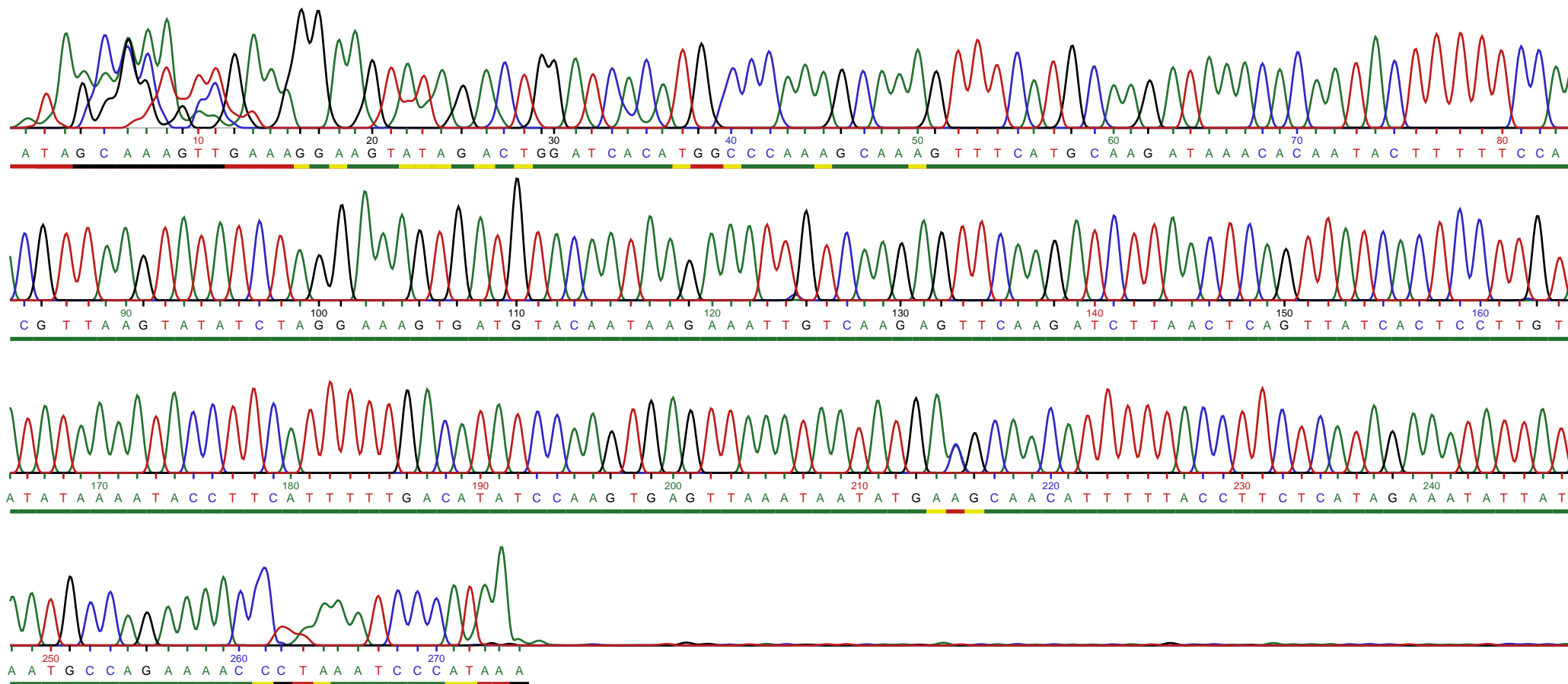

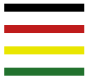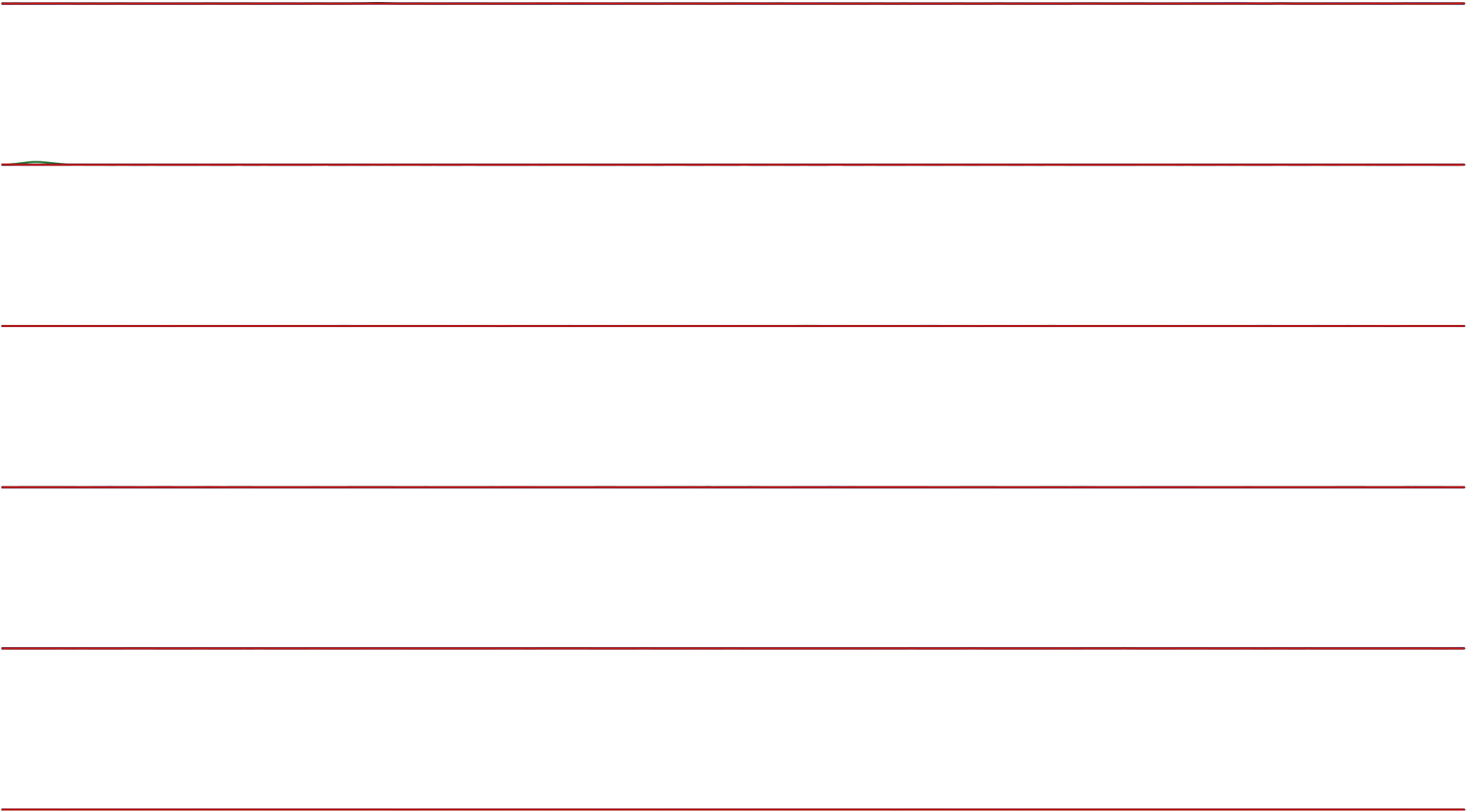

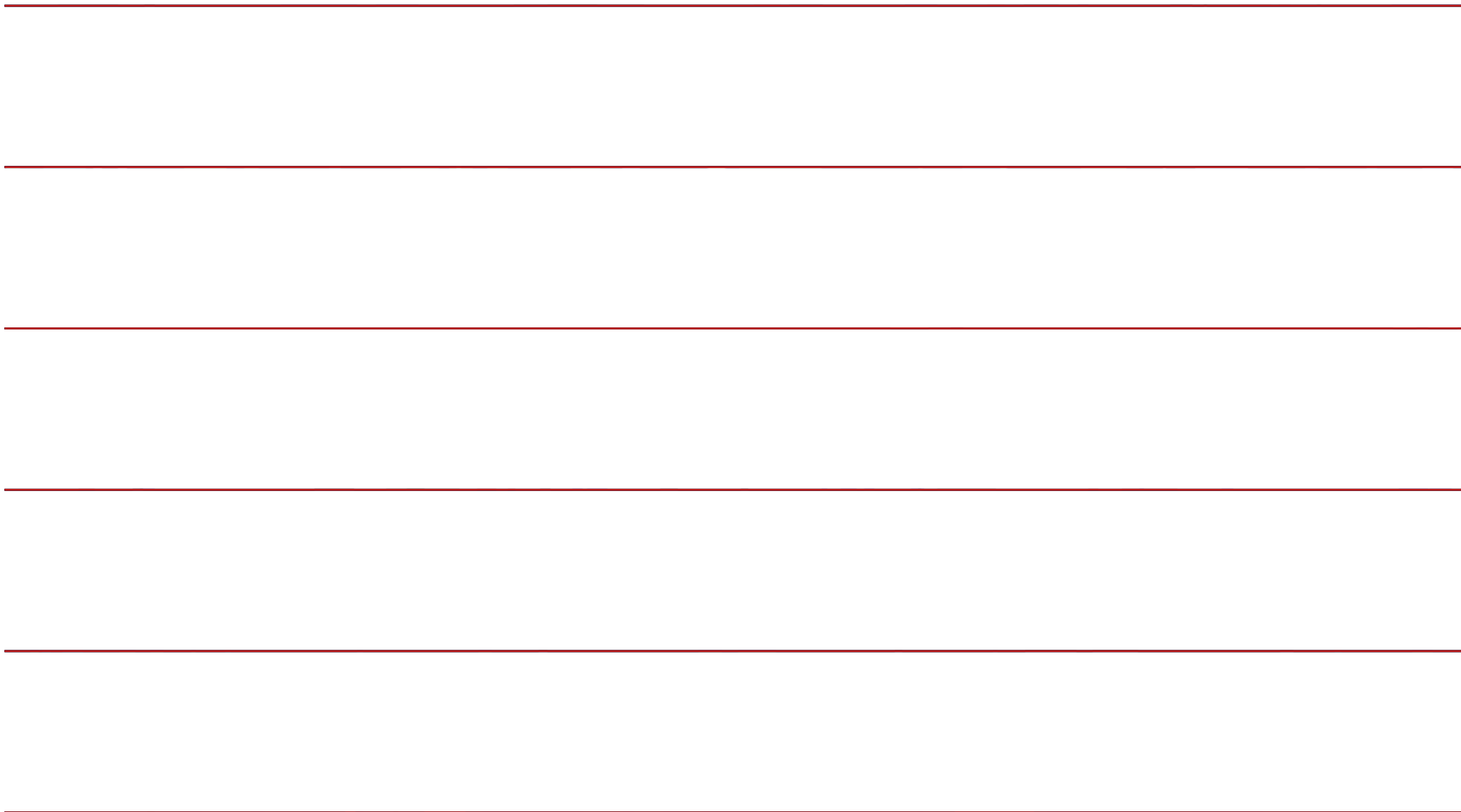

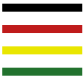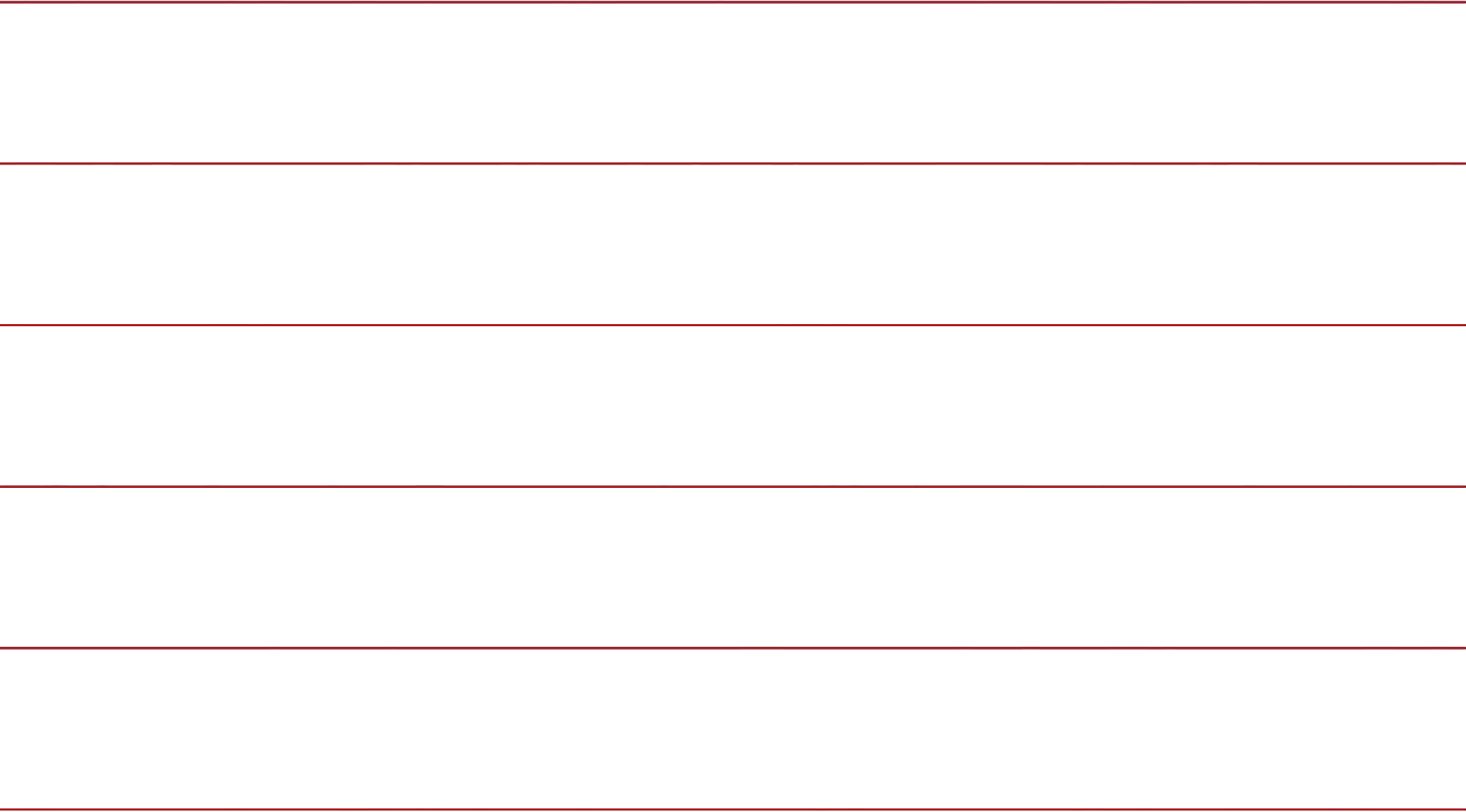

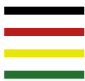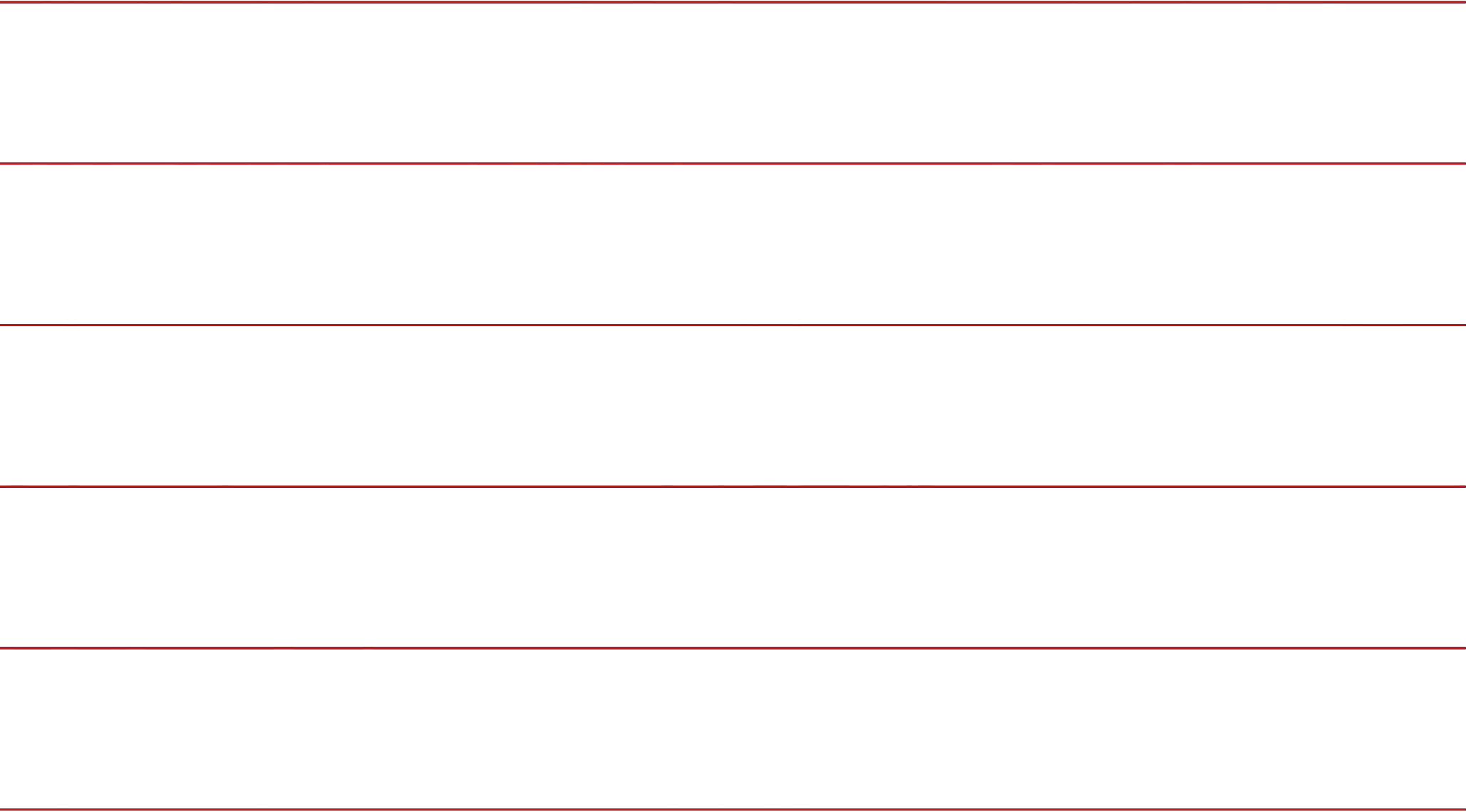

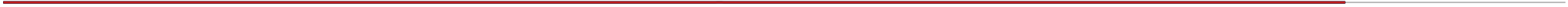

Supplement: Supplementary file 4 — Source data [file 41467_2026_68558_MOESM4_ESM.zip › Source data/Sanger-sequencing data/Suppl.Fig1f/BJ early SnrpnR3.pdf]

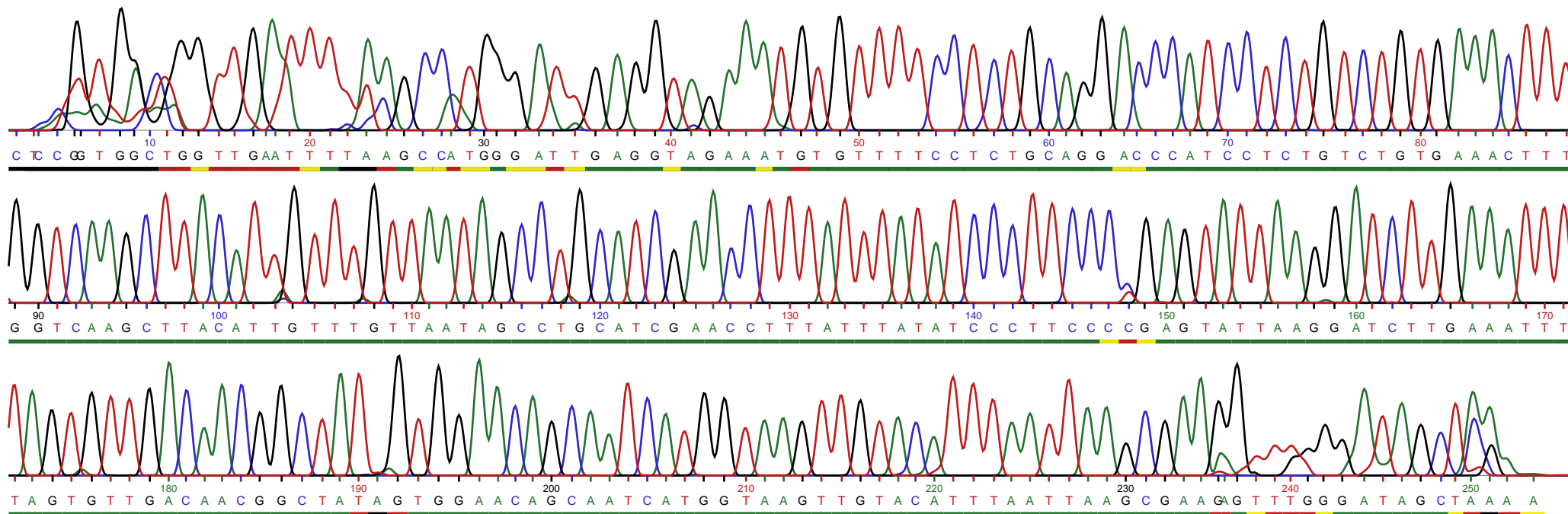

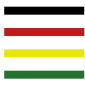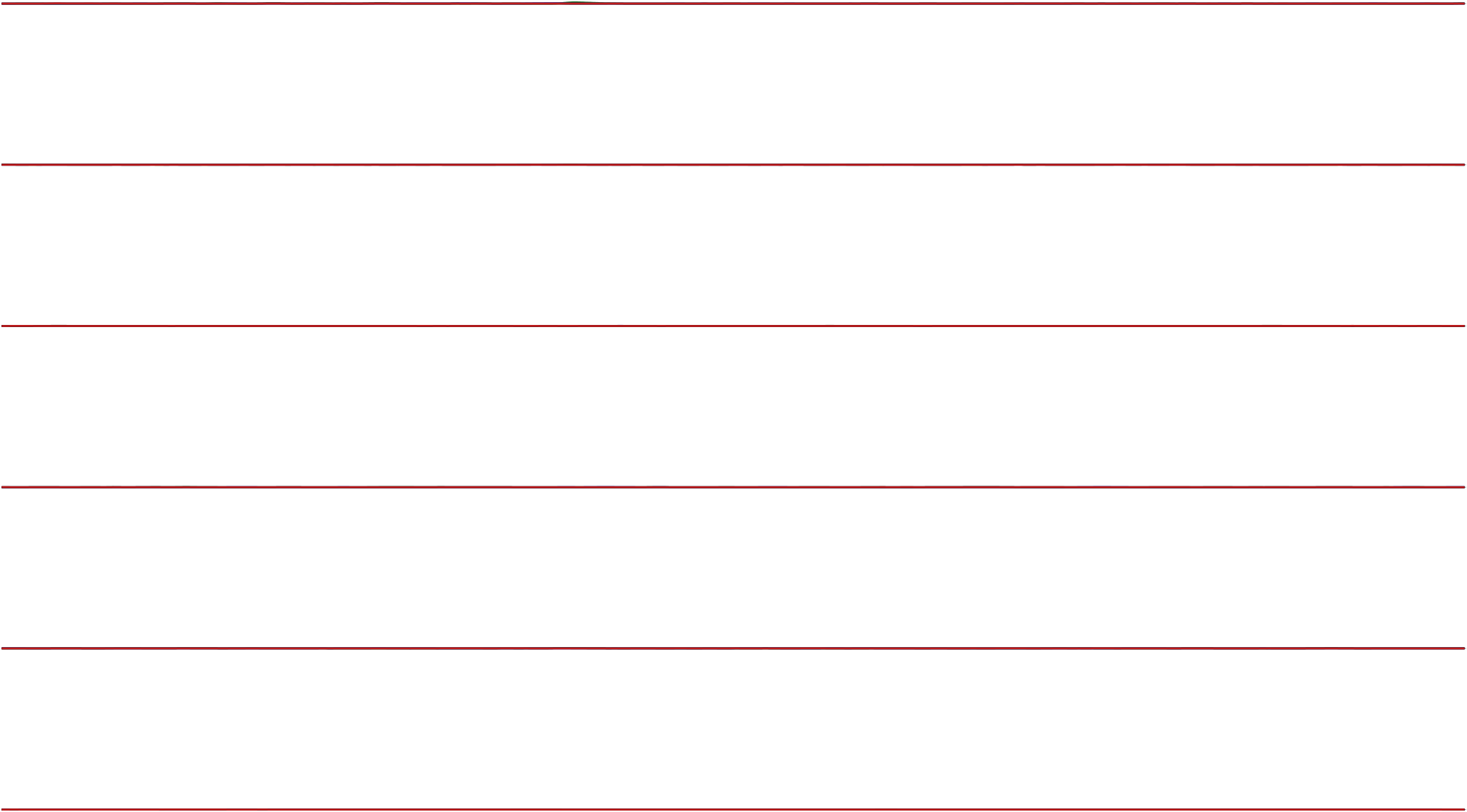

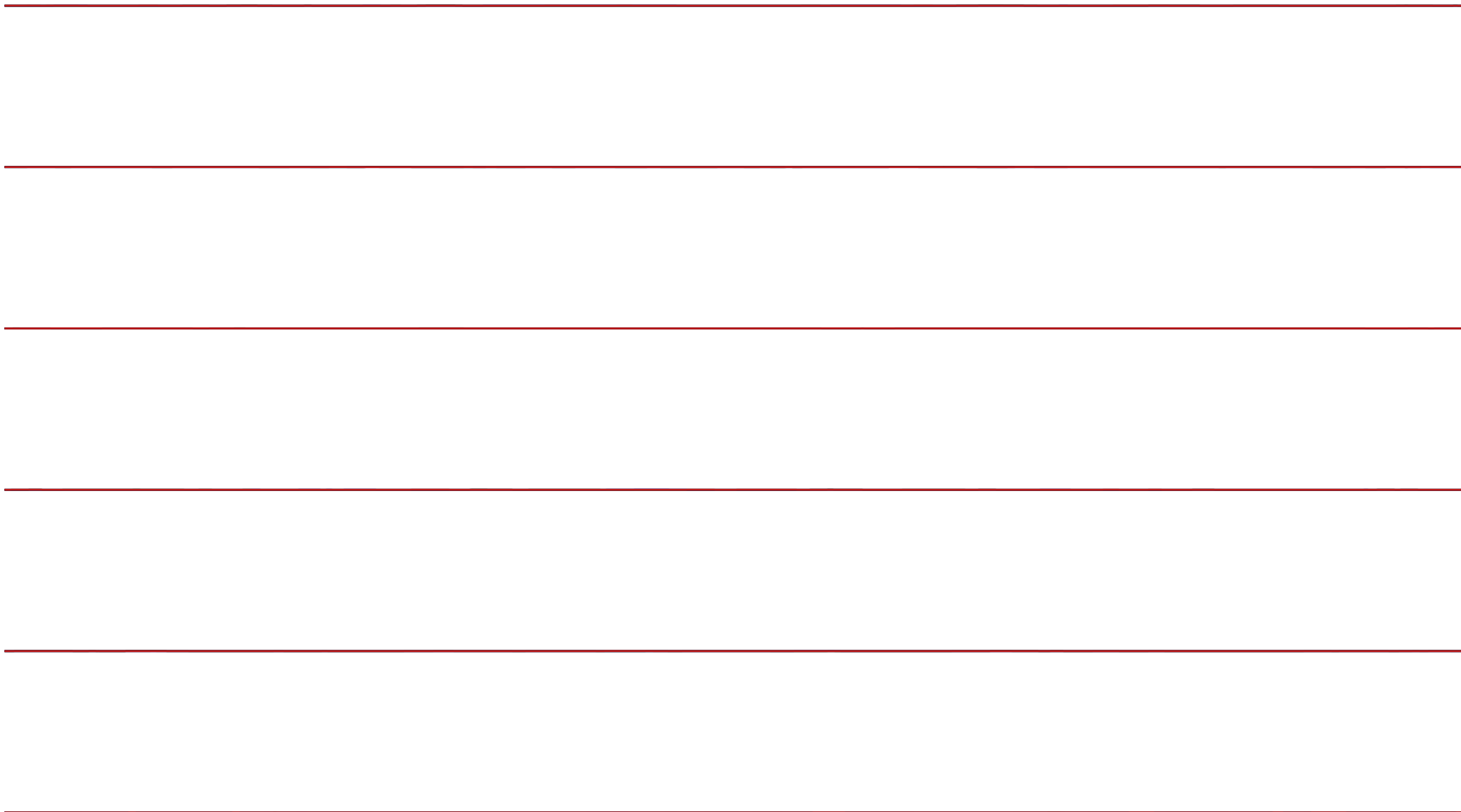

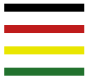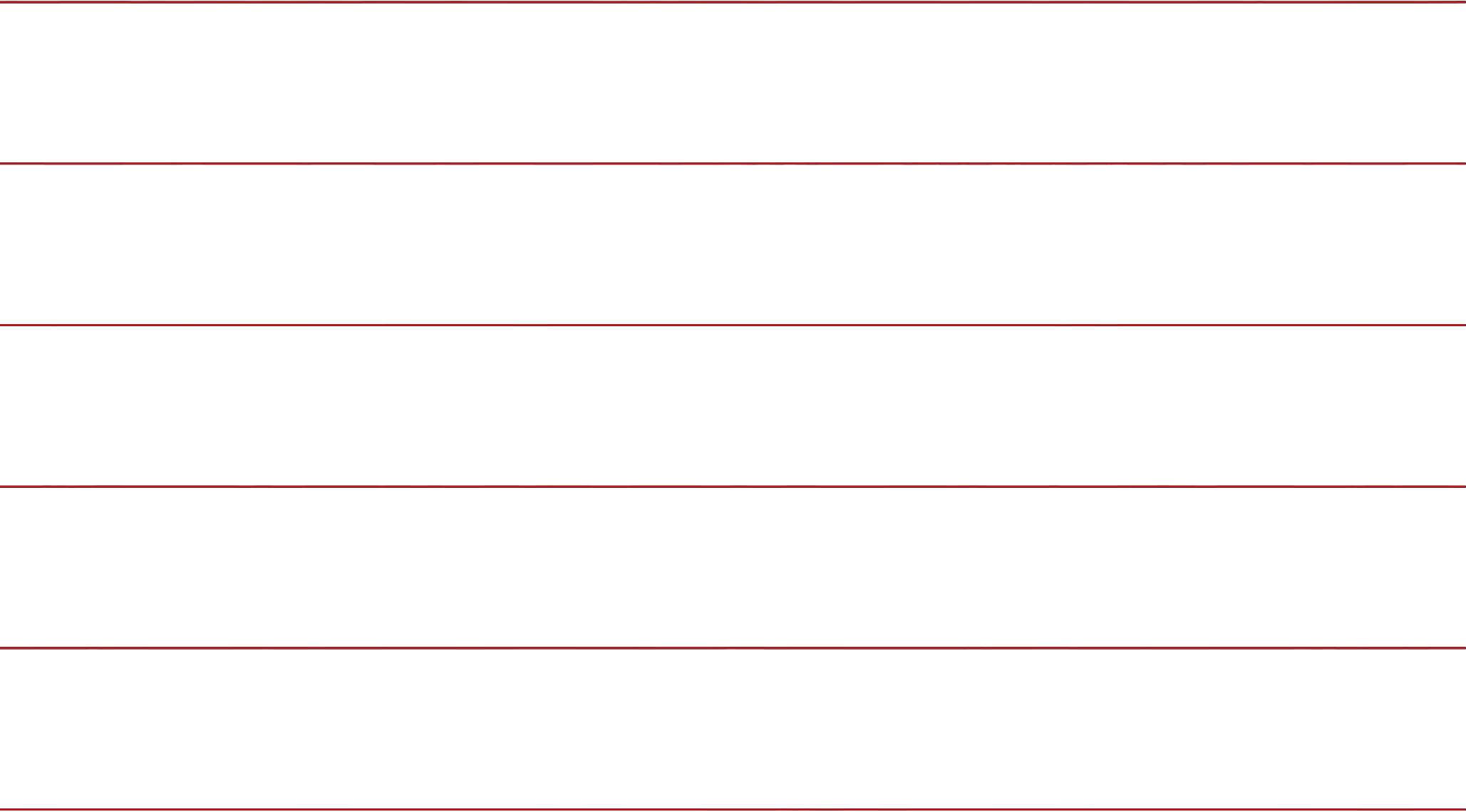

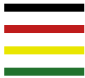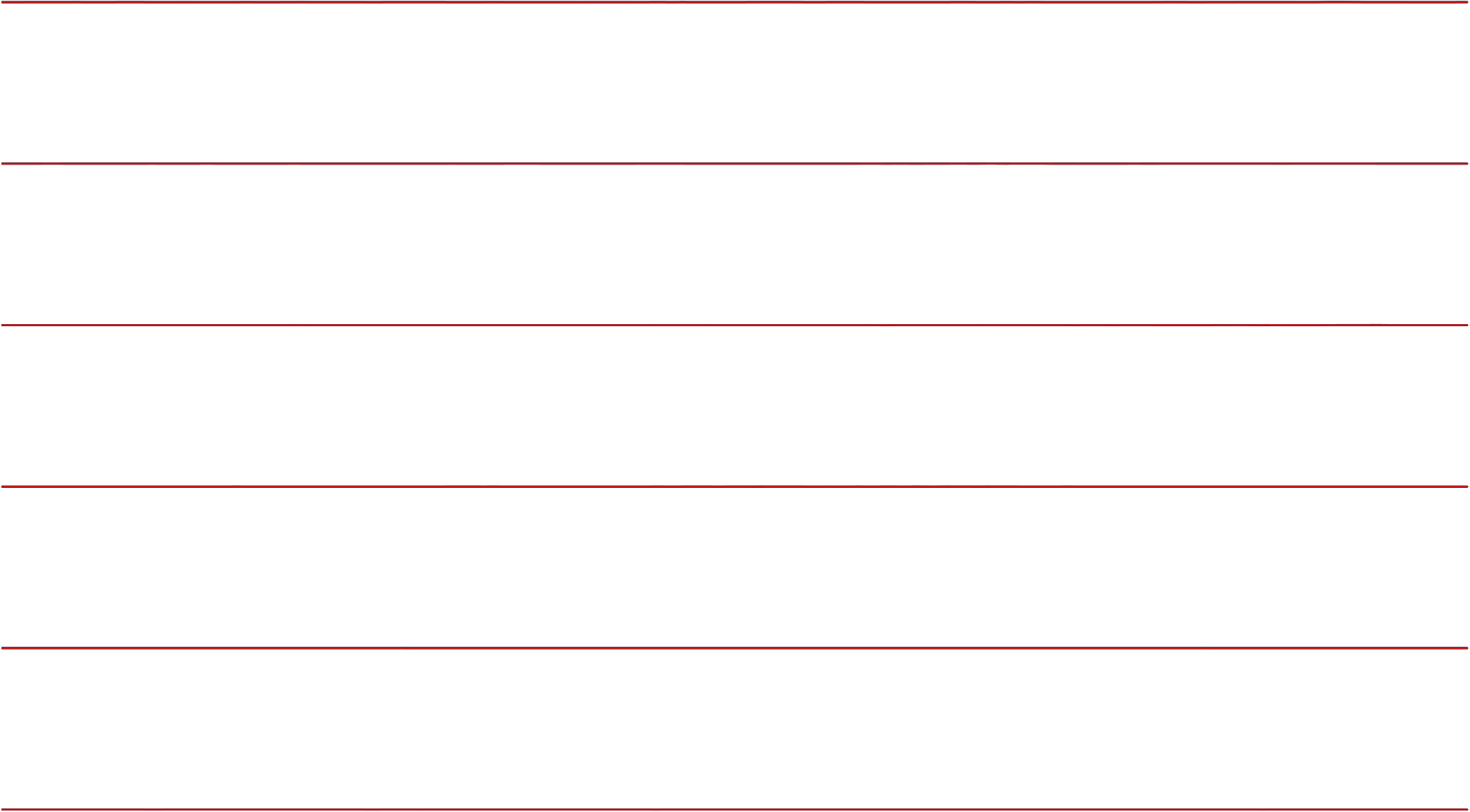

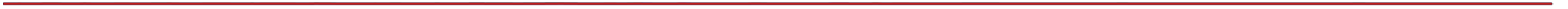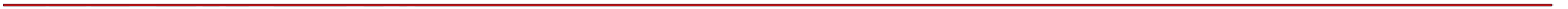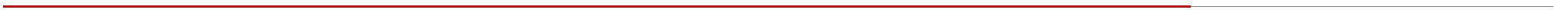

Supplement: Supplementary file 4 — Source data [file 41467_2026_68558_MOESM4_ESM.zip › Source data/Sanger-sequencing data/Suppl.Fig1f/BJ late SnrpnR1.pdf]

Sequence: EF72763573

Samples: 16300  
Bases: 475  
Average spacing: 35.0  
Average quality >= 10: 166, 20: 31, 30: 230

Quality: 0 - 9  
10 - 19  
20 - 29  
≥ 30

Page: 1 / 3  
24.04.2025

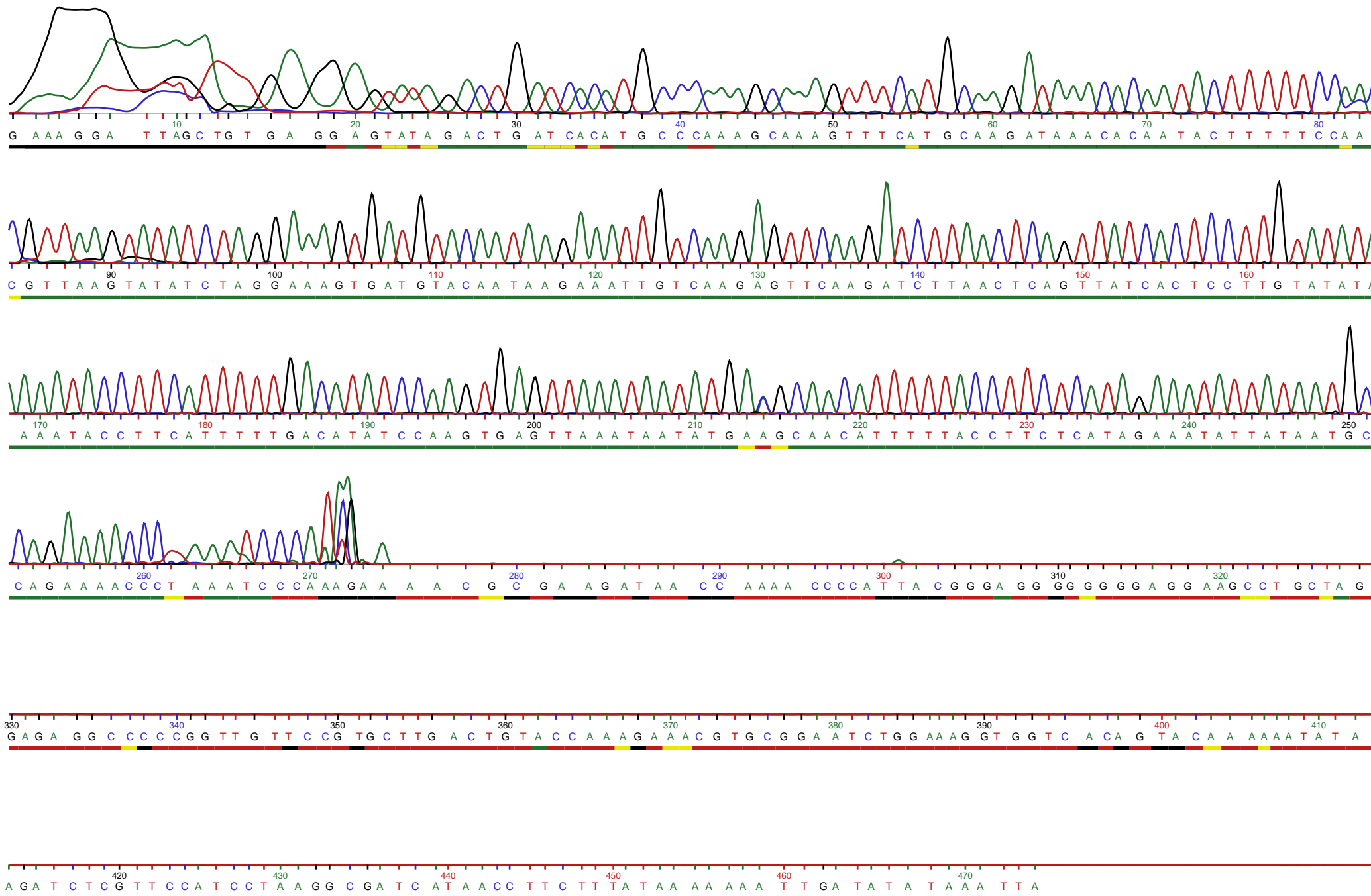

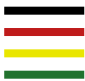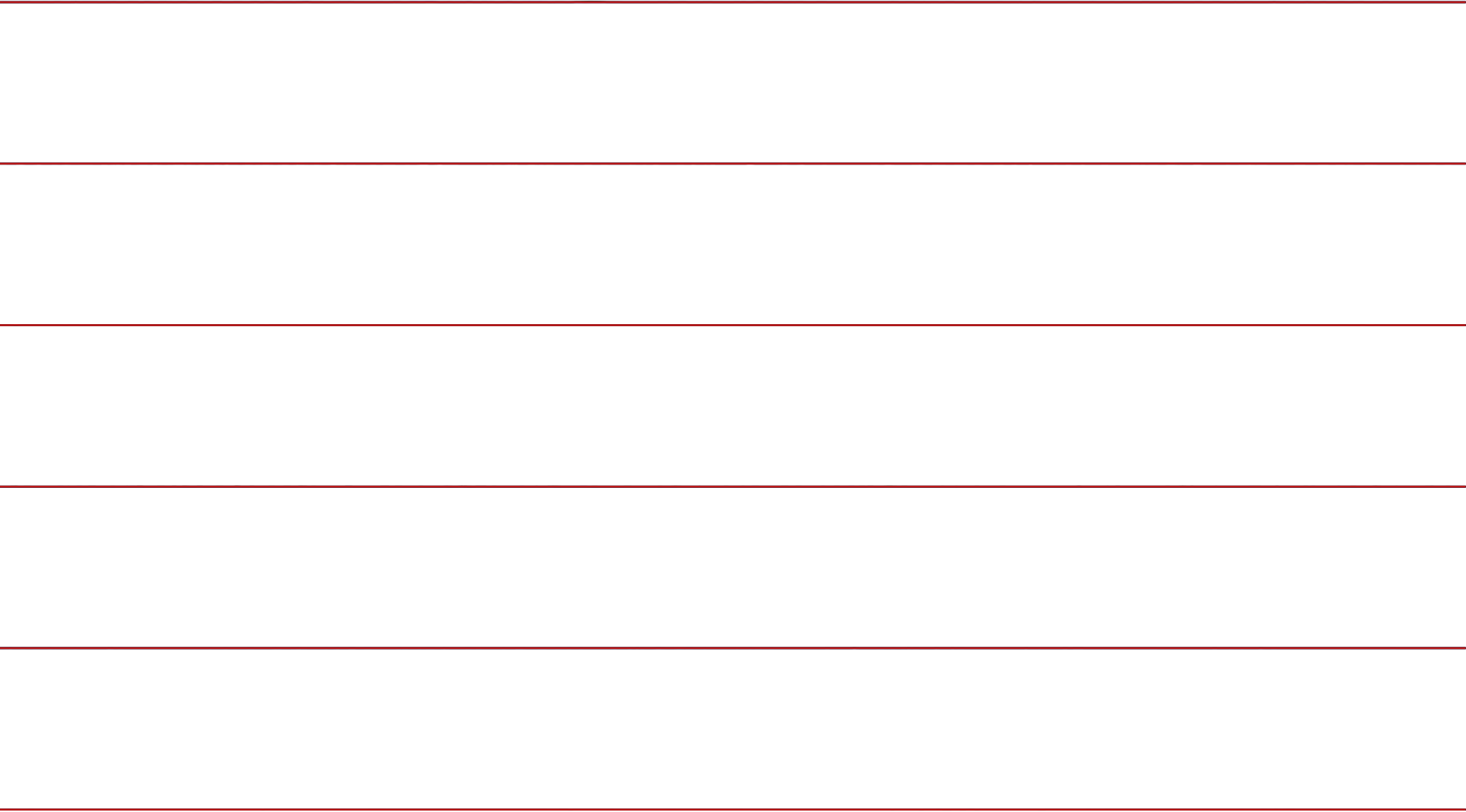

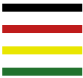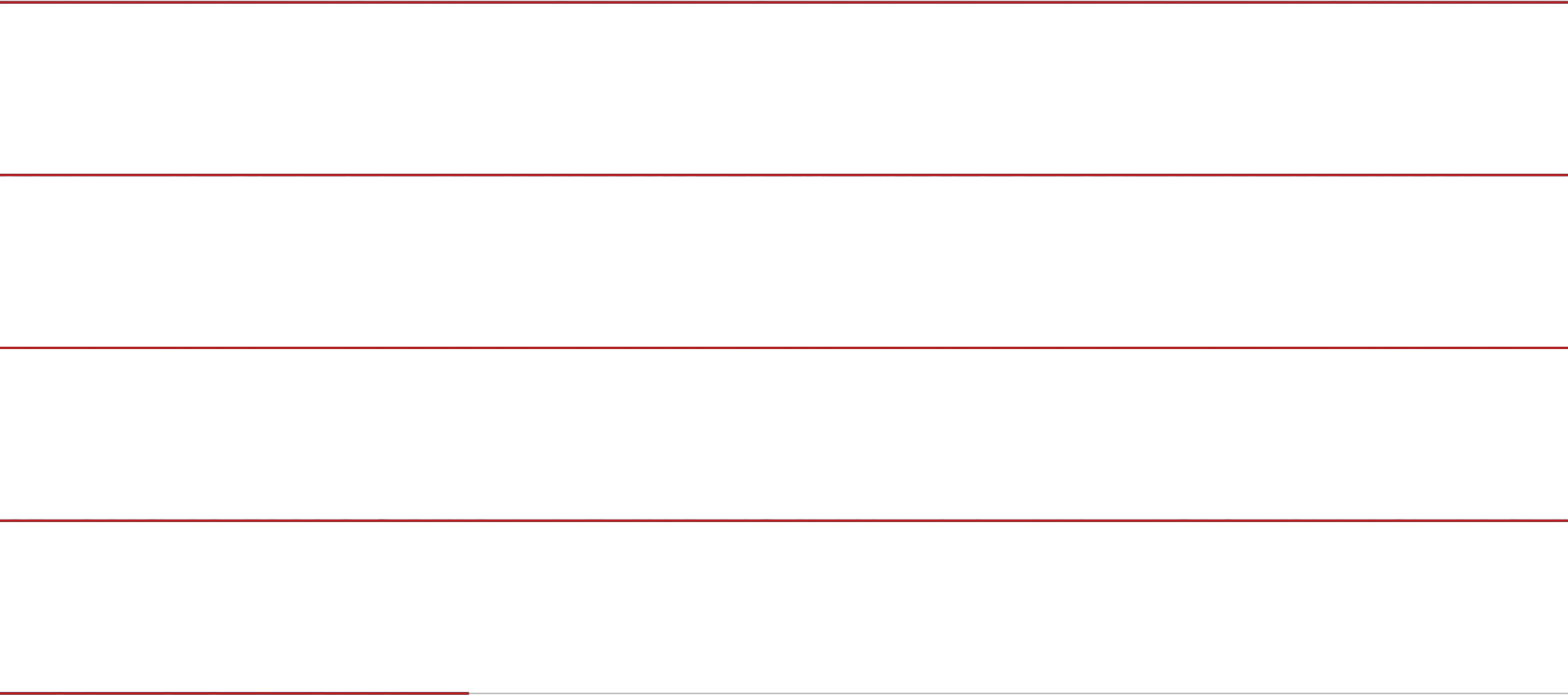

Supplement: Supplementary file 4 — Source data [file 41467_2026_68558_MOESM4_ESM.zip › Source data/Sanger-sequencing data/Suppl.Fig1f/BJ late SnrpnR3.pdf]

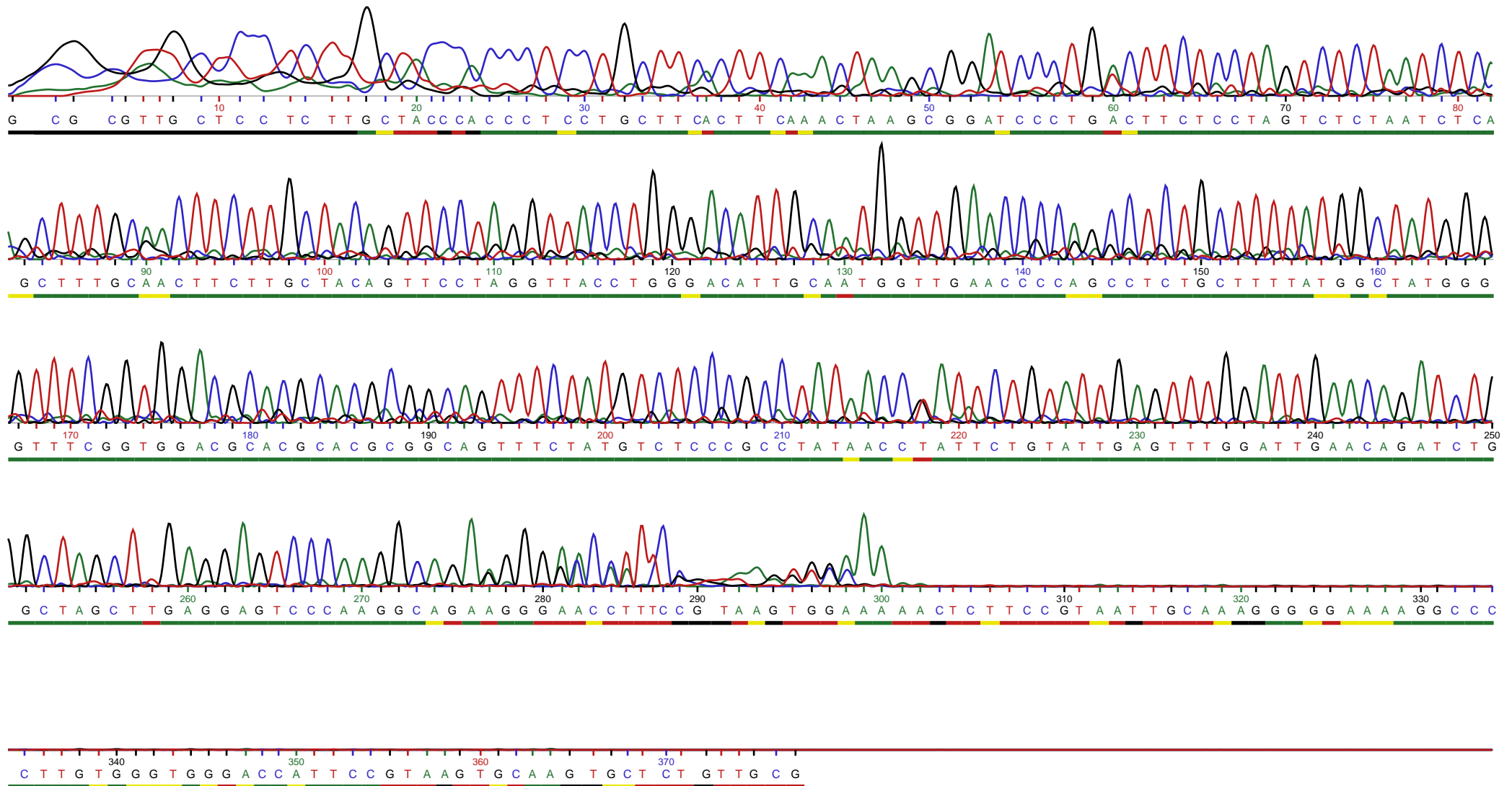

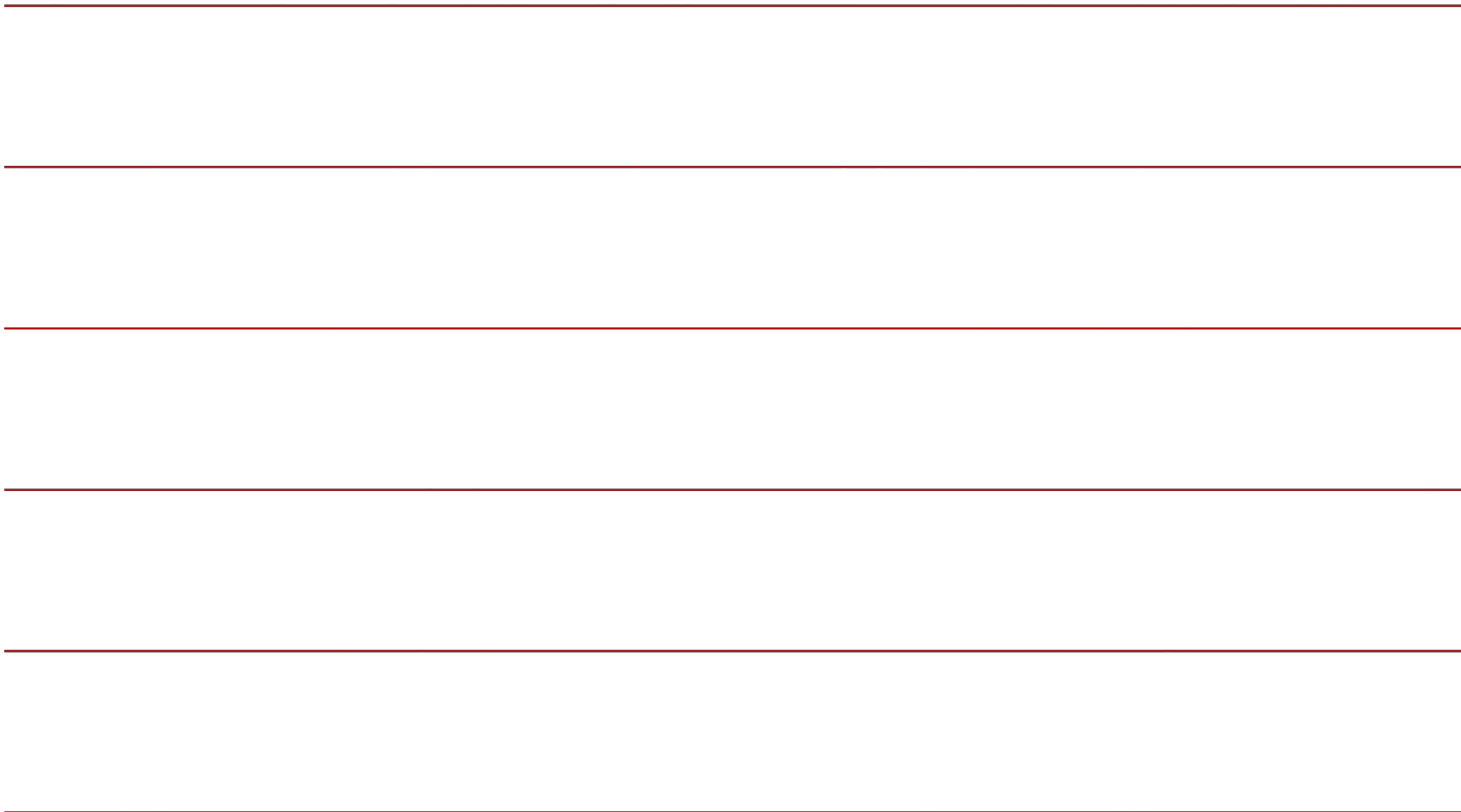

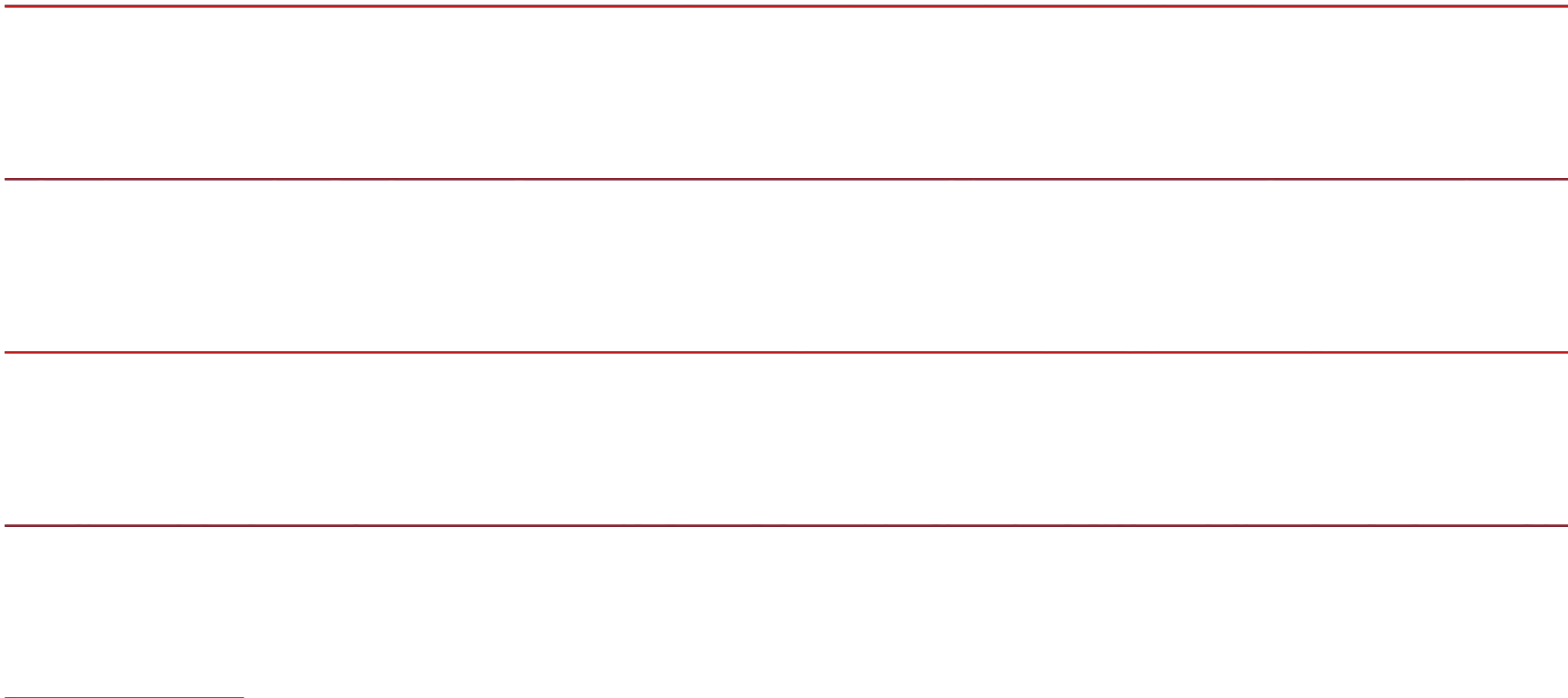

Supplement: Supplementary file 4 — Source data [file 41467_2026_68558_MOESM4_ESM.zip › Source data/Sanger-sequencing data/Suppl.Fig1g/BJ early H19.pdf]

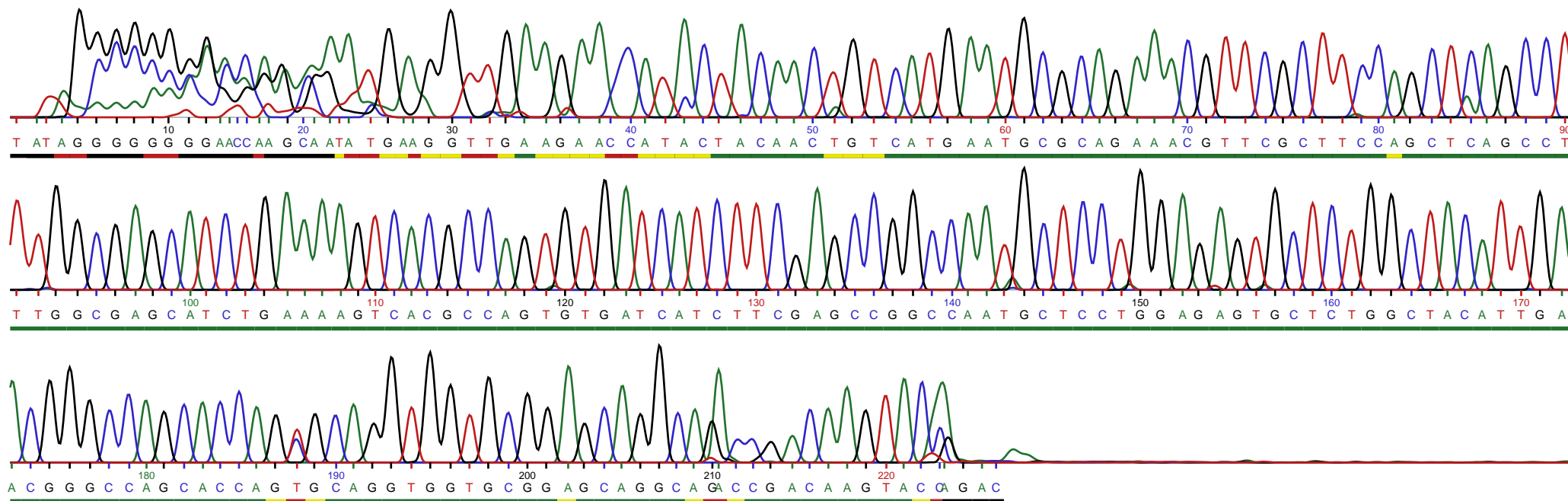

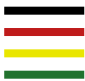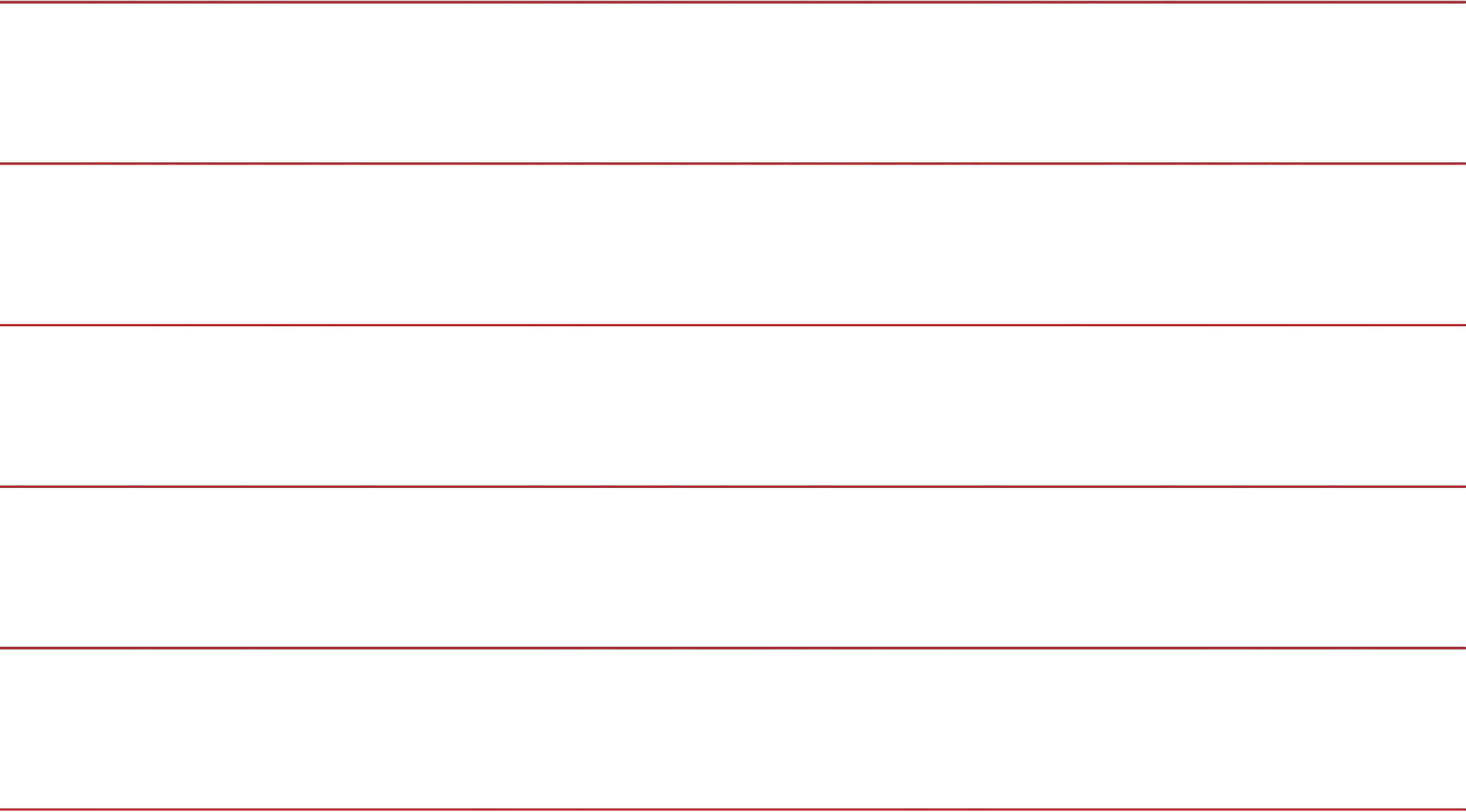

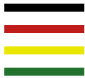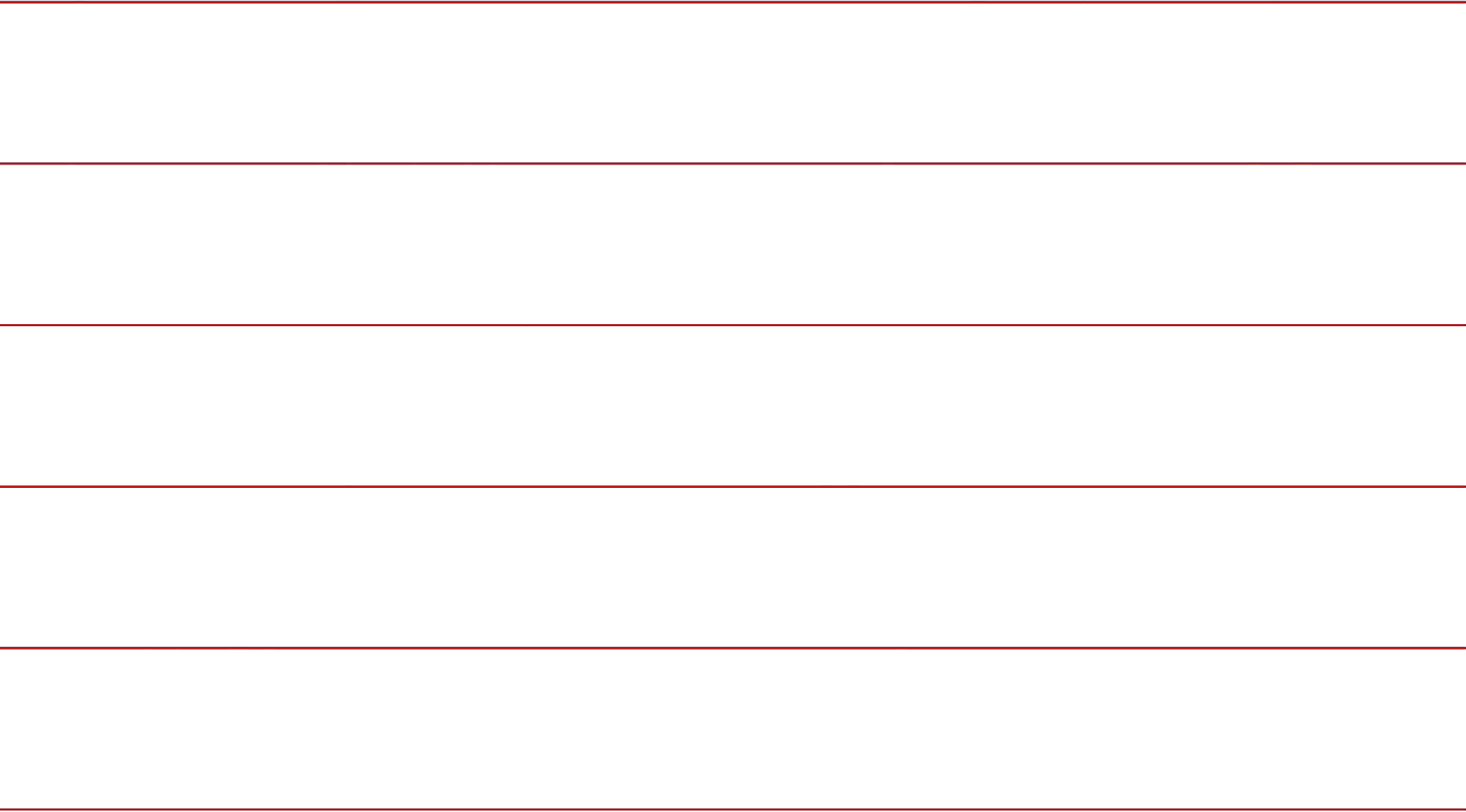

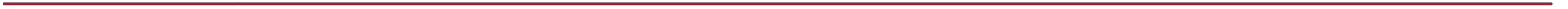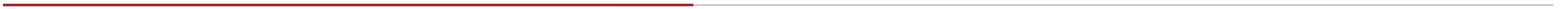

Supplement: Supplementary file 4 — Source data [file 41467_2026_68558_MOESM4_ESM.zip › Source data/Sanger-sequencing data/Suppl.Fig1g/BJ early Peg3.pdf]

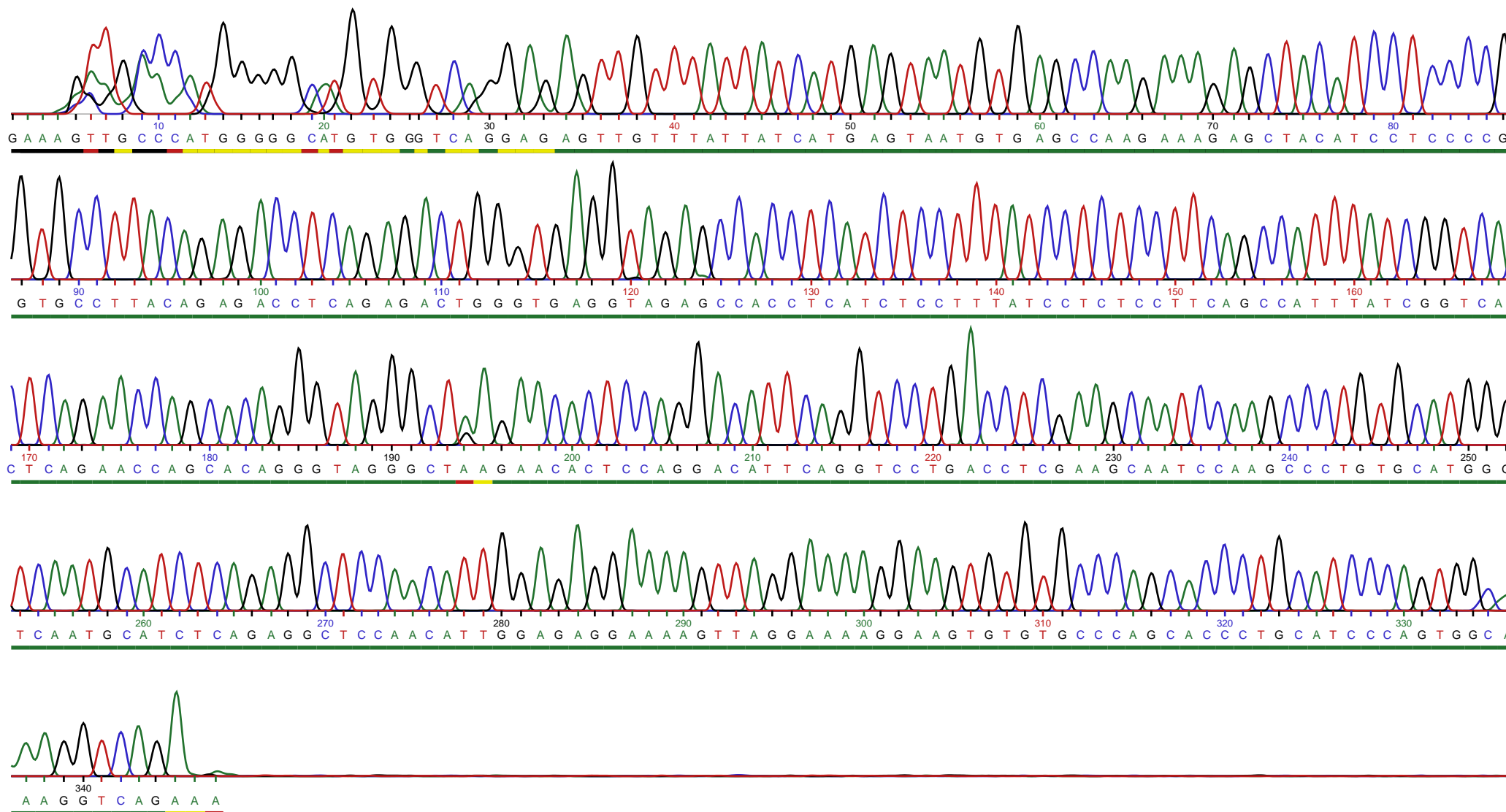

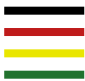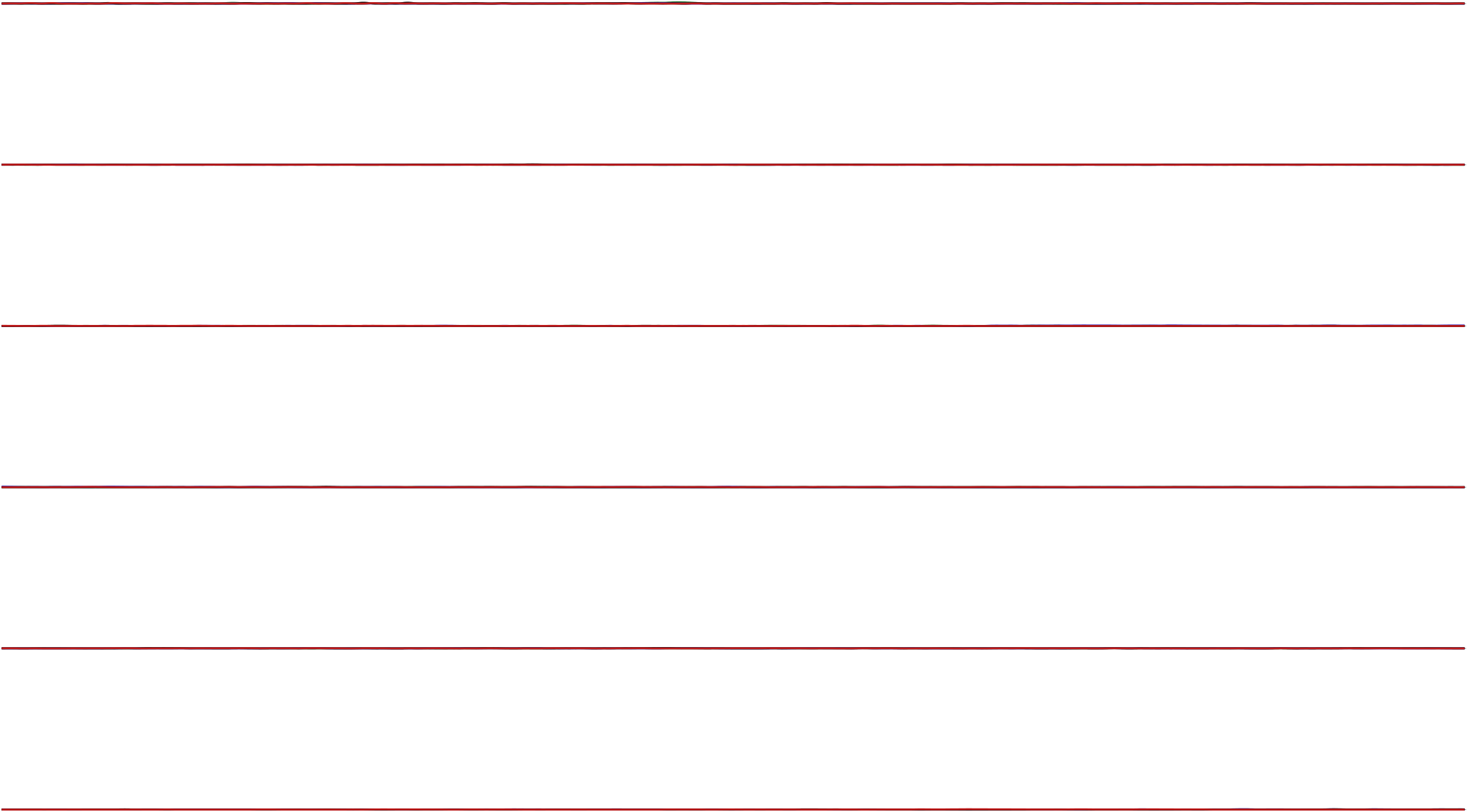

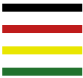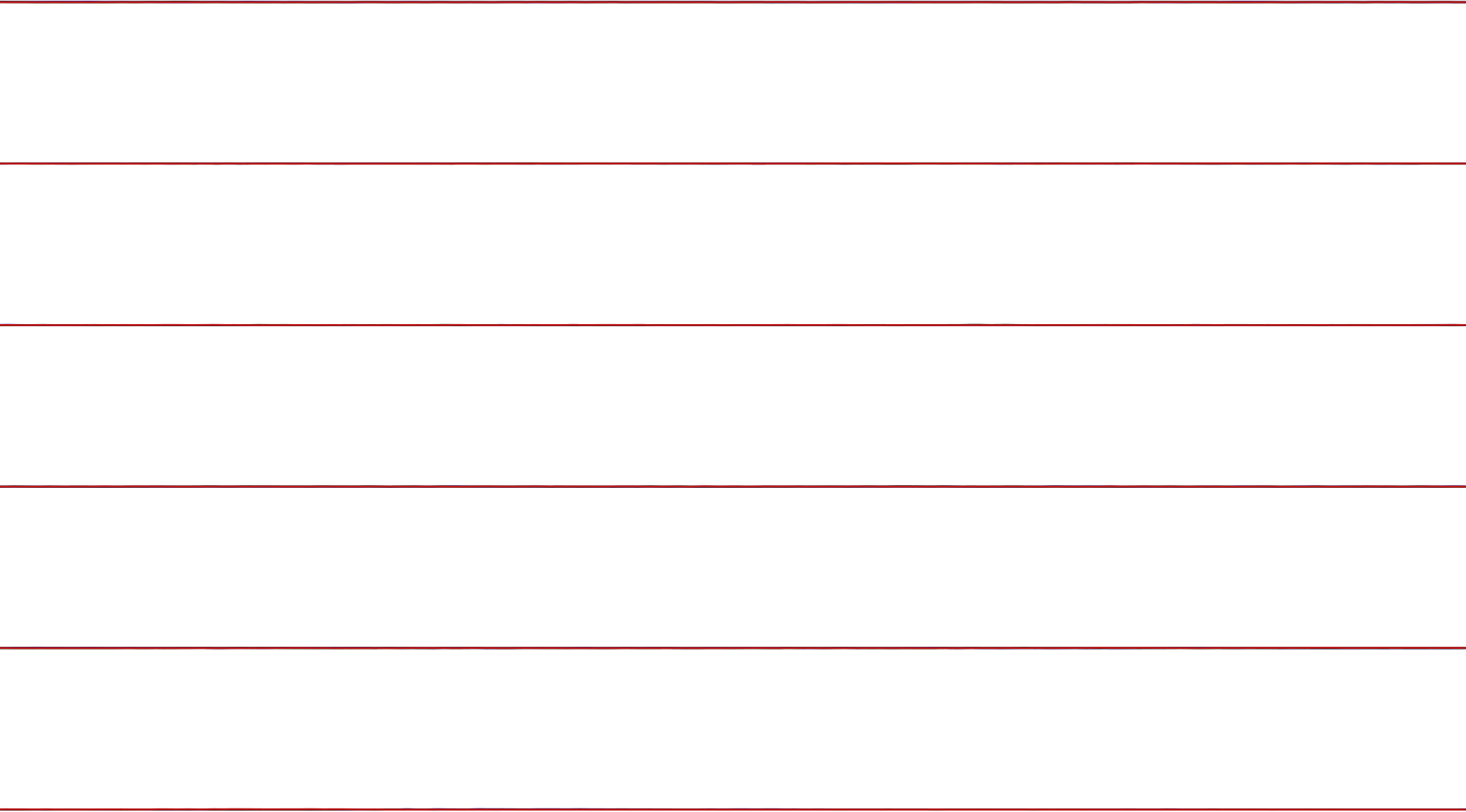

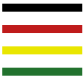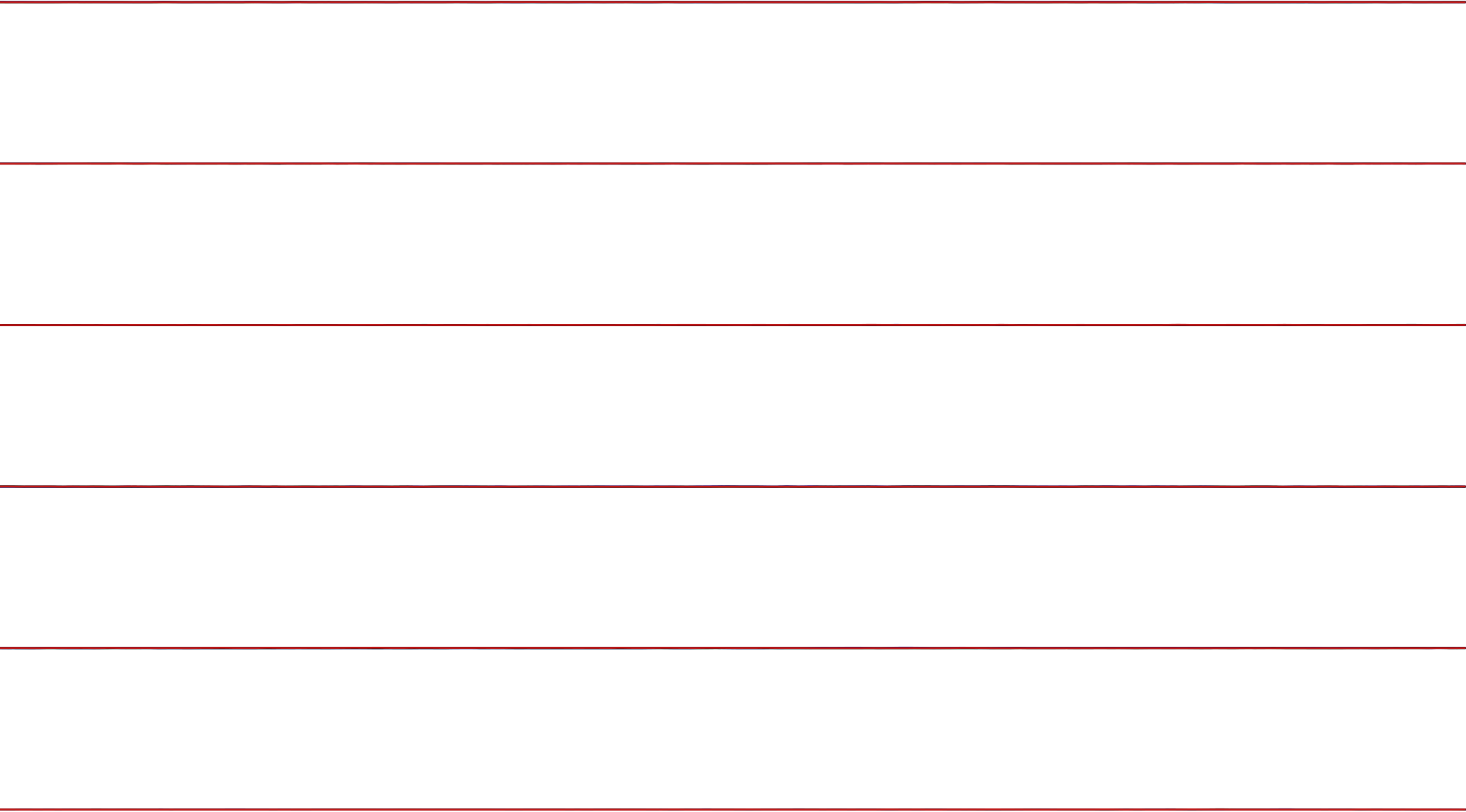

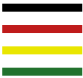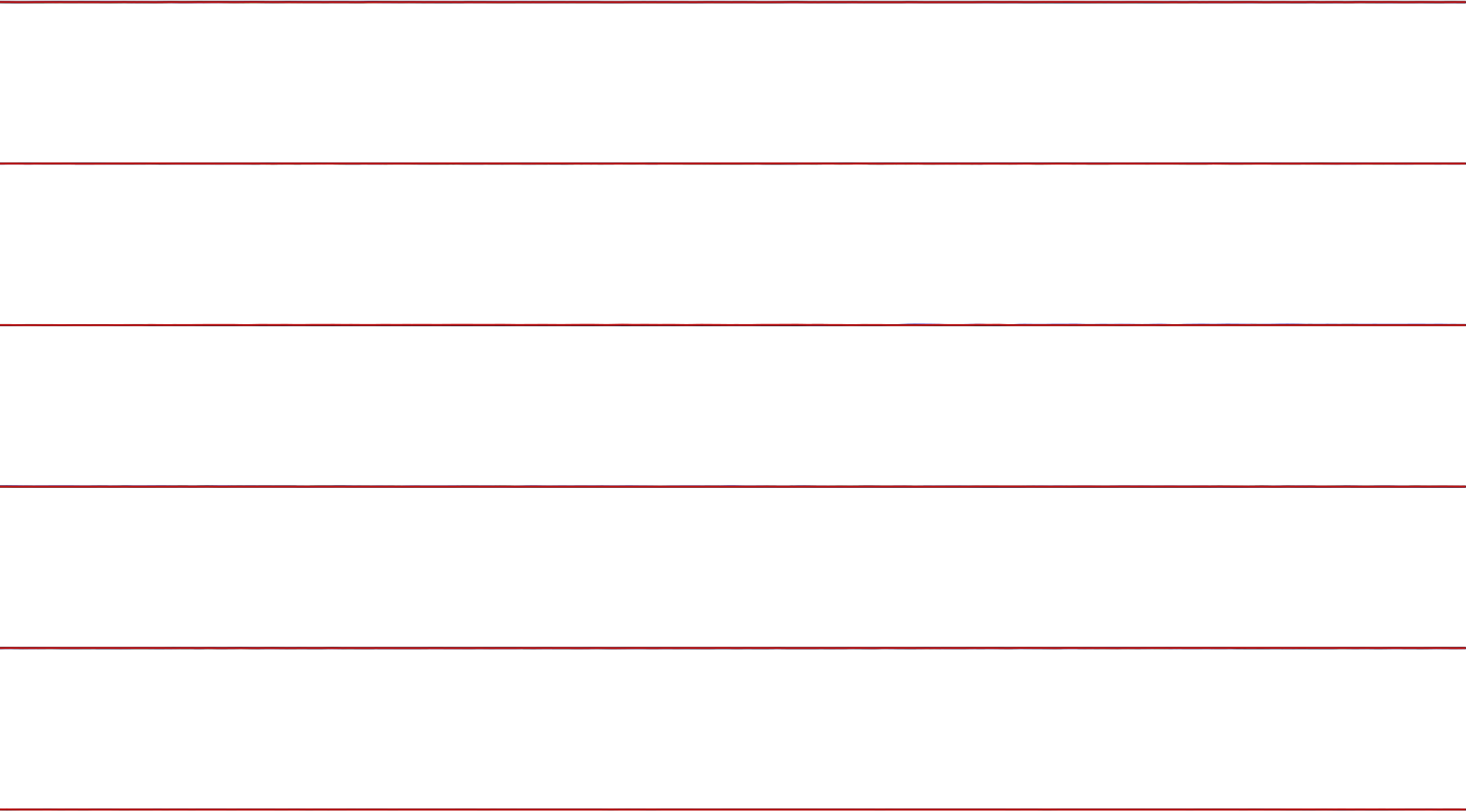

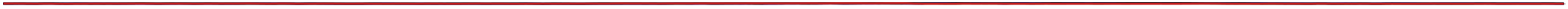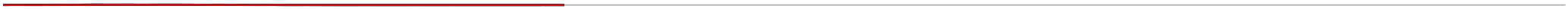

Supplement: Supplementary file 4 — Source data [file 41467_2026_68558_MOESM4_ESM.zip › Source data/Sanger-sequencing data/Suppl.Fig1g/BJ early Rasgrf1.pdf]

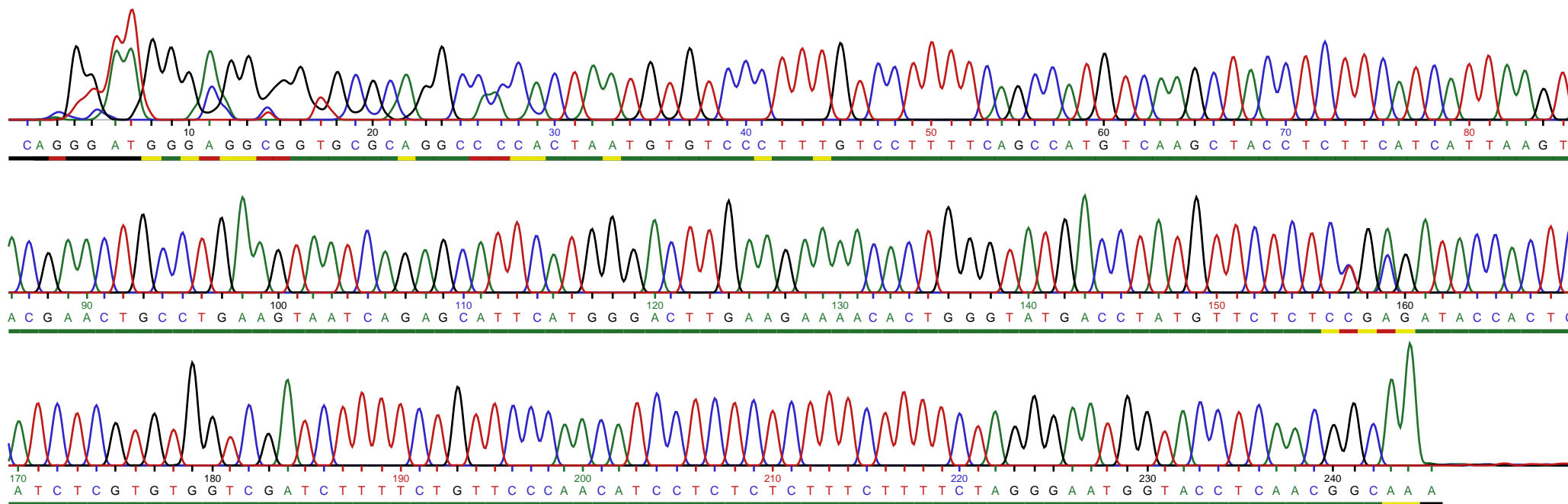

Sequence: EF72763598

Samples: 26912  
Bases: 246  
Average spacing: 110.0  
Average quality >= 10: 8, 20: 15, 30: 215

Quality: 0 - 9  
10 - 19  
20 - 29  
>= 30

Page: 2 / 5  
14.05.2025

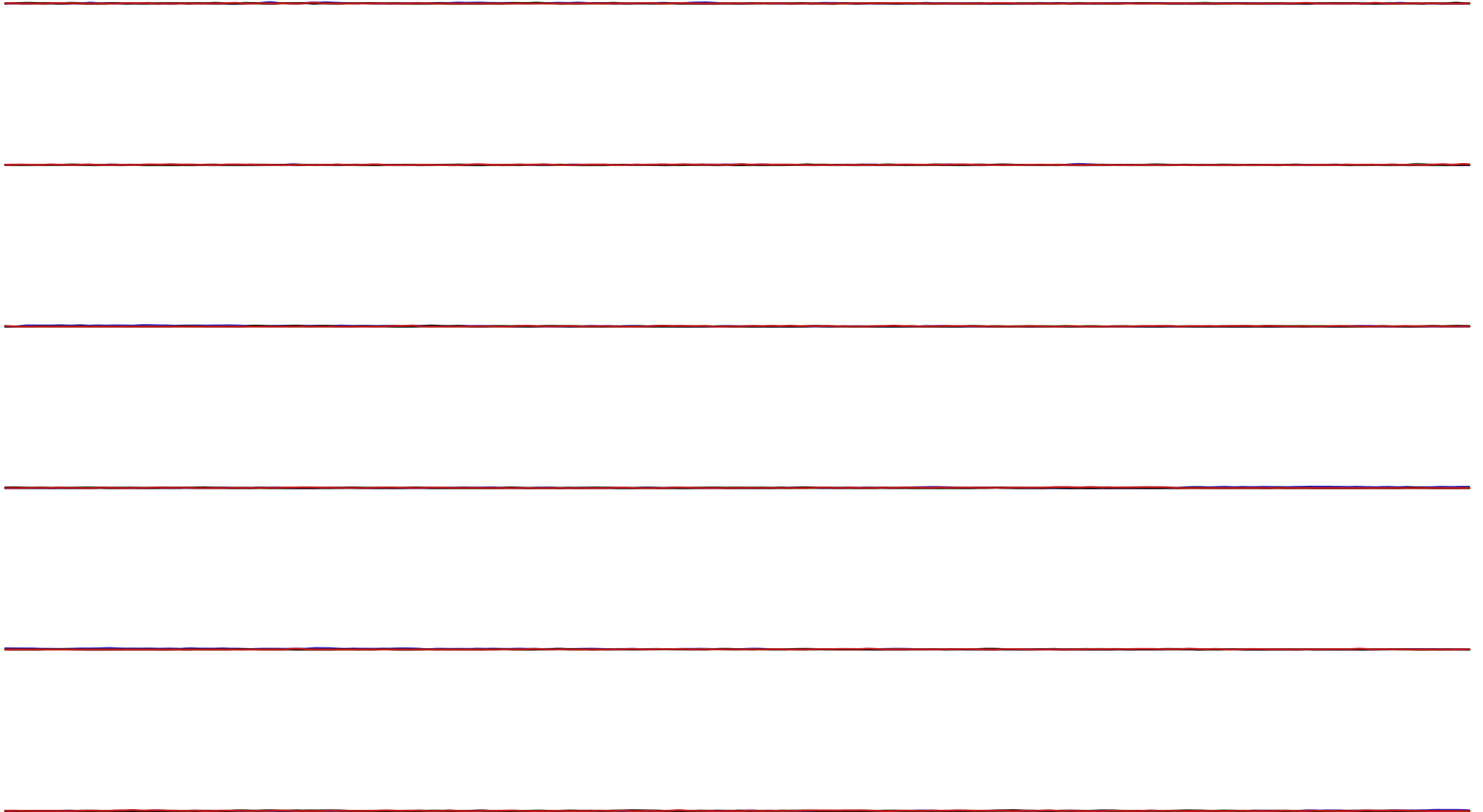

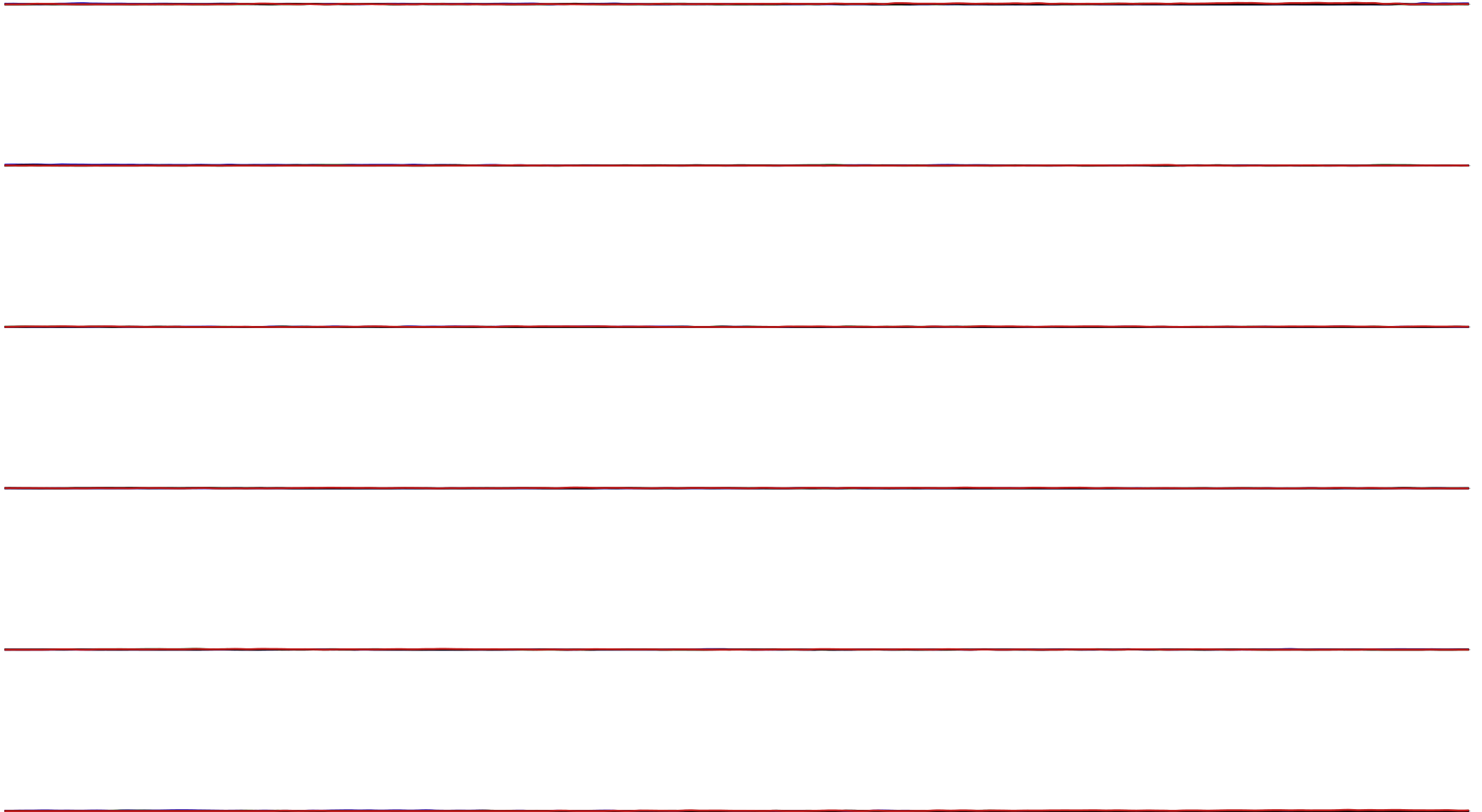

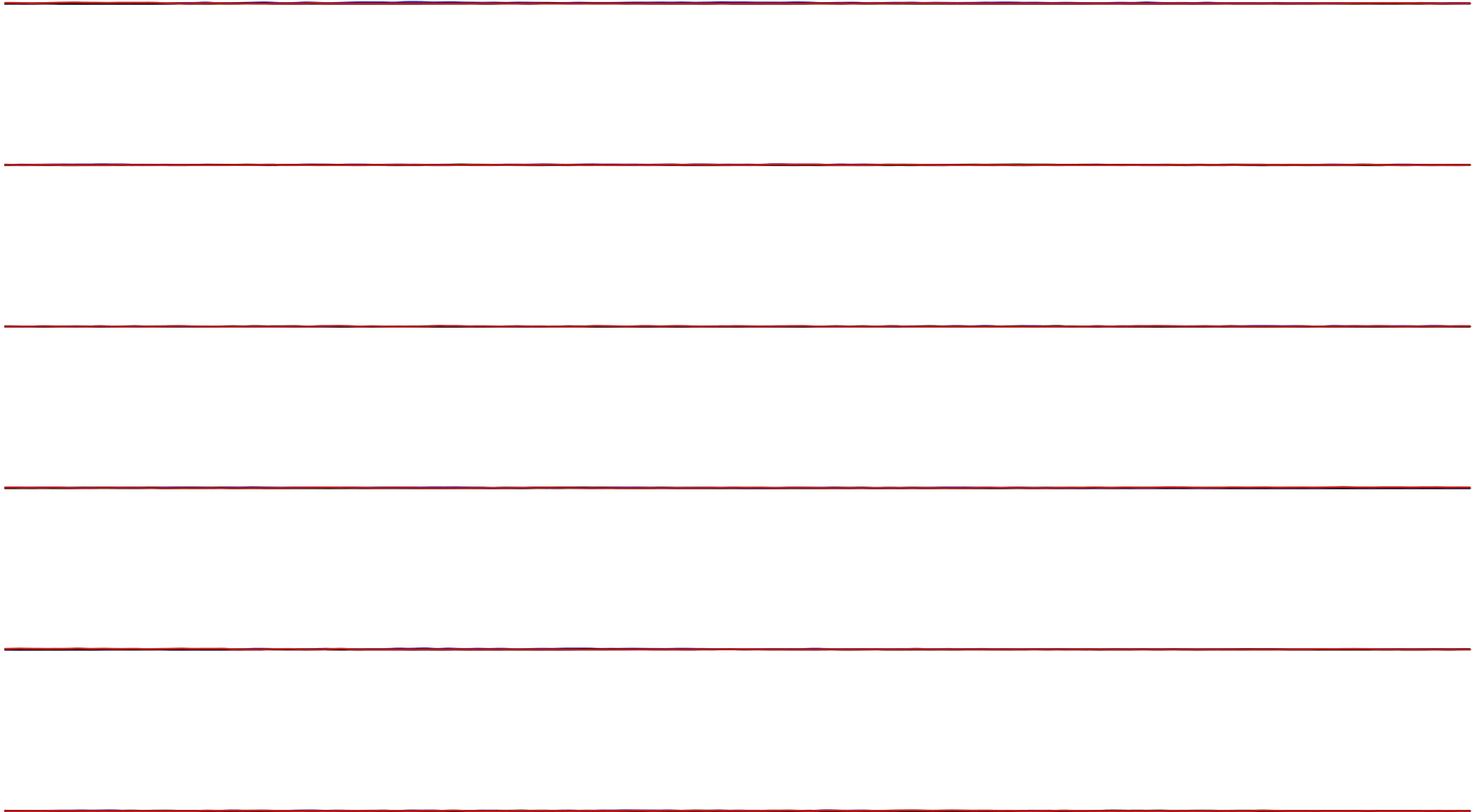

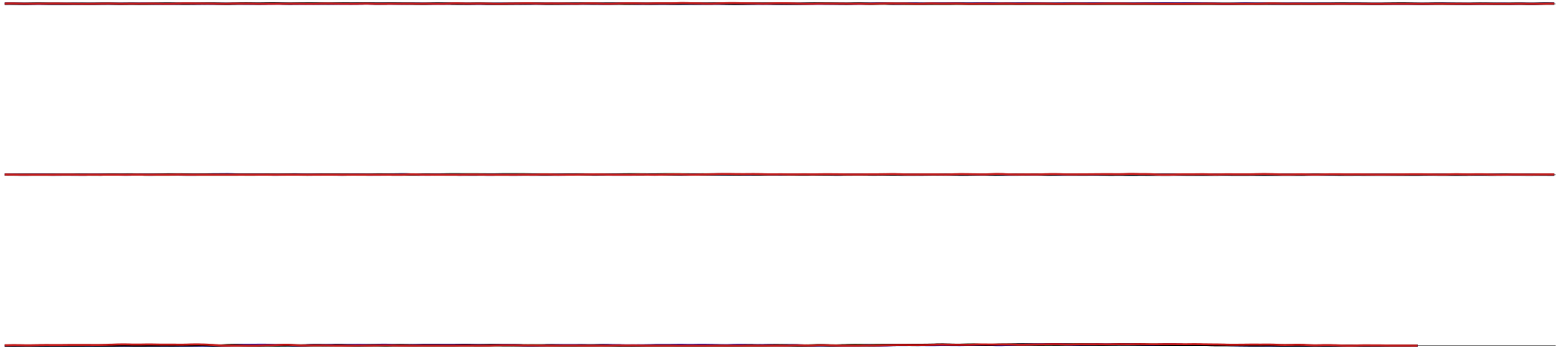

Supplement: Supplementary file 4 — Source data [file 41467_2026_68558_MOESM4_ESM.zip › Source data/Sanger-sequencing data/Suppl.Fig1g/BJ early Slc38a4.pdf]

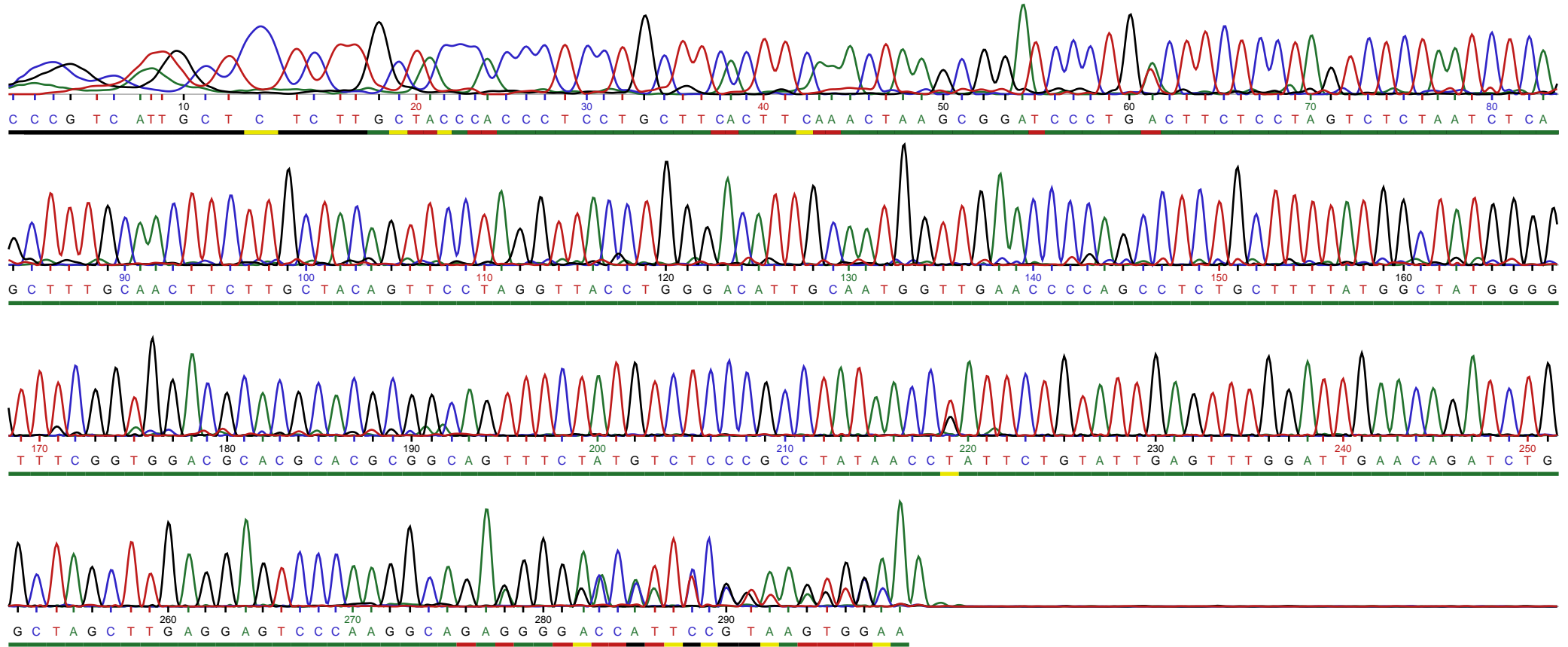

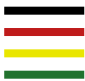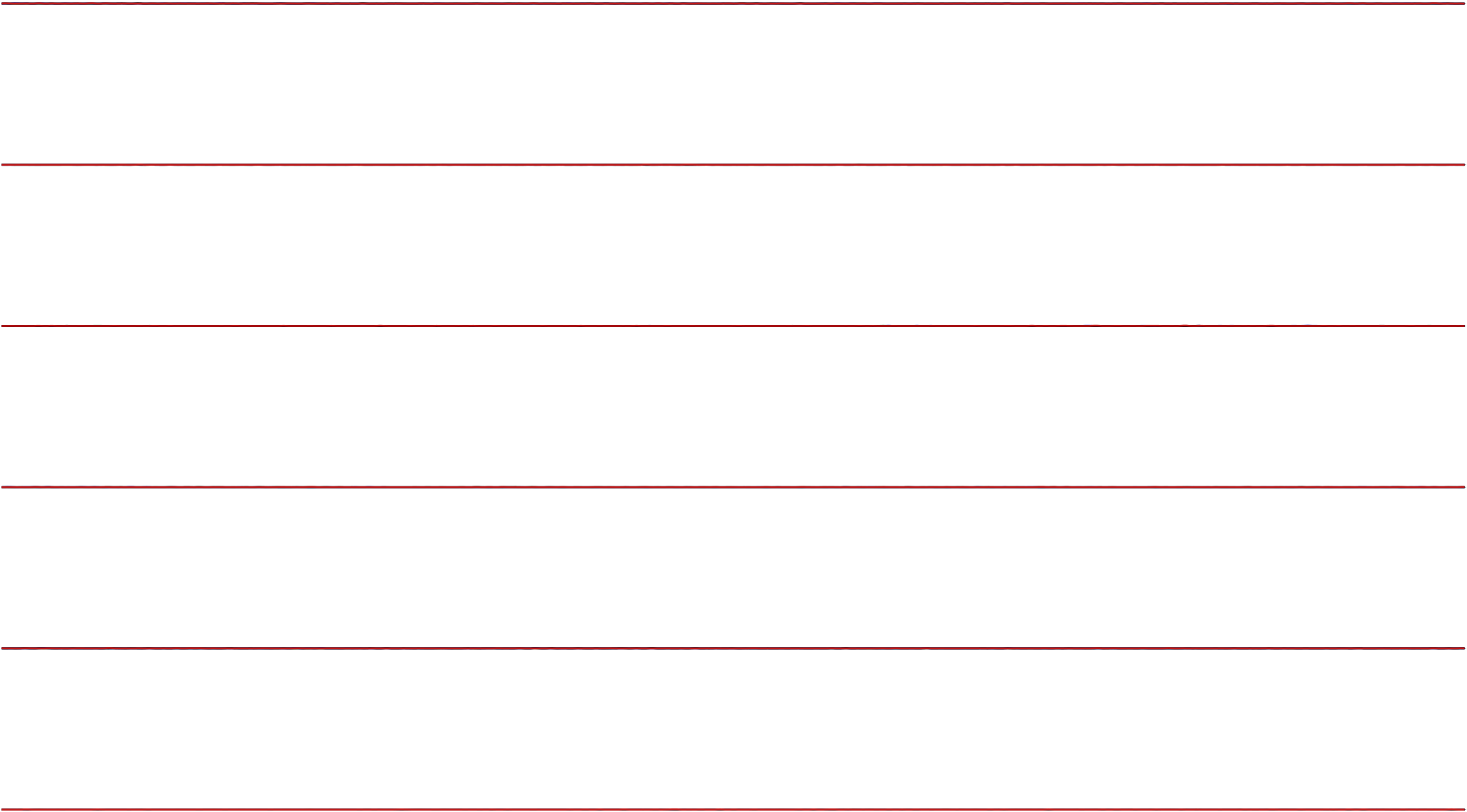

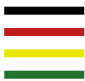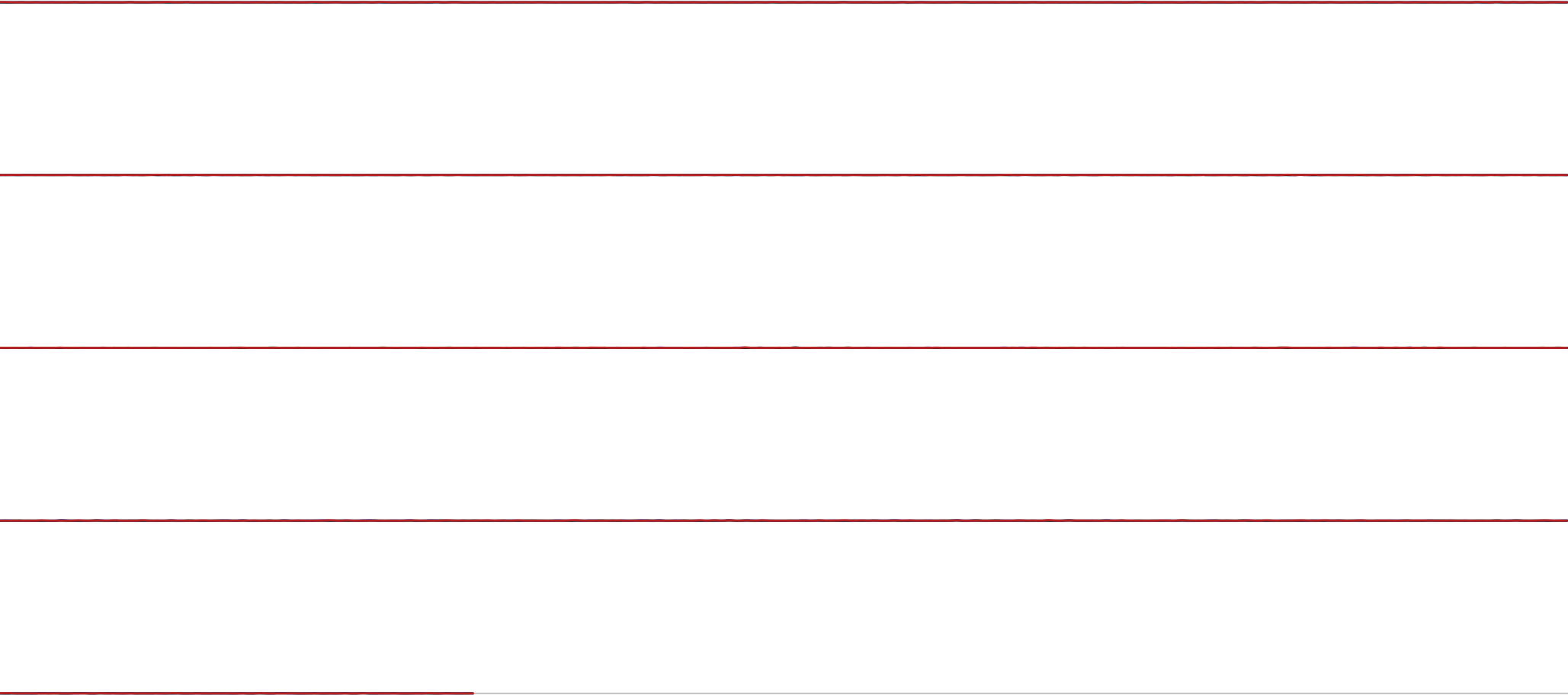

Supplement: Supplementary file 4 — Source data [file 41467_2026_68558_MOESM4_ESM.zip › Source data/Sanger-sequencing data/Suppl.Fig1g/BJ late H19.pdf]

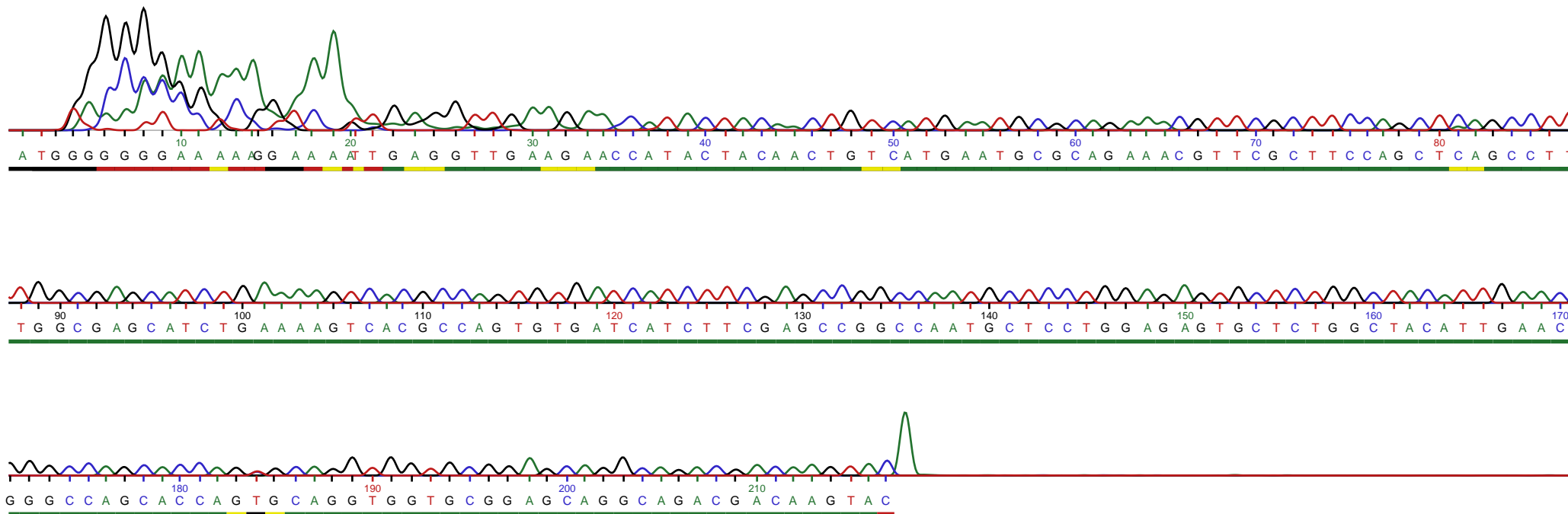

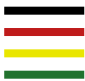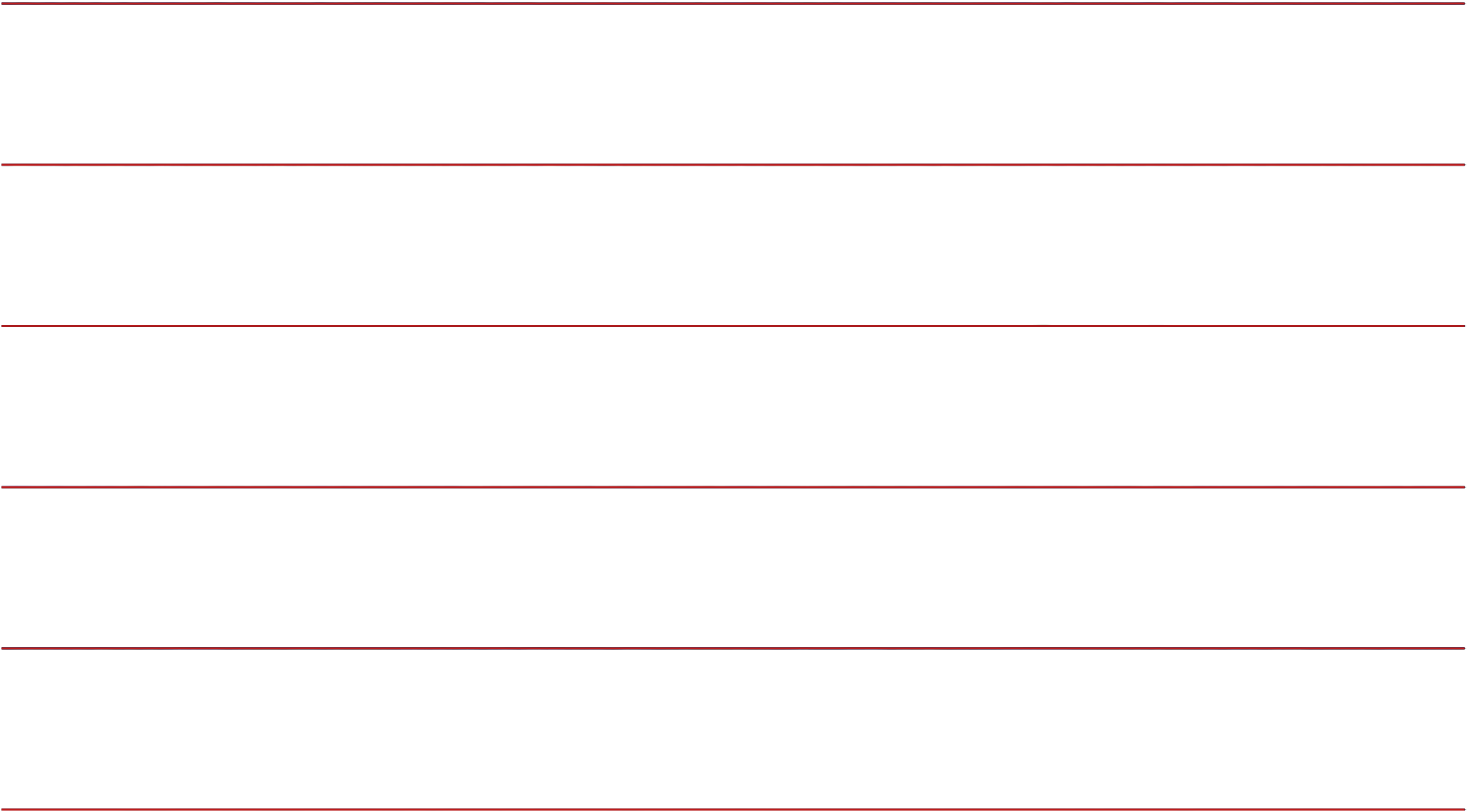

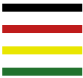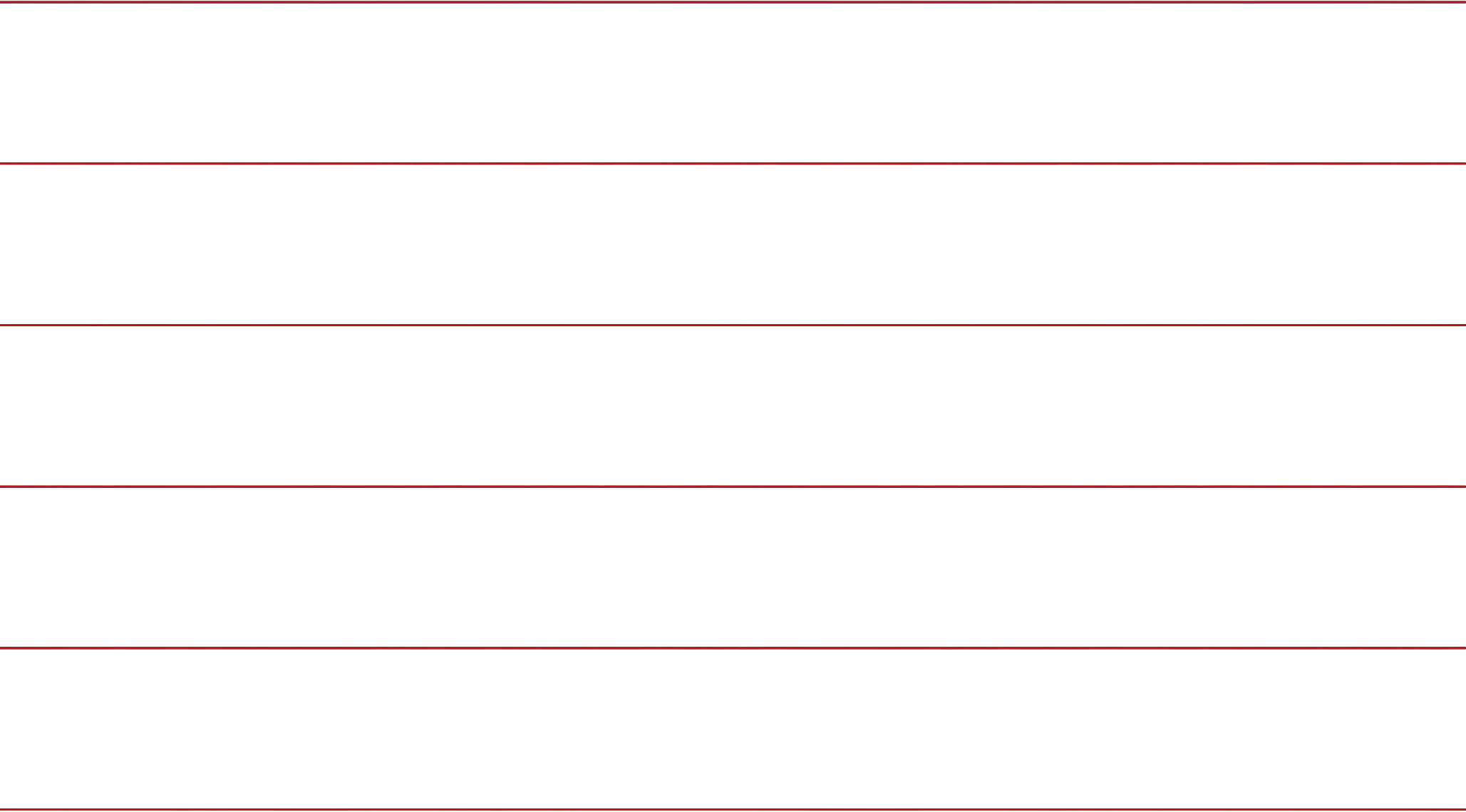

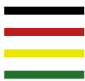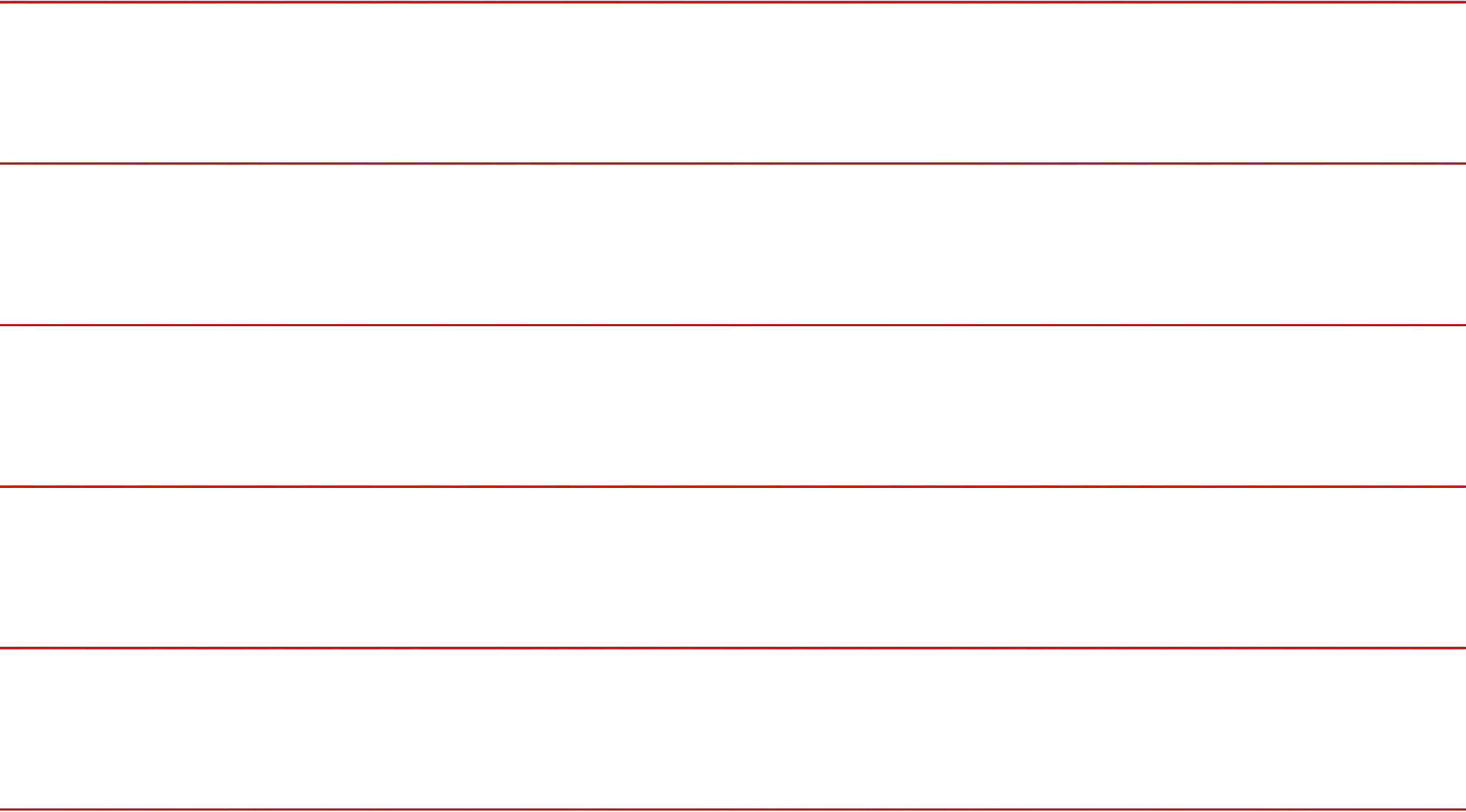

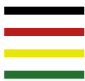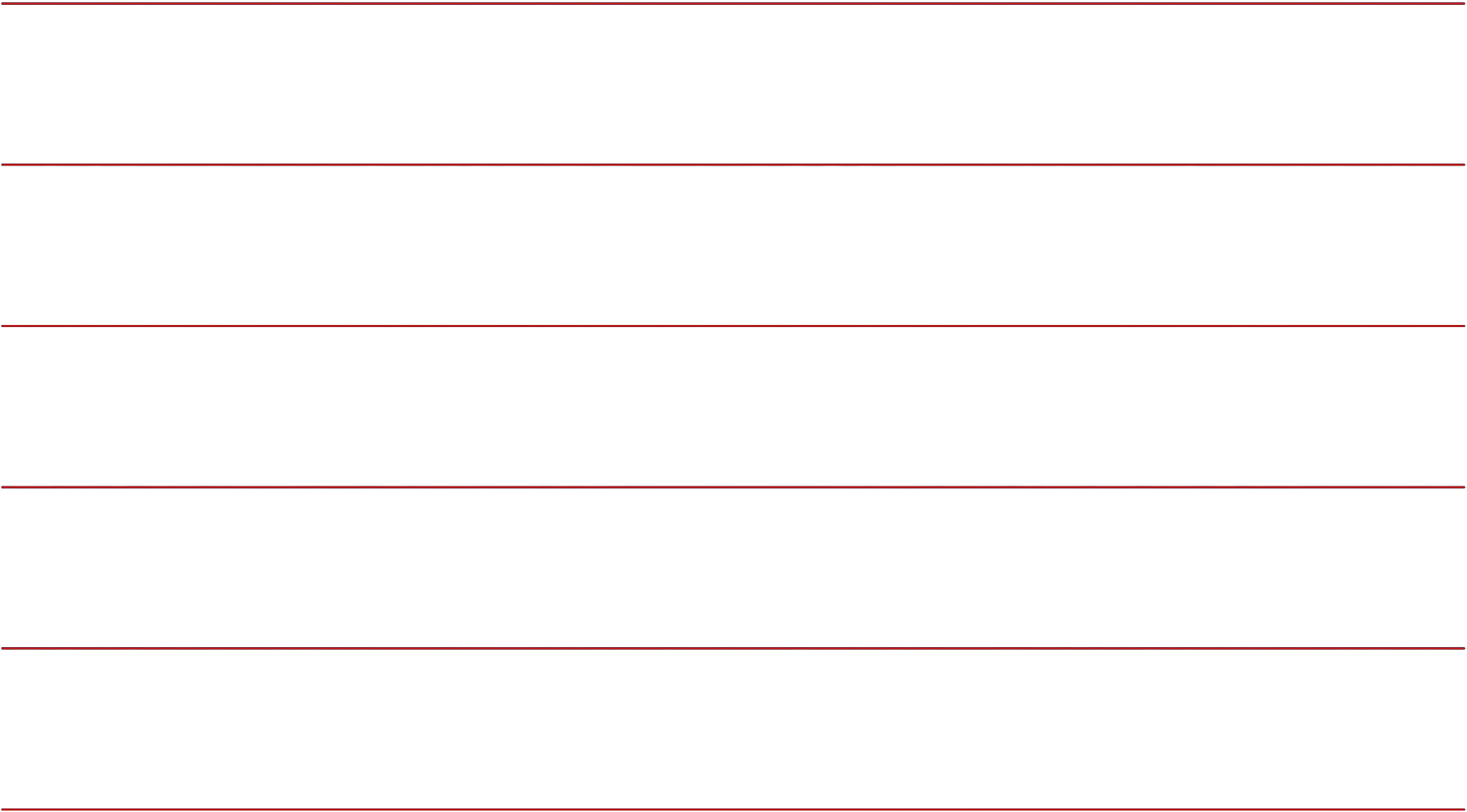

Sequence: EF73802546

Samples: 30774  
Bases: 218  
Average spacing: 142.0  
Average quality >= 10: 14, 20: 14, 30: 182

Quality: 0 - 9 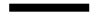  
10 - 19 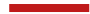  
20 - 29 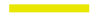  
>= 30 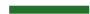

Page: 6 / 6  
27.05.2025

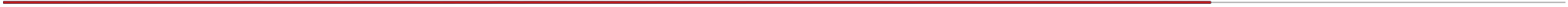

Supplement: Supplementary file 4 — Source data [file 41467_2026_68558_MOESM4_ESM.zip › Source data/Sanger-sequencing data/Suppl.Fig1g/BJ late Peg3.pdf]

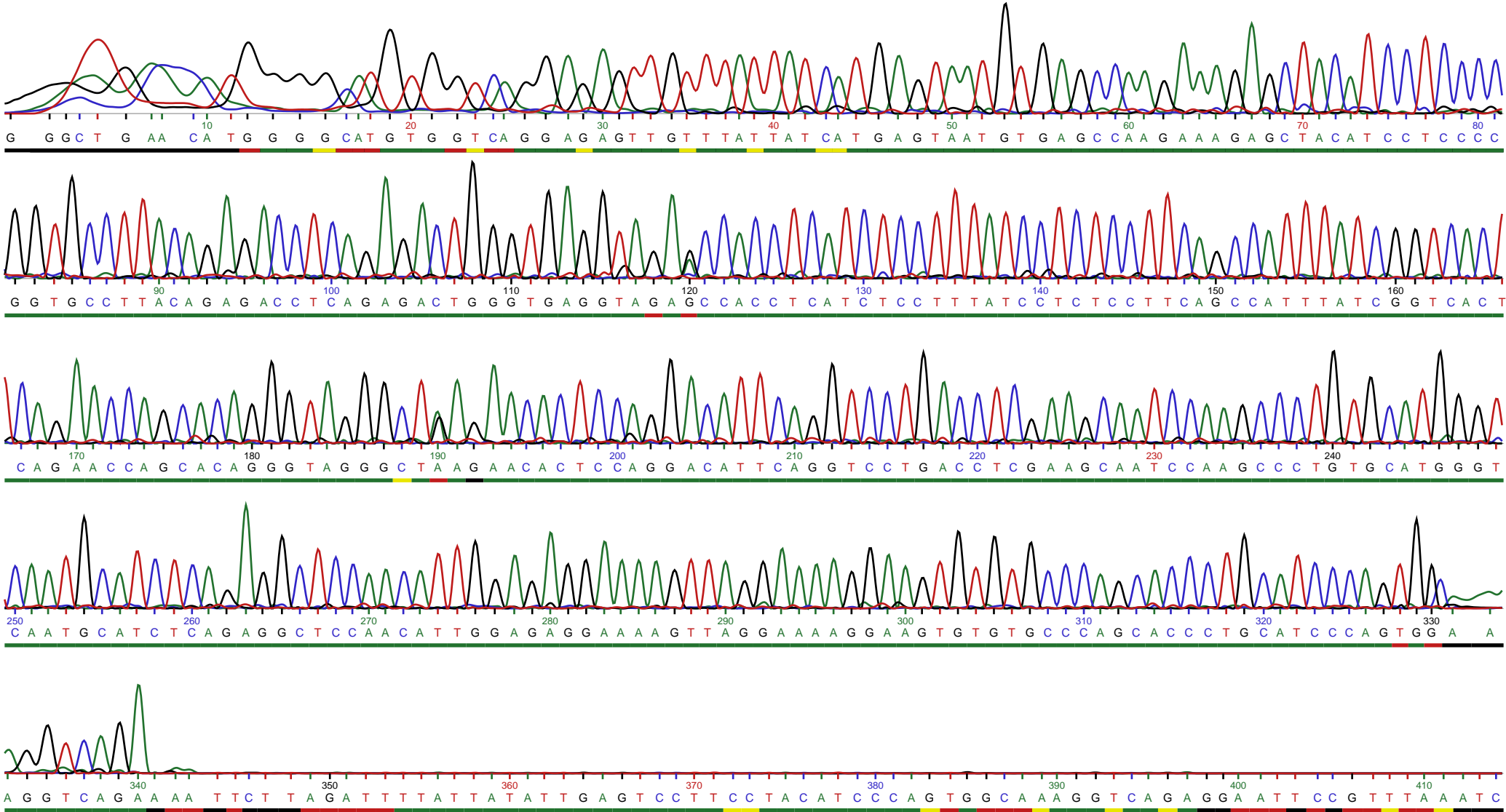

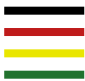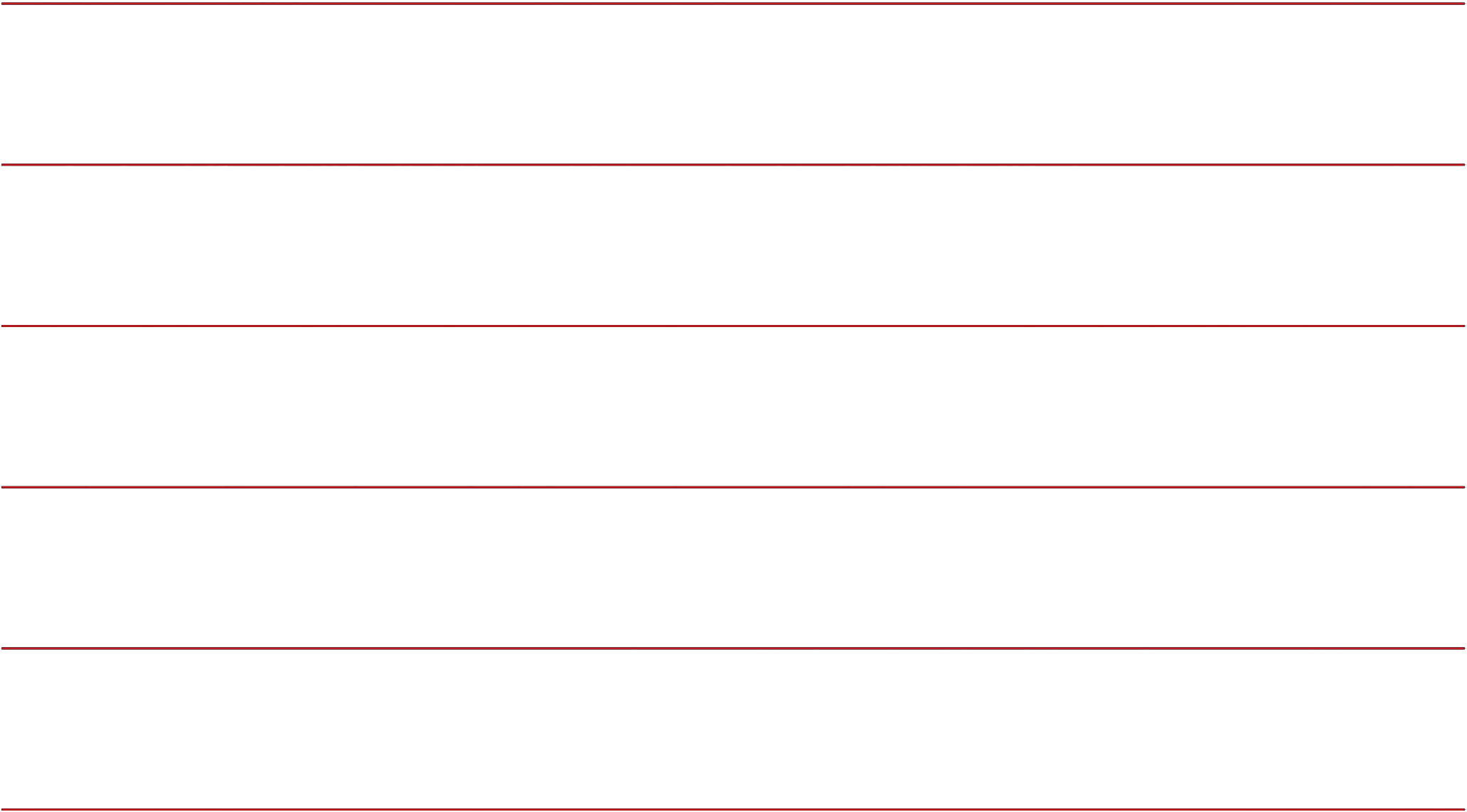

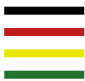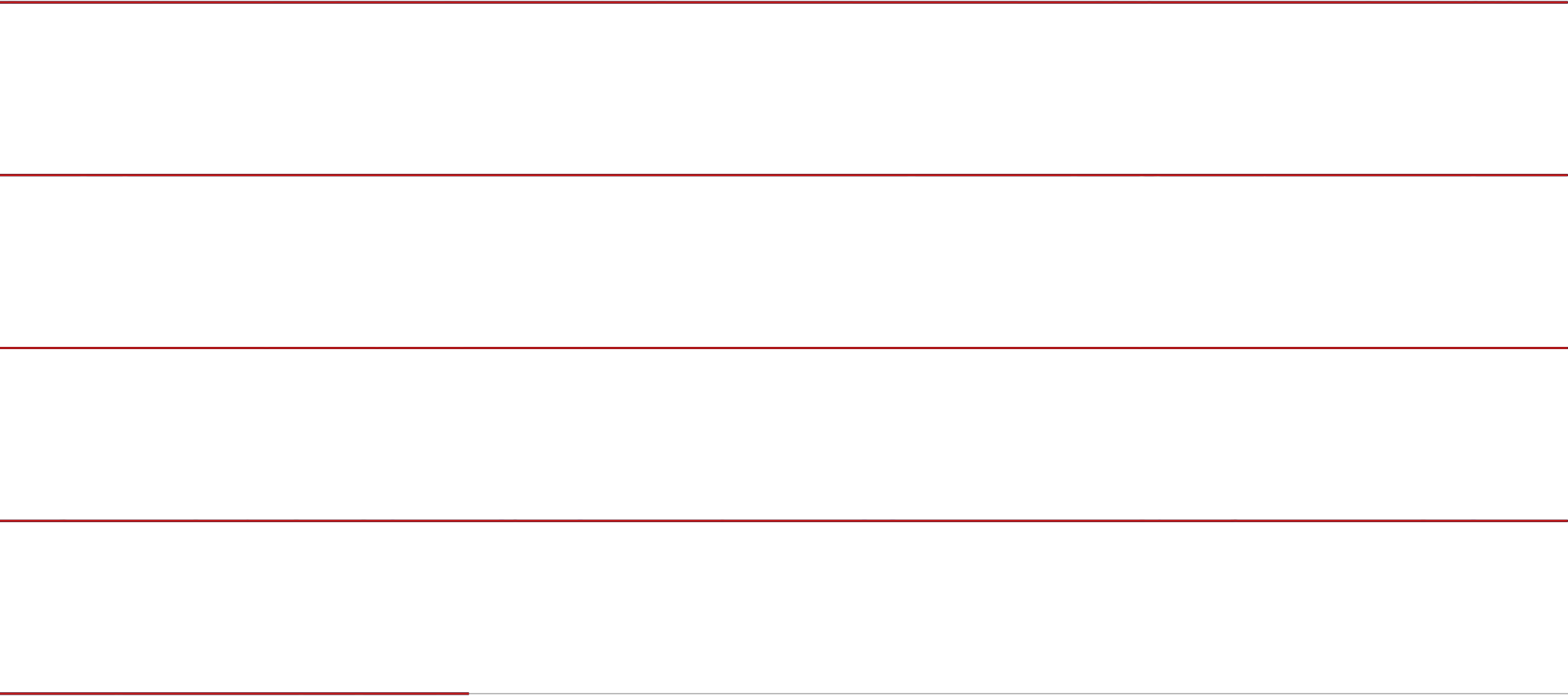

Supplement: Supplementary file 4 — Source data [file 41467_2026_68558_MOESM4_ESM.zip › Source data/Sanger-sequencing data/Suppl.Fig1g/BJ late Rasgrf1.pdf]

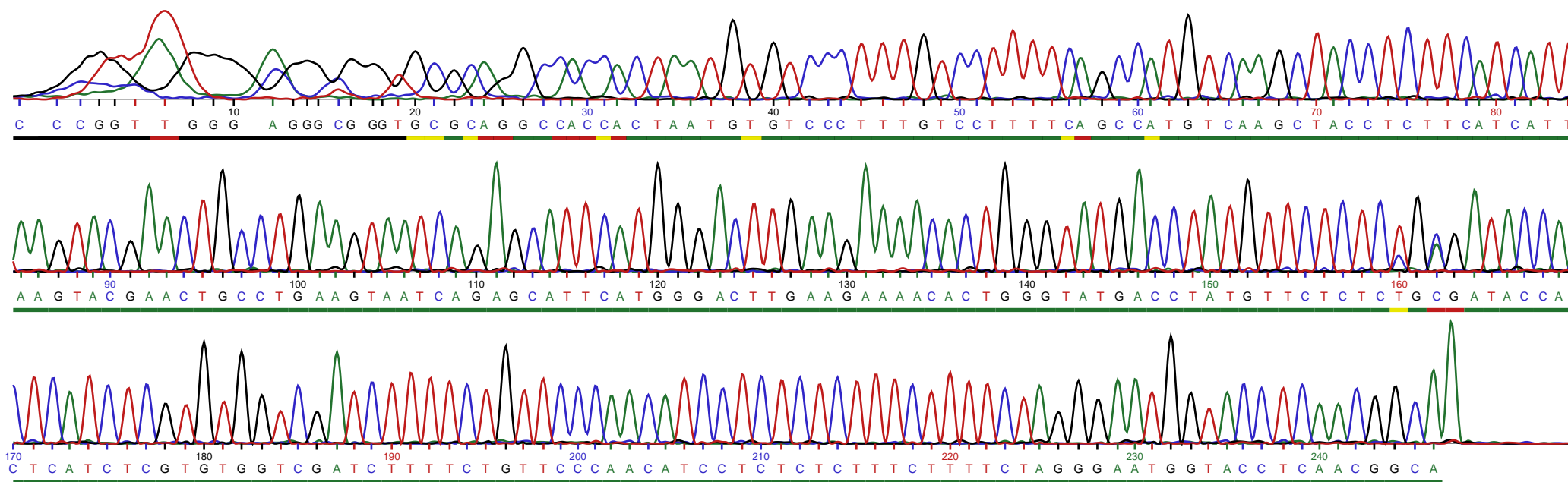

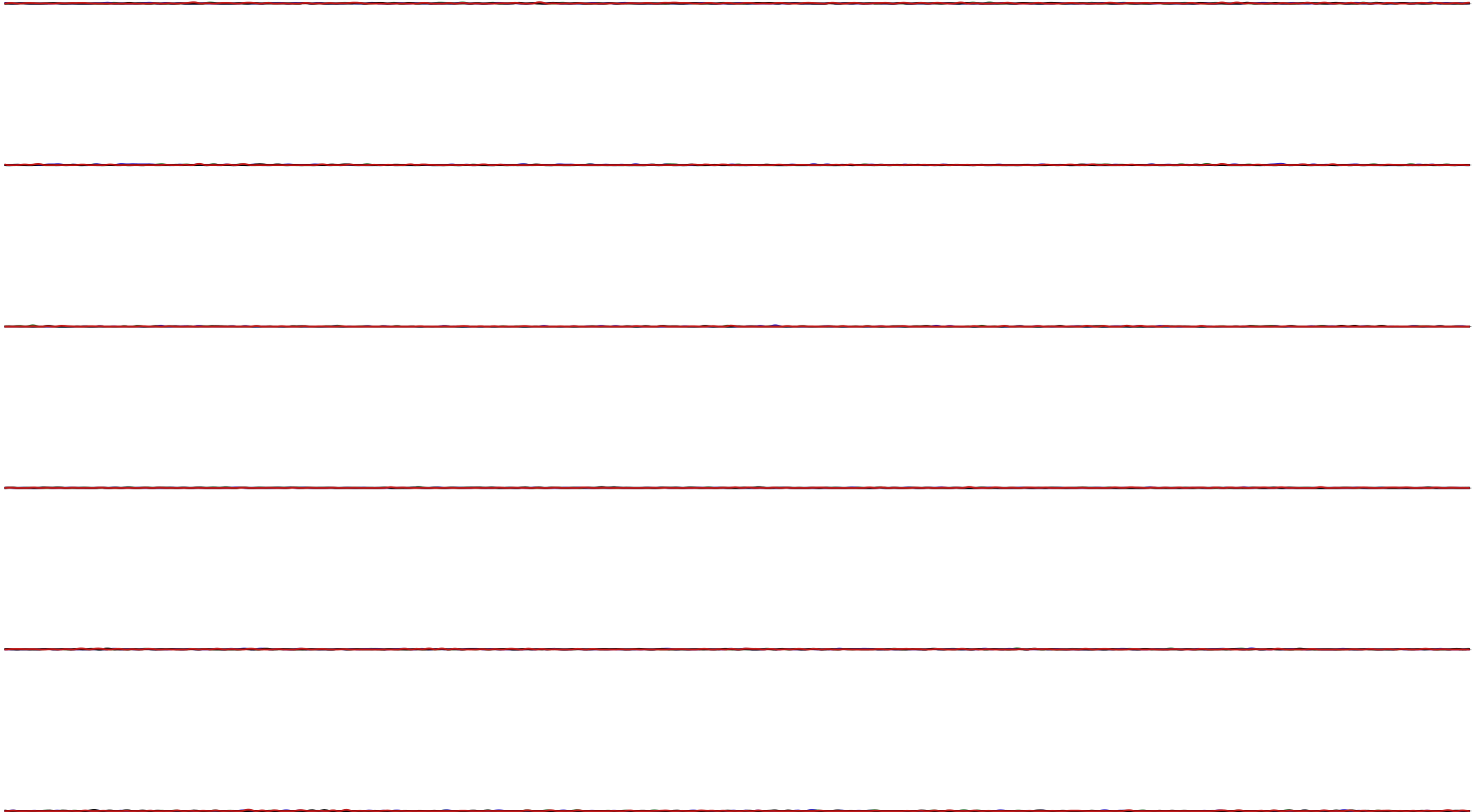

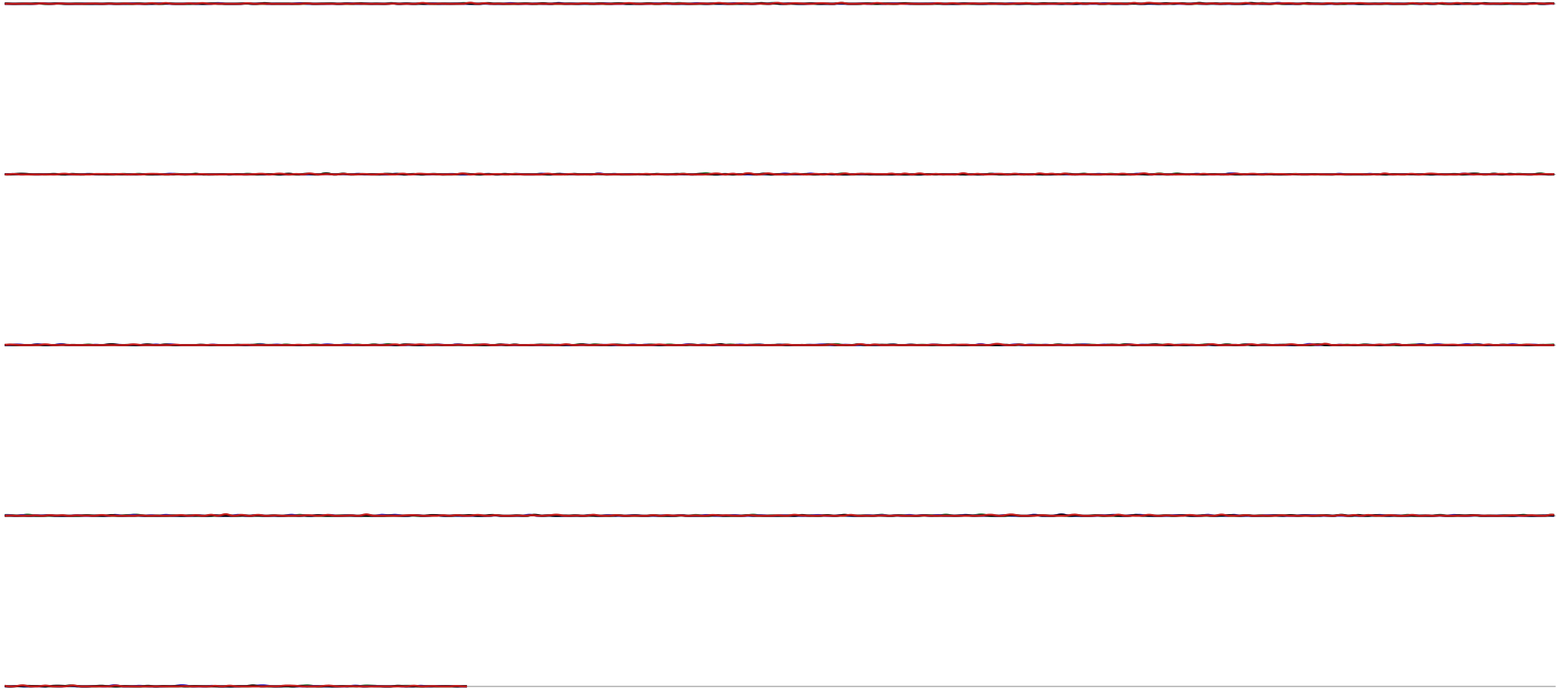

Supplement: Supplementary file 4 — Source data [file 41467_2026_68558_MOESM4_ESM.zip › Source data/Sanger-sequencing data/Suppl.Fig1g/BJ late Slc38a4.pdf]

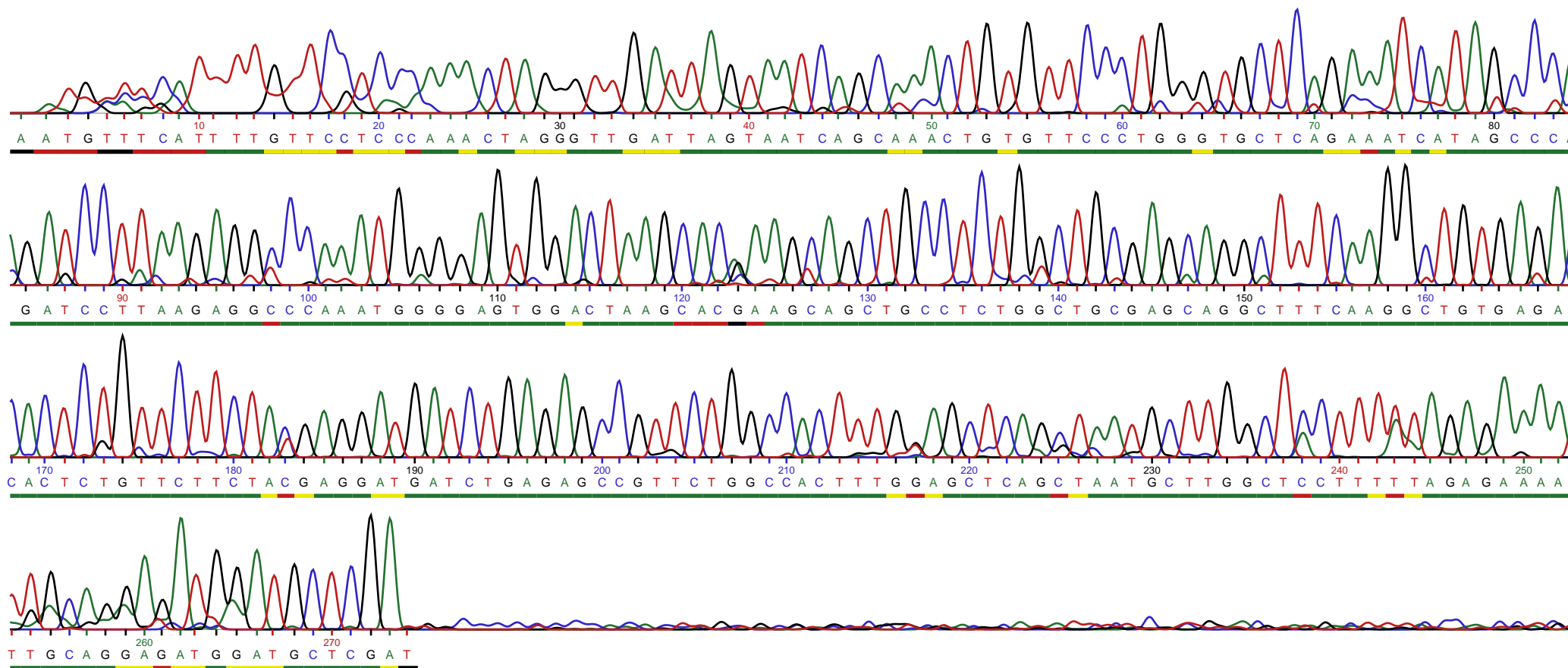

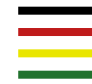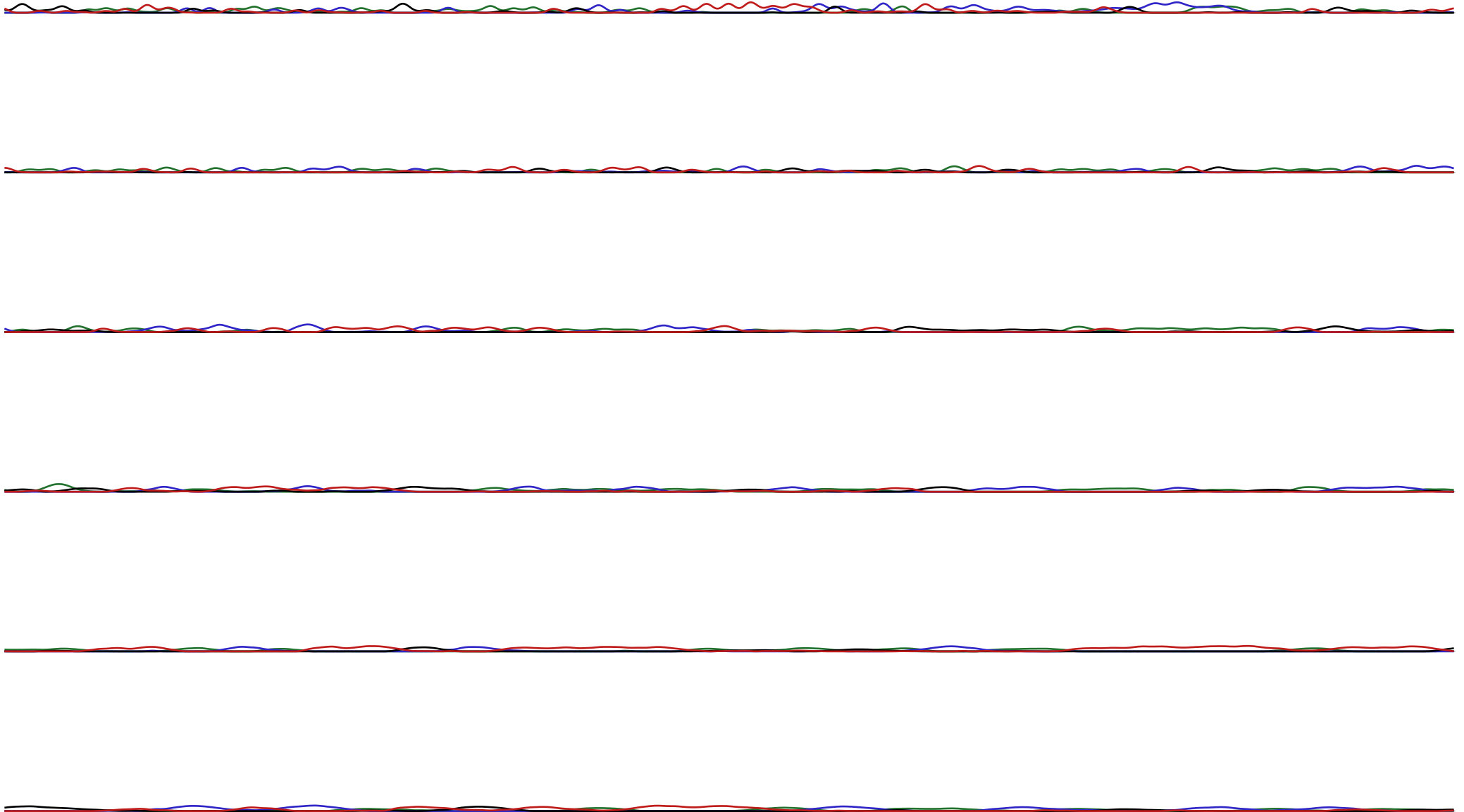

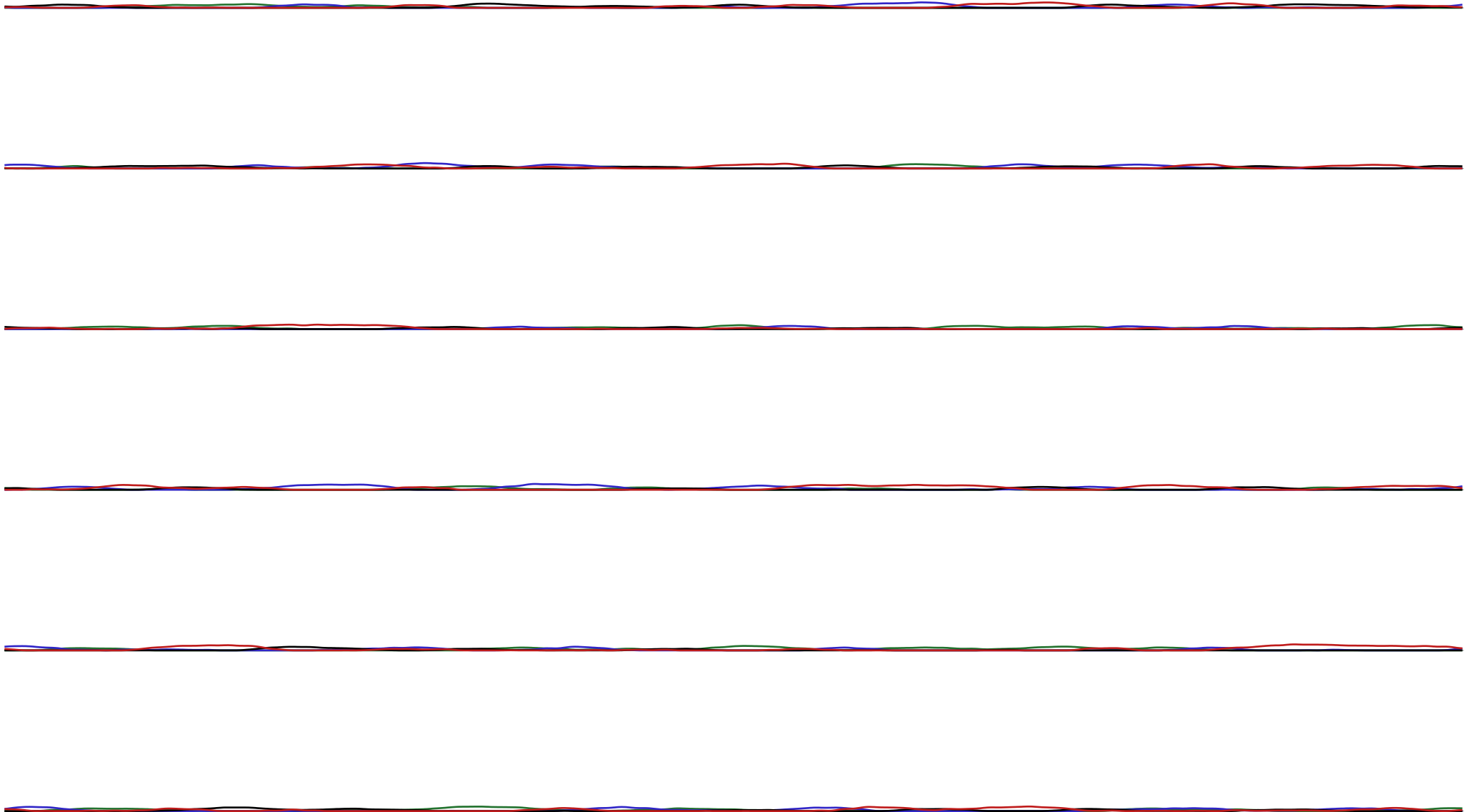

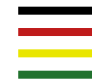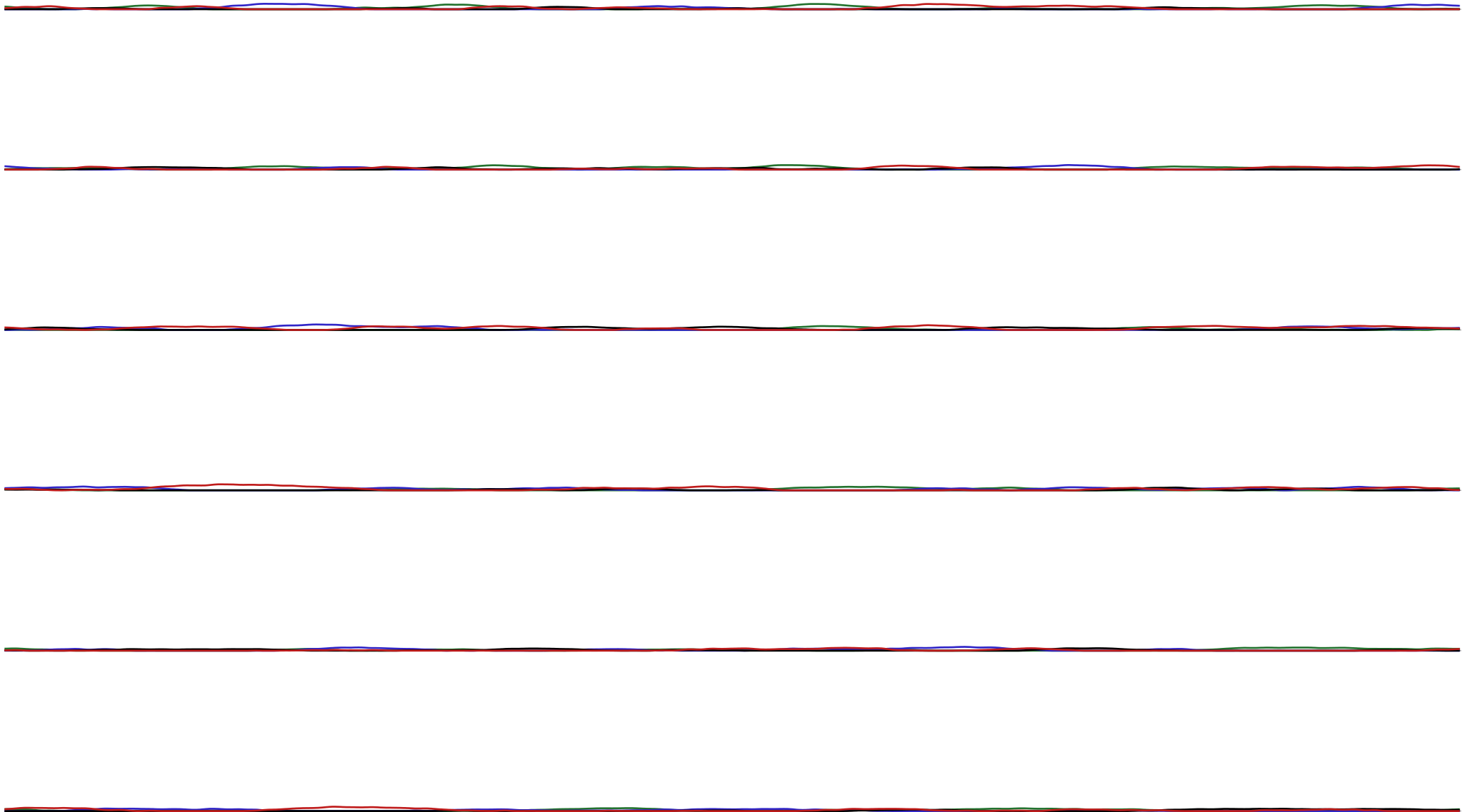

Sequence: EF70930011

Samples: 26780  
Bases: 275  
Average spacing: 98.0  
Average quality >= 10: 21, 20: 40, 30: 208

Quality: 0 - 9  
10 - 19  
20 - 29  
>= 30

Page: 5 / 5  
12.01.2024

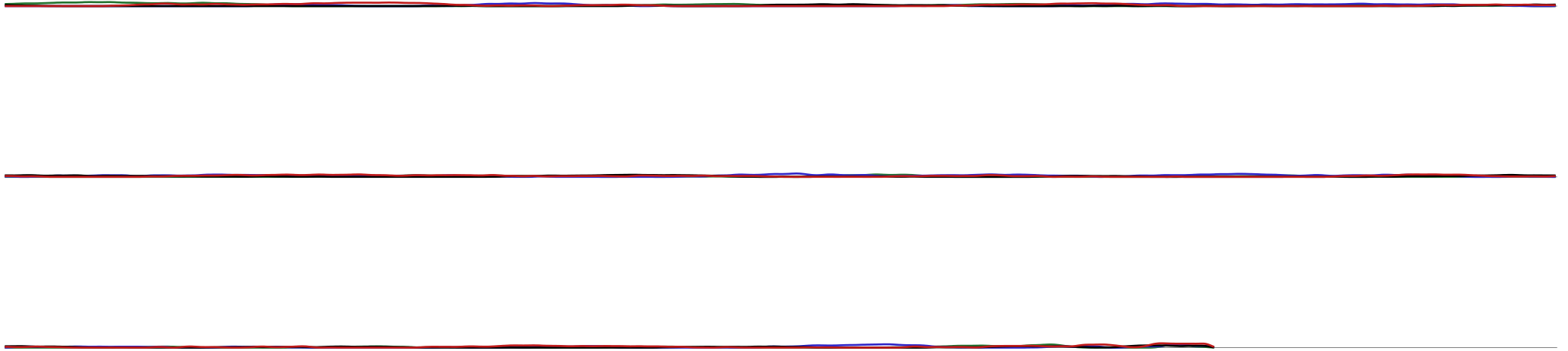

Supplement: Supplementary file 4 — Source data [file 41467_2026_68558_MOESM4_ESM.zip › Source data/Sanger-sequencing data/Suppl.Fig2g/Early-S-Meg3.pdf]

Sequence: EF70929848

Samples: 21041  
Bases: 1398  
Average spacing: 16.0  
Average quality >= 10: 489, 20: 355, 30: 227

Quality: 0 - 9  
10 - 19  
20 - 29  
≥ 30

Page: 1 / 4  
12.01.2024

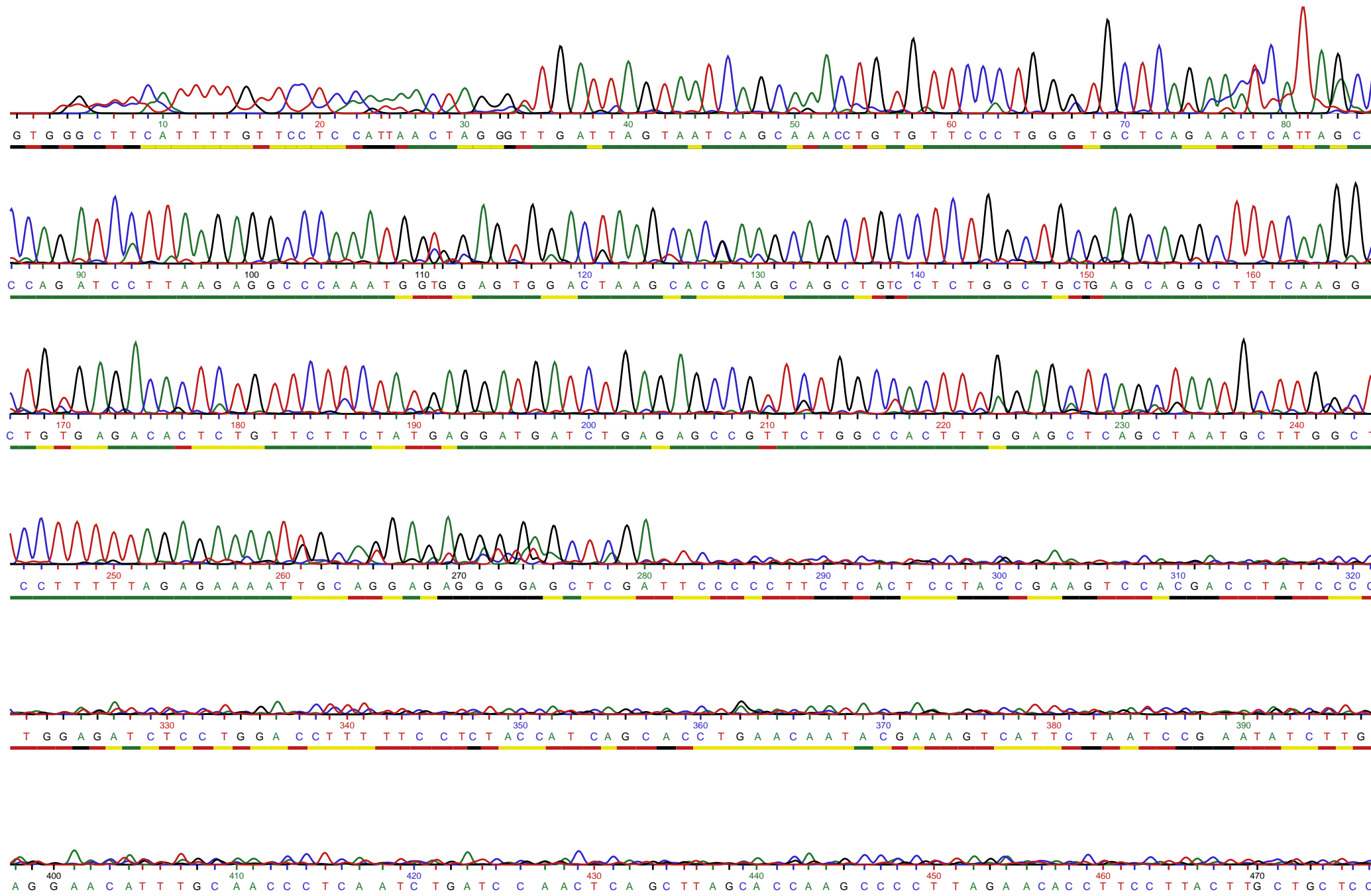

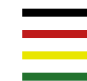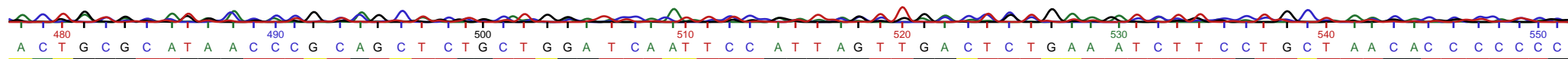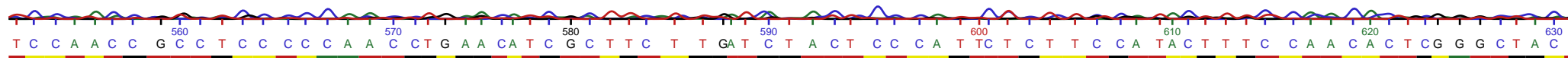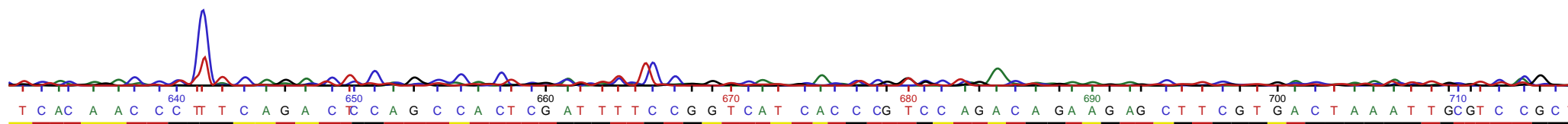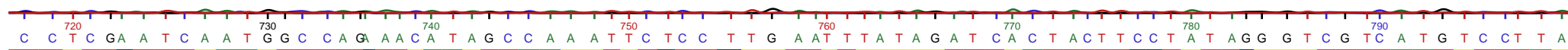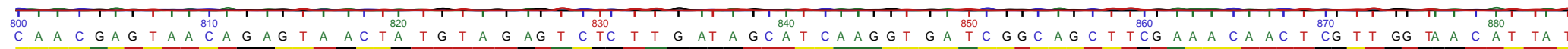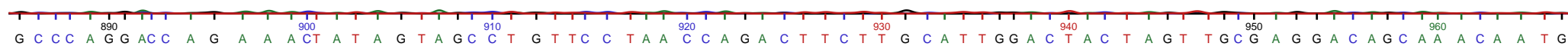

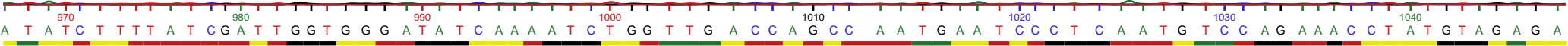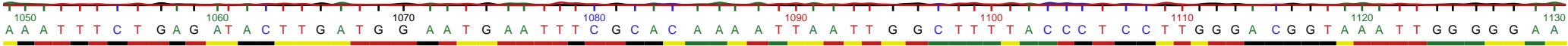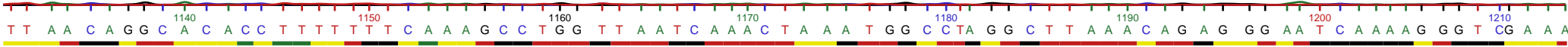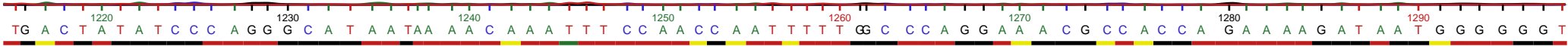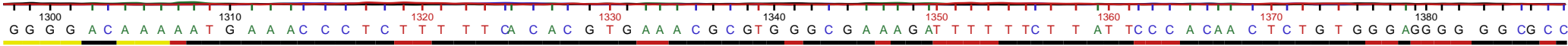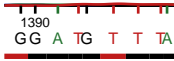

---

---

---

---

Supplement: Supplementary file 4 — Source data [file 41467_2026_68558_MOESM4_ESM.zip › Source data/Sanger-sequencing data/Suppl.Fig2g/Late-G1-Meg3.pdf]

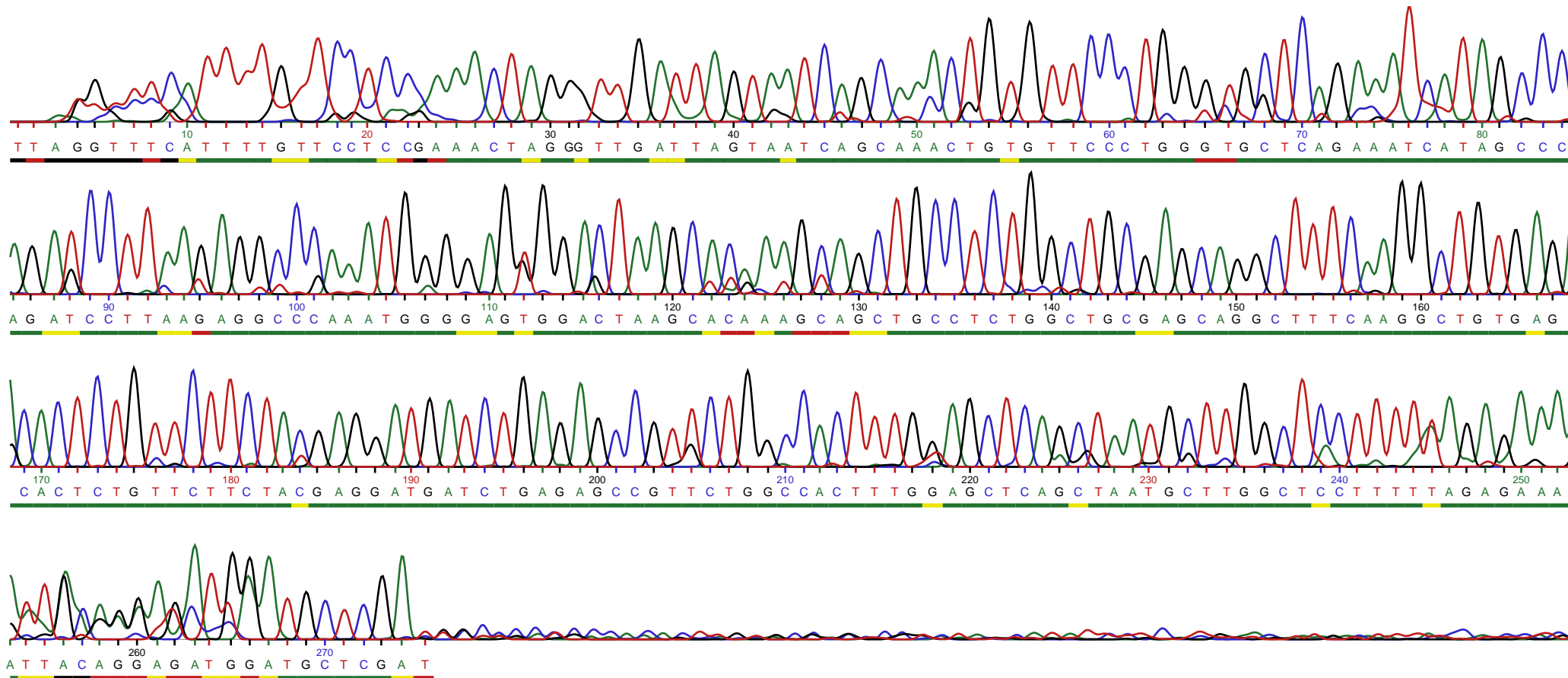

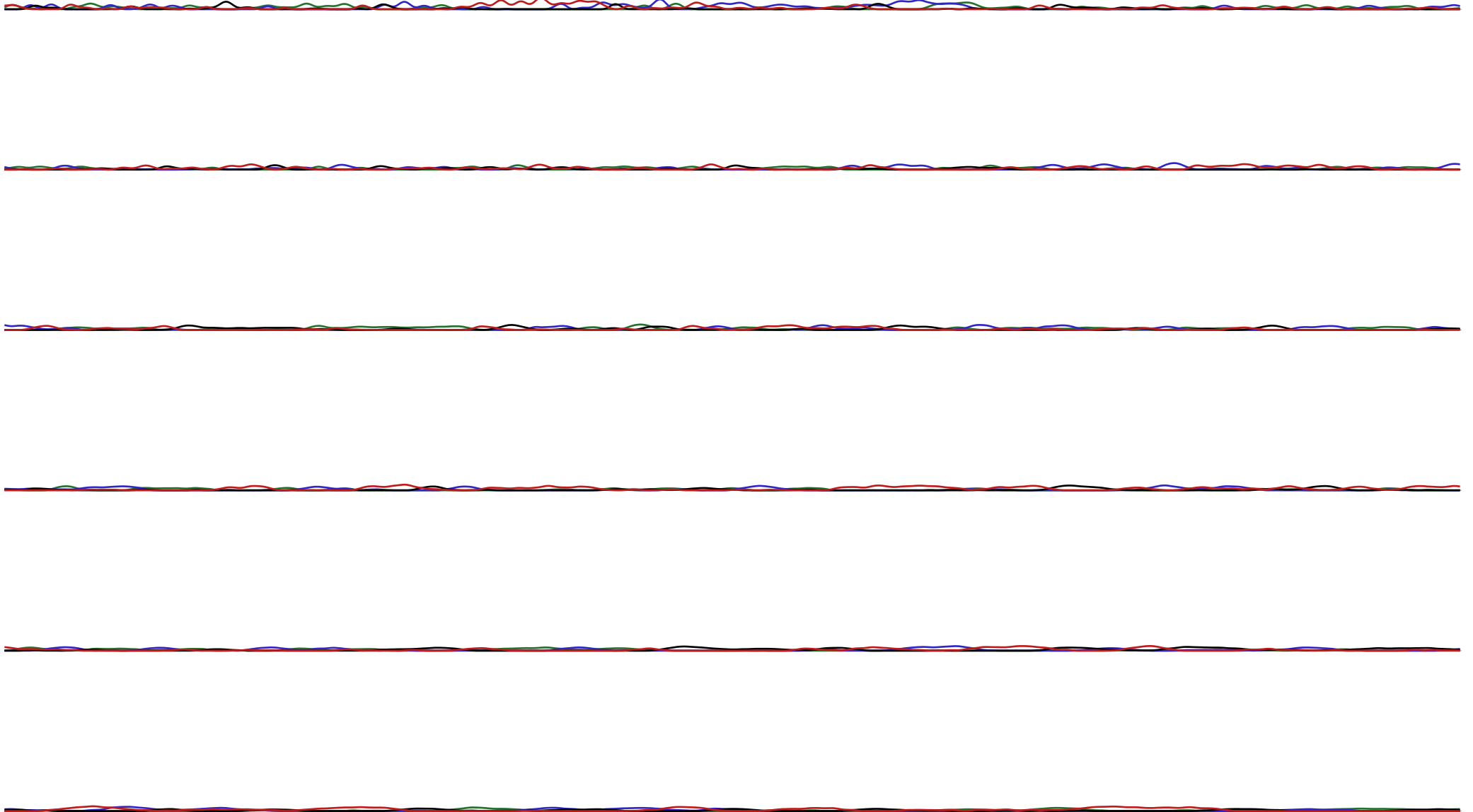

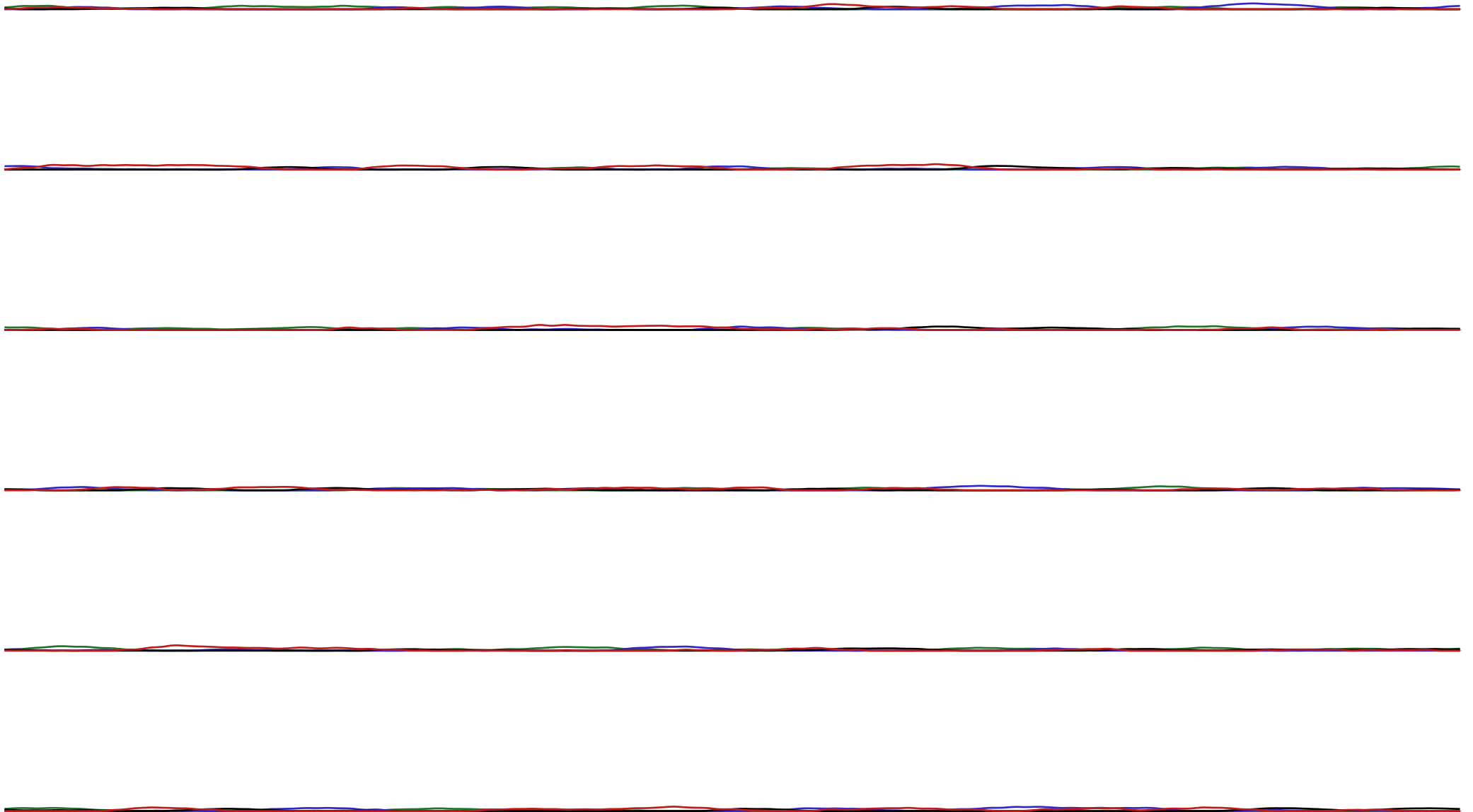

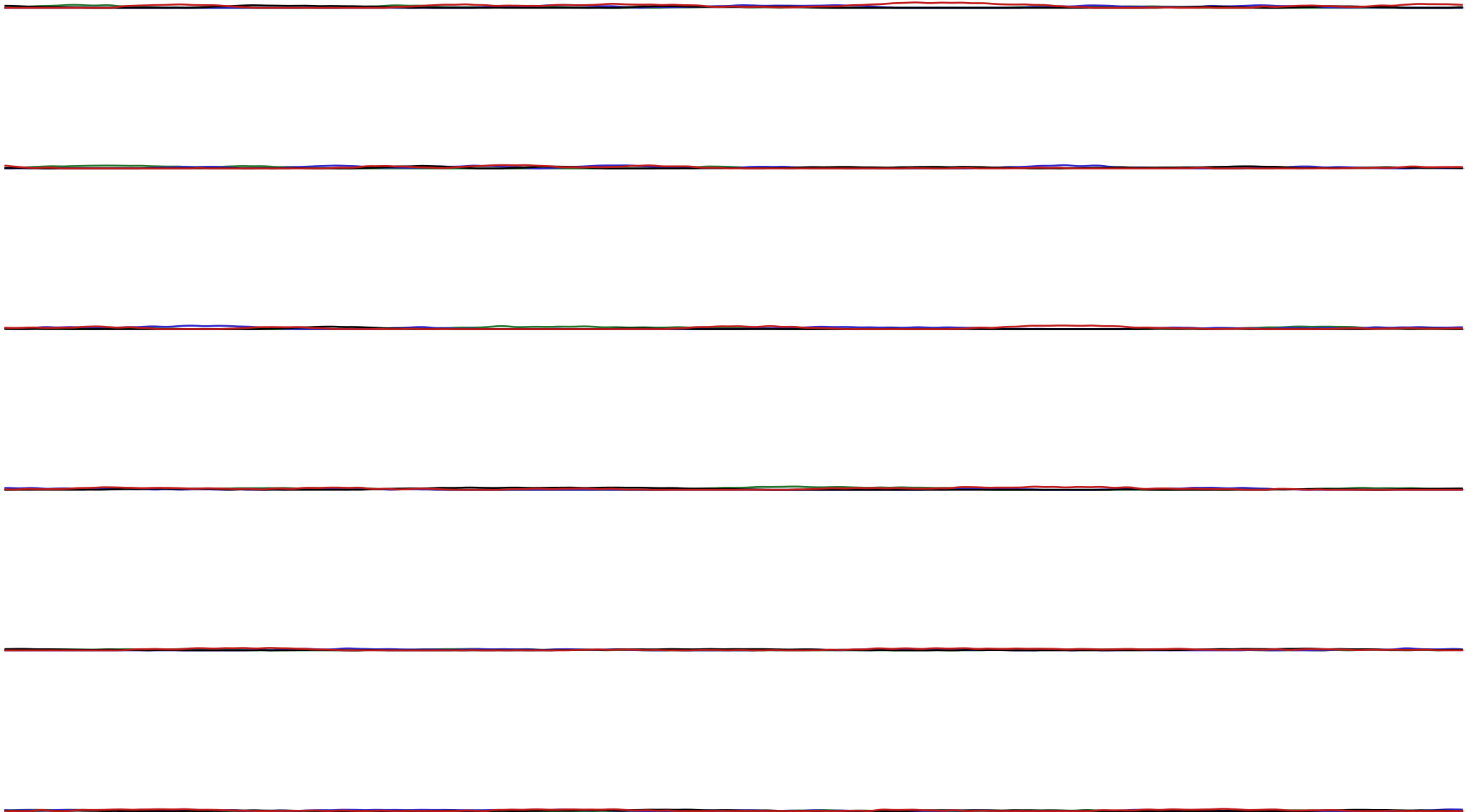

Sequence: EF70930012

Samples: 24265  
Bases: 276  
Average spacing: 88.0  
Average quality >= 10: 20, 20: 36, 30: 210

Quality: 0 - 9  
10 - 19  
20 - 29  
>= 30

Page: 5 / 5  
12.01.2024

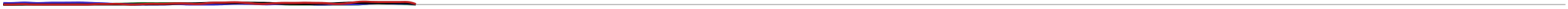

Supplement: Supplementary file 4 — Source data [file 41467_2026_68558_MOESM4_ESM.zip › Source data/Sanger-sequencing data/Suppl.Fig2g/Late-S-Meg3.pdf]

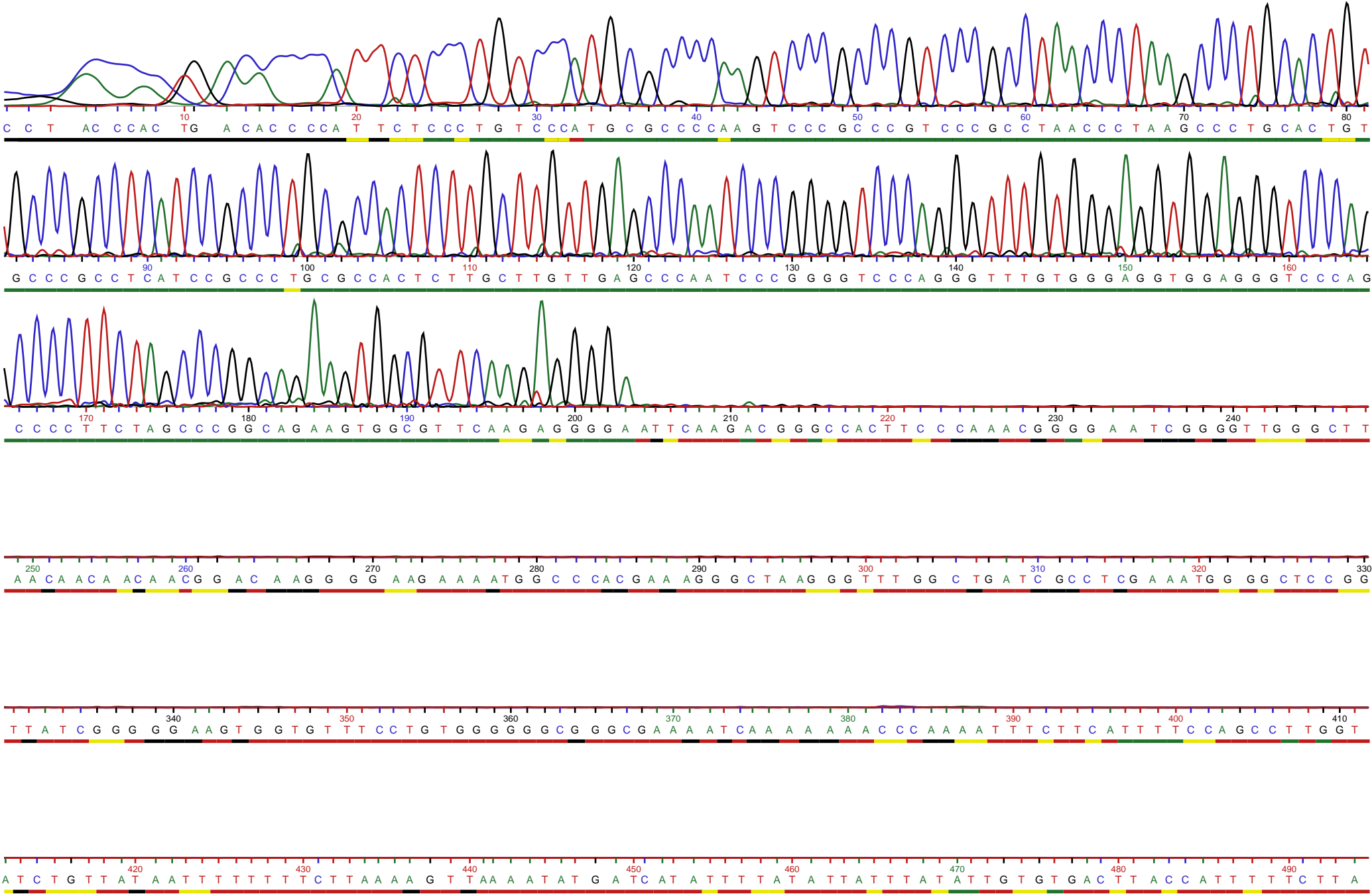

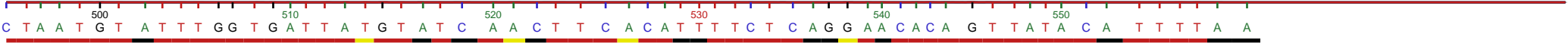

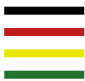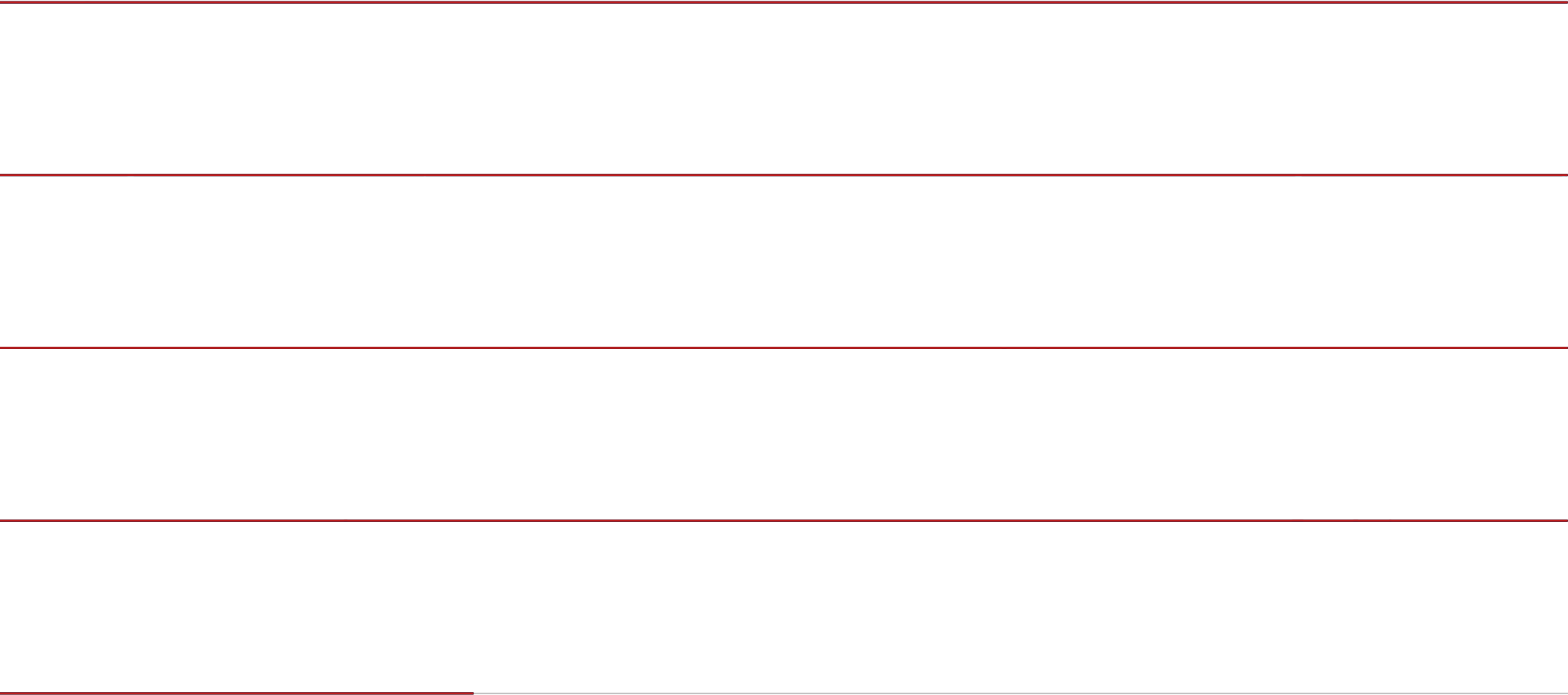

Supplement: Supplementary file 4 — Source data [file 41467_2026_68558_MOESM4_ESM.zip › Source data/Sanger-sequencing data/Suppl.Fig2h/Early-G2-Dlk1.pdf]

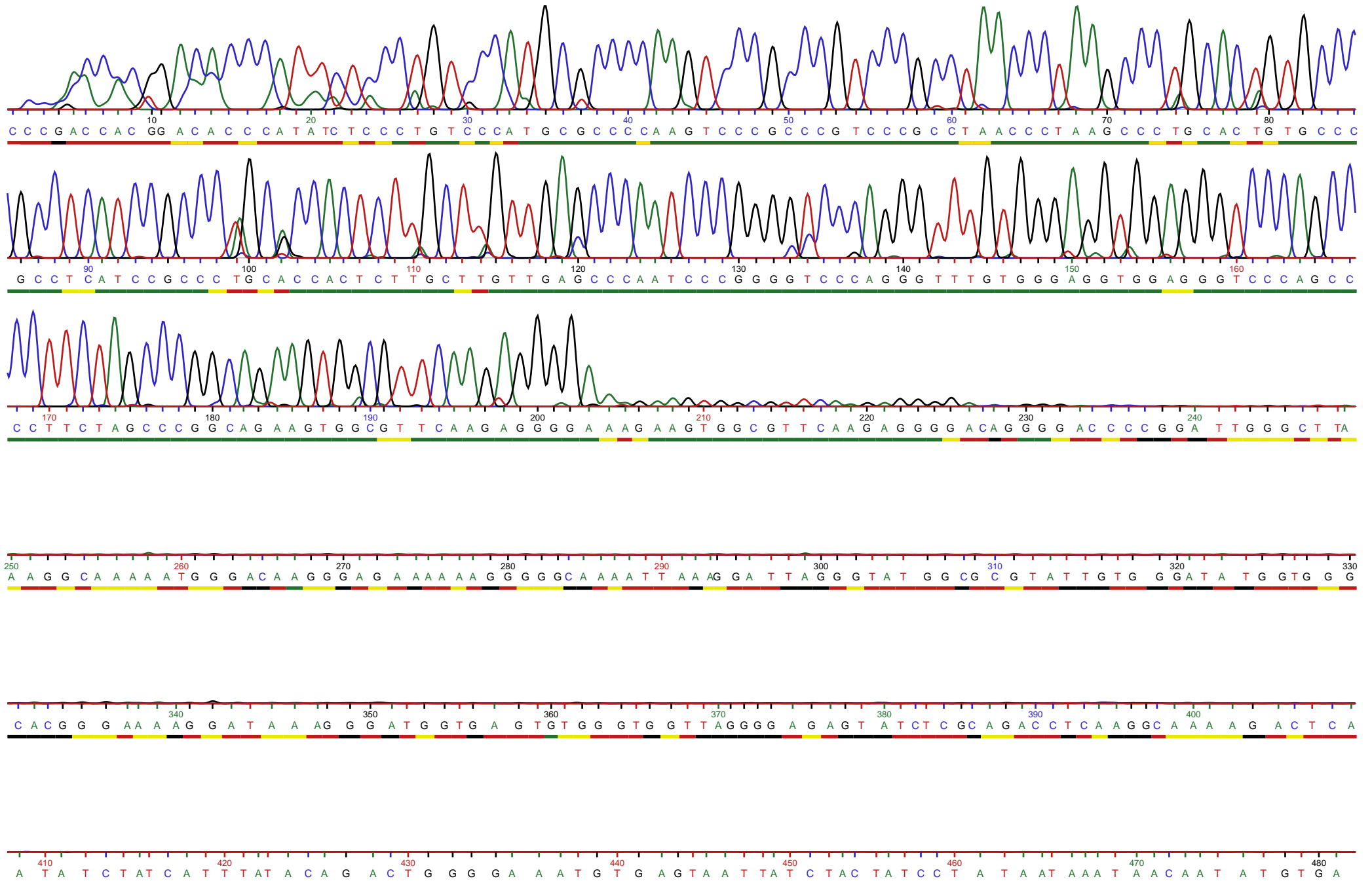

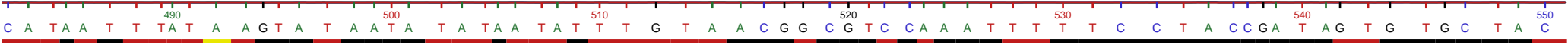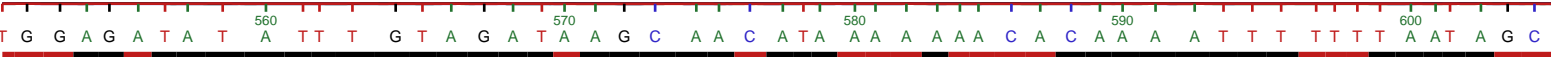

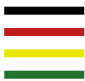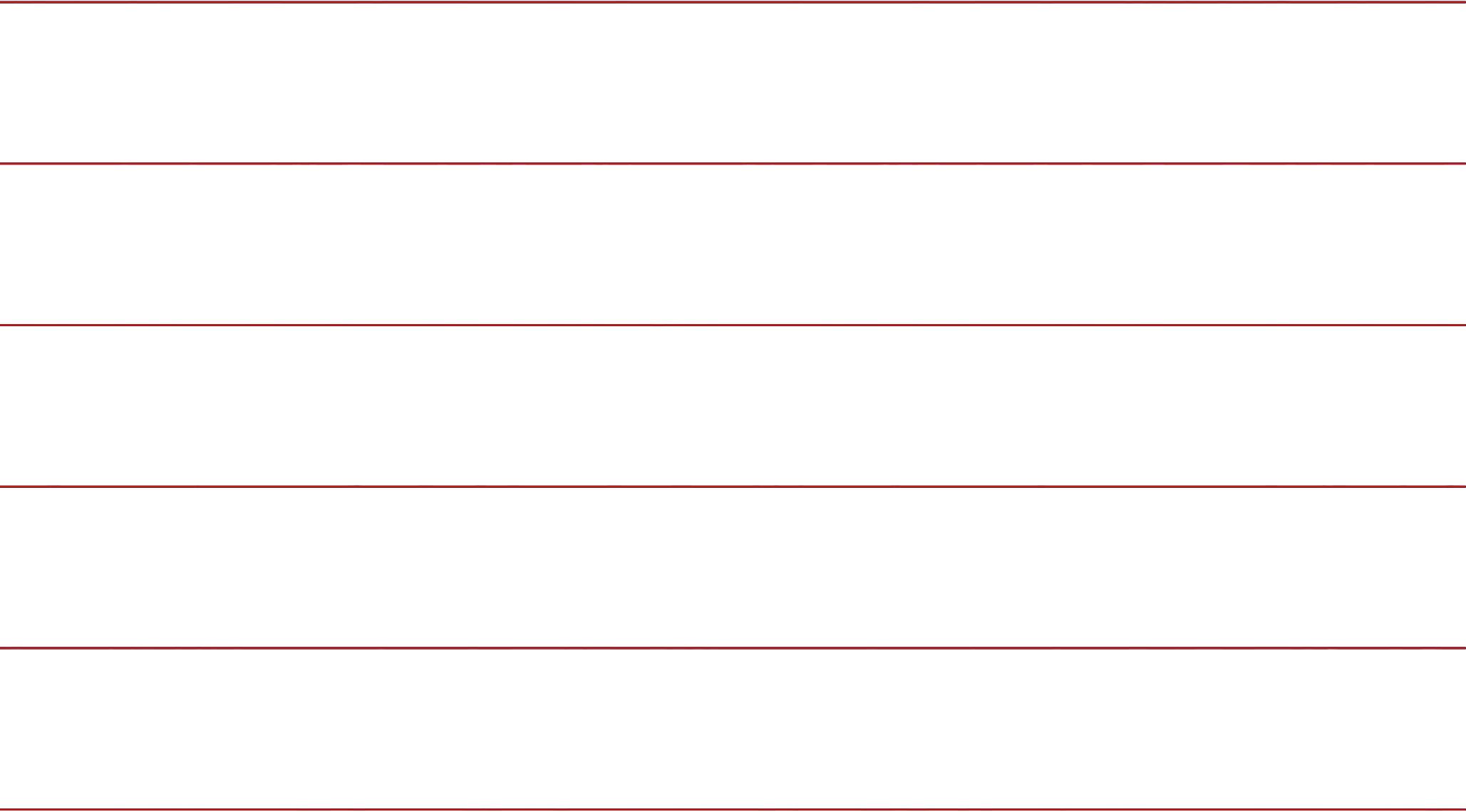

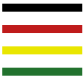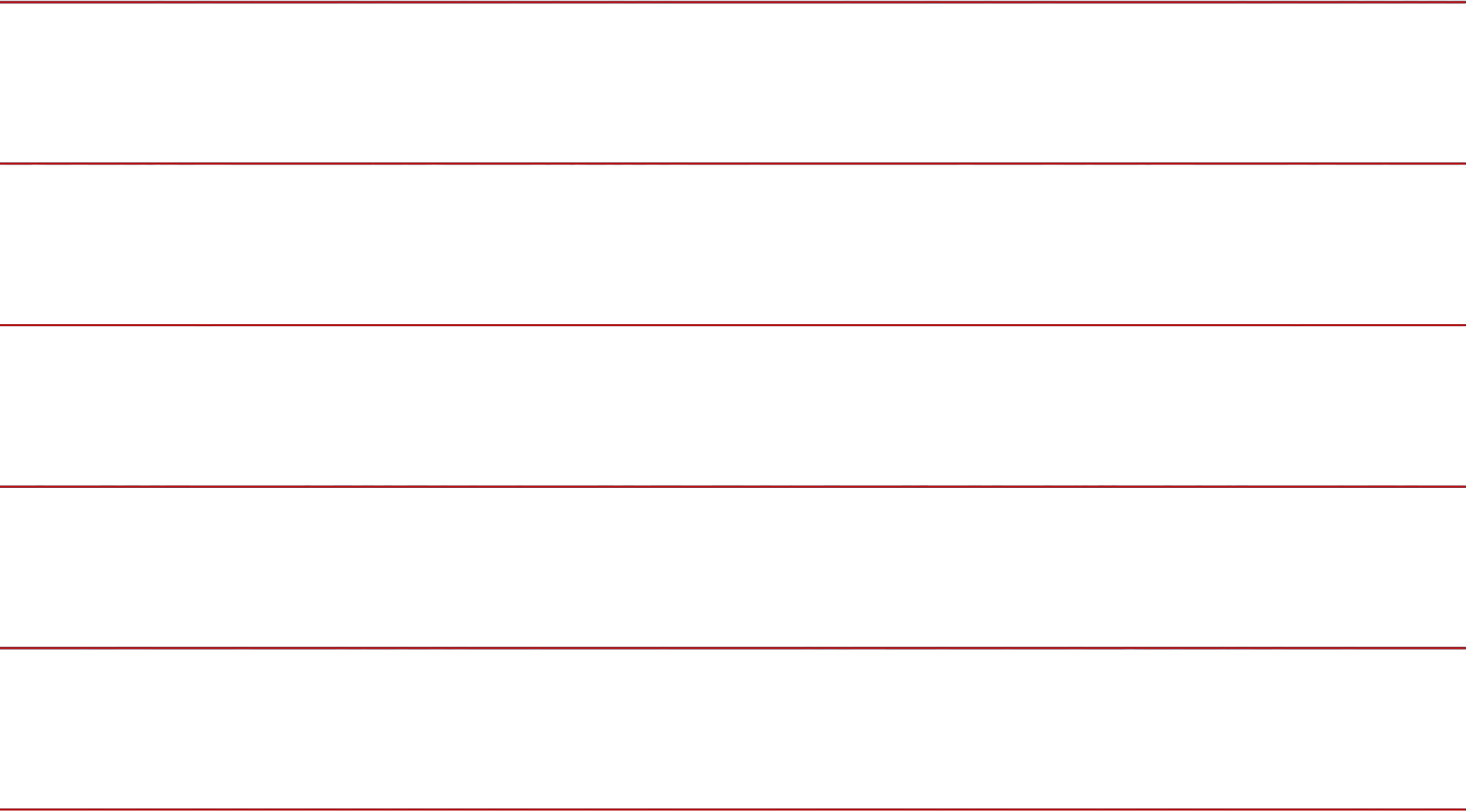

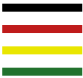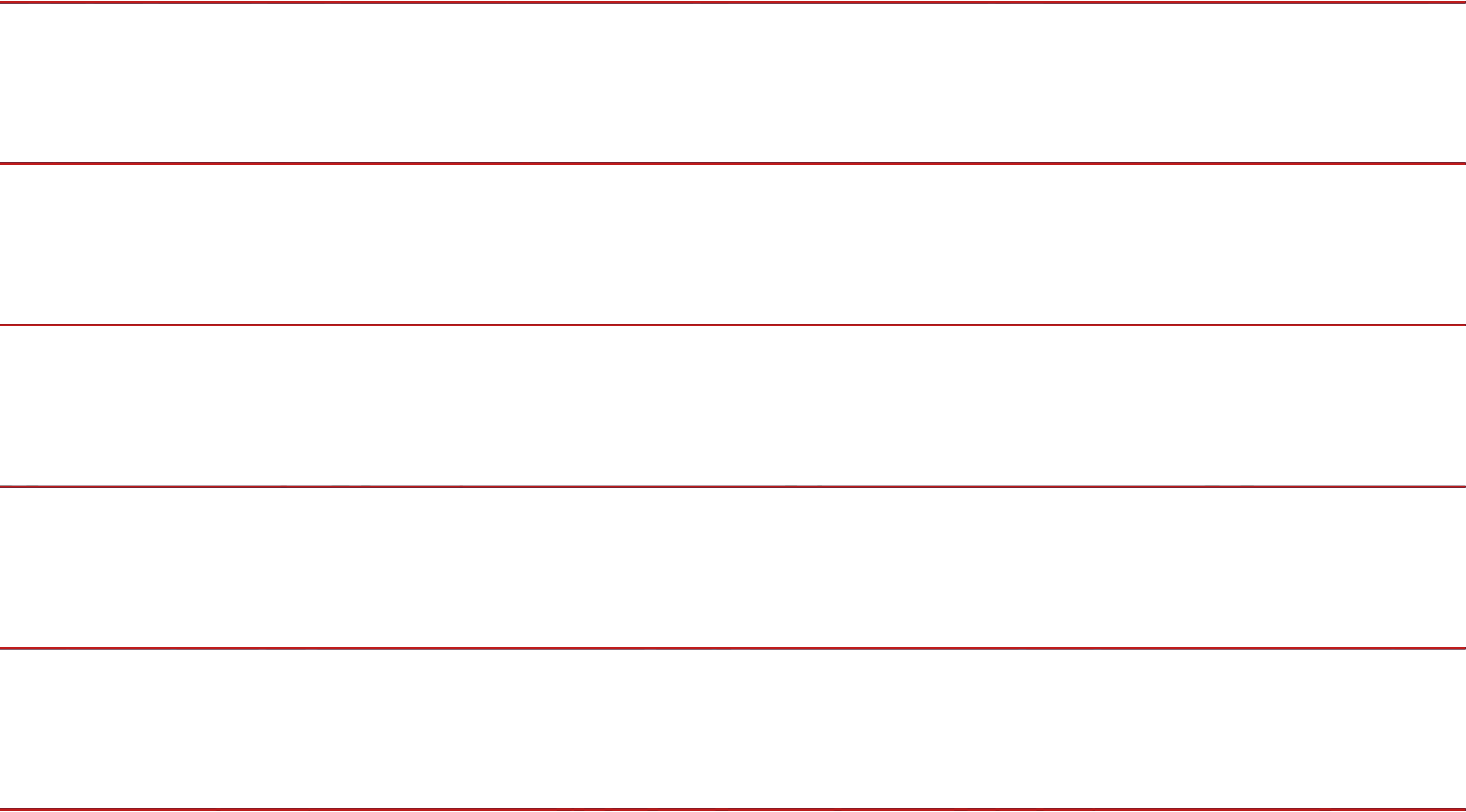

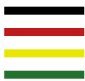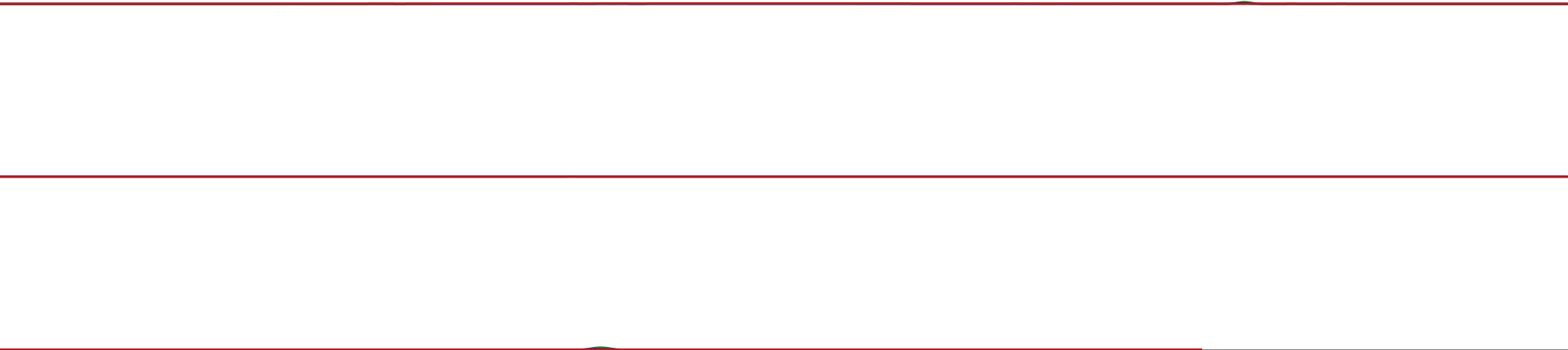

Supplement: Supplementary file 4 — Source data [file 41467_2026_68558_MOESM4_ESM.zip › Source data/Sanger-sequencing data/Suppl.Fig2h/Early-S-Dlk1.pdf]

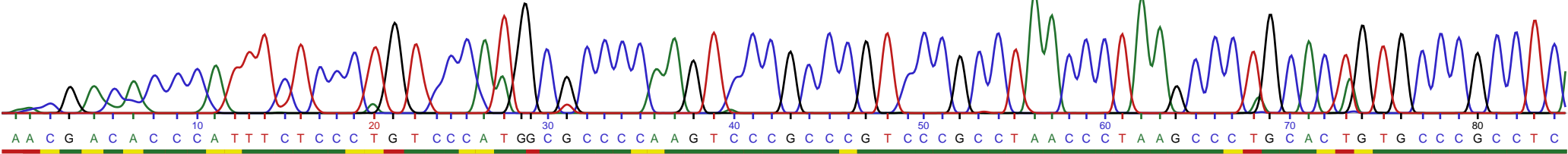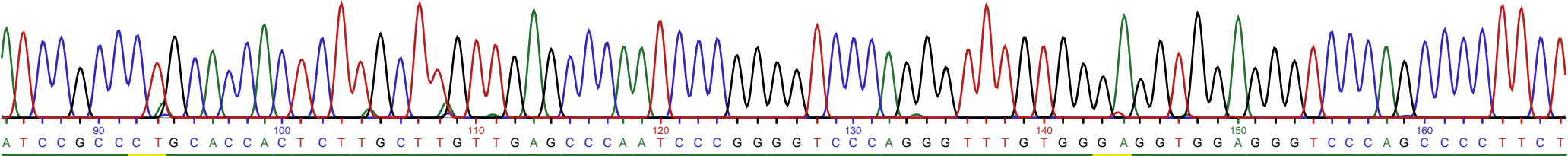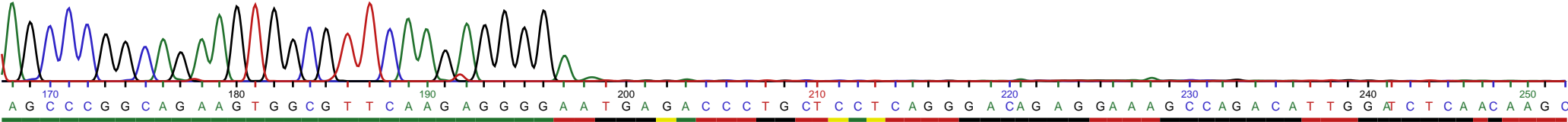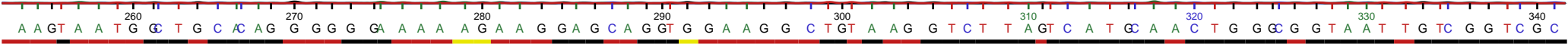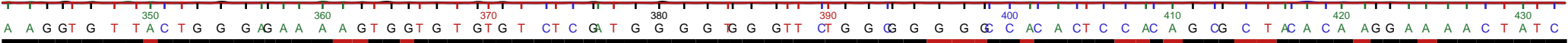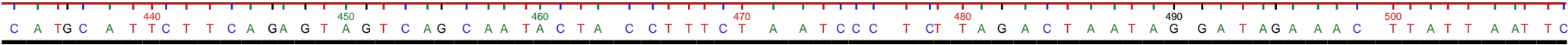

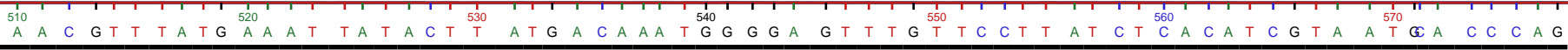

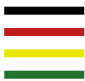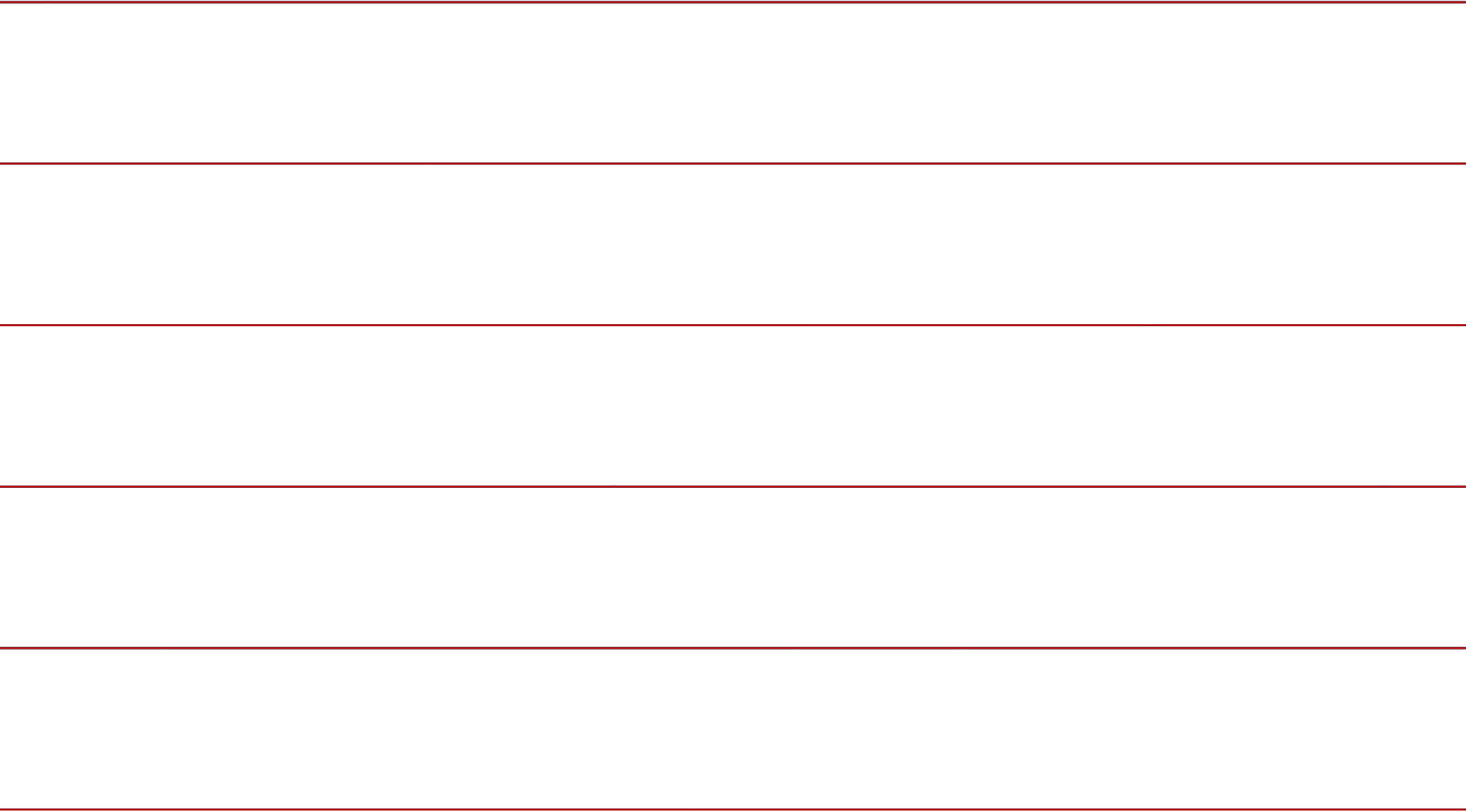

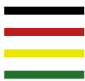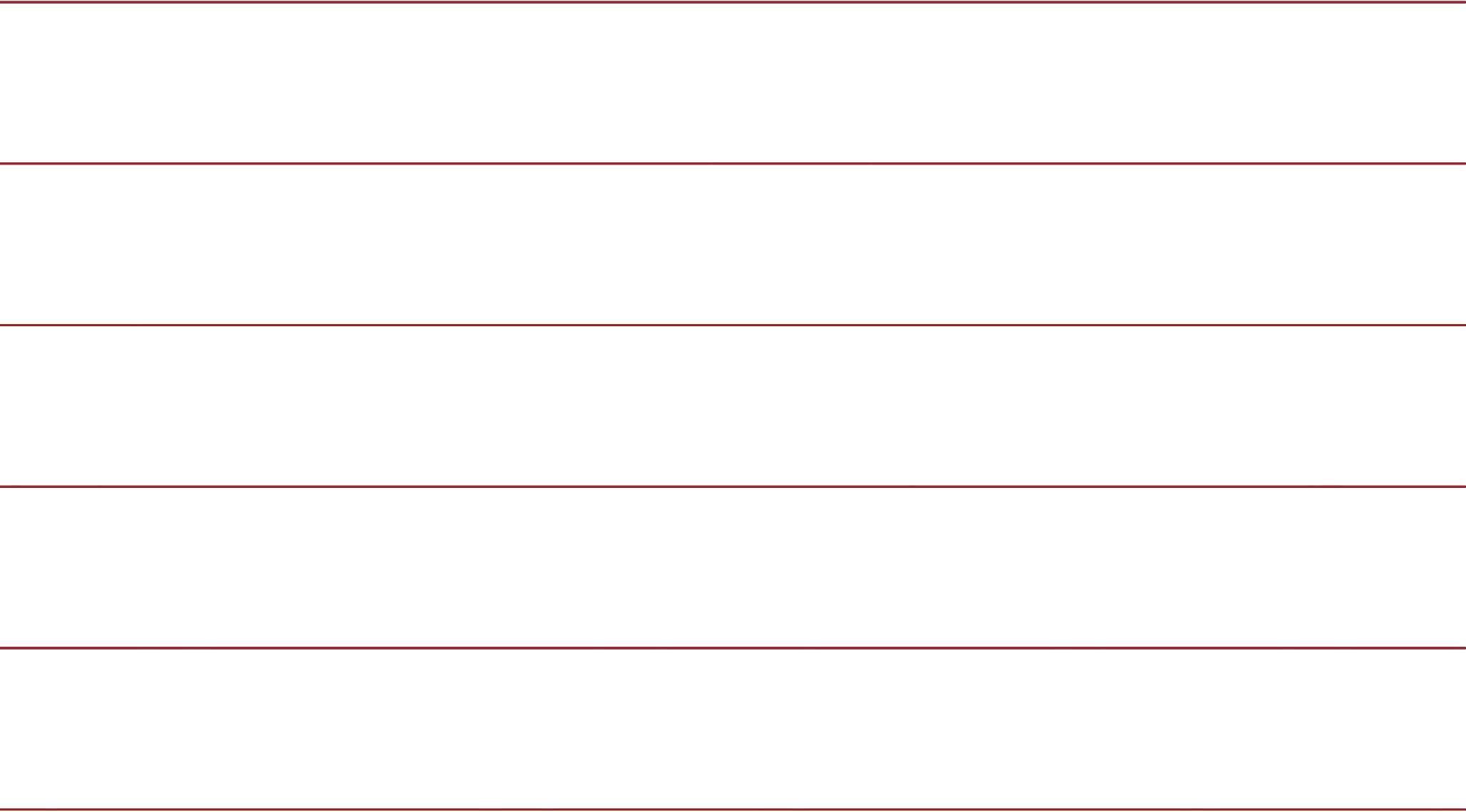

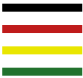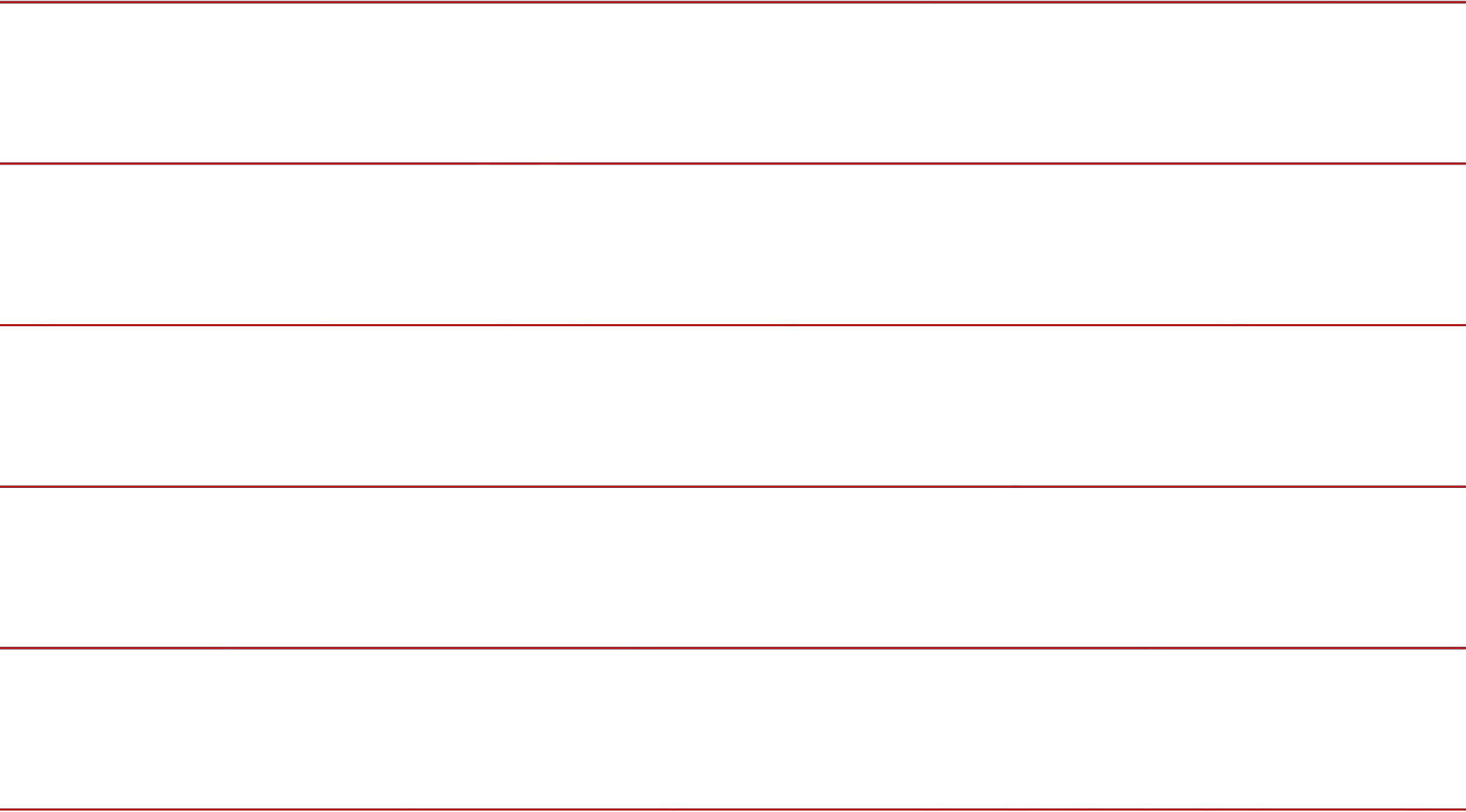

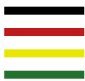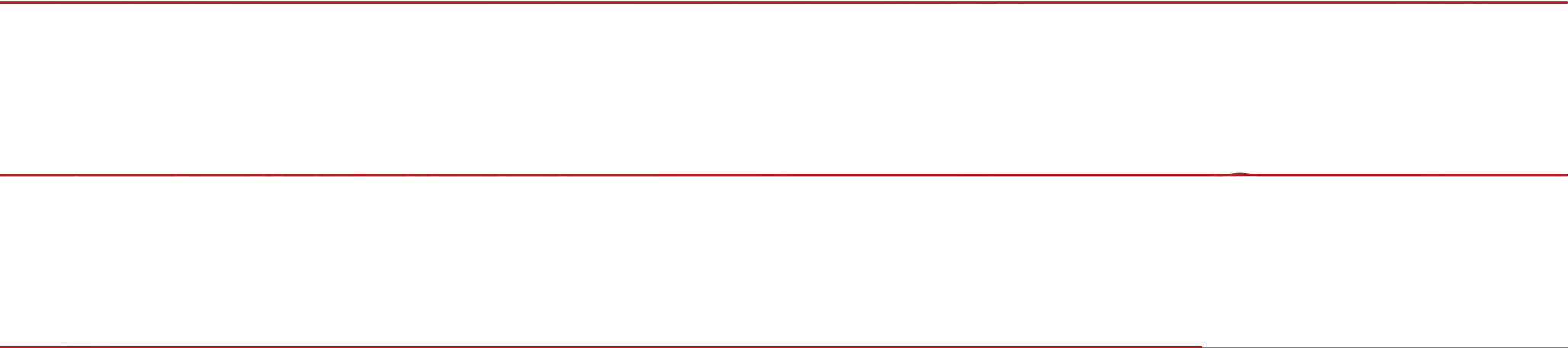

Supplement: Supplementary file 4 — Source data [file 41467_2026_68558_MOESM4_ESM.zip › Source data/Sanger-sequencing data/Suppl.Fig2h/Late-G1-Dlk1.pdf]

Page: 1 / 4  
15.01.2024

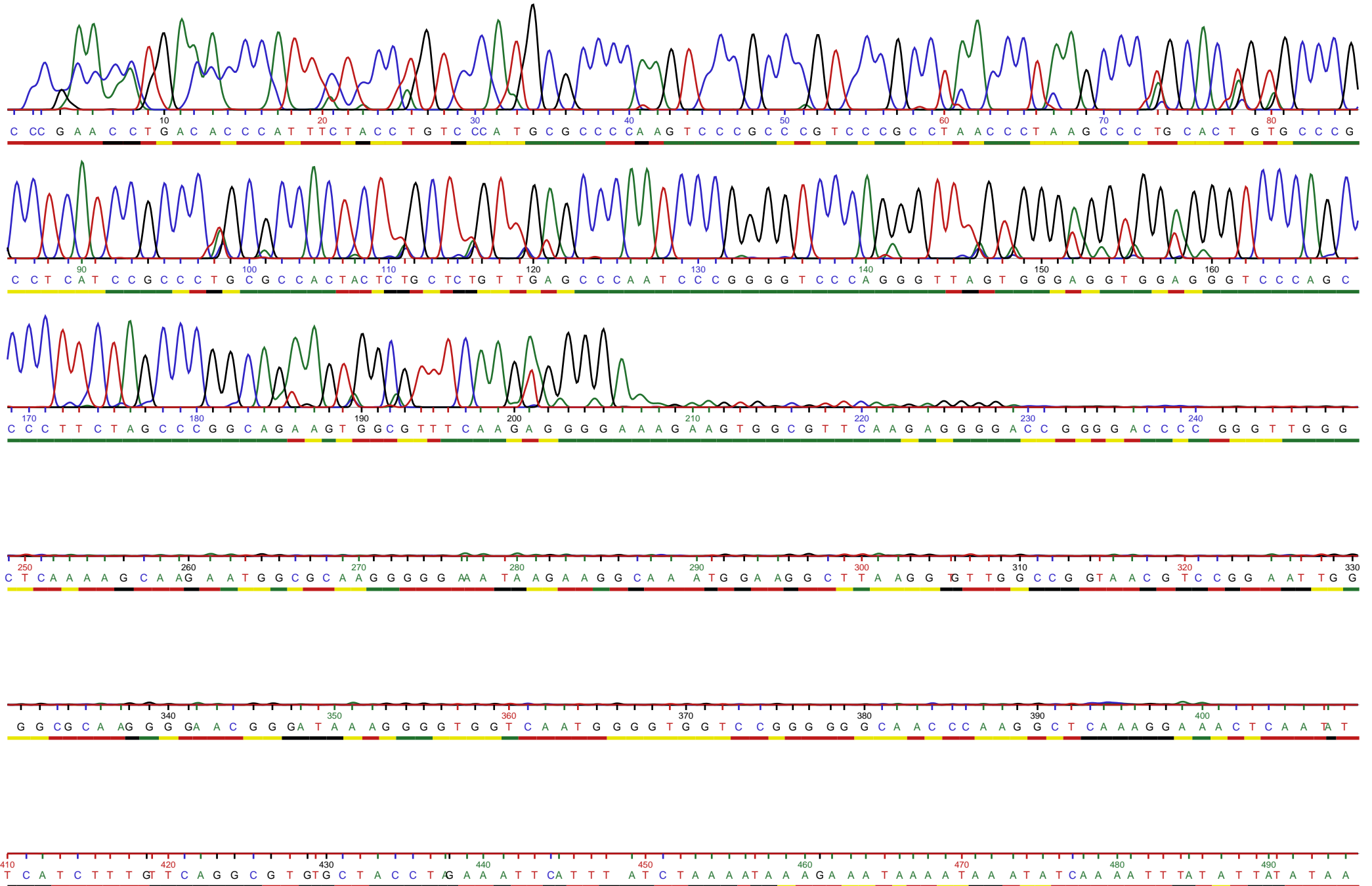

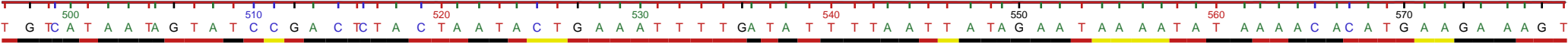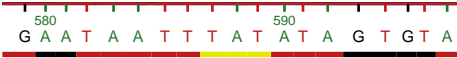

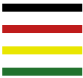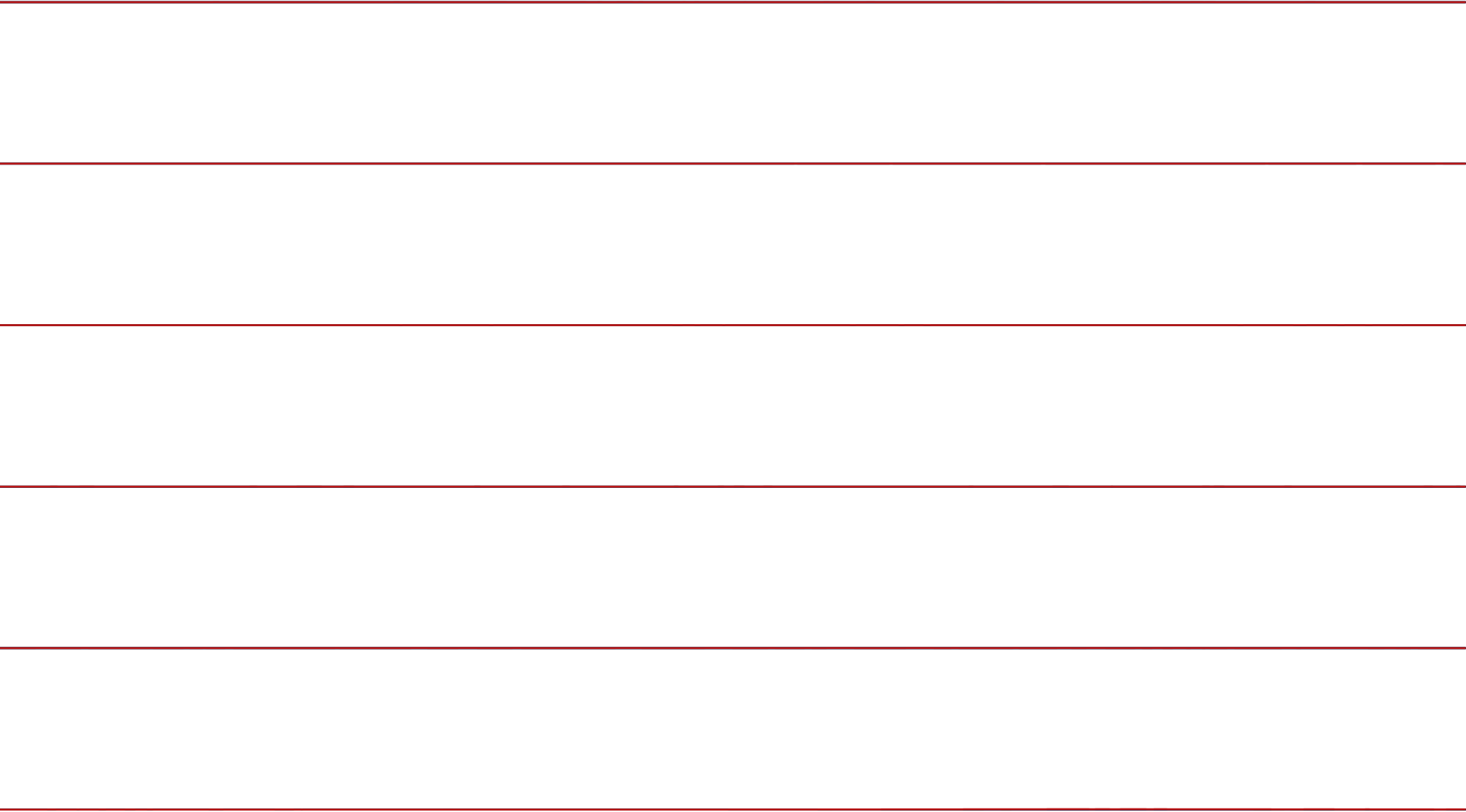

Sequence: EF71390802

Samples: 20668  
Bases: 598  
Average spacing: 35.0  
Average quality >= 10: 206, 20: 130, 30: 139

Quality: 0 - 9  
10 - 19  
20 - 29  
>= 30

Page: 4 / 4  
15.01.2024

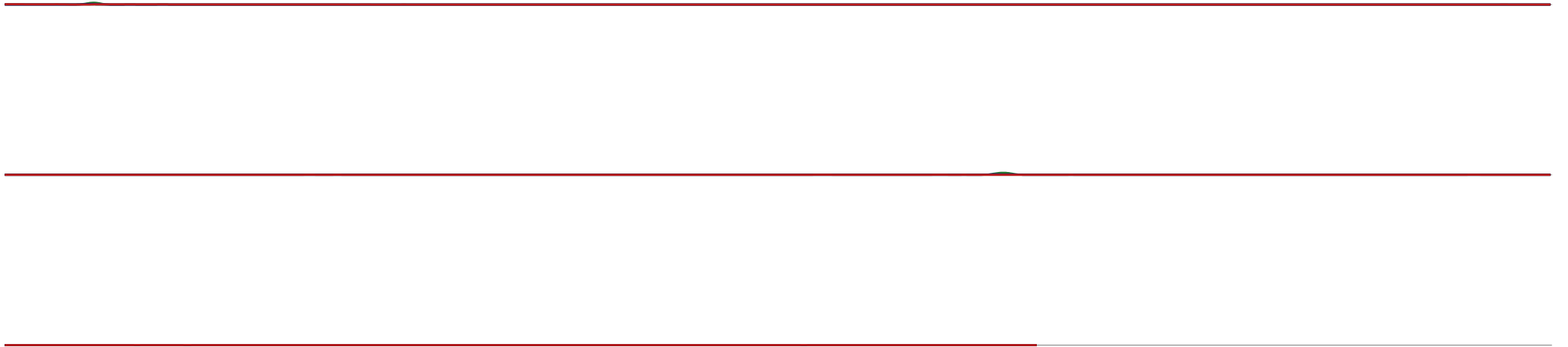

Supplement: Supplementary file 4 — Source data [file 41467_2026_68558_MOESM4_ESM.zip › Source data/Sanger-sequencing data/Suppl.Fig2h/Late-S-Dlk1.pdf]

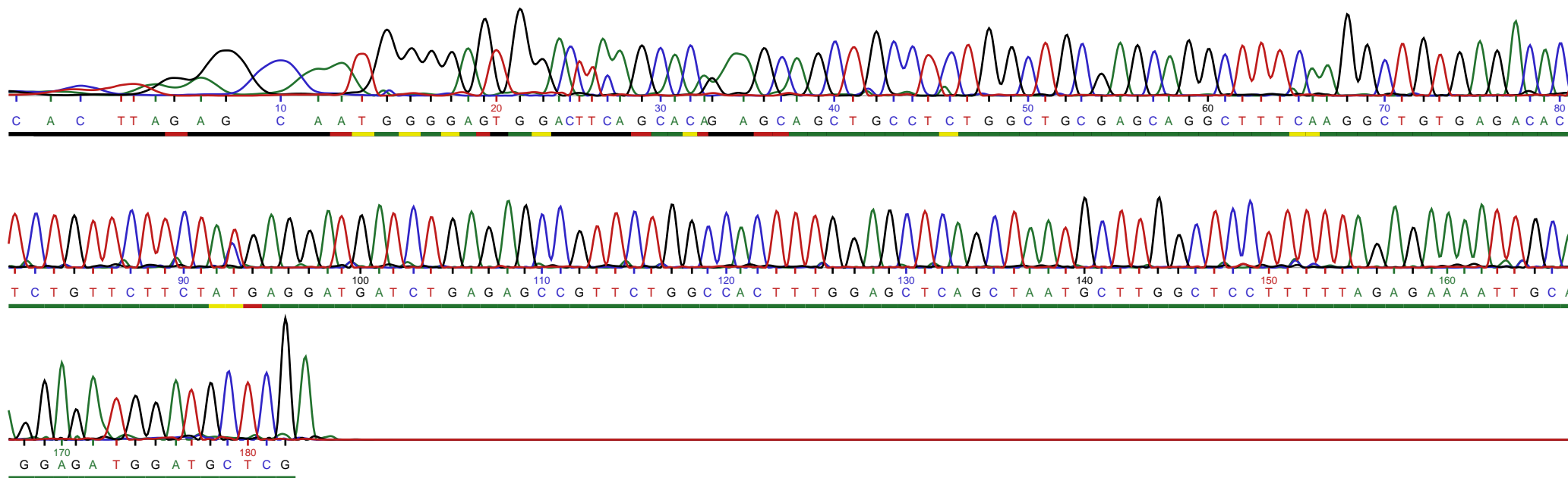

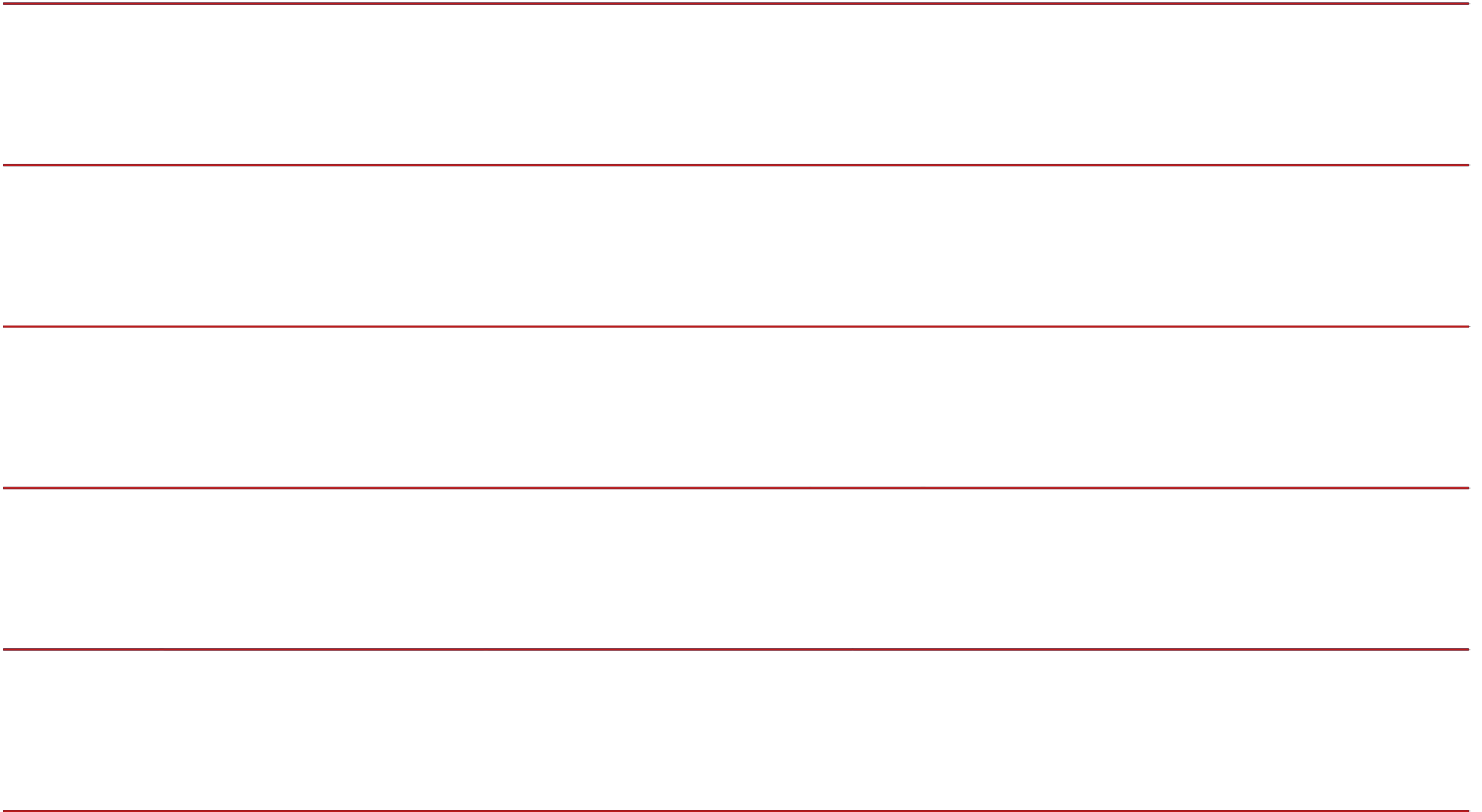

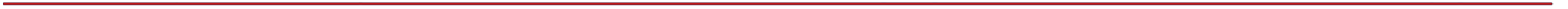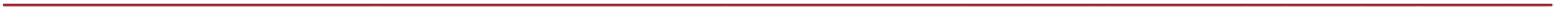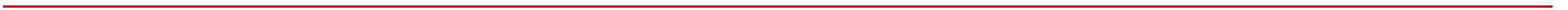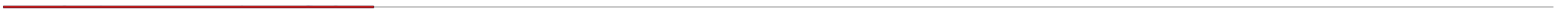

Supplement: Supplementary file 4 — Source data [file 41467_2026_68558_MOESM4_ESM.zip › Source data/Sanger-sequencing data/Suppl.Fig3c/Sh1-early-Meg3.pdf]

Sequence: EF70467505

Samples: 15770  
Bases: 186  
Average spacing: 85.0  
Average quality >= 10: 8, 20: 9, 30: 150

Quality: 0 - 9  
10 - 19  
20 - 29  
≥ 30

Page: 1 / 3  
20.10.2022

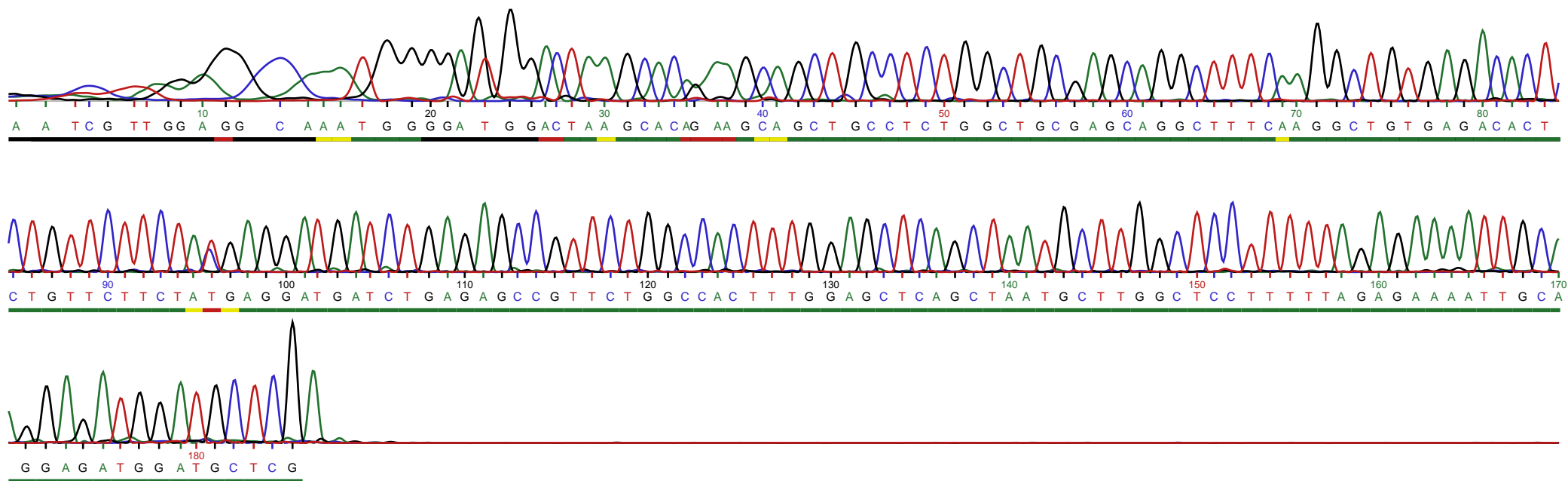

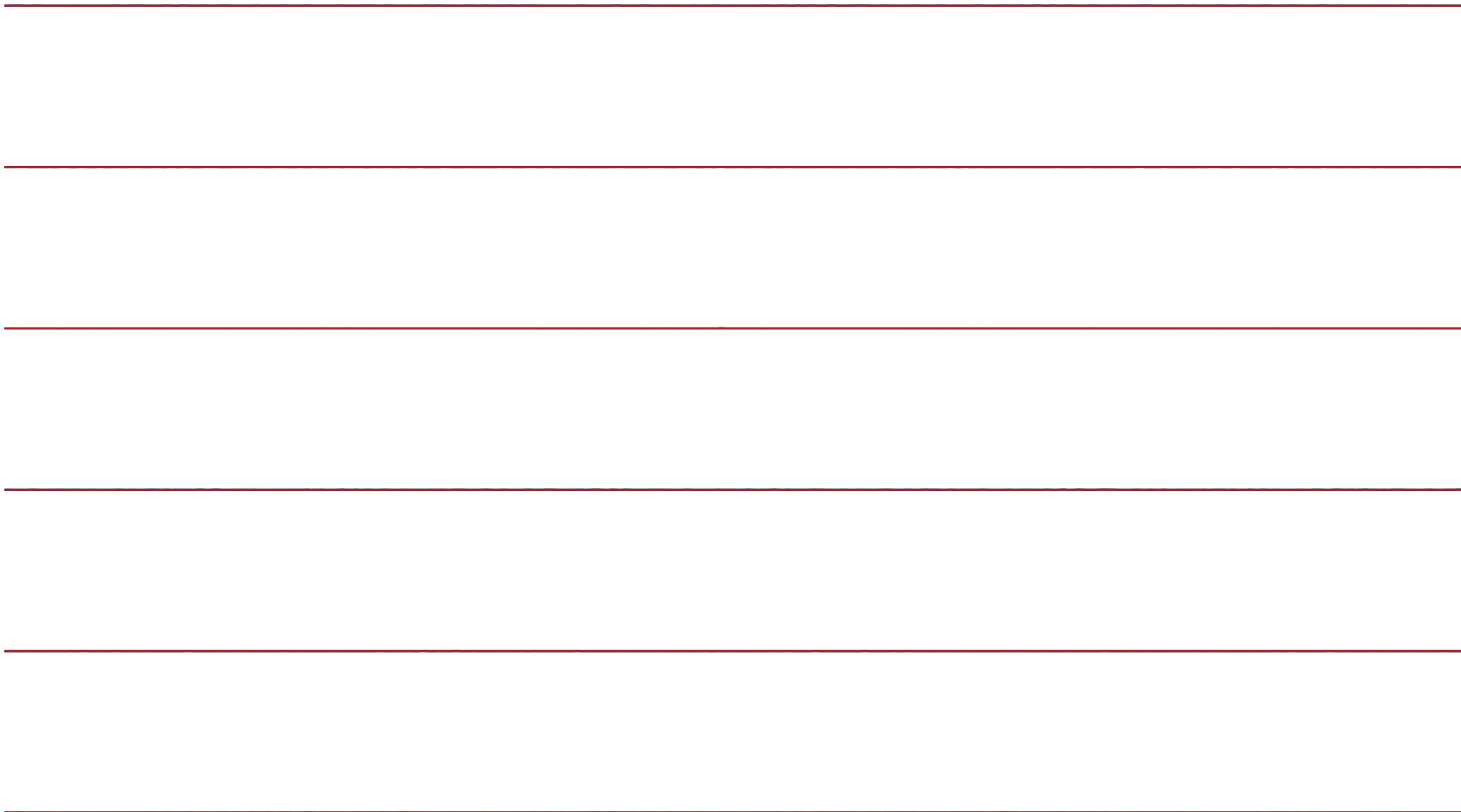

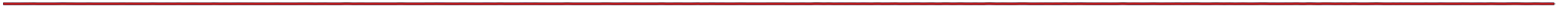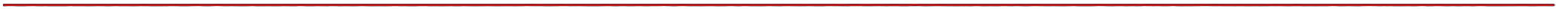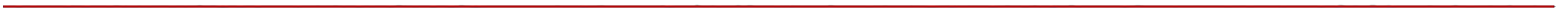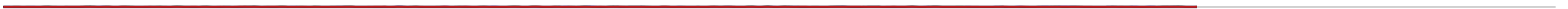

Supplement: Supplementary file 4 — Source data [file 41467_2026_68558_MOESM4_ESM.zip › Source data/Sanger-sequencing data/Suppl.Fig3c/Sh1-late-Meg3.pdf]

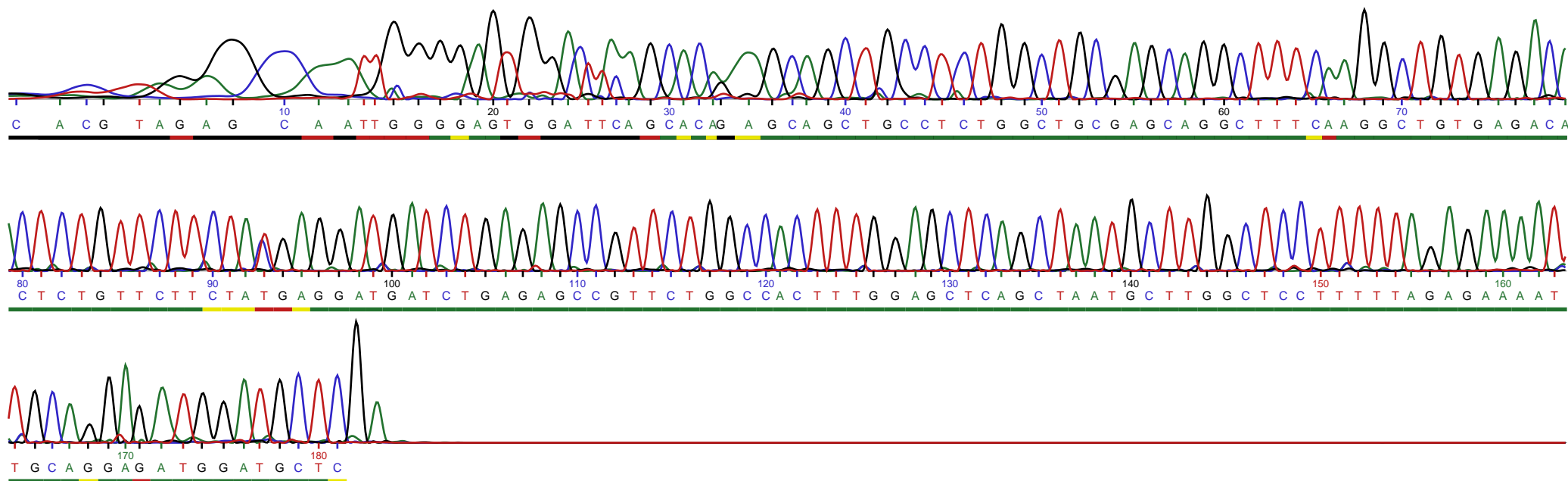

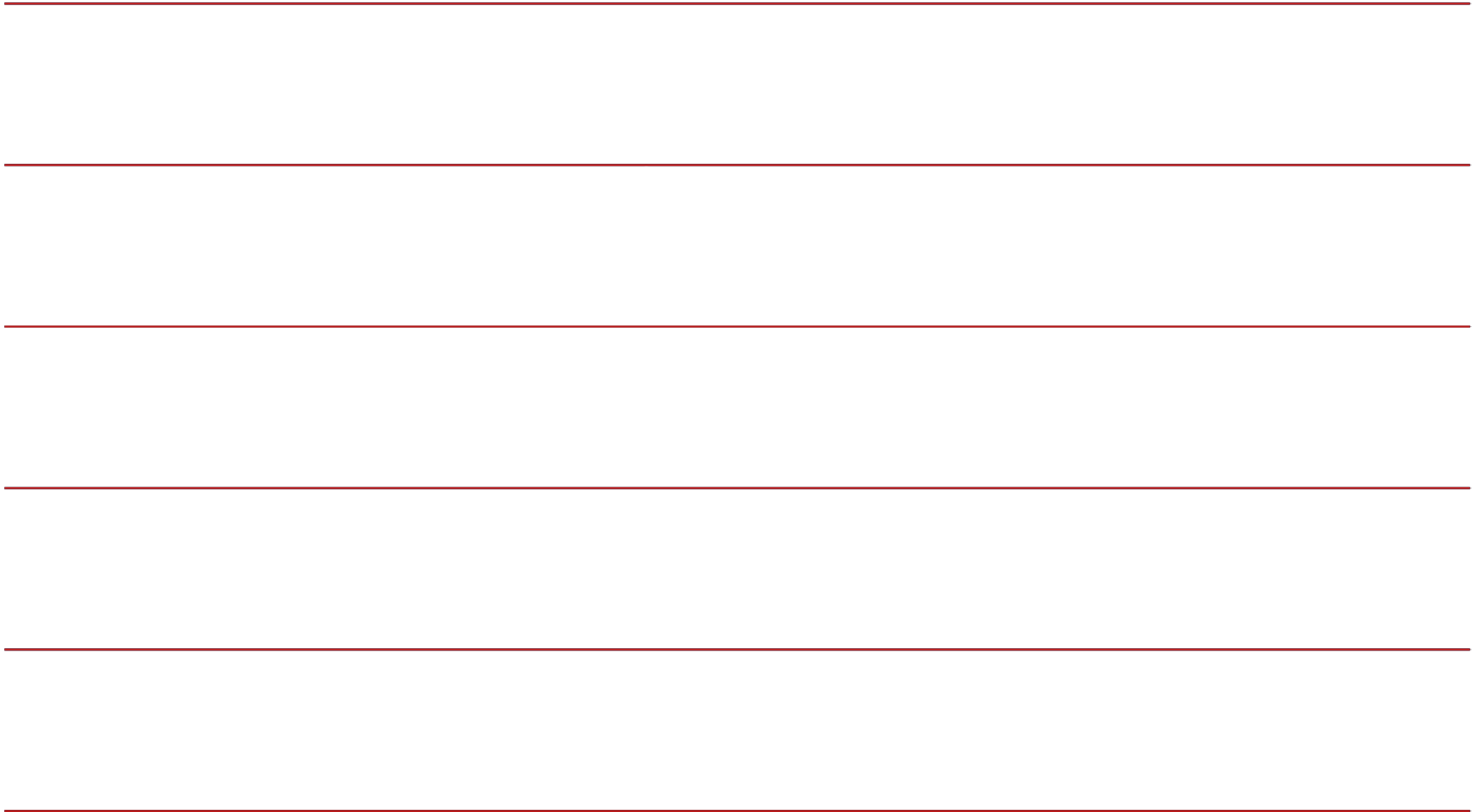

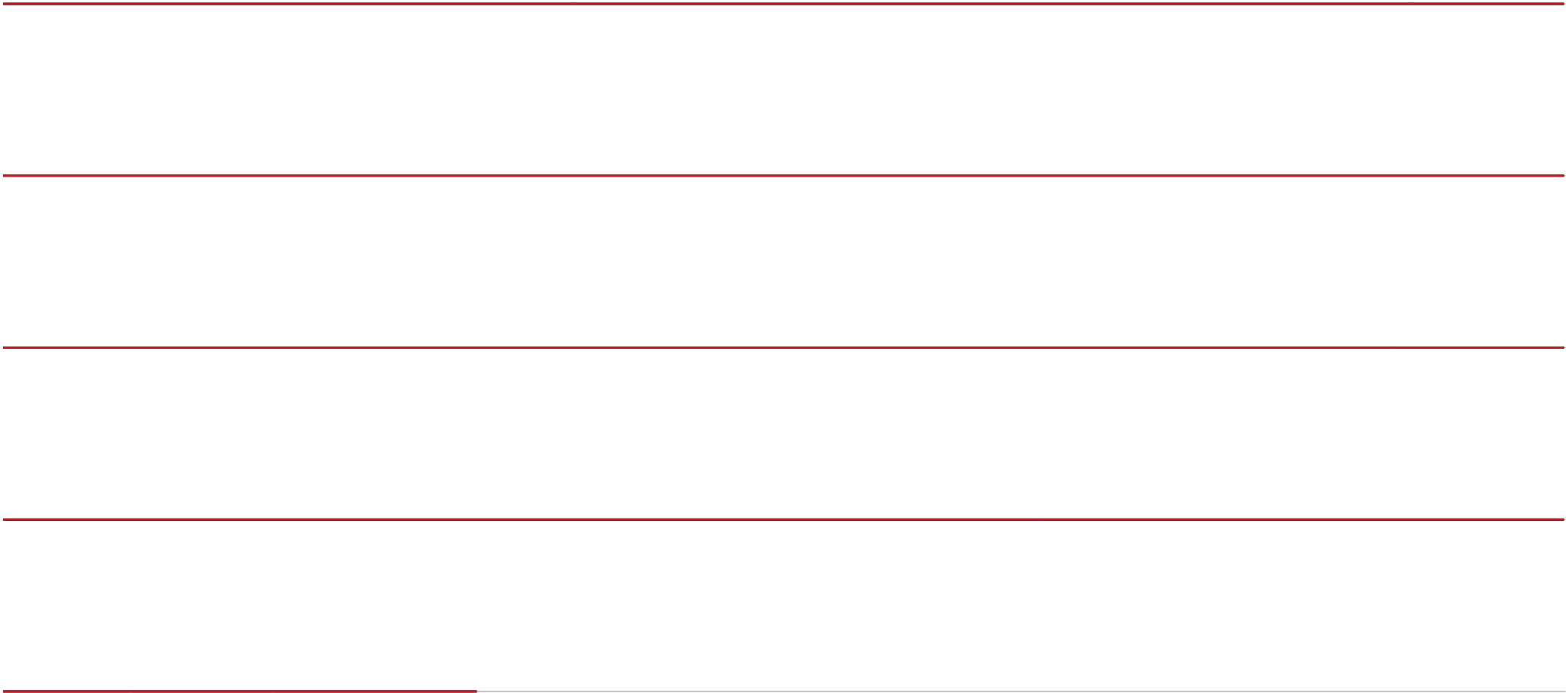

Supplement: Supplementary file 4 — Source data [file 41467_2026_68558_MOESM4_ESM.zip › Source data/Sanger-sequencing data/Suppl.Fig3d/Zfp57-early-Meg3.pdf]

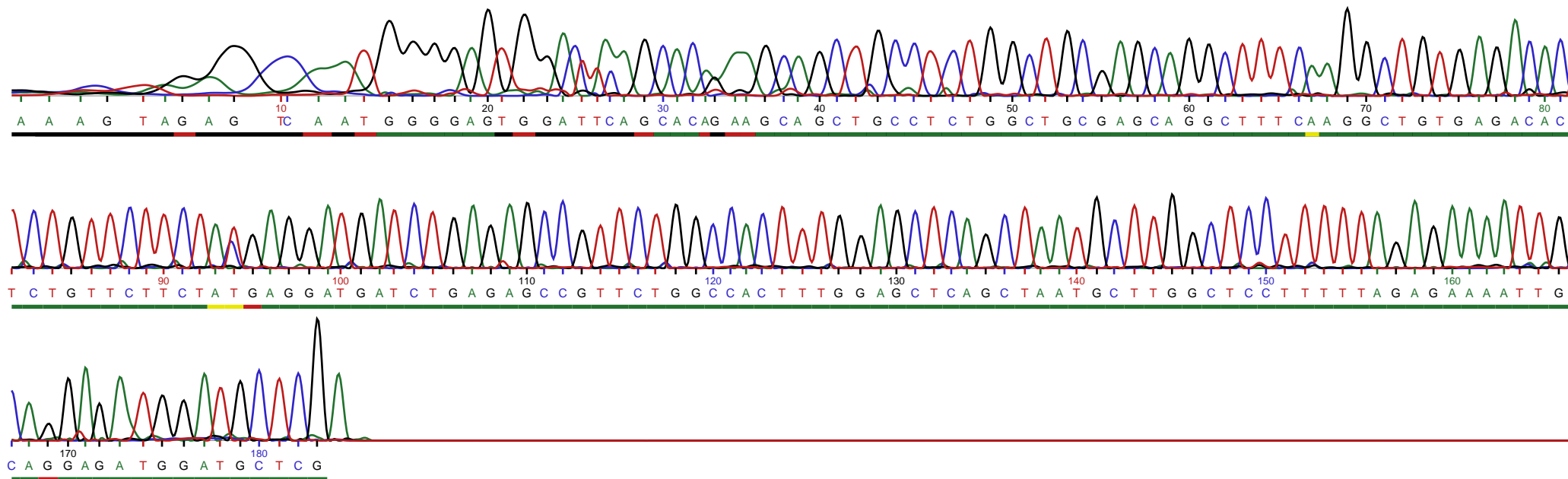

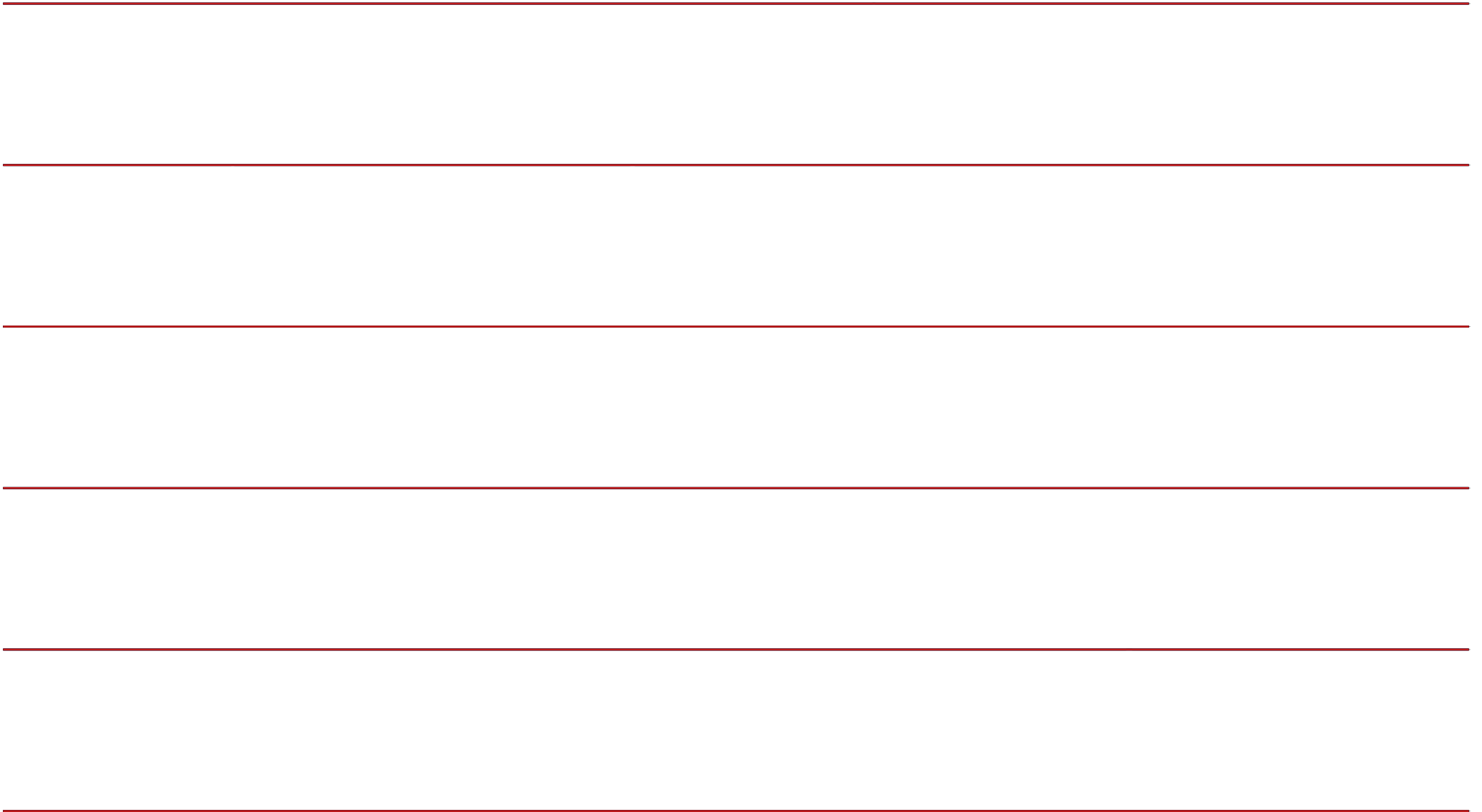

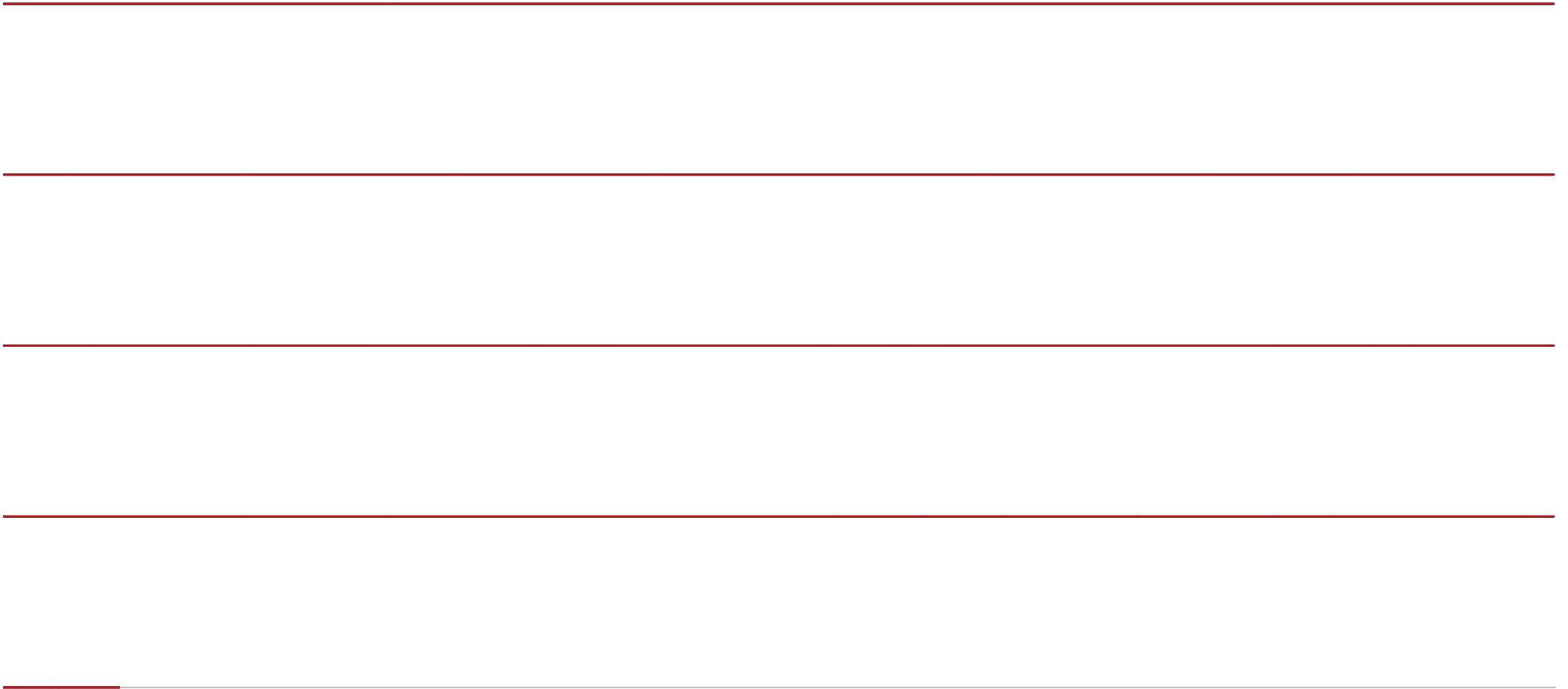

Supplement: Supplementary file 4 — Source data [file 41467_2026_68558_MOESM4_ESM.zip › Source data/Sanger-sequencing data/Suppl.Fig3d/Zfp57-late-Meg3.pdf]

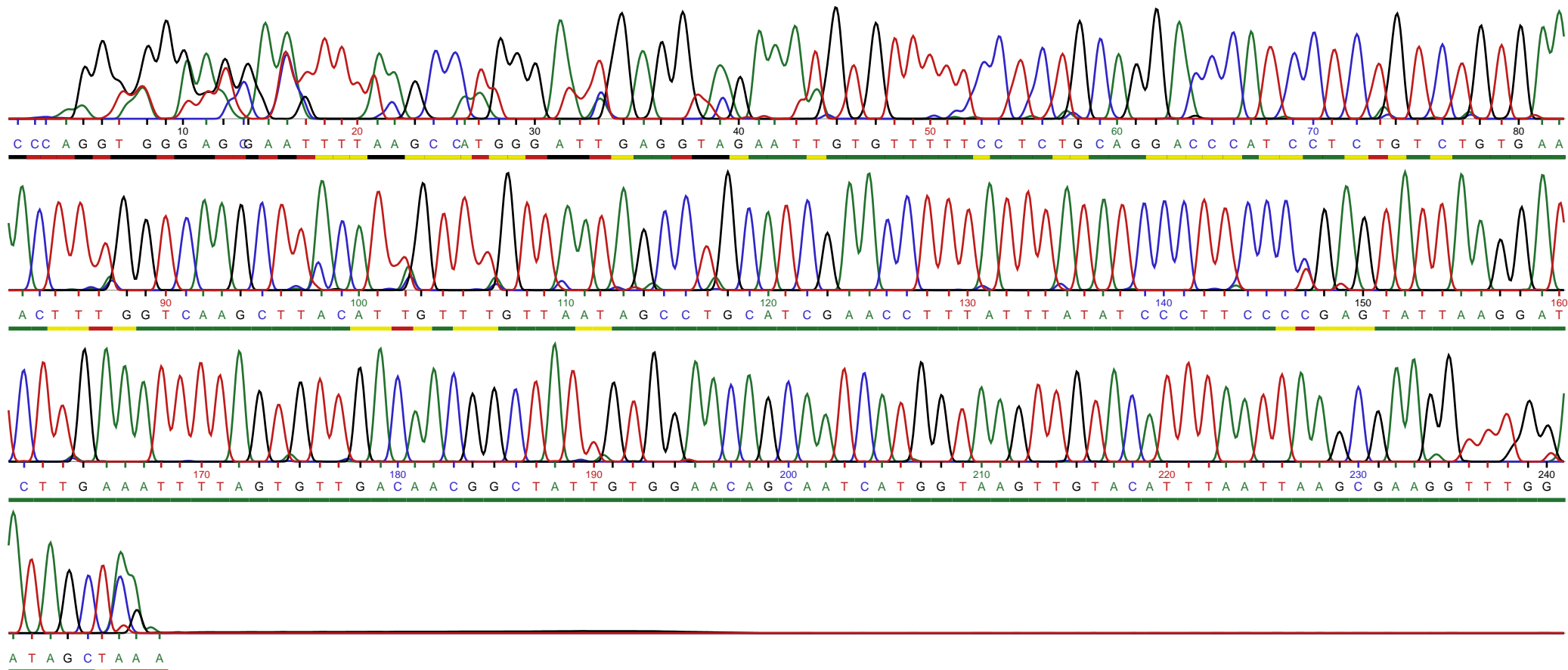

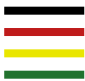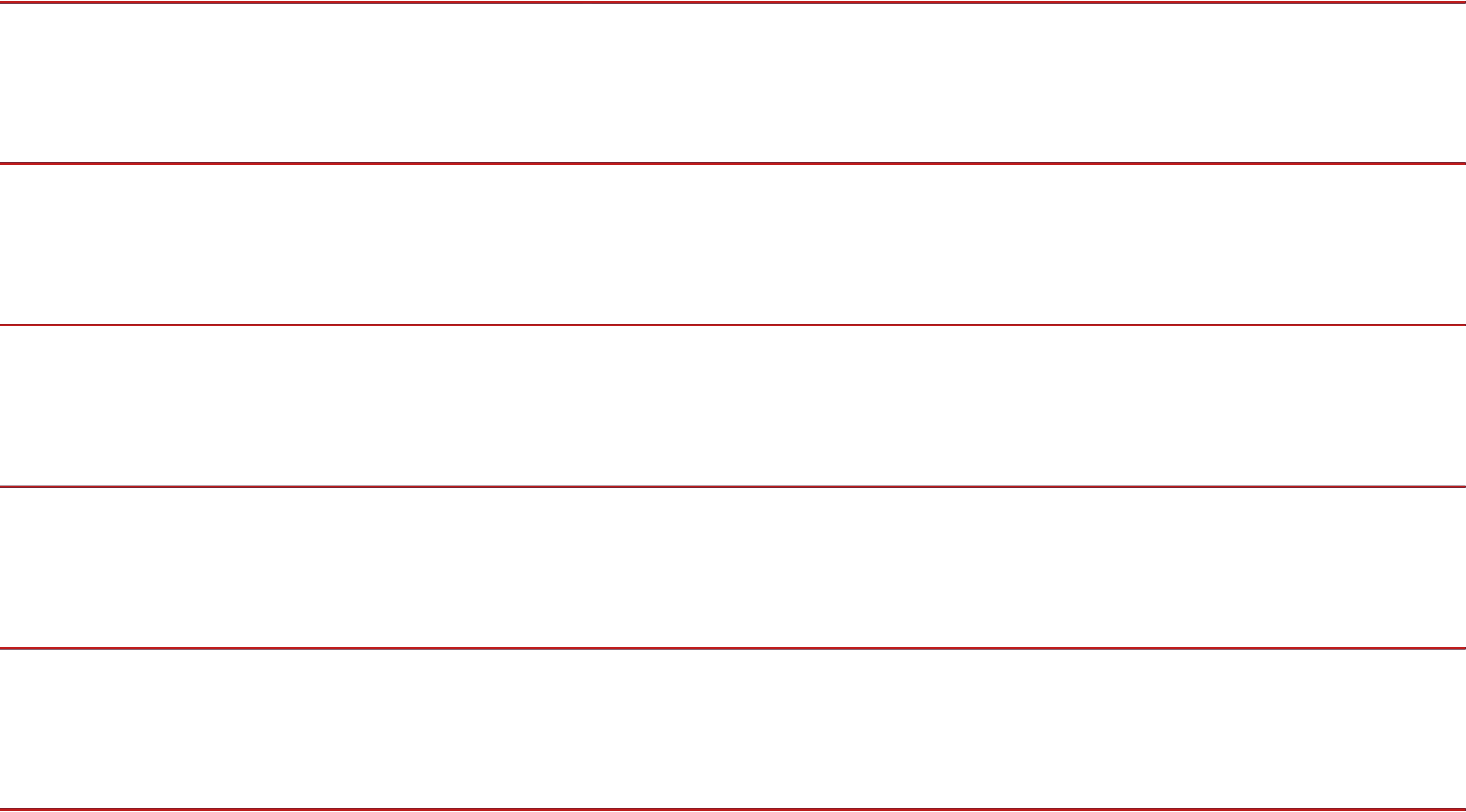

Sequence: EF72763632

Samples: 12551  
Bases: 250  
Average spacing: 51.0  
Average quality >= 10: 21, 20: 40, 30: 175

Quality: 0 - 9 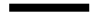  
10 - 19 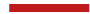  
20 - 29 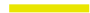  
>= 30 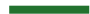

Page: 3 / 3  
03.06.2024

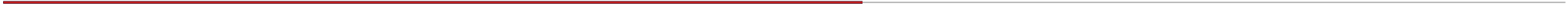

Supplement: Supplementary file 4 — Source data [file 41467_2026_68558_MOESM4_ESM.zip › Source data/Sanger-sequencing data/Suppl.Fig3f/Zfp57KO-early-Snrpn.pdf]

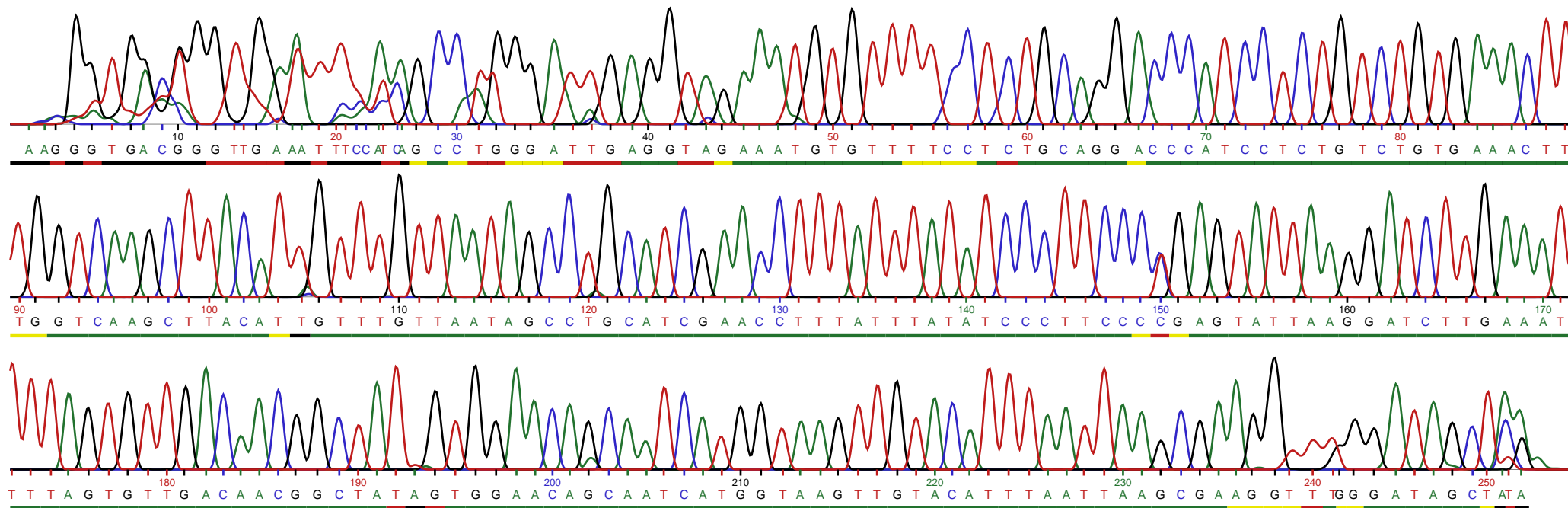

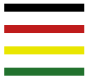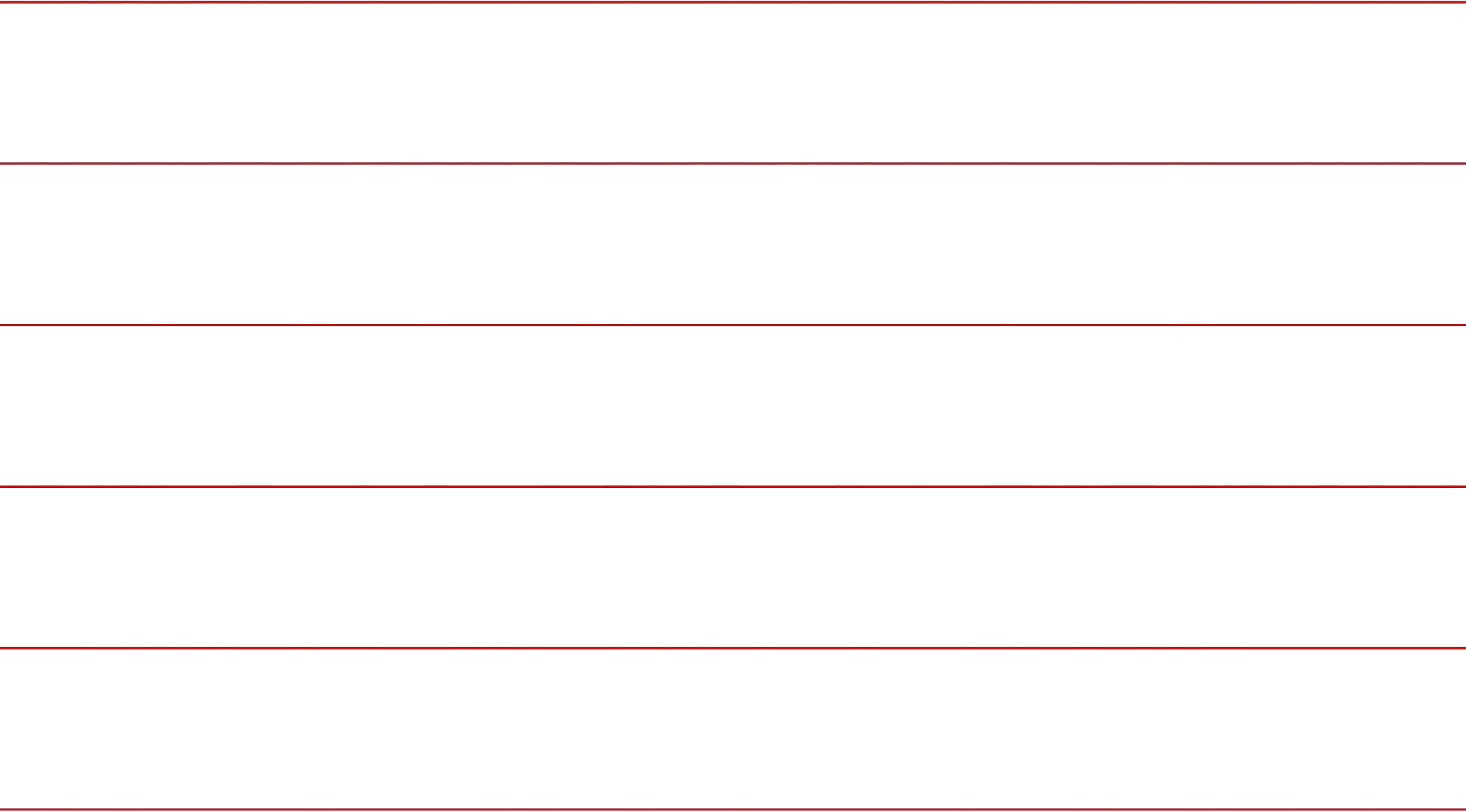

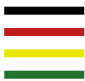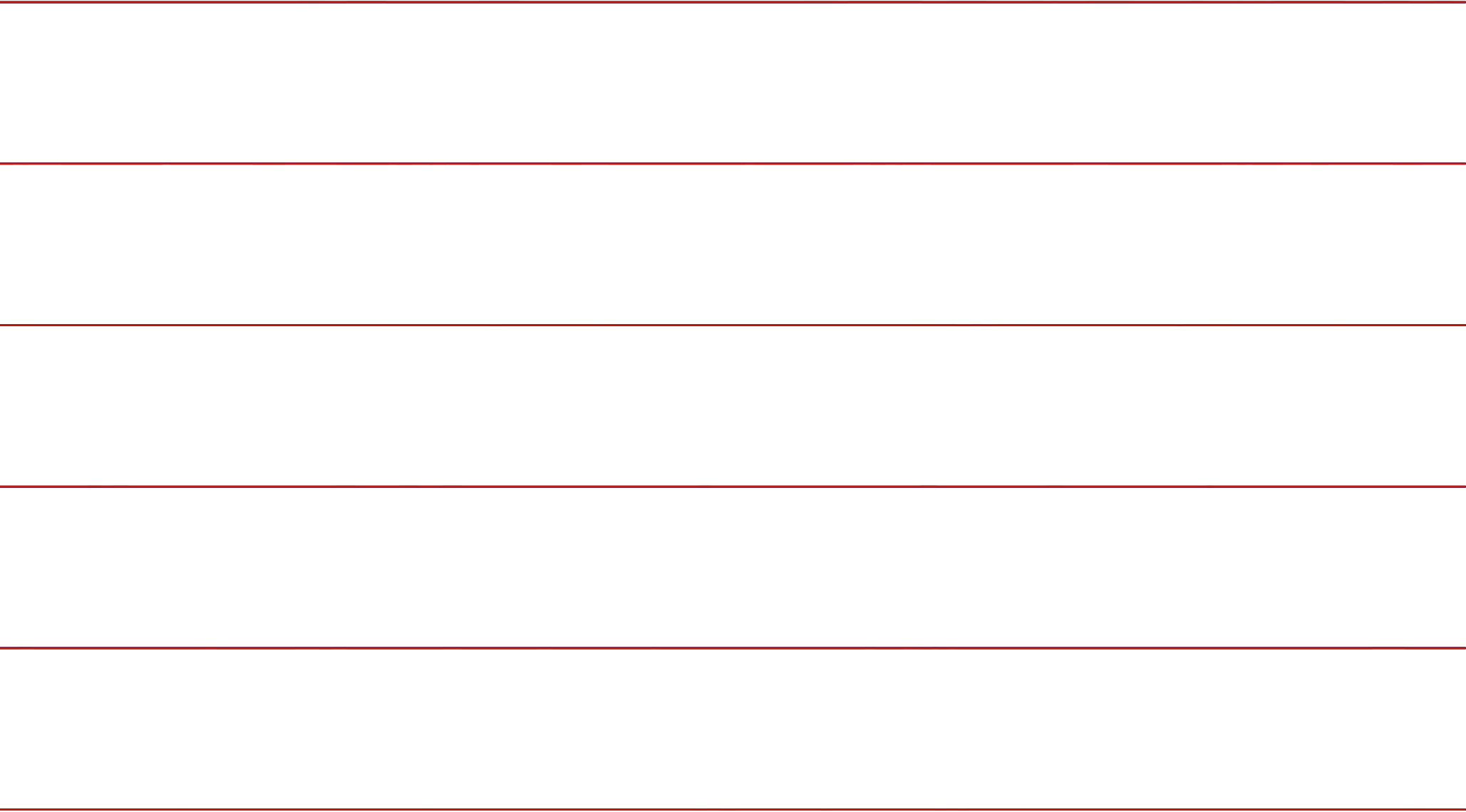

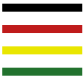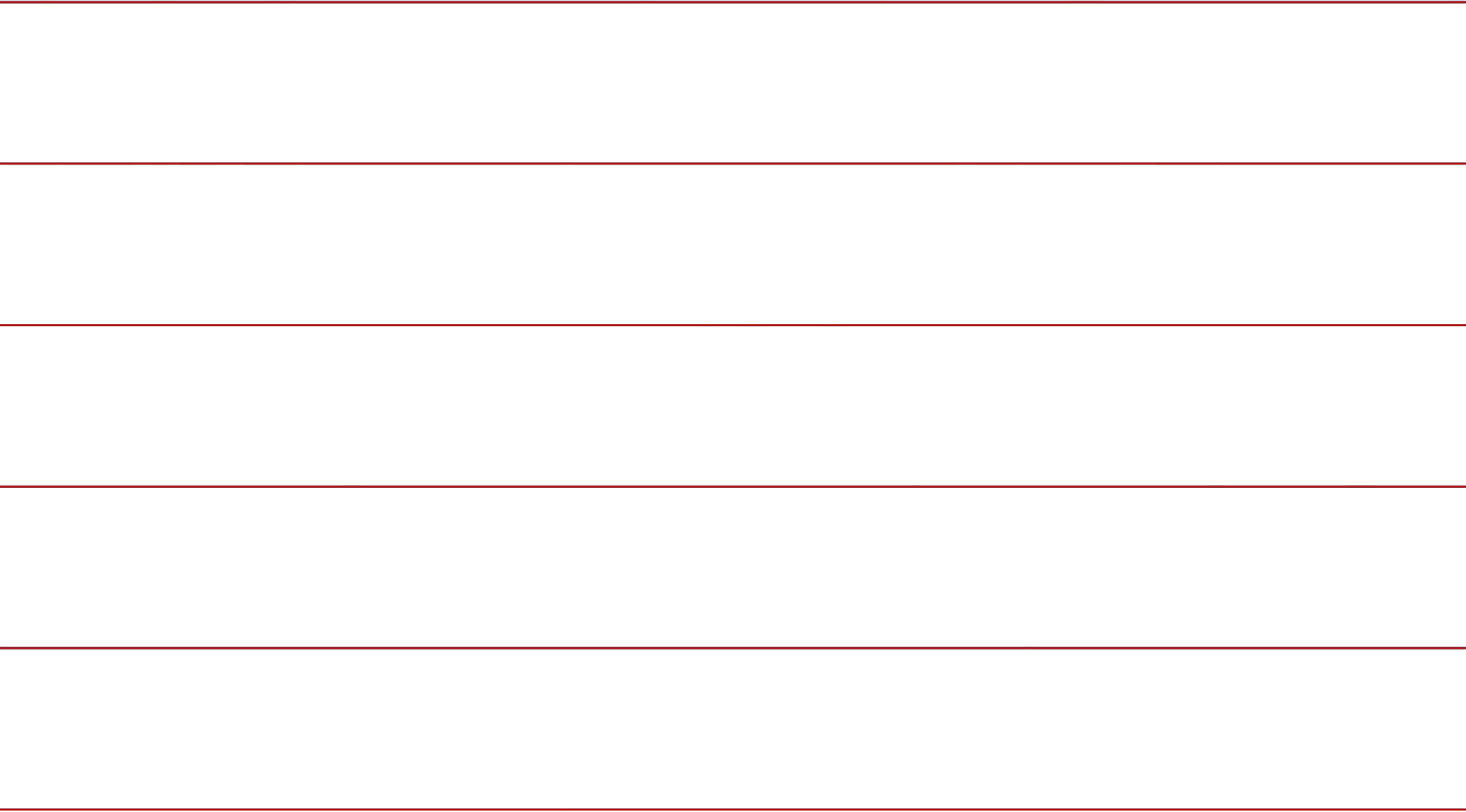

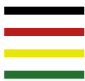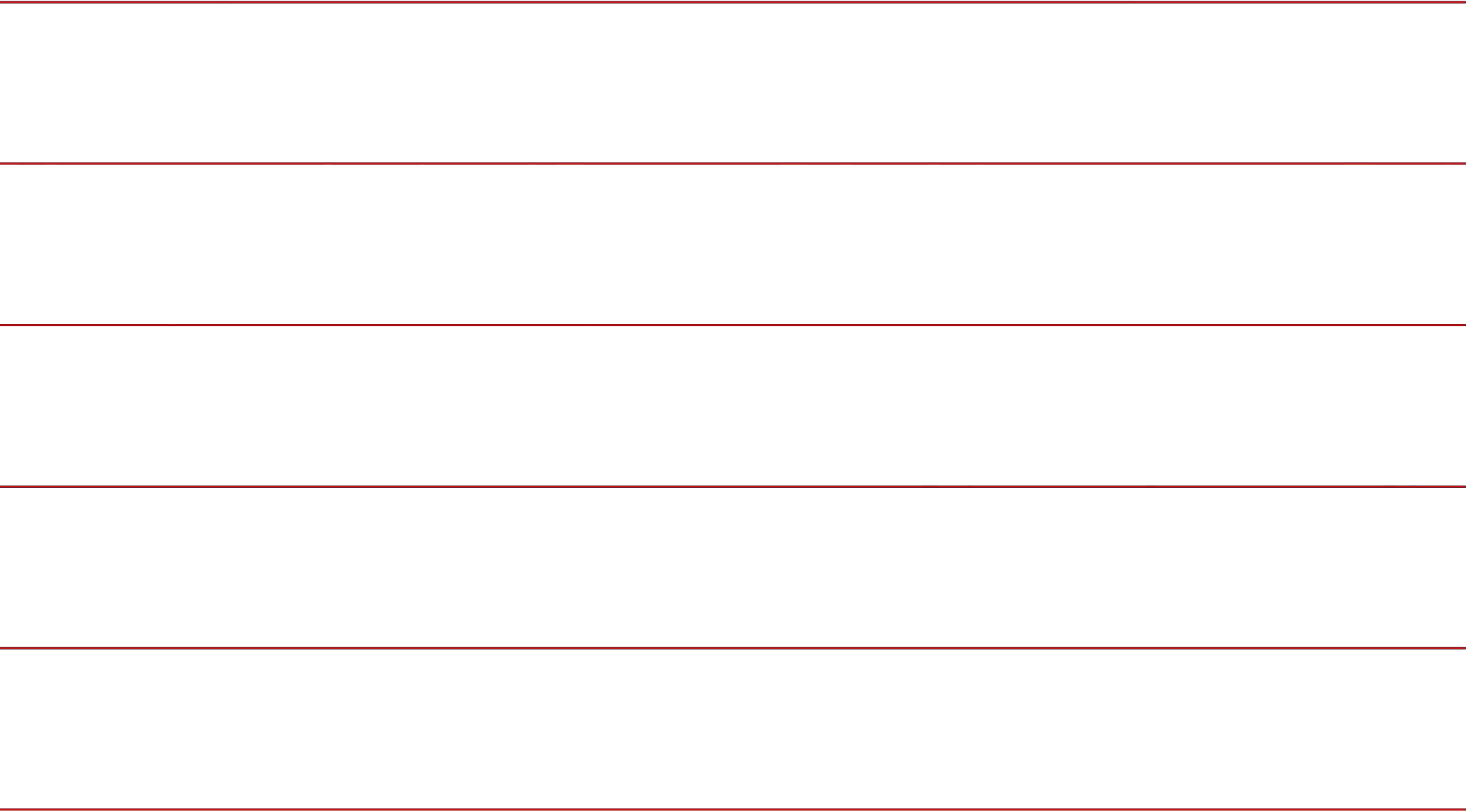

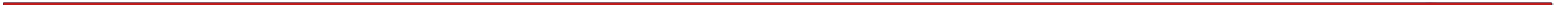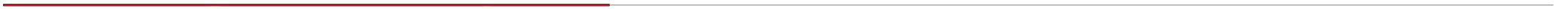

Supplement: Supplementary file 4 — Source data [file 41467_2026_68558_MOESM4_ESM.zip › Source data/Sanger-sequencing data/Suppl.Fig3f/Zfp57KO-late-Snrpn.pdf]

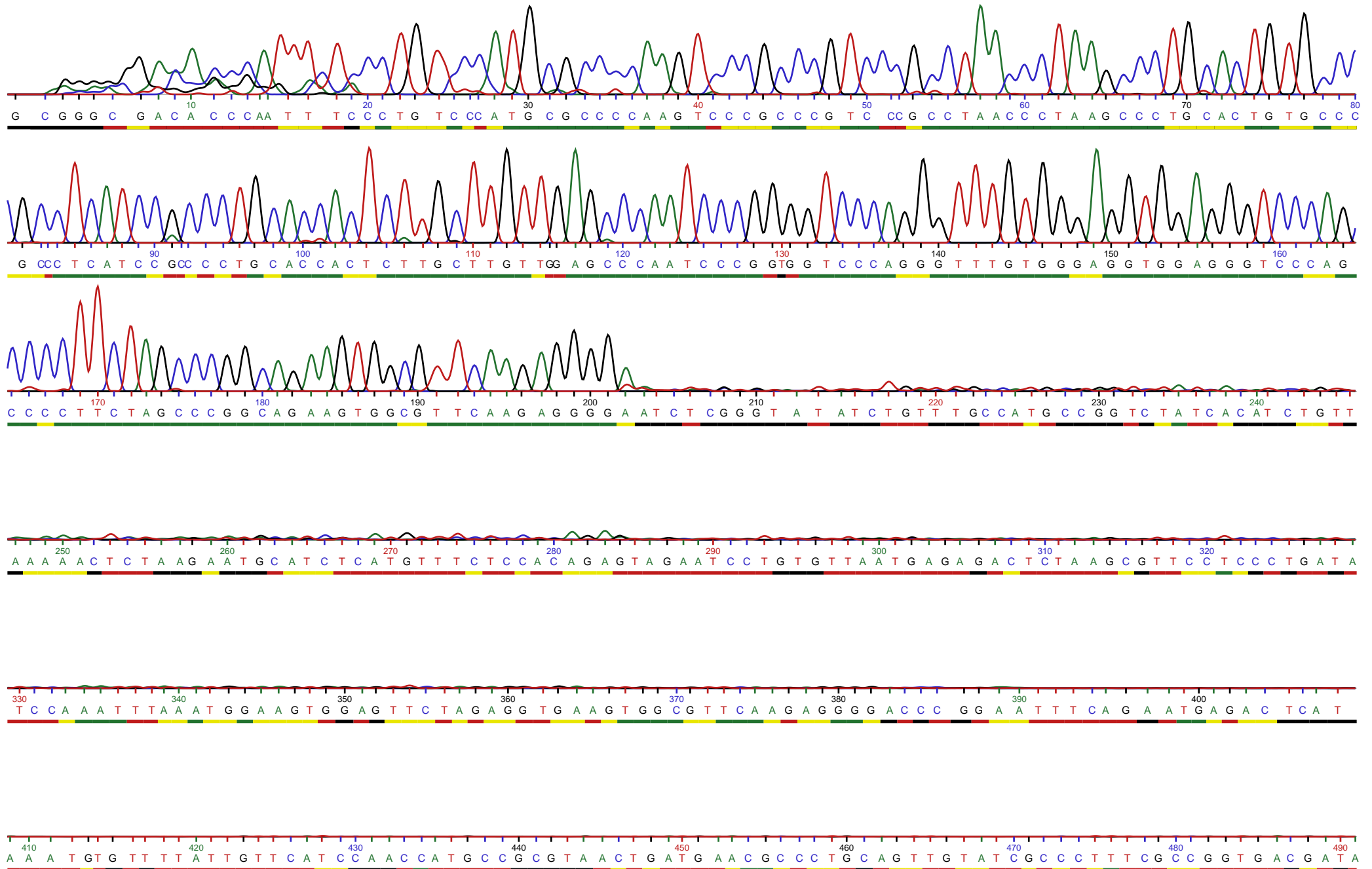

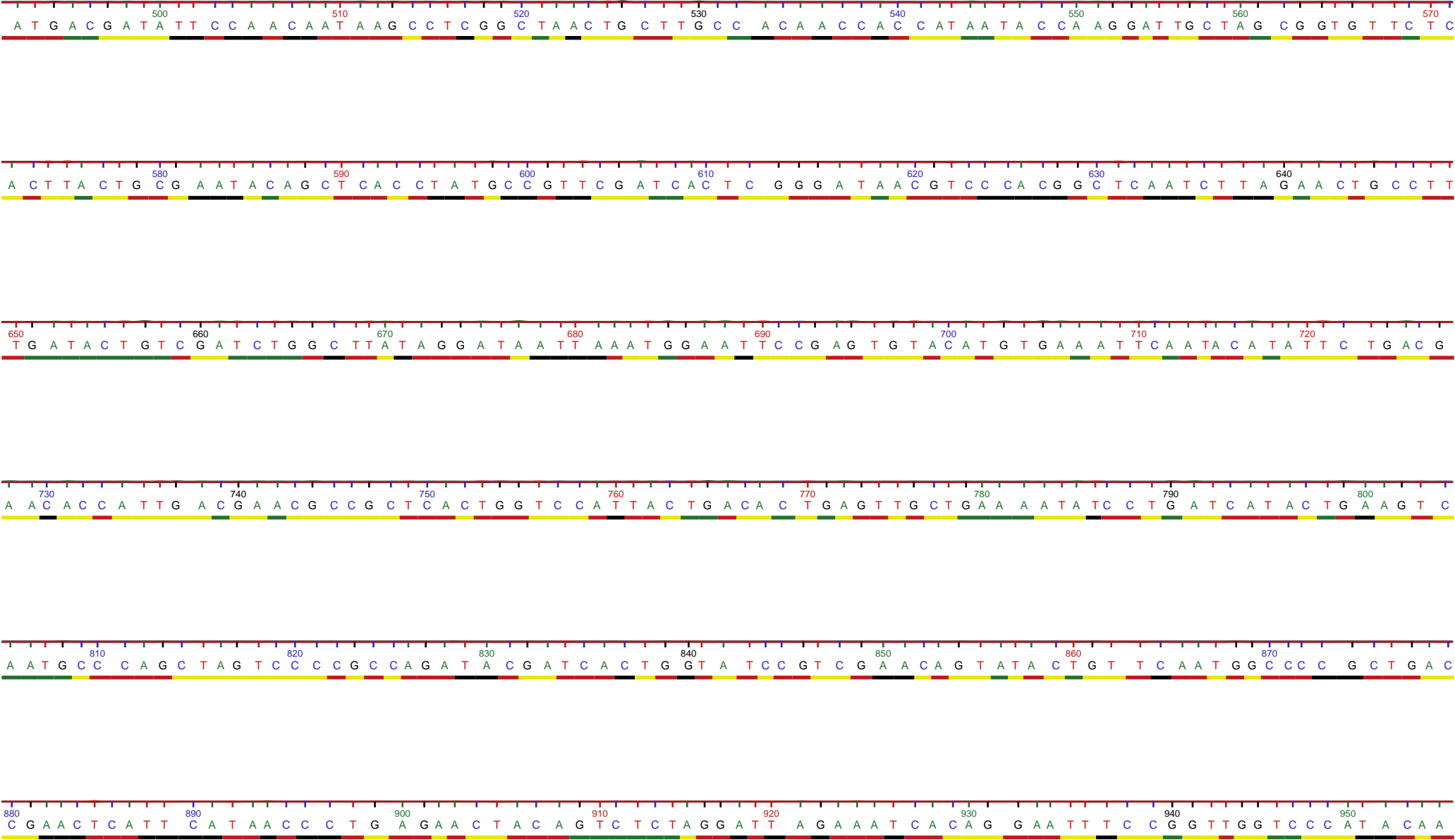

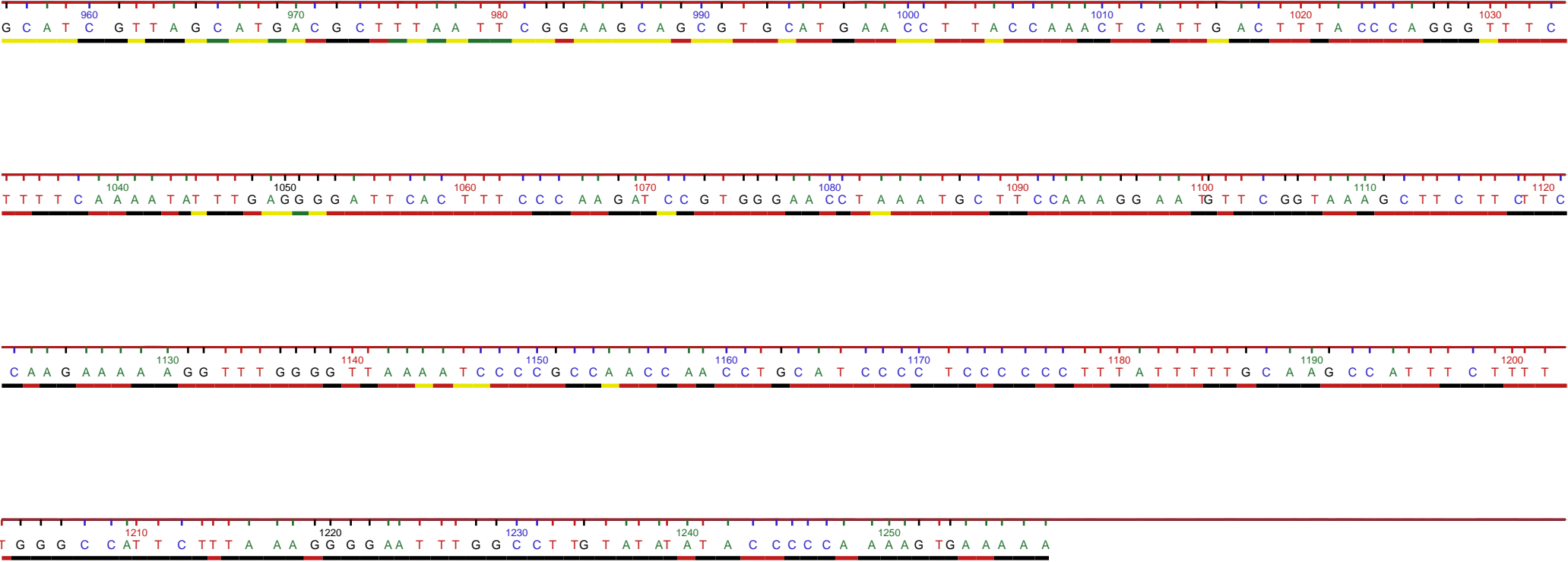

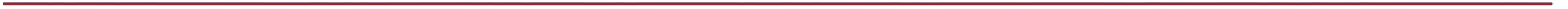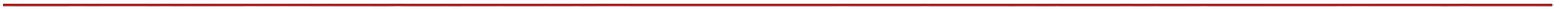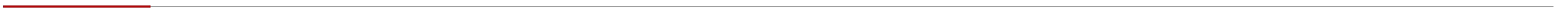

Supplement: Supplementary file 4 — Source data [file 41467_2026_68558_MOESM4_ESM.zip › Source data/Sanger-sequencing data/Suppl.Fig6f/Ori-early-Dlk1.pdf]

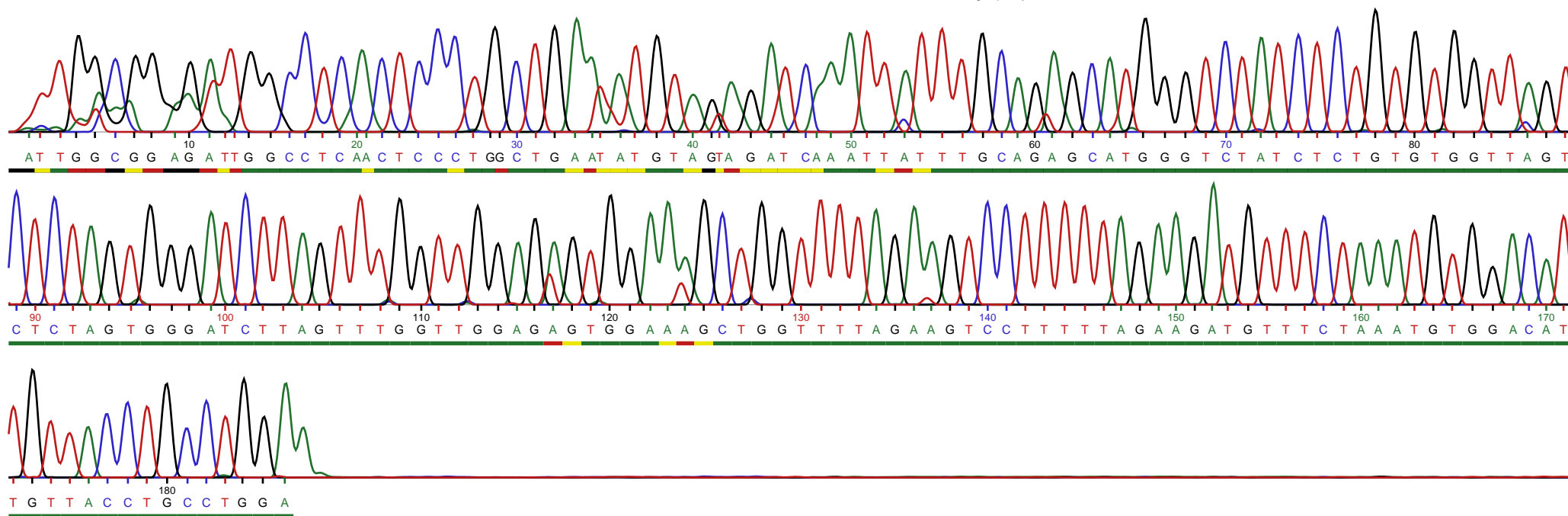

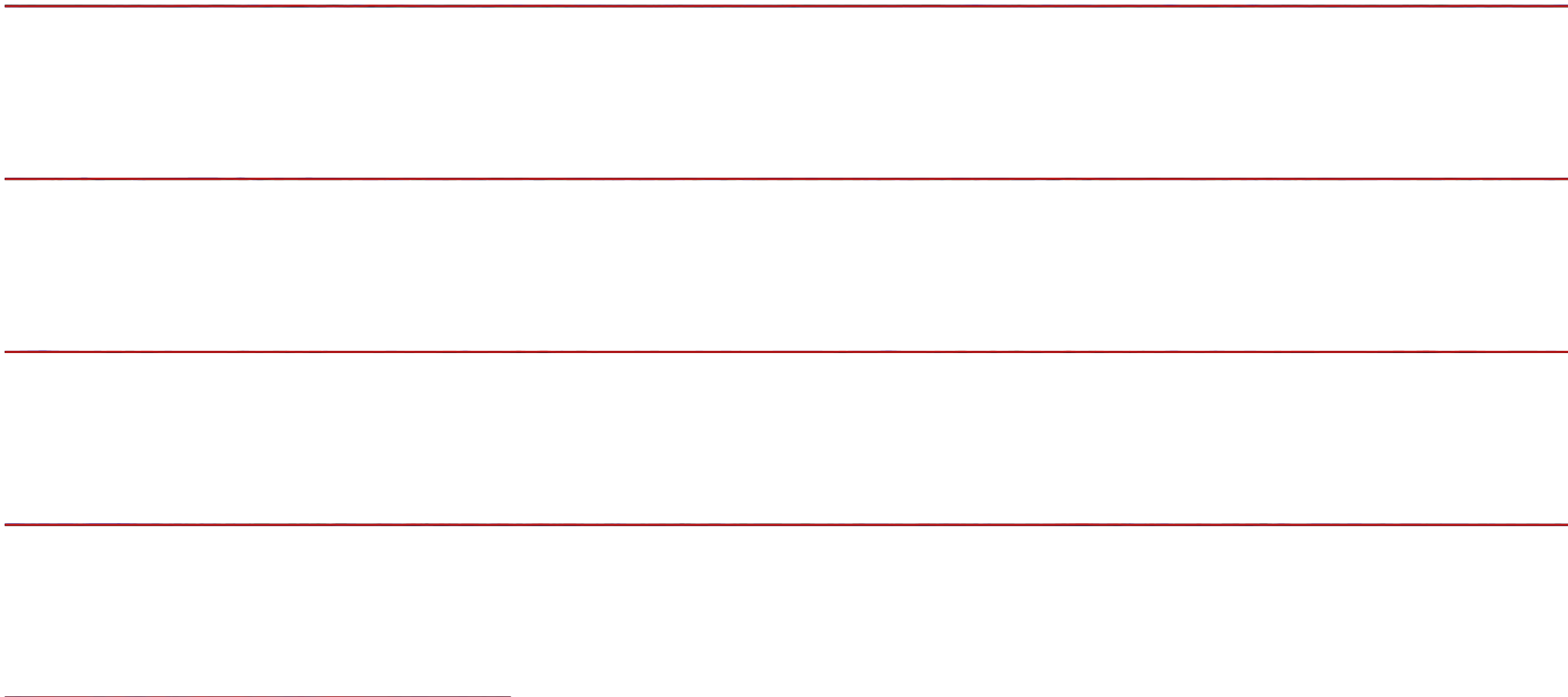

Supplement: Supplementary file 4 — Source data [file 41467_2026_68558_MOESM4_ESM.zip › Source data/Sanger-sequencing data/Suppl.Fig6f/Ori-early-Meg3.pdf]

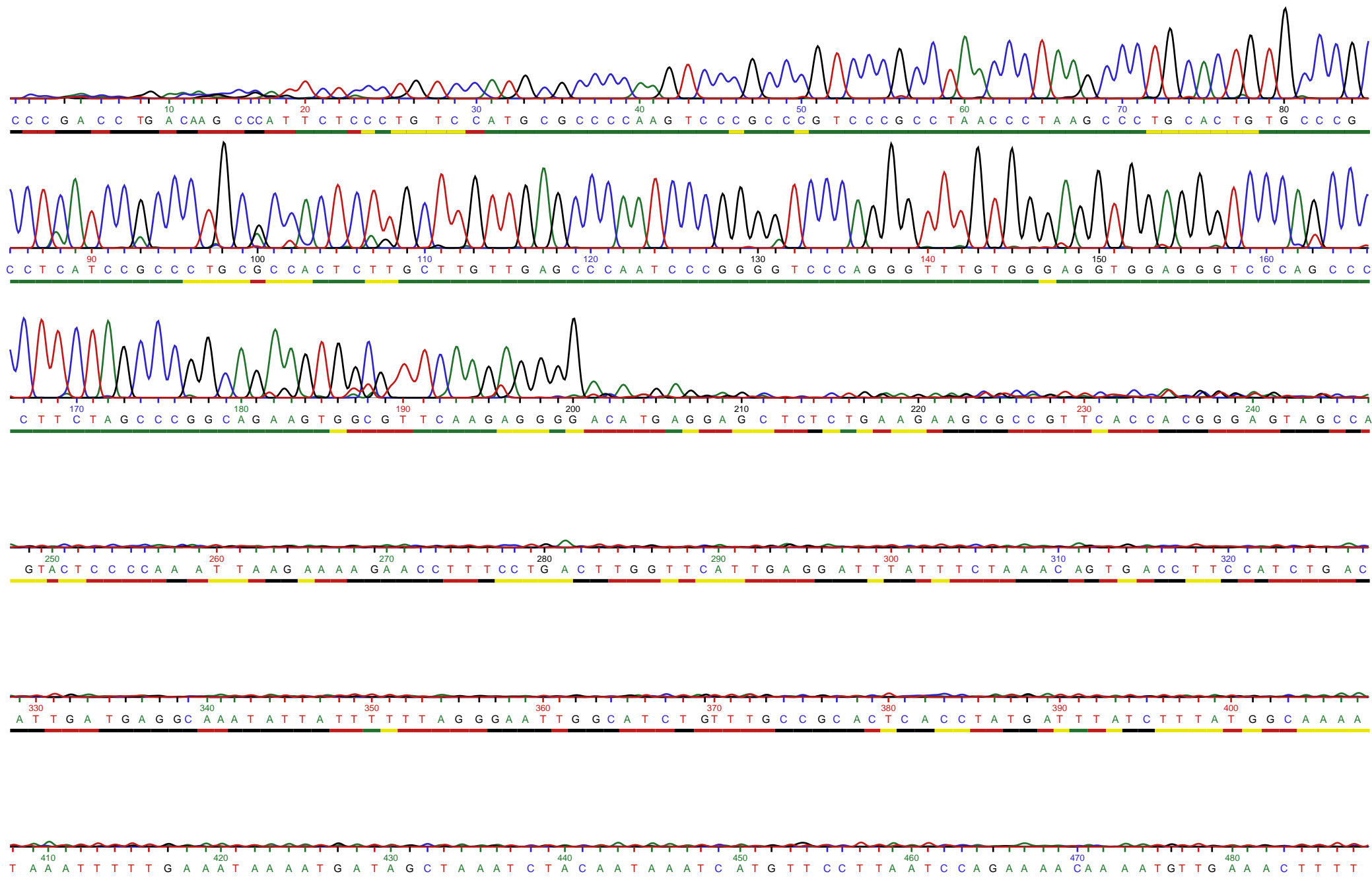

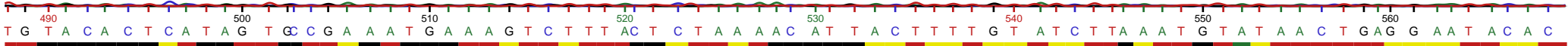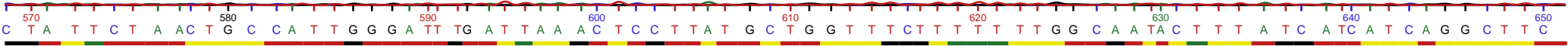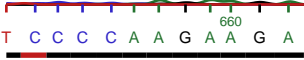

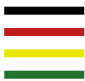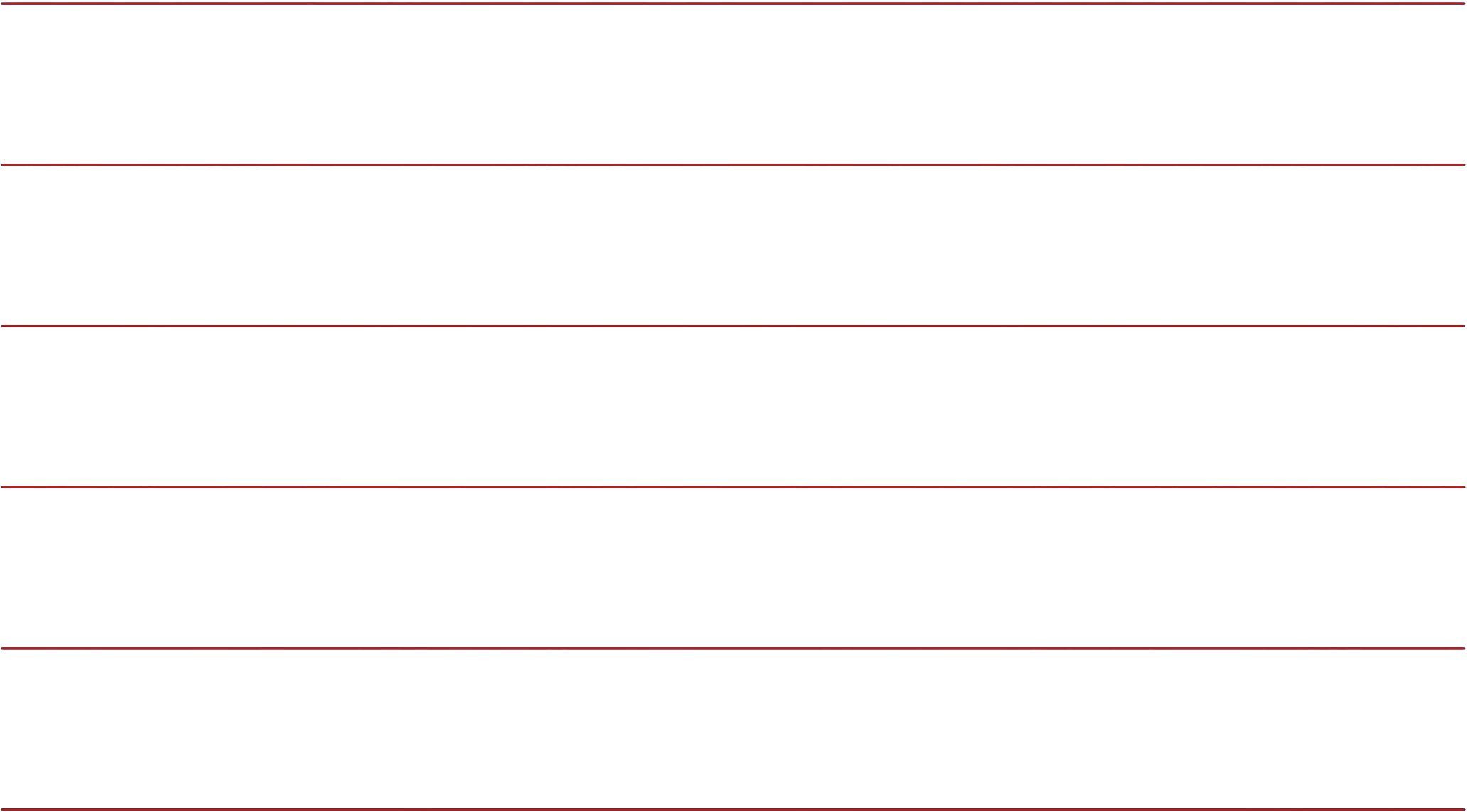

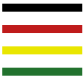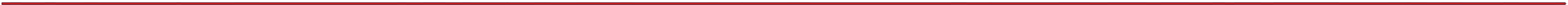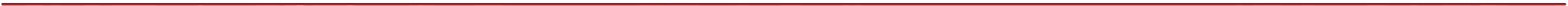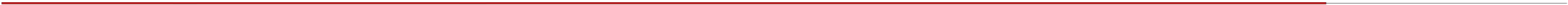

Supplement: Supplementary file 4 — Source data [file 41467_2026_68558_MOESM4_ESM.zip › Source data/Sanger-sequencing data/Suppl.Fig6f/Ori-late-Dlk1.pdf]

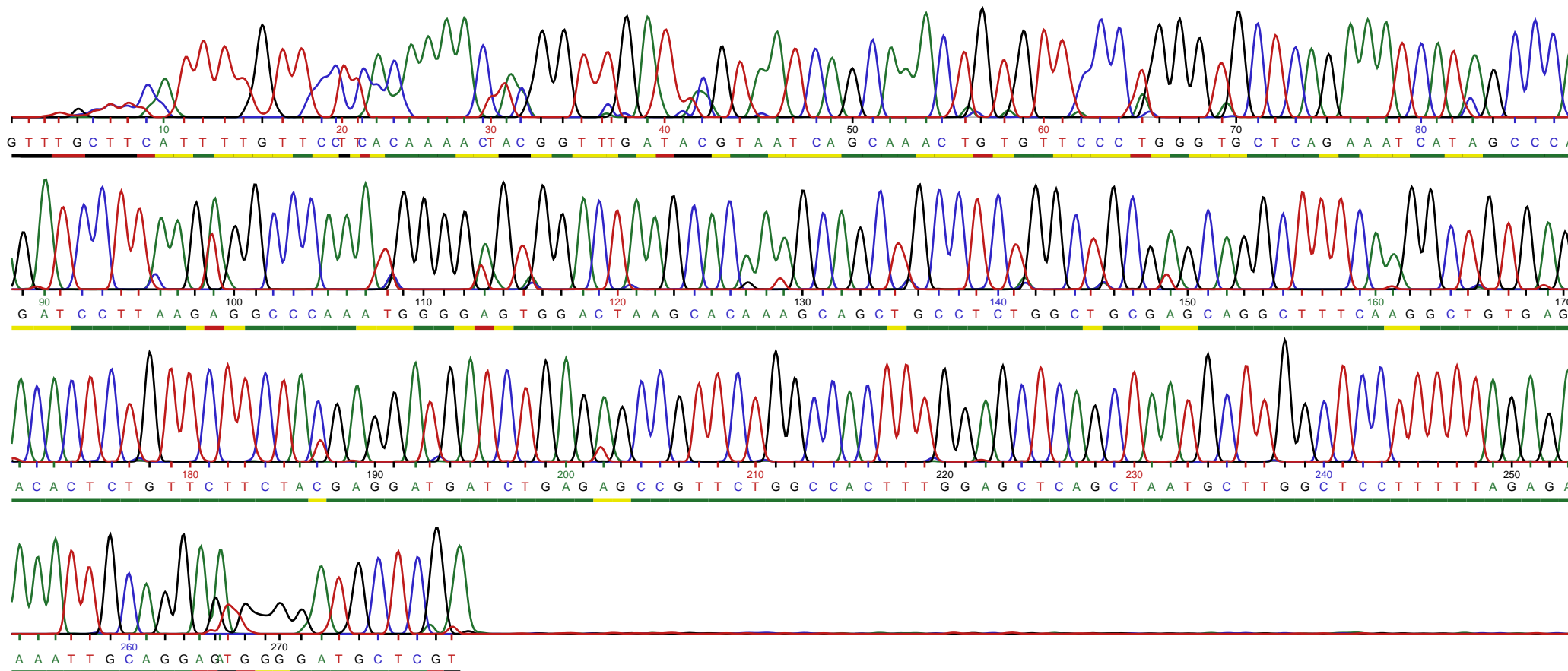

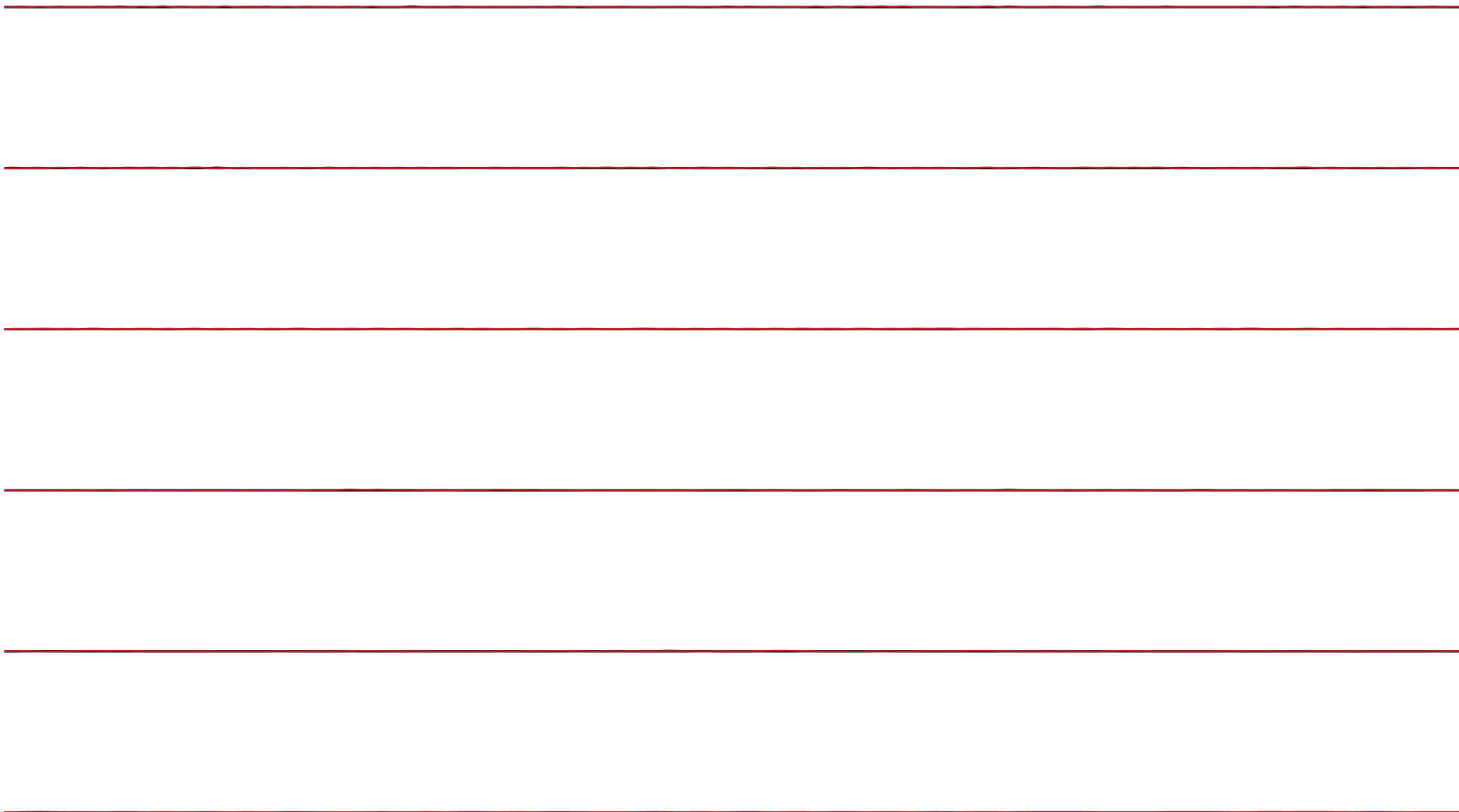

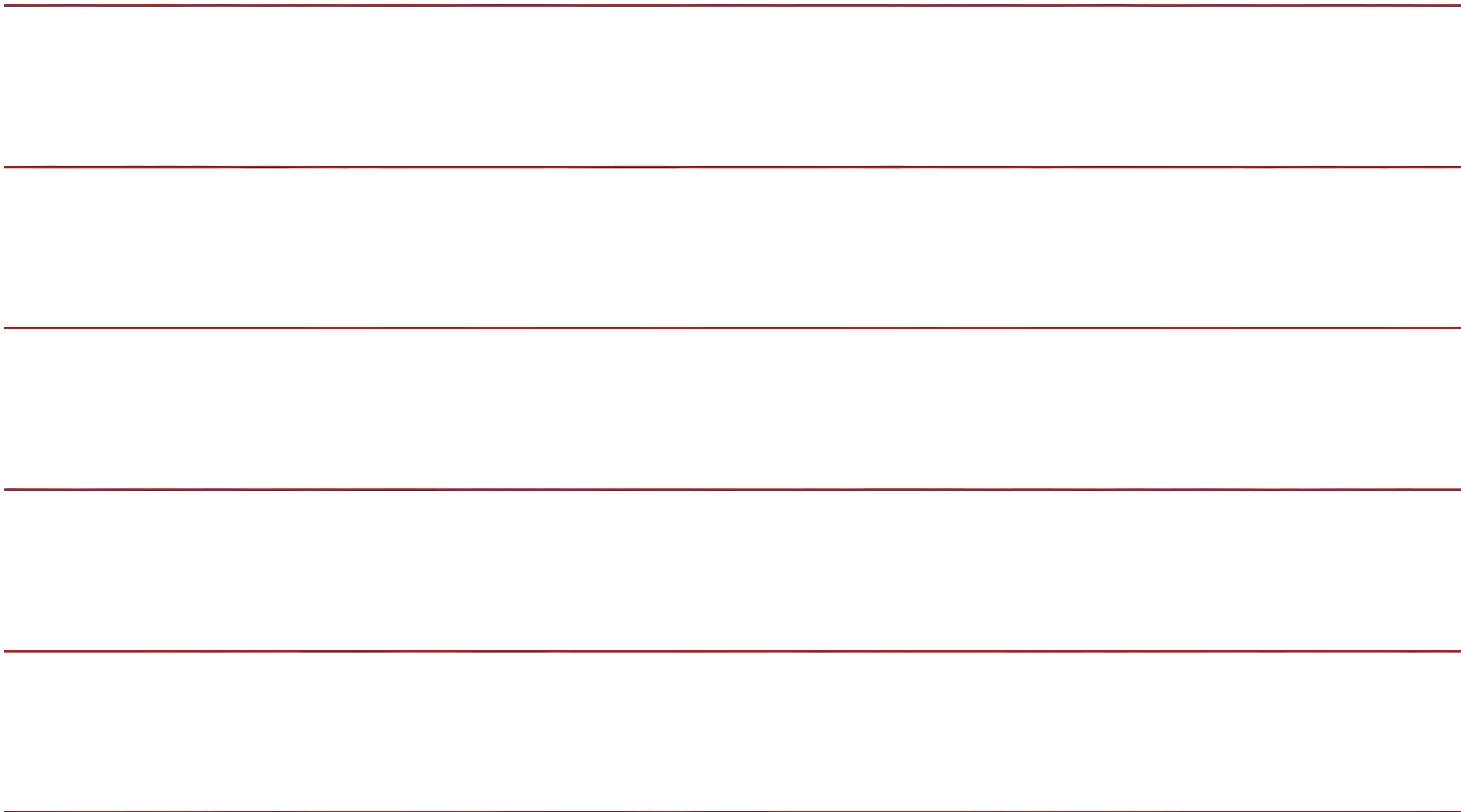

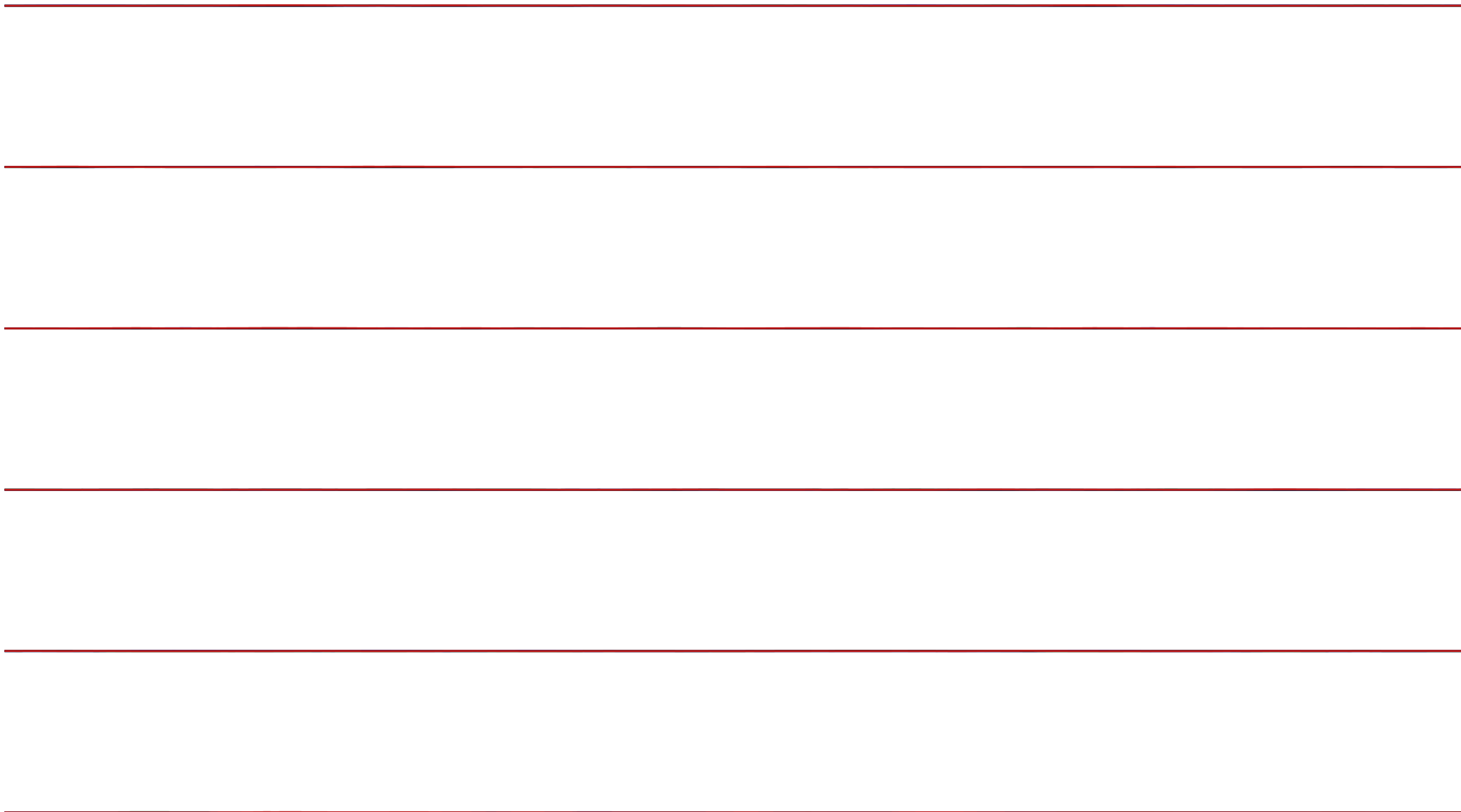

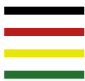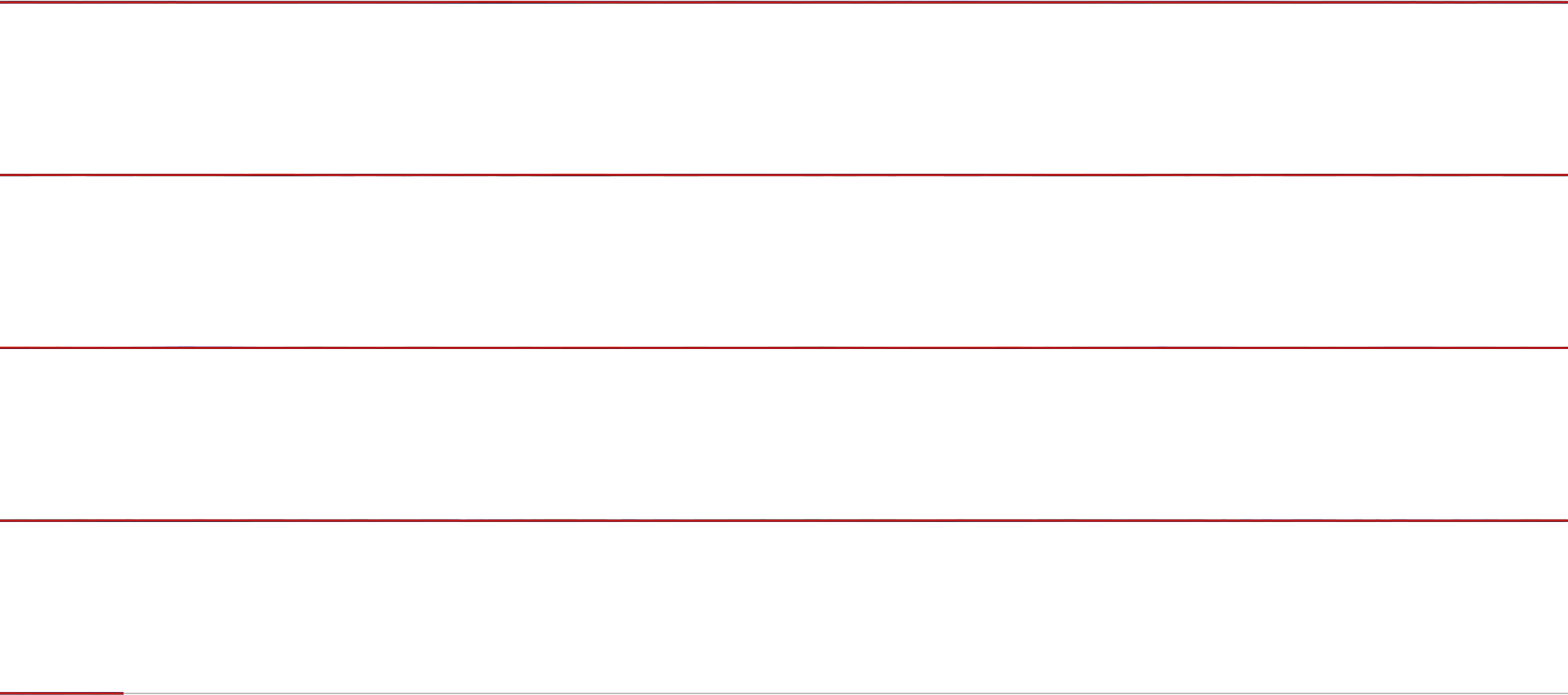

Supplement: Supplementary file 4 — Source data [file 41467_2026_68558_MOESM4_ESM.zip › Source data/Sanger-sequencing data/Suppl.Fig6f/Ori-late-Meg3.pdf]

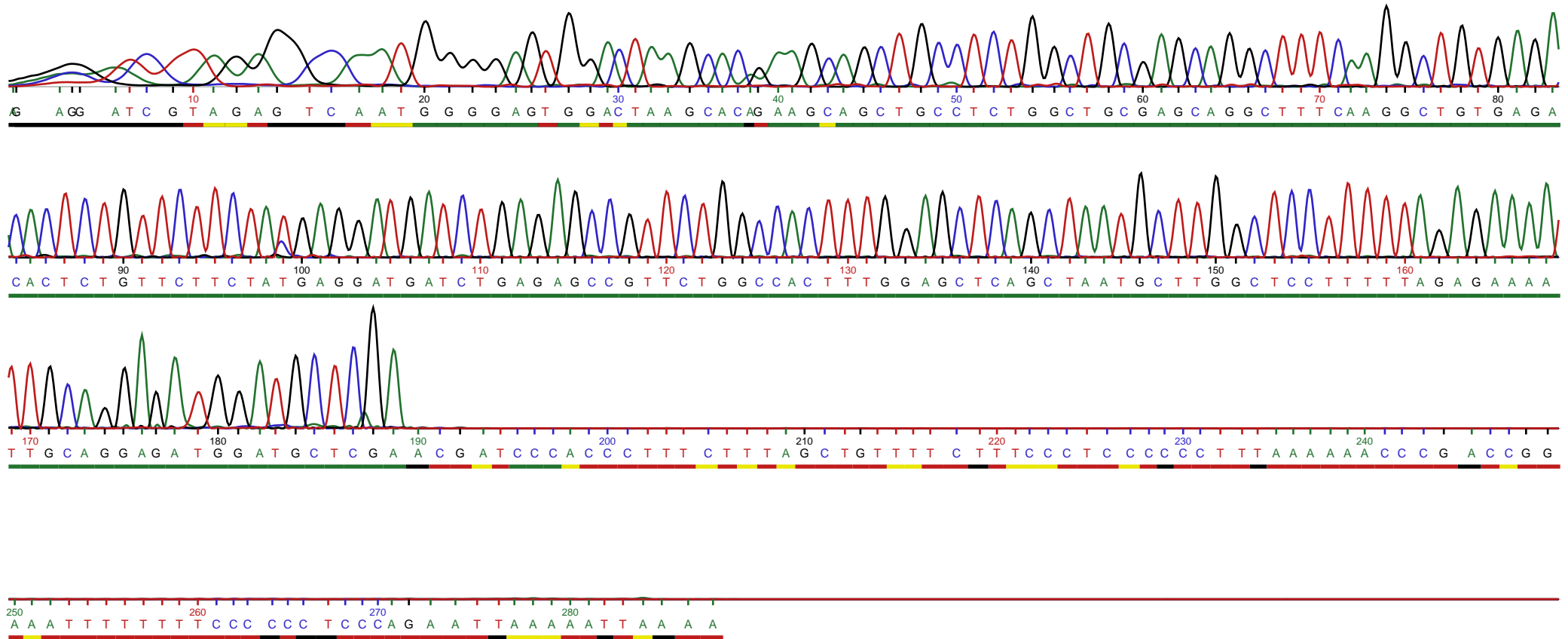

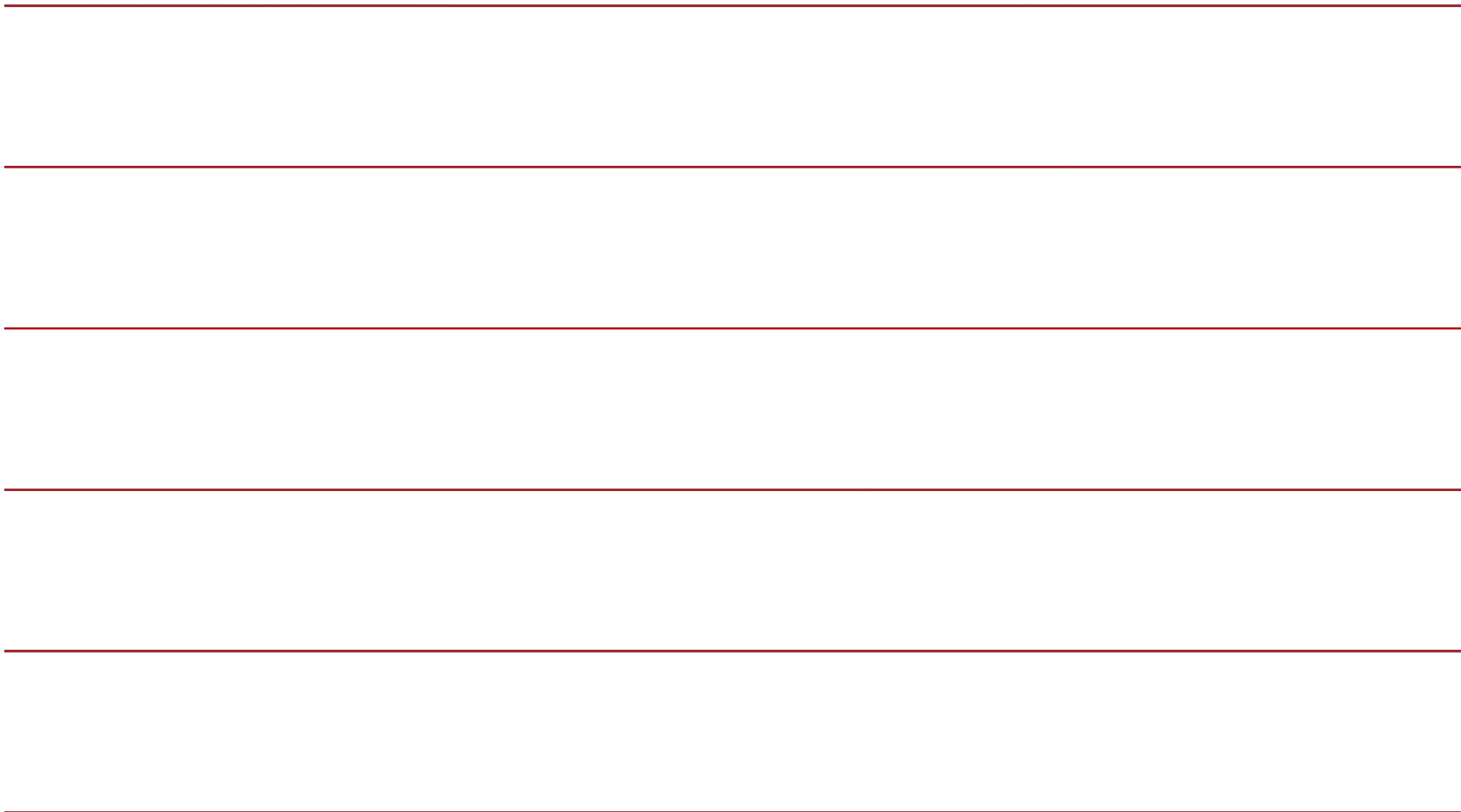

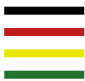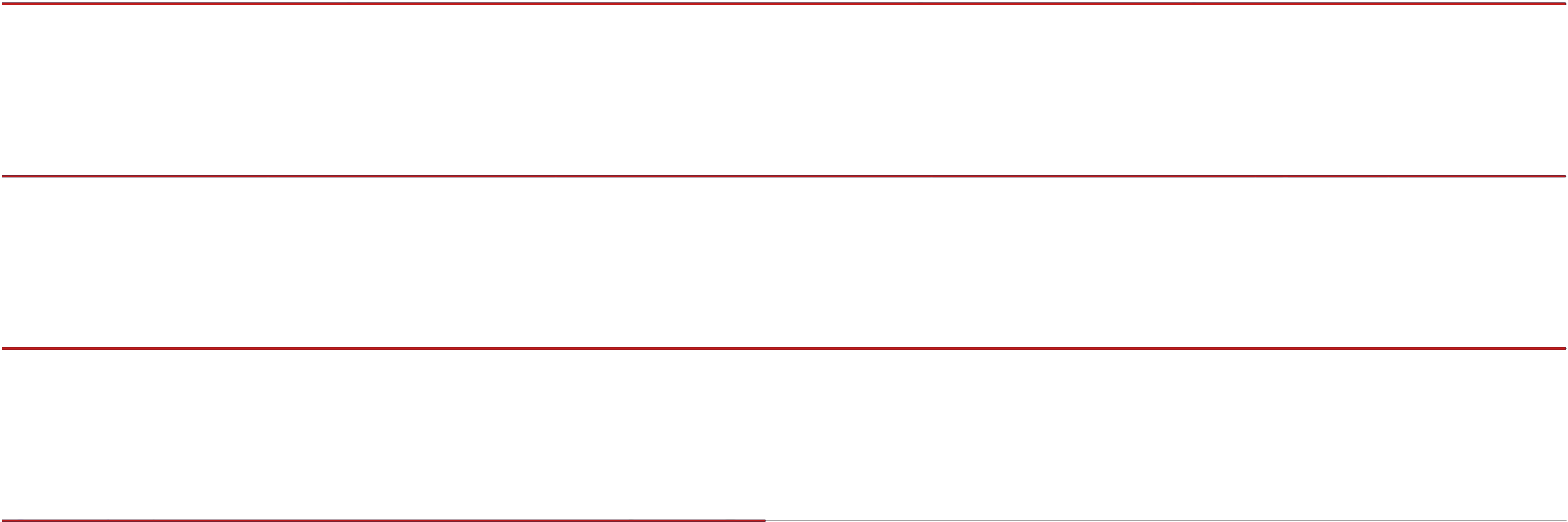

Supplement: Supplementary file 4 — Source data [file 41467_2026_68558_MOESM4_ESM.zip › Source data/Sanger-sequencing data/Suppl.Fig6g/in1-early-Meg3.pdf]

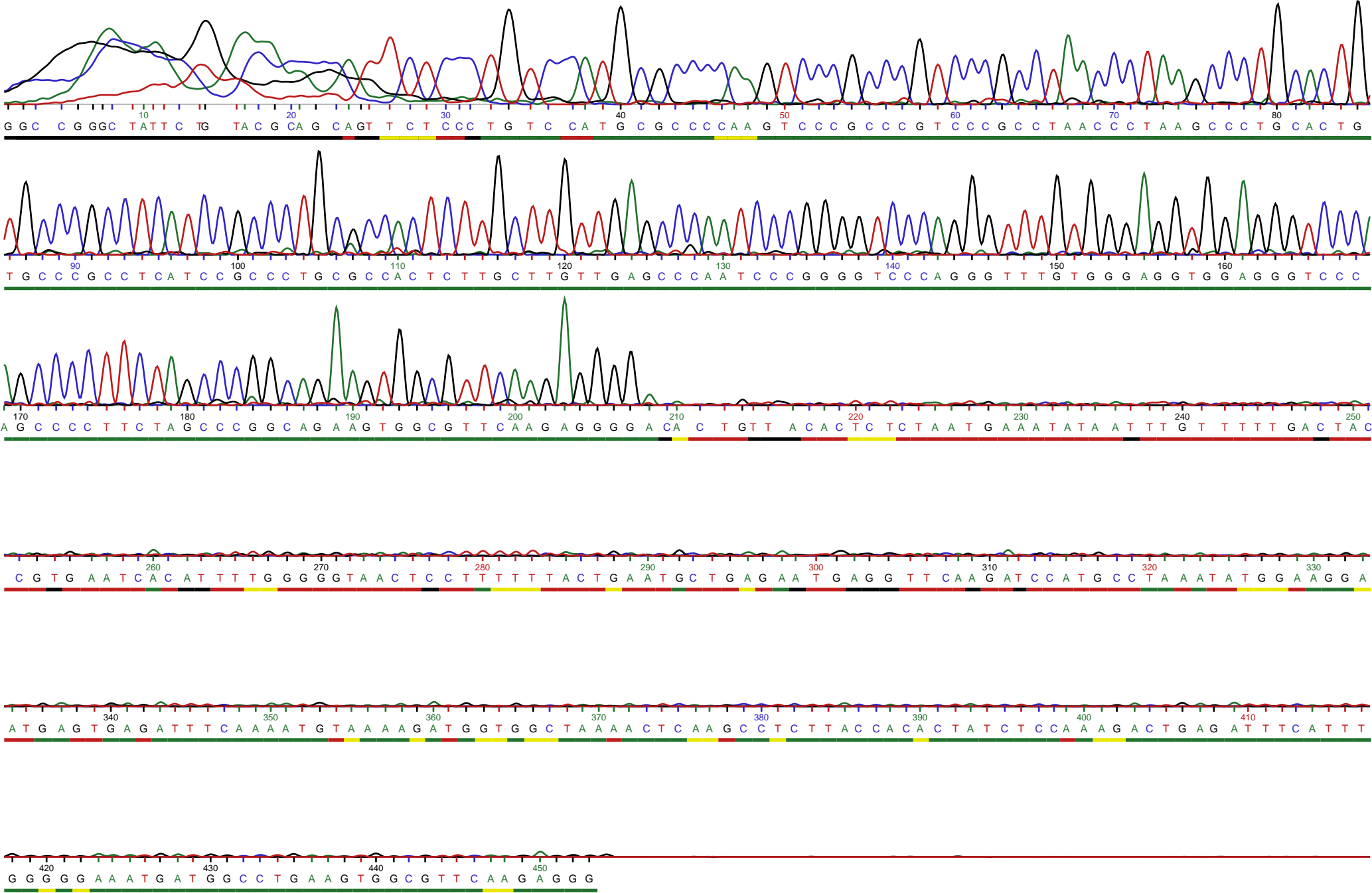

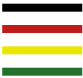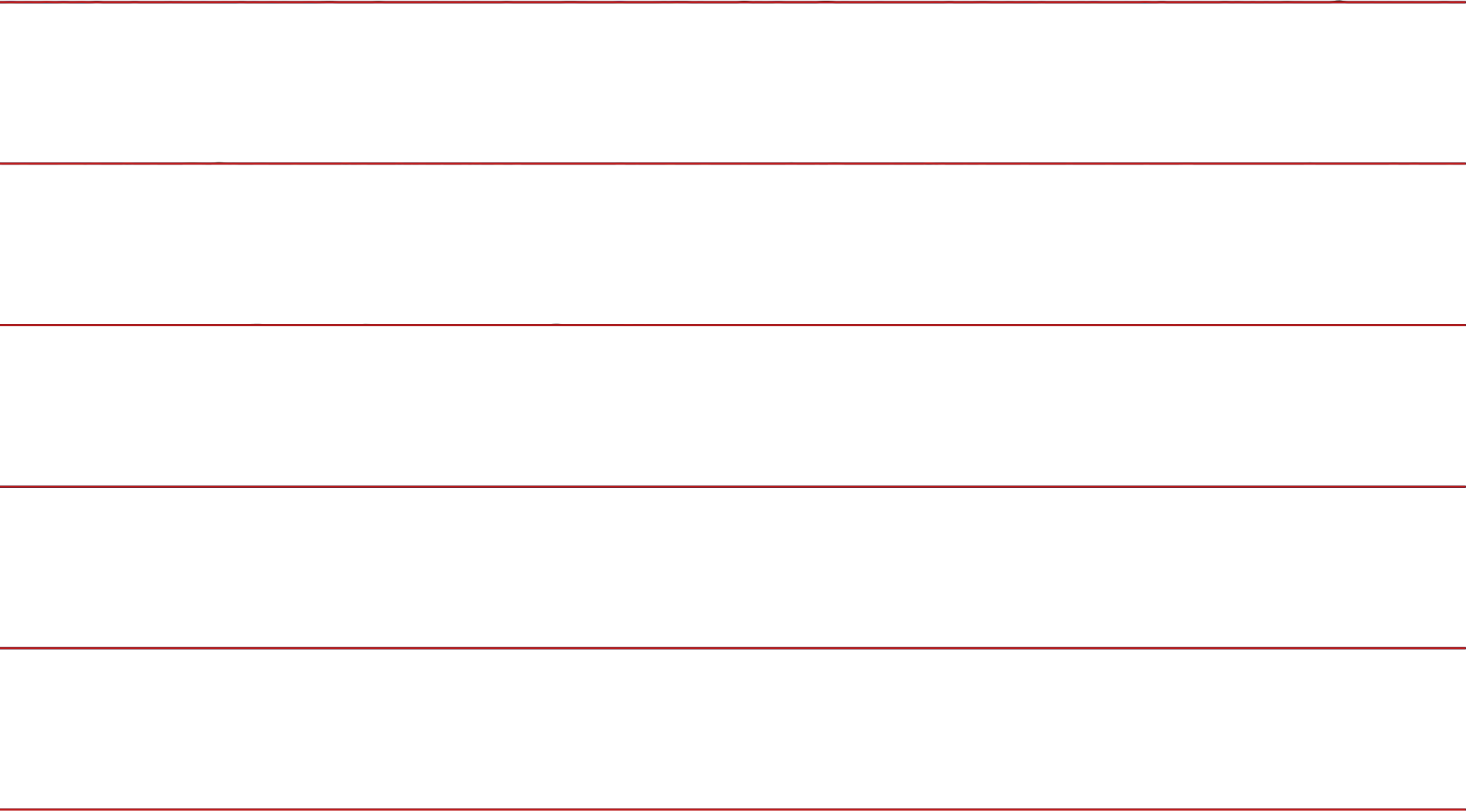

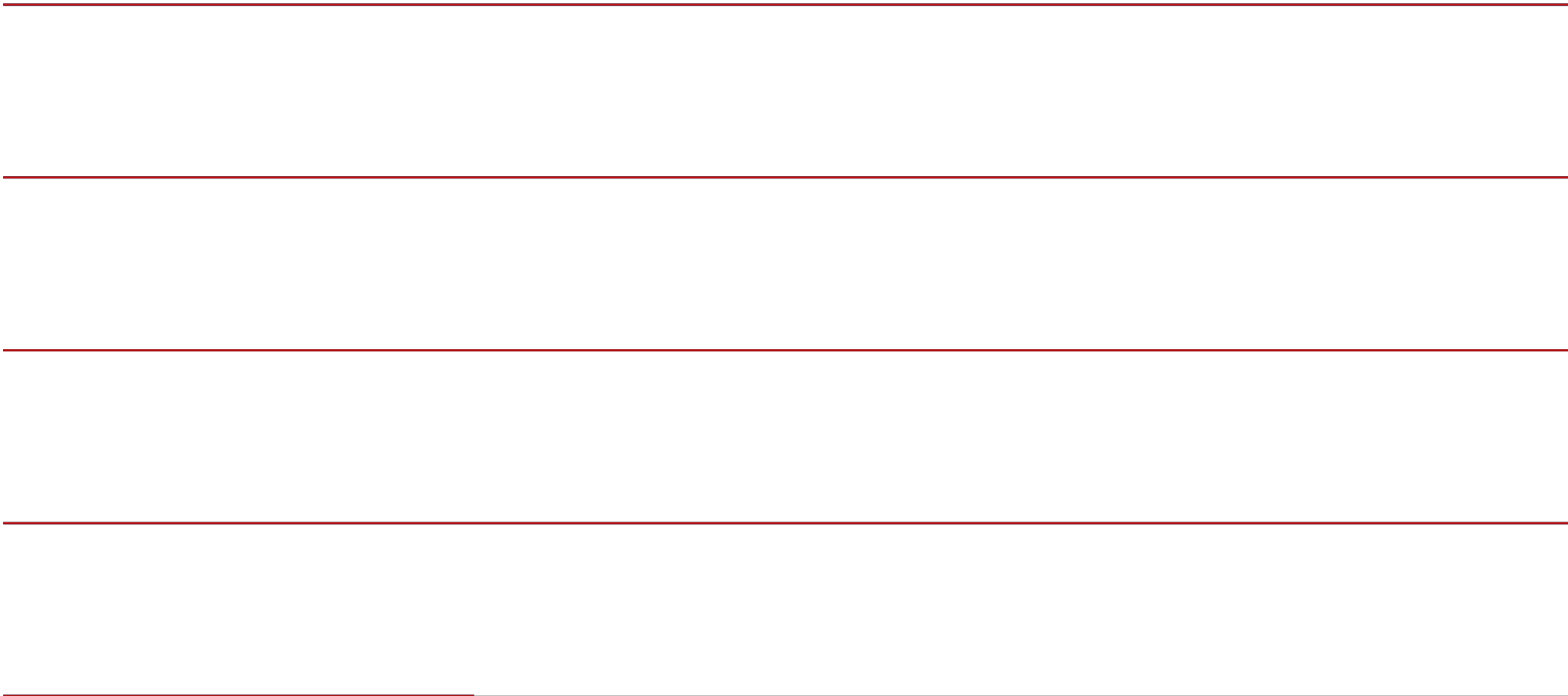

Supplement: Supplementary file 4 — Source data [file 41467_2026_68558_MOESM4_ESM.zip › Source data/Sanger-sequencing data/Suppl.Fig6g/in1-late-Dlk1.pdf]

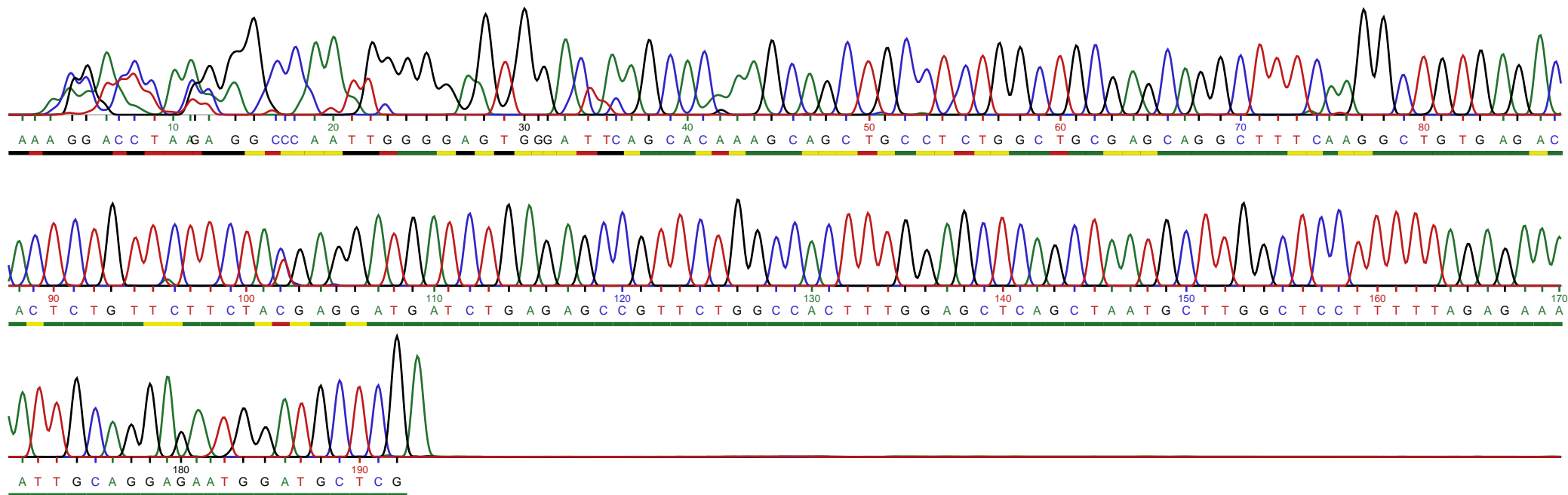

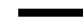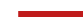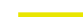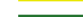

---

---

---

---

---

---

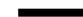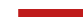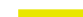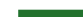

---

---

---

---

---

---

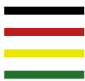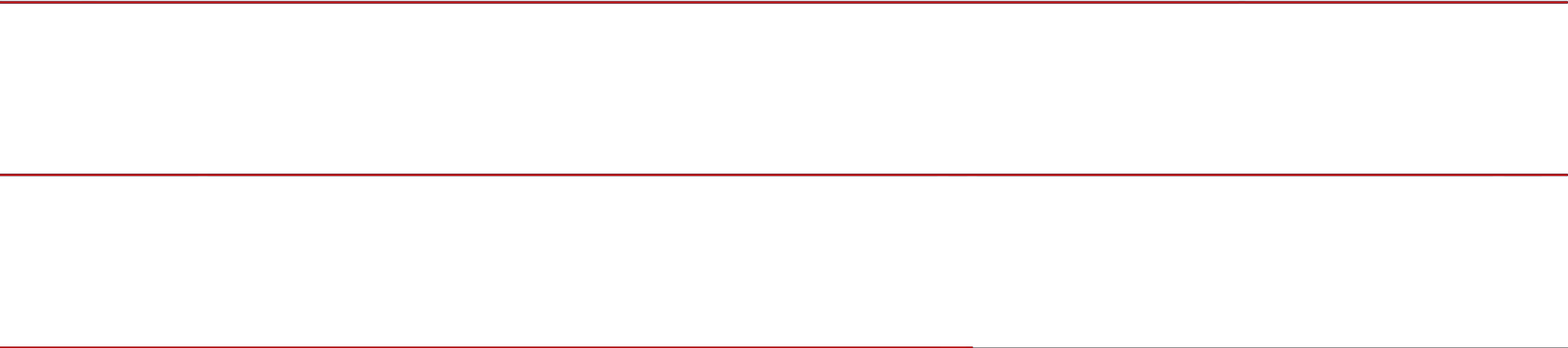

Supplement: Supplementary file 4 — Source data [file 41467_2026_68558_MOESM4_ESM.zip › Source data/Sanger-sequencing data/Suppl.Fig6g/in1-late-Meg3.pdf]

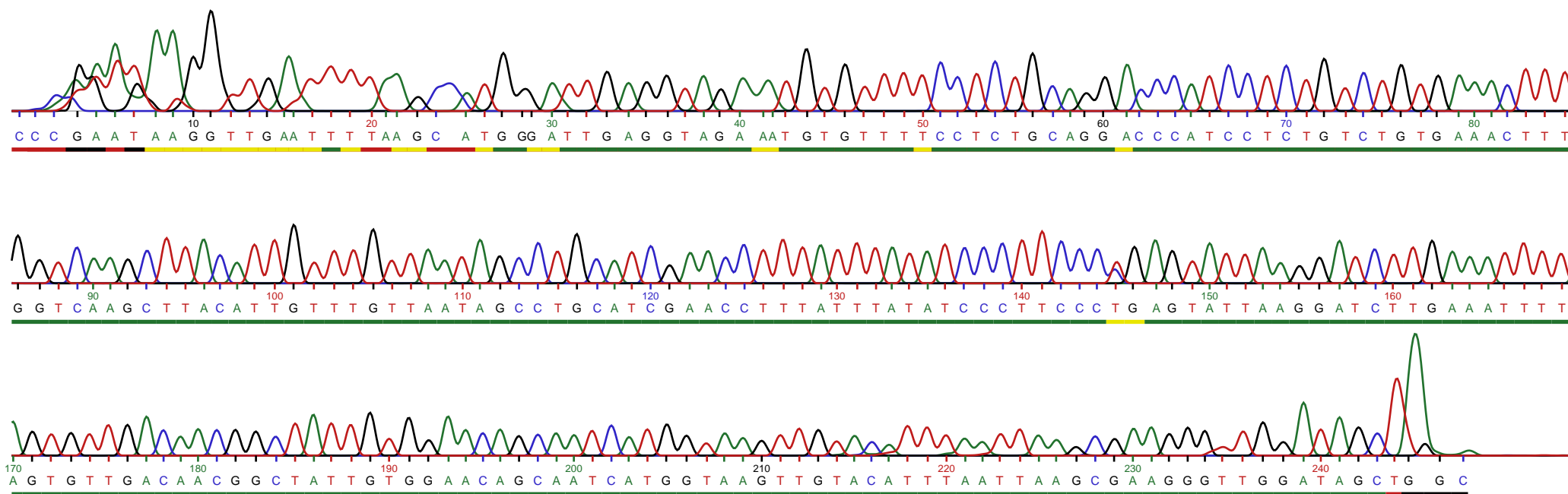

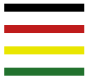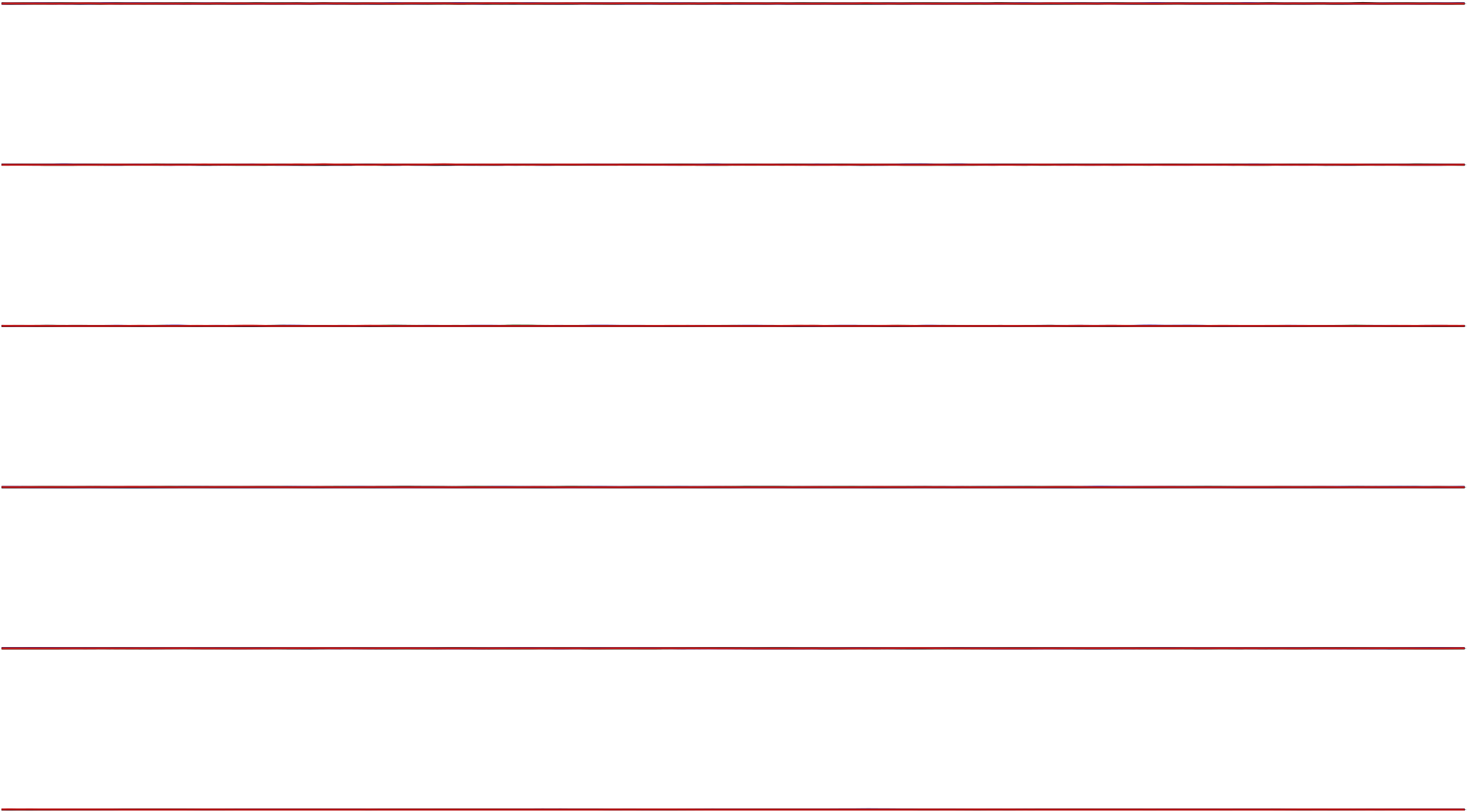

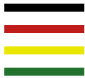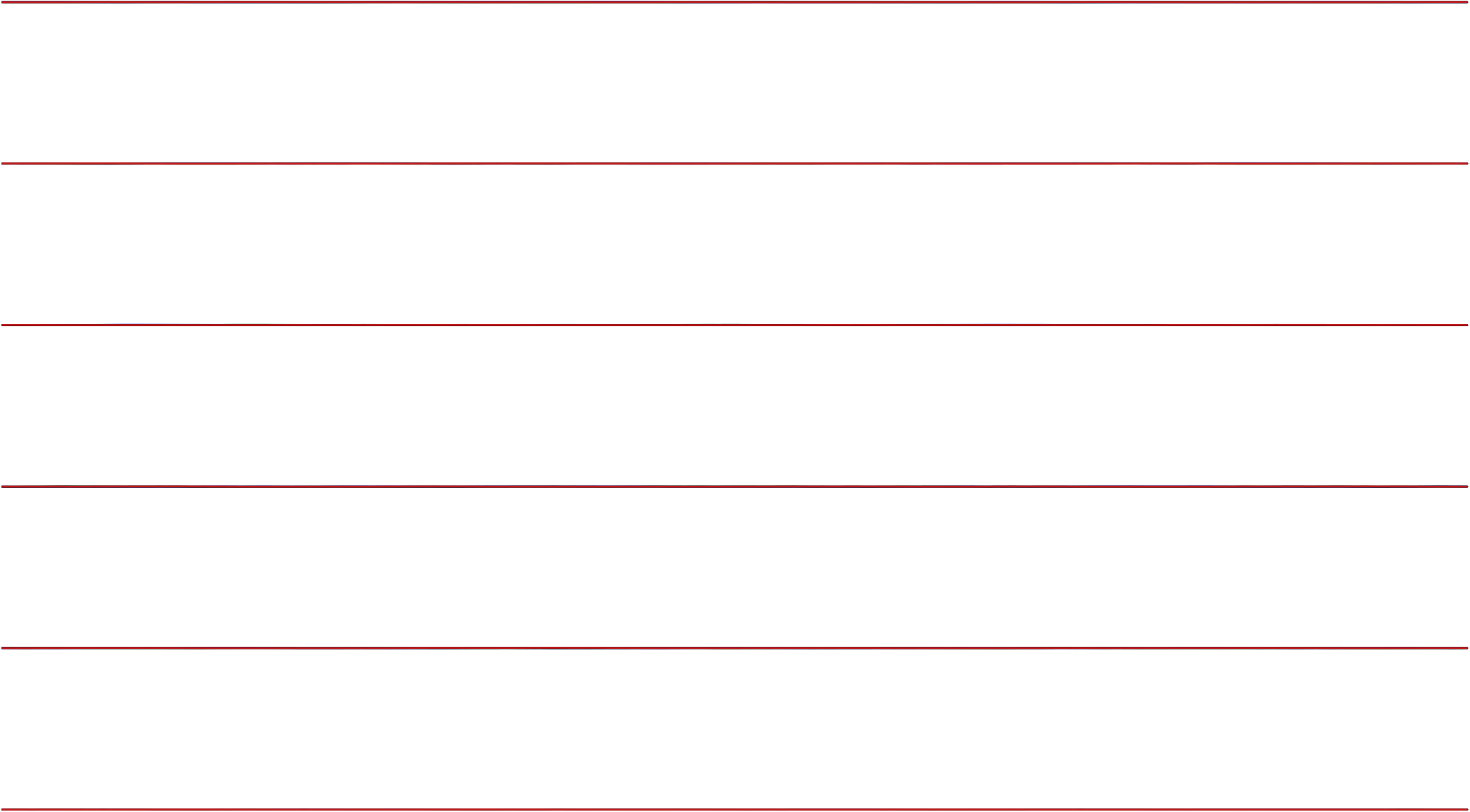

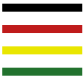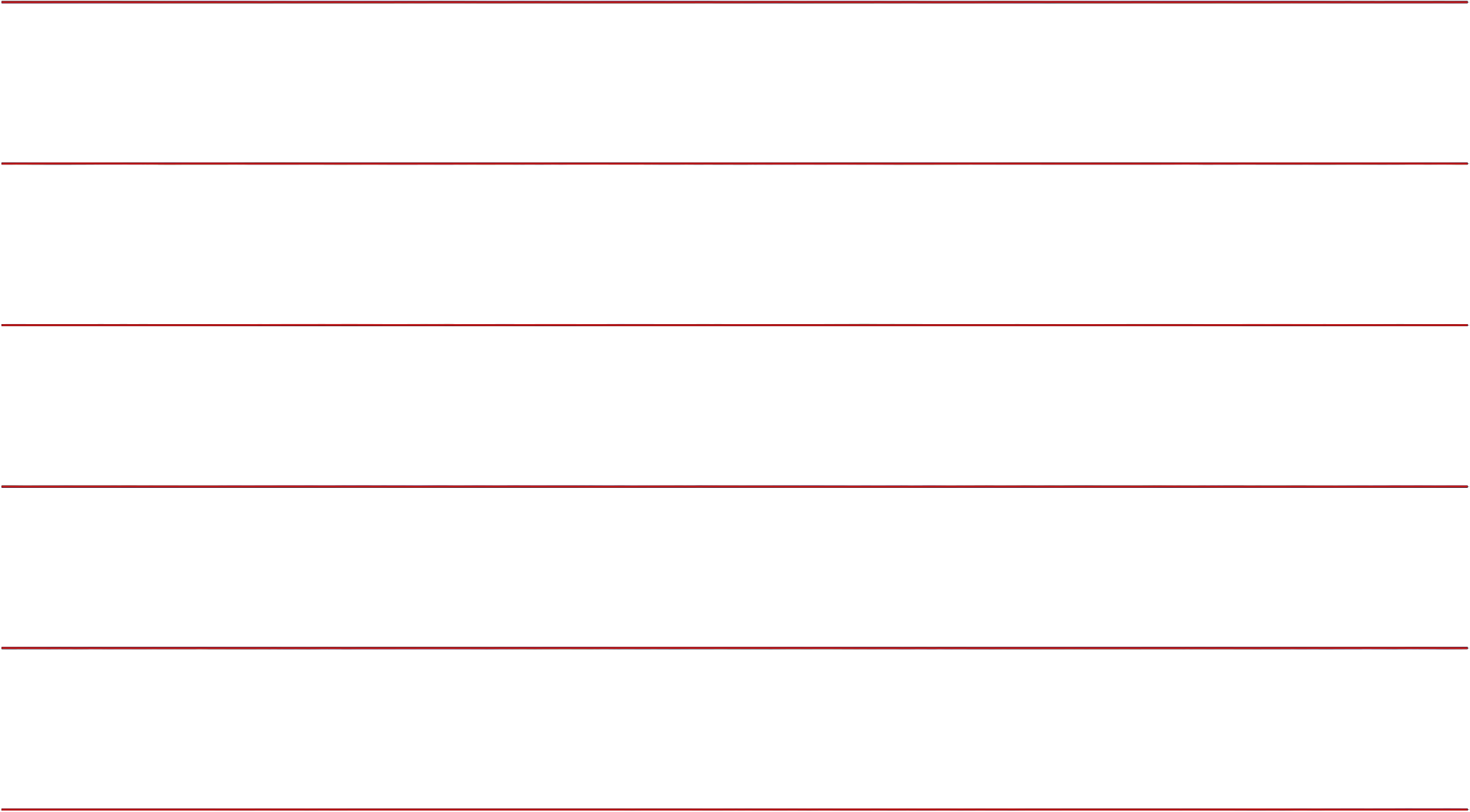

---

---

---

Supplement: Supplementary file 4 — Source data [file 41467_2026_68558_MOESM4_ESM.zip › Source data/Sanger-sequencing data/Suppl.Fig8f/NPC-early-Snrpn.pdf]

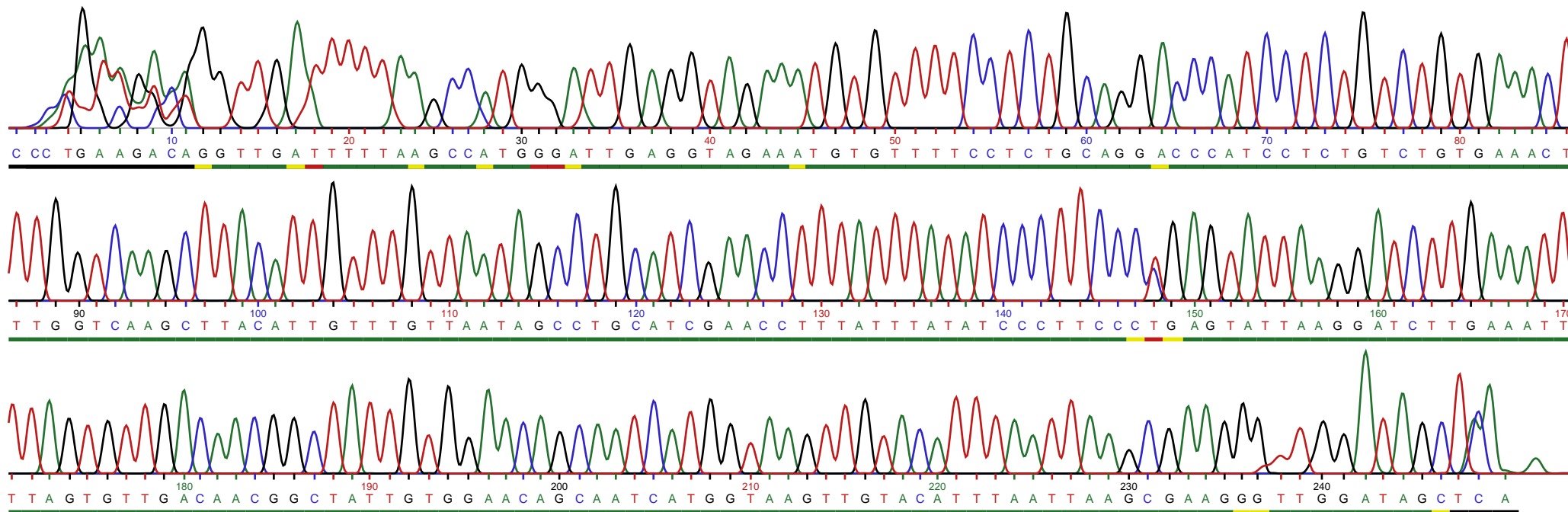

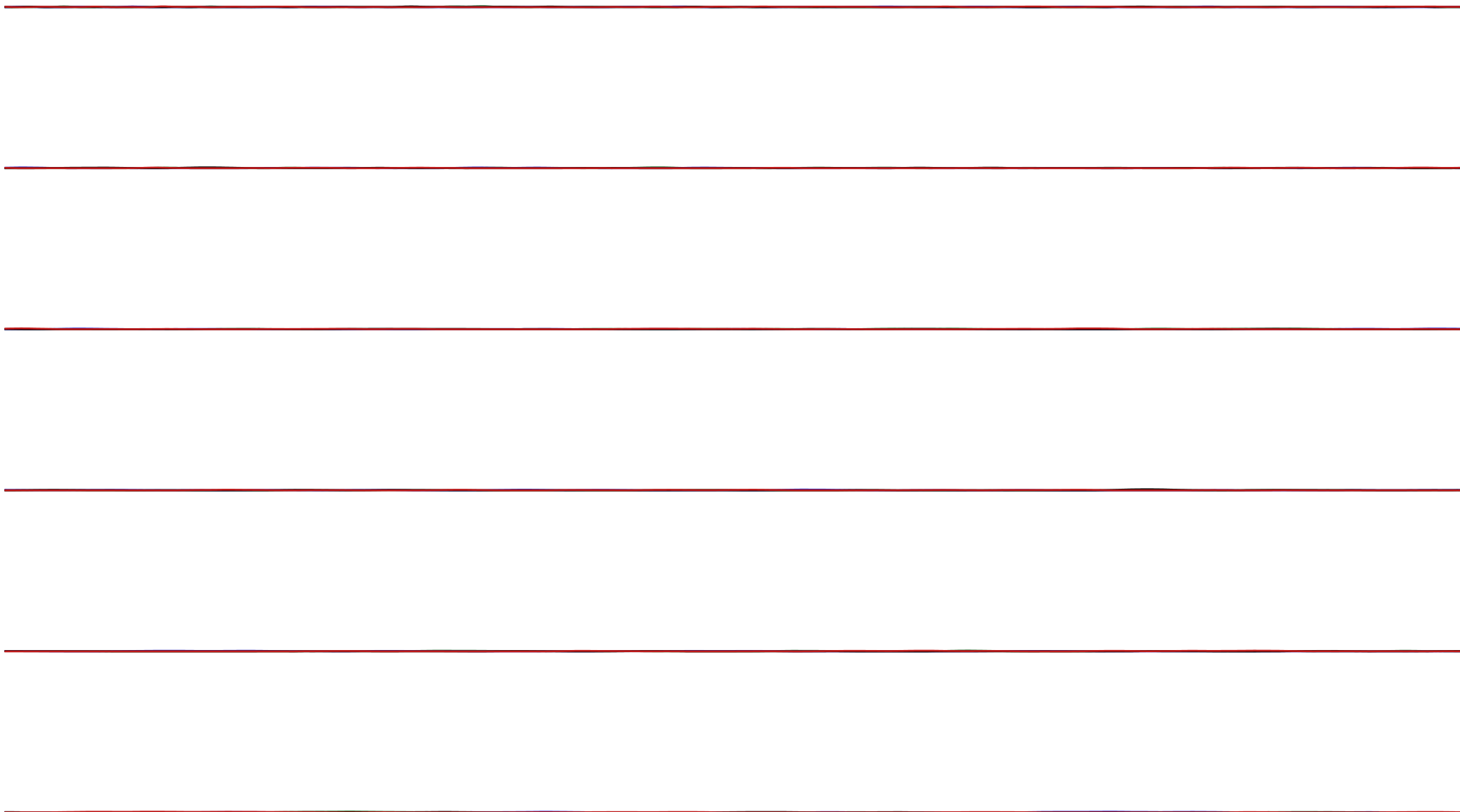

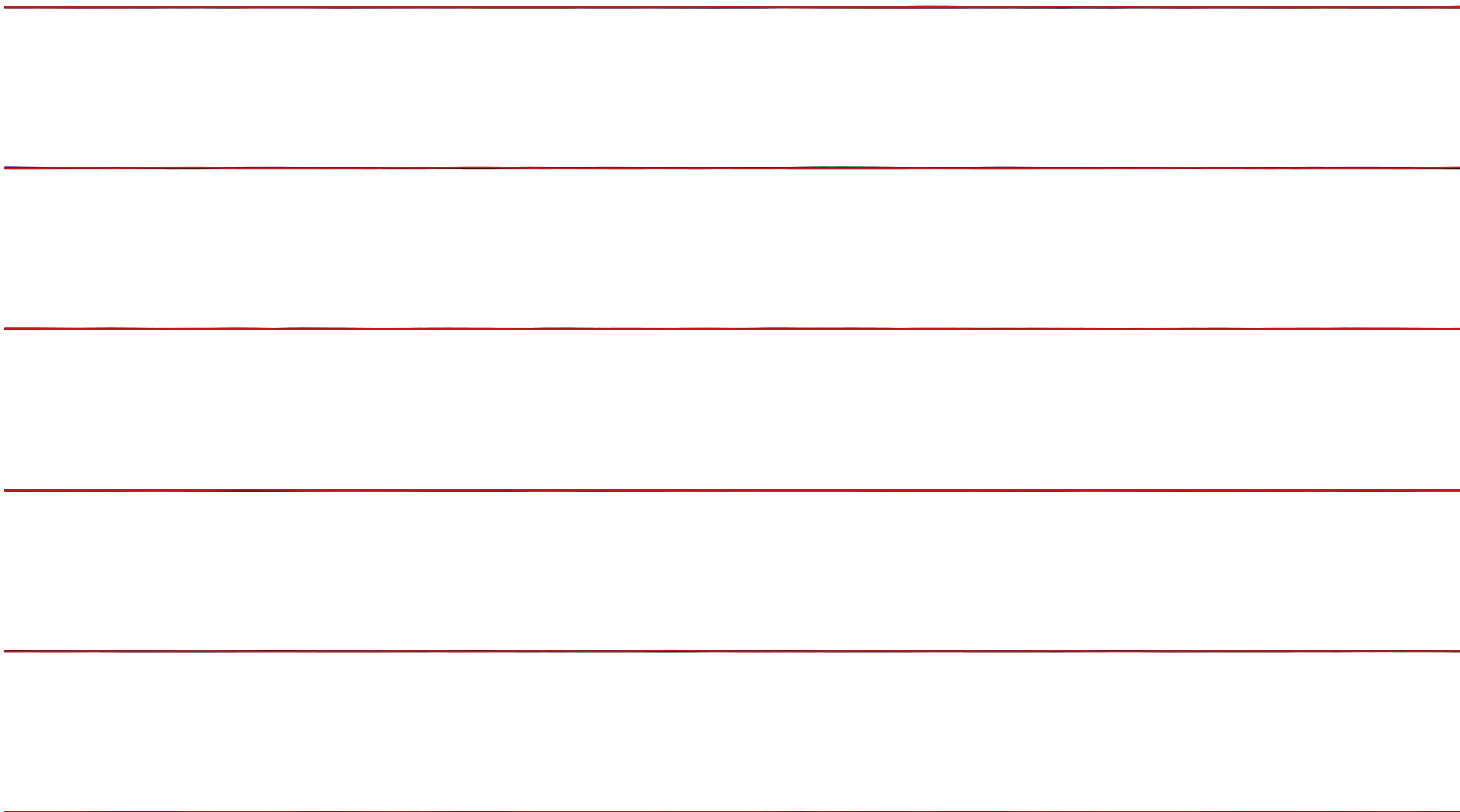

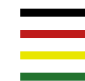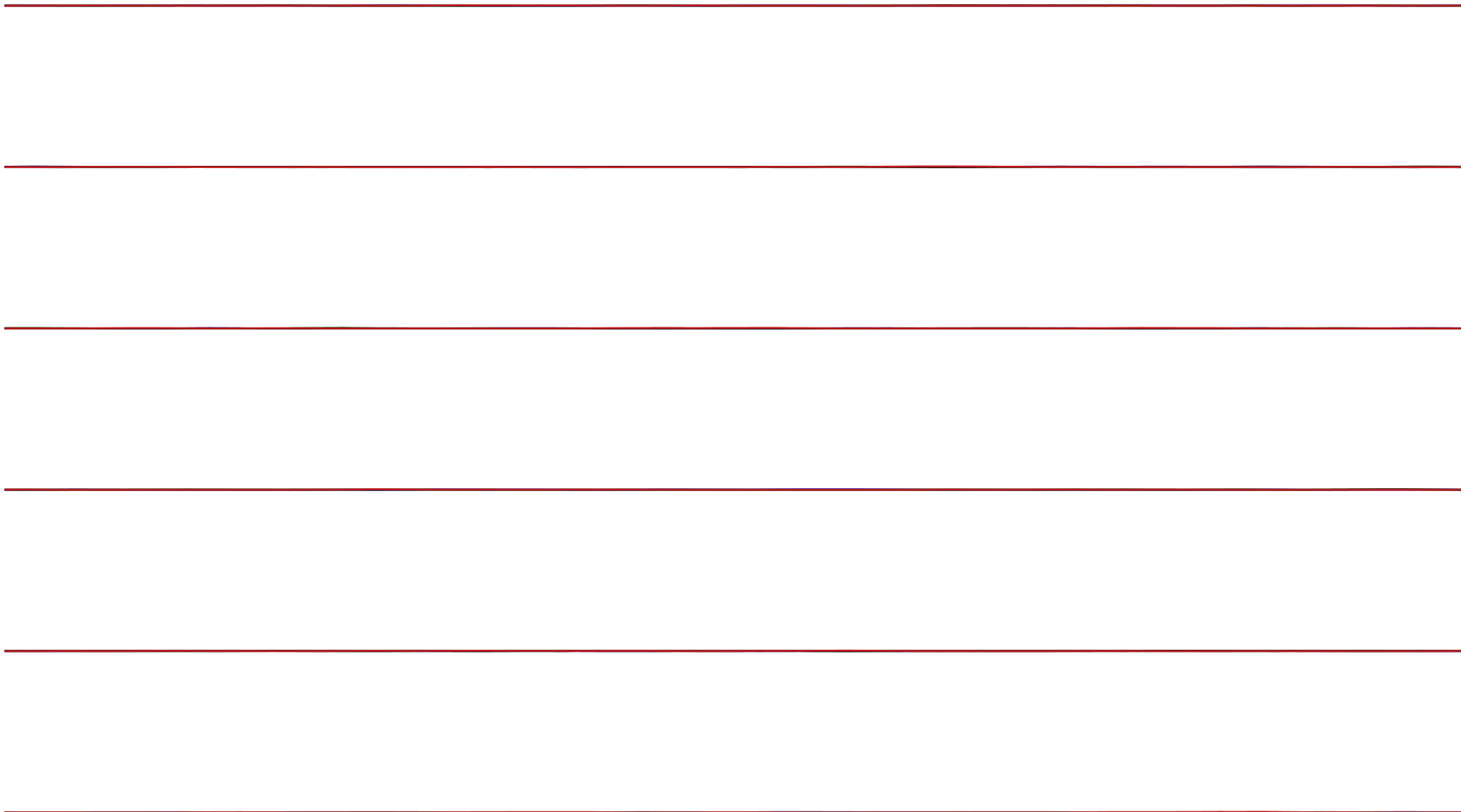

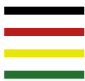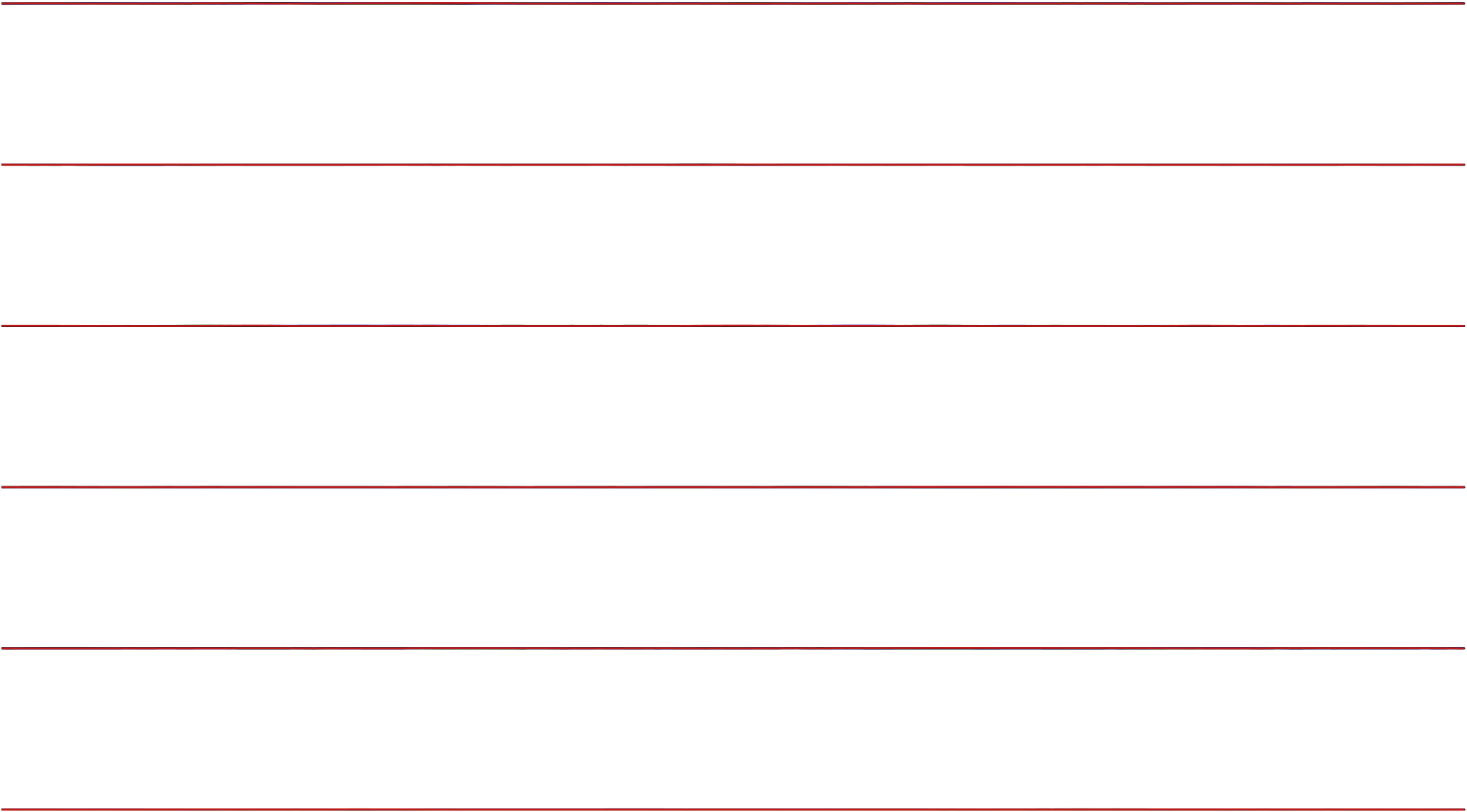

Supplement: Supplementary file 4 — Source data [file 41467_2026_68558_MOESM4_ESM.zip › Source data/Sanger-sequencing data/Suppl.Fig8f/NPC-late-Snrpn.pdf]
